# Proteomic Responses to Alkali Stress in Oats and the Alleviatory Effects of Exogenous Spermine Application

**DOI:** 10.3389/fpls.2021.627129

**Published:** 2021-04-01

**Authors:** Jianhui Bai, Ke Jin, Wei Qin, Yuqing Wang, Qiang Yin

**Affiliations:** ^1^Institute of Grassland Research of Chinese Academy of Agricultural Sciences, Hohhot, China; ^2^Inner Mongolia Technical College of Construction, Hohhot, China

**Keywords:** alkali stress, spermine, oat, proteome, carbohydrate, hormone

## Abstract

Alkali stress limits plant growth and yield more strongly than salt stress and can lead to the appearance of yellow leaves; however, the reasons remain unclear. In this study, we found that (1) the down-regulation of coproporphyrinogen III oxidase, protoporphyrinogen oxidase, and Pheophorbide a oxygenase in oats under alkali stress contributes to the appearance of yellow leaves (as assessed by proteome and western blot analyses). (2) Some oat proteins that are involved in the antioxidant system, root growth, and jasmonic acid (JA) and indole-3-acetic acid (IAA) synthesis are up-regulated in response to alkalinity and help increase alkali tolerance. (3) We added exogenous spermine to oat plants to improve their alkali tolerance, which resulted in higher chlorophyll contents and plant dry weights than in plants subjected to alkaline stress alone. This was due to up-regulation of chitinase and proteins related to chloroplast structure, root growth, and the antioxidant system. Spermine addition increased sucrose utilization efficiency, and promoted carbohydrate export from leaves to roots to increase energy storage in roots. Spermine addition also increased the IAA and JA contents required for root growth.

## Introduction

Of the 1.5 × 10^9^ ha of cultivated land in the world, about 0.34 × 10^9^ ha (23%) has saline soils (containing NaCl) and another 0.56 × 10^9^ ha (37%) has alkaline soils (caused by a mixture of NaHCO_3_ and Na_2_CO_3_) (Xiao et al., 2020). In northeast China, more than 70% of grassland is salt-alkalinized (Wang et al., 2011). Injury to plants due to salt stress is often attributed to ion injury and osmotic stress. Alkali stress, in addition to exerting these two stresses, also leads to high-pH stress (Guo et al., 2010). It has been reported that the limitation on crop growth and development due to alkali stress is more severe than that of salt stress (Zhang et al., 2012). Salt tolerance has been researched for almost 40 years; however, little attention has been paid to alkali stress (Xiao et al., 2020).

Only a few studies related to alkalinity can be summarized. Alkalinity can destroy the membrane structures of roots (Song et al., 2017), disrupt ionic homeostasis, cause reactive oxygen species (ROS) accumulation, and cause lipid peroxidation (Gong et al., 2016). The contents of metal elements (K, Ca, Mg, Fe, Cu, and Zn) are lower in alkali-treated plants, such as rice and wheat (Zhang A. et al., 2016). Several alkali tolerance-related mechanisms have been identified in plants, including improving antioxidant enzyme activity to eliminate ROS (Song et al., 2017), increasing soluble sugar contents to stabilize cellular membranes and facilitate carbon storage (Zhang et al., 2012), and increasing the contents of amino acids and organic acids to facilitate osmotic adjustment (Zhang et al., 2012; Guo et al., 2015). It has been proved that some proteins, such as 14-3-3, can enhance alkali stress tolerance in plants (Zhang et al., 2012).

All the above plant responses to alkali stress are similar to those of salt stress; however, the adverse effects of alkali stress on plants are more severe. For example, the dry mass of cotton shoots and roots decreases under salt and alkali stresses, with greater reductions occurring under alkali stress (Chen et al., 2011). Alkalinity inhibits photosynthesis, stomatal conductance, and transpiration rates more severely than salinity (Chen et al., 2011). Greater limitations on germination rate and yield have been observed under alkali stress than under salt stress in oats (Bai et al., 2018). However, the mechanisms of alkali toxicity are not fully understood.

Our previous study on oats showed that alkalinity caused the appearance of yellow leaves and decreased chlorophyll contents more than salinity (Bai et al., 2018). He et al. (2017) also found that alkali stress caused yellow leaves in rice. A total of 90%–95% of crop biomass is generated from photosynthesis (Wang H. et al., 2016) and chlorophyll is the main photosynthetic pigment. We hypothesized that decreases in chlorophyll content caused by alkalinity may lead to decreases in photosynthesis that limit crop growth and yield. However, very little is known about the molecular mechanisms of the effects of alkalinity on chlorophyll metabolism.

Polyamines (PAs) are essential for plant growth and development, and play important roles in improving the tolerance to adverse environmental conditions. Triamine spermidine (Spd), putrescine (Put), and tetraamine spermine (Spm) are the three main forms of PAs. Spm has proven to be the most effective PA for improving the drought tolerance of plants (Talaat et al., 2015; Chen et al., 2019). It has been reported that transgenic plants overexpressing *S*-adenosyl-*L*-methionine synthetase (*SlSAMS1*) showed a significant increase in tolerance to alkali stress. *SlSAMS1* mainly functions in the accumulation of Spd and Spm in transgenic lines (Gong et al., 2014). Exogenous spermine increased the chlorophyll contents of *Malus hupehensis* Rehd. under alkali stress (Gong et al., 2018). However, the physiological and molecular mechanisms of increased alkali tolerance due to spermine application have not been elucidated completely.

Proteins participate directly in biological processes and the key signaling networks in plants (Wang J. et al., 2016). Due to post-transcriptional events and post-translational modifications, transcriptome data do not always correlate with protein expression levels; therefore, it is essential to detect protein expressions directly (Jiang et al., 2017). Proteomic analysis is an effective method for exploring global protein expression and can provide large amounts of information related to individual proteins involved in specific biological responses (Li et al., 2015). The TMT-based proteomic technique was used in this study. Oats, which are grown throughout the world, are important feed, grain, cover, and rotation crops (Bai et al., 2017). In this research, the proteomic and physiological mechanisms involved in alkalinity responses and the alleviatory effects of exogenous spermine were studied. The main objectives of this study were to (1) identify the proteins related to alkali injury and decreased chlorophyll accumulation due to alkalinity, (2) identify the proteins and physiological mechanisms related to alkali tolerance in oats, (3) identify the proteins and physiological processes related to improved alkali tolerance due to exogenous spermine application, and (4) provide a reference for oat cultivation in alkaline soils and breeding of alkaline-tolerant genotypes.

## Materials and Methods

### Plant Materials and Treatments

This study was conducted in a greenhouse at the Institute of Grassland Research of the Chinese Academy of Agricultural Sciences in Hohhot, China, during the years 2017 to 2019.

**FIGURE 1:**
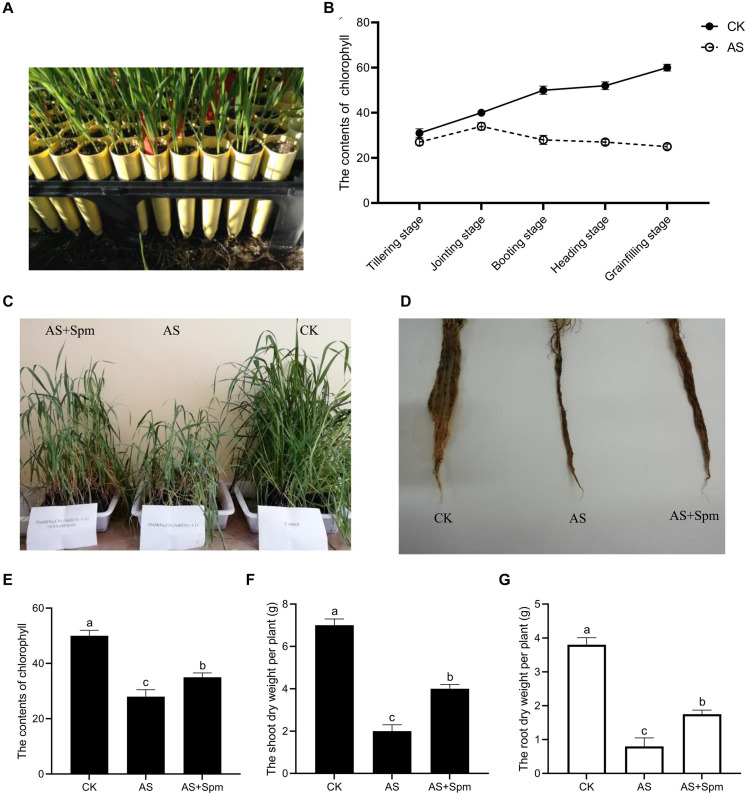
Effects of exogenous spermine on oat growth under alkali stress at booting stage. **(A)** The cone-tainer planting method. **(B)** Effects of alkali stress on chlorophyll contents at varied stages. **(C)** Image related to the effects of exogenous spermine on shoot growth under alkali stress at booting stage. **(D)** Image related to the effects of exogenous spermine on root growth under alkali stress at booting stage. **(E)** Effects of spermine on chlorophyll contents under alkali stress at booting stage. **(F)** Effects of spermine on shoot dry weight per plant under alkali stress at booting stage. **(G)** Effects of spermine on root dry weight per plant under alkali stress at booting stage. CK, control; AS, alkali stress (35 mmol.L^–1^ Na_2_CO_3_:NaHCO_3_ = 1:1); AS + Spm, 35 mmol.L^–1^ Na_2_CO_3_:NaHCO_3_ + 0.01 mmol.L^–1^ spermine.

Oat cultivar OA1414-2 plants were planted in 16 cm-tall plastic cone-tainers filled with vermiculite (Figure 1A) with an approximate volume of 170 cm^3^ (one plant per cone-tainer). One plastic rack could hold 30 cone-tainers. One plastic rack was placed in a 30 L sterilite container with hoagland solution to keep the plants moist. Each cone had two holes at the bottom, allowing the plants to take up water and nutrients from the sterilite container.

At the three-leaf stage, a total of three treatments were created: (a) control, (b) alkali treatment, and (c) alkali + spermine treatment (Supplementary Table 1). The pH value of the alkali stress used in this study was 9.93. For each treatment type, 10 L solutions were added to the bottom of separate sterilite containers. Each sterilite container represented a treatment, and each cone represented a replication within the treatment. There were three replicates.

### Determination of Chlorophyll Content and Dry Weight

When 90% of the oat plants reached the tillering, jointing, booting, heading, and grain-filling stages, we used a non-destructive portable soil-plant analysis development meter (SPAD-502, Konica-Minolta, Japan) to measure chlorophyll content. The SPAD value of the same second leaf of each plant was recorded. Three replicates were examined for each treatment.

At the booting stage, oat plants from all three treatments were taken from the cones. The shoots and roots from each oat plant were dried at 105°C for 0.5 h and then at 85°C for 24 h, then weighed by an electronic balance.

### Indoleacetic Acid (IAA), Amino-Cyclopropane-1-Carboxylic Acid (ACC), Abscisic Acid (ABA), and Jasmonate Acid (JA) Contents

Leaves or roots were homogenized into powder using liquid nitrogen. Samples (50 mg) were extracted with a solution of methanol:water:formic acid = 15:4:1 (v:v:v:) at 4°C. For quantification of ABA, IAA, ACC, and JA, internal standards were added. The homogenate was centrifuged at 6000 × *g* for 10 min at 4°C. The pellets were re-extracted with 100 μL 80% aqueous methanol (v/v), and the solution was filtrated with a 0.22 μm PTFE filter.

Separation of ABA, IAA, ACC, JA in the extracts was carried out by HPLC on a reverse-phase C18 column (Waters Acquity UPLC HSS T3, 1.8 μm, 2.1 mm × 100 mm) using a linear gradient: 0 min water/acetonitrile (95:5 V/V), 1 min water/acetonitrile (95:5 V/V), 8 min water/acetonitrile (5:95 V/V), 9 min water/acetonitrile (5:95 V/V), 9.1 min water/acetonitrile (95:5 V/V), and 12 min water/acetonitrile (95:5 V/V). The flow rate was 0.35 mL.min^–1^. The extracts of ABA, IAA, ACC, and JA were further quantified and qualified by tandem mass spectrometry (MS/MS). The MWDB Metware Database was used to qualitatively determine the ABA, IAA, ACC, and JA contents. The parameters related to qualitative analysis are listed in the Supplementary Materials and Methods 1. The calibration curve method contributed to the quantitative analysis. We took the standard concentration in the range 0.01–500 ng.mL^–1^ as the abscissa of the calibration curve and the peak area as the ordinate.

The internal standard method was used to quantify the contents of ABA, IAA, ACC, and JA. Eleven levels of ABA, IAA, ACC, and JA standard concentrations were set (0.01 ng.mL^–1^, 0.05 ng.mL^–1^, 0.1 ng.mL^–1^, 0.5 ng.mL^–1^, 1 ng.mL^–1^, 5 ng.mL^–1^, 10 ng.mL^–1^, 50 ng.mL^–1^, 100 ng.mL^–1^, 200 ng.mL^–1^, 500 ng.mL) to acquire data corresponding to the mass spectrum peak. A calibration curve was drawn and the internal standard concentration was regarded as the abscissa, while the peak area was regarded as the ordinate. The ABA, IAA, ACC, and JA contents were estimated according to the calibration curve.

### Glucose, Sucrose, Fructose, Starch, and Soluble Sugar Contents

The contents of glucose, sucrose, and fructose were determined by the high-performance liquid chromatography (HPLC) method, as described in Zushi and Matsuzoe (2006). The starch contents were determined according to the method of Buysse and Merckx (1993). The soluble sugar contents were assayed by the anthrone method (Yemm and Willis, 1954).

### Antioxidant Enzyme and MDA Contents

Oat leaves or roots (0.5 g) were ground into powder with liquid nitrogen, then we added 5 mL potassium phosphate buffer (0.05 mmol.L^–1^, pH 7.8, 10 mL) containing 2% (w/v) polyvinylpolypyrrolidone, 3 mmol.L^–1^ 2-mercaptoethanol, and 1 mmol.L^–1^ EDTA. The samples were centrifuged at 13,000 × *g* for 20 min at 4°C. The supernatant was used for measurement of the SOD, POD, and CAT activity. SOD activity was determined by the nitroblue tetrazolium (NBT) method and assayed by spectrophotometrically monitoring the ability to inhibit the photochemical reduction of nitroblue tetrazolium at 560 nm. The assay solution contained 0.1 mL enzyme extract, 2 mL 15 mmolL^–1^ methionine, 2 mL 0.1 mmolL^–1^ NBT, 0.1 mL 0.1 mmolL^–1^ riboflavin, and 0.1 mL 0.003 mmolL^–1^ ethylenediaminetetraacetic acid (EDTA). POD activity was assayed by monitoring the increase in absorption at 460 nm in 3 mL of reaction solution containing 50 mmol.L^–1^ potassium phosphate buffer (pH 6.0), 200 mmol.L^–1^ H_2_O_2_, 25 mmol.L^–1^ guaiacol, and initiating this reaction by adding 1 mL enzyme extract. CAT activity was assayed by monitoring the consumption of H_2_O_2_ at 240 nm. Some 0.2 mL of the above crude enzyme extract was used to initiate the reaction in 2.7 mL of a reaction solution containing 1.5 mL of 50 mmol.L^–1^ sodium phosphate buffer (pH 7.8), 0.3 mL of 100 mmol.L^–1^ H_2_O_2_, and 1.0 mL of deionized water.

The determination of MDA content was performed as follows: 0.5 g of leaves or roots was ground into powder with liquid nitrogen, then 5 mL of 10% trichloroacetic acid and 0.3% of thiobarbituric acid were added. The mixture was boiled for 30 min and centrifuged at 10,000 × *g* for 20 min. The absorbance of the supernatant was measured at 532 nm and 600 nm.

### Proline and Total Flavonoid Contents

Proline contents were assayed as follows: 0.5 g of leaves and roots were ground into powder with liquid nitrogen, then 5 ml of 3% sulfosalicylic acid was added. The samples were boiled for 10 min, placed on ice for 5 min, and centrifuged at 9000 × *g* for 10 min. Then, 2 ml of supernatant was mixed with a solution containing 2 ml of 2.5% ninhydrin and 2 ml of HAc. The mixture was boiled for 30 min then chilled on ice. After adding 4 ml of toluene, the mixture was centrifuged. The absorbance of the supernatant was measured at 520 nm.

Total flavonoid contents were measured according to Saeed et al. (2012).

### TMT-Based Proteomic Analysis

Oat leaf and root samples (1 g) were ground into powder with liquid nitrogen and then 3 mL of ice-cold BPP buffer was added (100 mM Tris, pH 8.0, containing 50 mM borax, 100 mM EDTA, 50 mM vitamin C, 2β-mercaptoethanol v/v, 1% Triton X-100 v/v, 1% PVPP w/v, 30% sucrose w/v). At room temperature, the samples were vortexed for 10 min, mixed with 6 mL Tris-saturated phenol, and vortexed for a further 15 min. The samples were centrifuged at 15,000 × *g* for 15 min at 4°C, then the supernatant was collected and mixed with an equal volume of BPP buffer. The samples were vortexed for 15 min and centrifuged under the same conditions and the supernatant collected. Five volumes of ammonium-sulfate-saturated methanol were mixed with the supernatant to precipitate proteins at −22°C for 8 h. After centrifugation under the same conditions, protein pellets were collected. Using pre-chilled methanol followed by ice-cold acetone, the protein pellets were washed twice and centrifuged as described above. The pellets were air-dried and stored in liquid nitrogen.

Protein samples (100 μg) were placed in new tubes. Tris(2-carboxyethyl) phosphine (TCEP) was added to the protein samples with a final concentration of 10 mM and incubated at 37°C for 60 min. Then, the samples were mixed with iodoacetamide to a final concentration of 40 mM and incubated at room temperature for 40 min in darkness. To precipitate protein, six volumes of ice-cold acetone were added and the protein mixture was incubated at −20°C for 4 h and centrifuged at 10,000 × *g* for 20 min. Some 50 mM of 100 μl triethylammonium bicarbonate (TEAB) was used to dissolve the protein pellets. Protein digestion was performed at 37°C overnight with a 1:50 w:w ratio of trypsin to protein.

The Bradford method was used to determine the protein concentration. Ten-plex TMT labeling was carried out according to the manufacturer’s protocol. The labeled peptides were desalted with a Strata X C18 SPE column and lypophilized by vacuum centrifugation.

High-performance liquid chromatography (HPLC; Thermo Scientific, MA, United States) coupled with a reverse phase column (XBridge C18 Column 1.7 μm, 2.1 mm, X150 mm) (Waters Corporation, United States) was used to fractionate the labeled peptides. A gradient from 3.04% buffer B to 80% buffer B in 40 min was used (buffer B: 20 mM ammonium formate in 80% ACN, pH 10.0). The column flow rate was maintained at 200 μl.min^–1^. For leaves, 30 fractions were collected and combined into 15 fractions. For roots, 20 fractions were collected and combined into 10 fractions. The combined peptides were lypophilized in a vacuum concentrator.

The peptides were dissolved in buffer C (0.1% formic acid) for MS analysis. With an Easy-nLC 1200 UPLC system connected to a Q-Exactive Plus hybrid quadrupole orbit trap mass spectrometer (Thermo, United States), LC-MS/MS analysis was carried out in the positive-ion mode. Peptide separation was carried out using a reversed-phase analytical column (C18 column, 75 μm × 25 cm, Thermo, United States) with the following gradient: 0%–5% solvent B (0.1% FA in 98% ACN) for 1 min, 5%–23% solvent B for 62 min, 23%–48% solvent B for 25 min, increased to 100% solvent B for 1 min, then held at 100% for 6 min, all at a flow rate of 300 nL.min^–1^.

The Q Exactive instrument was operated in the data-dependent mode (DDA) to automatically switch between full-scan MS and MS/MS acquisition. The survey of full-scan MS spectra (m/z 350–1300) was acquired in the Orbitrap at 70,000 resolution after accumulation of ions to a 1 × 10^6^ target value based on predictive automatic gain control (AGC) from the previous full scan. Dynamic exclusion was set to 18 s. The 20 most intense multiply charged ions were sequentially isolated and fragmented in the octopole collision cell by higher-energy collisional dissociation (HCD) and 35,000 resolution for the fast scanning method. The maximum injection time was 50 ms.

We used the DDA mode and data-dependent top-20 to choose the most abundant precursor ions per survey scan for HCD fragmentation. The MS1 mass resolution was set as 70,000 with automatic gain control of 3e6. The MS/MS resolution was set to 35,000 with automatic gain control of 1e5. The maximum injection time was 50 ms and the dynamic exclusion time was 18 s. The normalized collision energy was set at 30.

The resulting MS/MS data were searched against the NCBInr protein database using ProteomeDiscoverer Software 2.2. The parameters of the identified protein were as follows: precursor mass tolerance = 20 ppm, fragment mass tolerance = 0.02 Da, trypsin (full) was set as the cleavage enzyme with not more than two missing cleavages. Carbamidomethyl at cysteine, TMT 10plex (K), and TMT 10plex (N-term) were indicated as static modifications, while oxidation on methionine and acetylation at protein N-terminals were indicated as dynamic modifications. For protein quantification, only peptides with peptide FDRs ≤ 1 were used. For selection of differentially expressed proteins, we used thresholds of *P* < 0.05 (*t*-tests) and fold changes of >1.2 or <0.83.

Hierarchical cluster analysis (HCL) on these differentially expressed proteins was carried out with Scipy (Version 1.0.0) Python software to better understand the relationships between the protein expression patterns of different treatments. Principal components analysis (PCA) was performed using the ropls (Version 1.6.2) R software from Bioconductor. To determine the categories of proteins of different abundance, gene ontology (GO) annotation was performed with BLAST2GO (2.5.0) software, while Kyoto Encyclopedia of Genes and Genomes database (KEGG) enrichment analyses were performed in Python. Analysis of the correlation between DEPs and DEGs was conducted in RStudio (1.2.5033) software.

### Western Blotting Analysis

For western blotting, 20 μg of total proteins was electrophoresed on a 4% stacking gel and 8% resolving gel (SDS-PAGE). Proteins were blotted to polyvinylidene difluoride membranes (Millipore, Schwalbach, Germany) by a western transfer apparatus (BioRad, CA, United States). Membranes were blocked with 5% skimmed milk in TBST solution (PH 7.4) for 1 h and labeled overnight at 4°C with primary antibody. After washing in TBST three times, membranes were reacted with HRP- conjugated goat anti-rat immunoglobulin G (IgG; 1:5000; Abcam, Cambridge, United Kingdom). Antibody binding was detected by a Tanon 6600 detection system (Tanon, Shanghai, China), then analyzed by an Image Pro Plus 6.0 system (Media Cybernetics, MD, United States).

### Quantitative Real-Time PCR

Samples of roots or leaves (0.2 g) were ground into powder in a mortar with liquid nitrogen. Total RNA was isolated from oat leaves and roots using TruSeq RNA sample preparation kits (Illumina, San Diego, CA, United States) following the manufacturer’s protocol. DNA contamination was removed by adding DnaseI (TaKaRa, Japan). First-strand cDNA was synthesized using First Strand cDNA Synthesis Kits (Stratagene, La Jolla, CA, United States), according to the manufacturer’s instructions, at 42°C for 30 min with 1 μg total RNA. The primer pairs were designed using Primer Premier 5.0 software (Supplementary Materials and Methods 2). Real-time PCR was performed using an ABI PRISM 7500 Real-Time PCR System (Applied Bio-systems, Foster, United States) with a Fast SYBR Green Master Mix Bulk Pack (Invitrogen, United States). RT-qPCR reaction mixtures containing cDNA, 2 × TB Green Premix Ex Taq II, primer and water were amplified with the following cycling parameters: 95°C for 5 min, 40 cycles of 95°C for 30 s, 61°C for 15 s, and 72°C for 20 s. A melting curve analysis with continuous fluorescence measurement was performed at 95°C for 15 s, 60°C for 60 s, then increasing by 0.5°C per cycle to 95°C. Three biological replicates were used.

Actin (Gene Bank accession: AF111812) was set as a constitutive reference. The relative mRNA expression of the target gene was calculated using the 2^–Δ^ ^Δ^ ^*Ct*^ comparative threshold cycle (CT) method.

### Statistical Analysis

Physiological data are expressed as the mean ± standard error (SE) of three biological replicates. Data were analyzed by ANOVA tests using SAS 9.0 statistical software, with *P* < 0.05 used as the significance threshold.

## Results

### Excess Spermine Increases Dry Weight and Chlorophyll Content

As shown in Figure 1B, chlorophyll contents decreased under alkali stress compared to controls, and decreased sharply at the booting stage. Thus, the booting stage was studied in this research. Compared to the single alkali stress plants, adding spermine improved the chlorophyll content and dry weight significantly and promoted root growth under alkali stress (Figures 1C–G). Thus, spermine was used to study the physiological mechanisms related to improving alkali tolerance.

### Quantitative Proteomics Analysis of Control and Alkali Stress in Leaves

**FIGURE 2:**
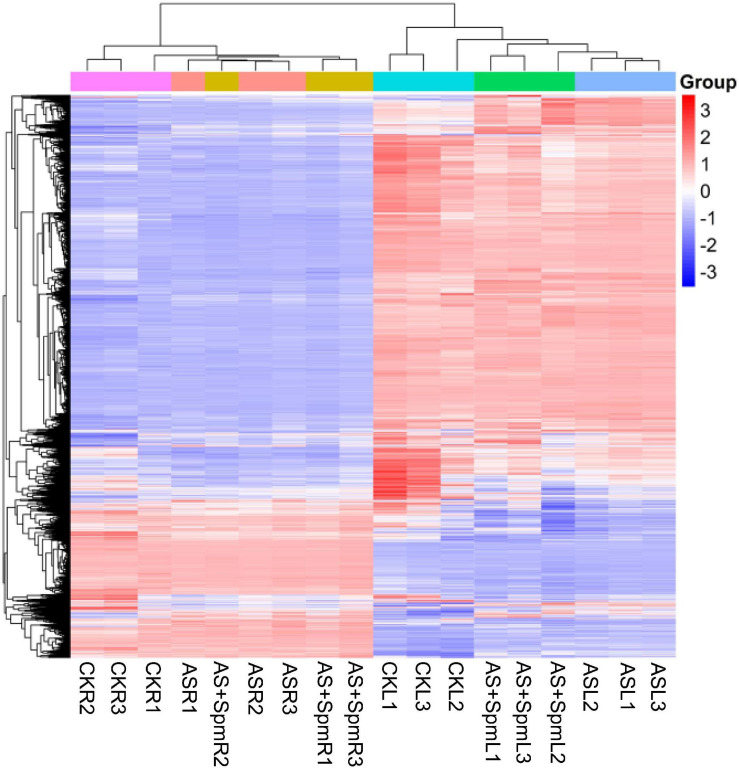
Principal component analysis among all the replicates and treatments. CK, control; AS, alkali stress (35 mmol.L^–1^ Na_2_CO_3_:NaHCO_3_ = 1:1); AS + Spm, alkali stress + spermine (35 mmol.L^–1^ Na_2_CO_3_:NaHCO_3_ + 0.01 mmol.L^–1^ spermine); R, root; L, leaf, 1, replicate 1; 2, replicate 2; 3, replicate 3.

We sought to elucidate the reasons why alkali stress severely inhibits growth and chlorophyll accumulation, and demonstrate the mechanisms of alkali stress tolerance in oats. TMT-based quantitative proteomics was applied to analyze the protein extracted from oat leaves and roots under the control (CK), alkali (AS), and alkali + spermine (AS + Spm) treatments. Three biological replicates were used and the reproducibility of the proteomic analysis is shown in Figure 2. First, we analyzed the differentially expressed proteins (DEPs) in the control and alkali treatments for the leaves and roots.

**FIGURE 3:**
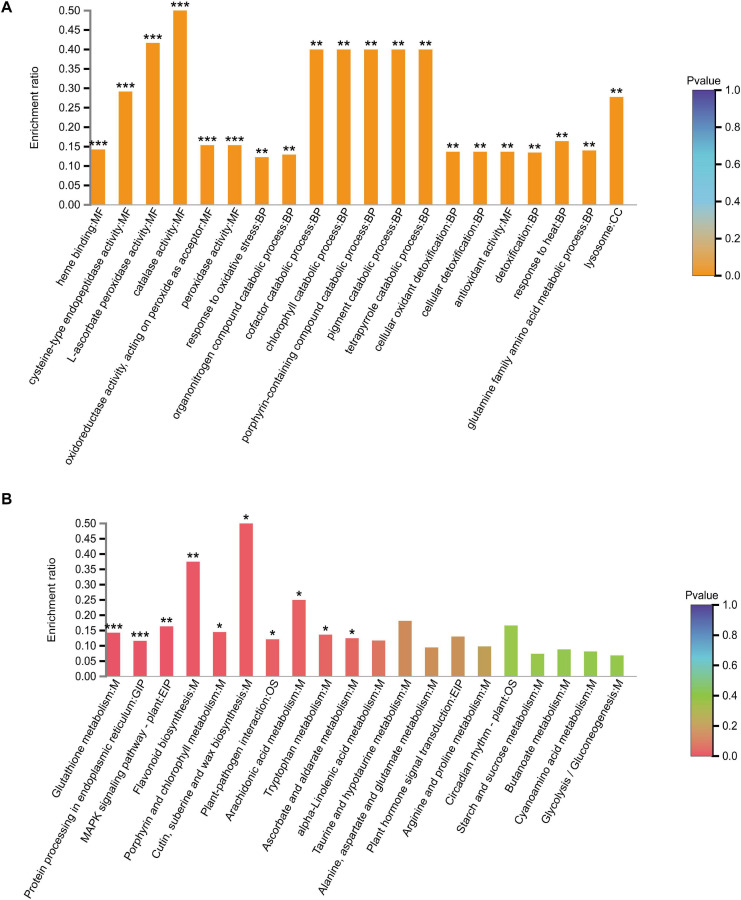
**(A)** GO and **(B)** KEGG analysis of differentially expressed proteins in leaves at CK vs. AS. CK, control; AS, alkali stress (35 mmol.L^–1^ Na_2_CO_3_:NaHCO_3_ = 1:1). The color gradient indicated significance, *** indicated *P* < 0.001, ** indicated *P* < 0.01, * indicated *P* < 0.05.

We identified 408 DEPs in leaves under alkali stress compared to controls. GO enrichment analysis (Figure 3) showed that these 408 proteins are related to the chlorophyll catabolic process, cellular oxidant detoxification, antioxidant activity, L-ascorbate peroxidase activity, and catalase activity, indicating that antioxidant enzymes play important roles in responding to alkali stress. We further verified that the activities of some antioxidant enzymes (SOD, POD, CAT) in leaves increased significantly in response to alkali stress (Supplementary Figure 1). KEGG pathway enrichment analysis demonstrated that most of these proteins are related to glutathione metabolism, porphyrin and chlorophyll metabolism, and flavonoid biosynthesis (Figure 3). We further verified that alkalinity increased the flavonoid contents of leaves (Supplementary Figure 2). These 408 proteins included 303 up-regulated and 105 down-regulated DEPs (Supplementary Table 2).

Among the 303 up-regulated proteins in leaves, 19 were proposed to act as antioxidants, thereby protecting plants from oxidative stress damage (Supplementary Table 3). Thirteen proteins were associated with chloroplast structures and CO_2_ fixation (Supplementary Table 3).

We found some interesting proteins related to improving alkali tolerance, which can be divided into three groups. (1) Proteins involved in the control of carbon balance. Sucrose-phosphate synthase and sucrose synthase type 3 mainly function in sucrose synthesis. Neutral/alkaline invertase 1 catalyzes the irreversible hydrolysis of sucrose into glucose and fructose, and invertase inhibitors interact with neutral/alkaline invertases (Inv) to inhibit its activity. Up-regulation of these proteins indicates that alkali stress affects the carbohydrate metabolism of oat leaves. (2) Four proteins were related to plant hormone metabolism. 1-aminocyclopropane-1-carboxylate oxidase-1-like protein is the ethylene precursor, while histone deacetylase is probably involved in jasmonic acid and ethylene signaling. Up-regulation of these four proteins indicates that plant hormones in oat leaves respond to alkali stress. (3) Two proteins are essential enzymes for proline synthesis. (4) Five proteins are considered to be involved in plant defense against environmental stress, such as chitinase, zinc finger protein. Some of these are related to pathogen defense, such as thaumatin-like protein and PR-1a pathogenesis, indicating that these proteins may play important roles in responding to alkali stress (Supplementary Table 3).

Pheophorbide a oxygenase is an essentia1 enzyme for chlorophyll breakdown and its up-regulation may be one of the reasons why alkali stress decreased chlorophyll accumulation and led to the appearance of yellow leaves (Supplementary Table 3).

Among the 105 down-regulated DEPs in leaves, eight proteins related to chloroplasts were down-regulated (Supplementary Table 3). Coproporphyrinogen III oxidase (CPO) and protoporphyrinogen IX participate in chlorophyll synthesis. Cytochrome f, protein chlororespiratory reduction 6, pyridoxamine 5′-phosphate oxidase, and chlorophyll a-b binding protein 1B-20 are important components of chloroplasts. Down-regulation of these five proteins could disturb chloroplast function and inhibit photosynthesis (Supplementary Table 3).

**FIGURE 4:**
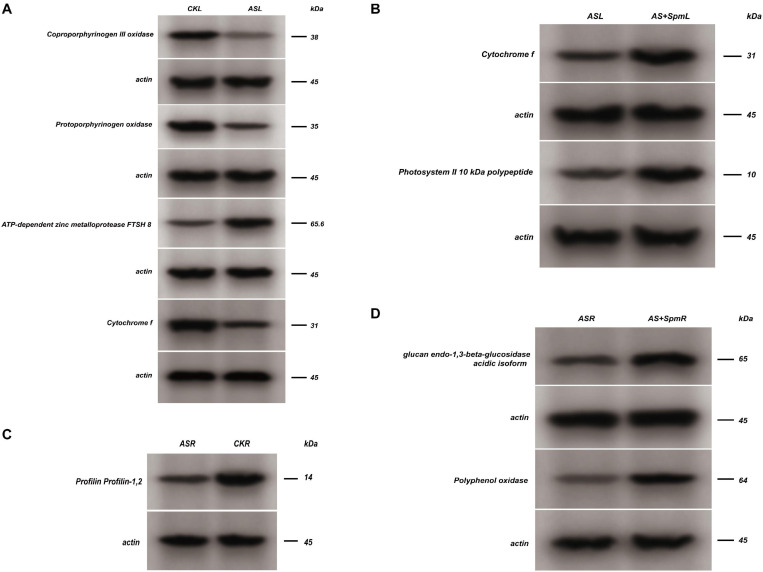
Western blot analysis. CK, control; AS, alkali stress (35 mmol.L^–1^ Na_2_CO_3_:NaHCO_3_ = 1:1); AS + Spm, alkali stress + spermine (35 mmol.L^–1^ Na_2_CO_3_:NaHCO_3_ + 0.01 mmol.L^–1^ spermine); R, roots; L, leaves. The coproporphyrinogen III oxidase, protoporphyrinogen oxidase, ATP-dependent zinc metalloprotease FTSH 8 and cytochrome f expressions of leaves at CK vs. AS were detected by western blot assay, and representative bands were shown in panel **(A)**, the cytochrome f and photosystem II 10 kDa polypeptide expressions of leaves at AS vs. AS + Spm were detected by western blot assay, and representative bands were shown in panel **(B)**, the profilin expressions of roots at AS vs. CK was detected by western blot assay, and representative bands was shown in panel **(C)**, the glucan endo-1,3-beta-glucosidase, polyphenol oxidase expressions of roots at AS vs. AS + Spm were detected by western blot assay, and representative bands were shown in panel **(D)**.

We further verified the up-regulation of ATP-dependent zinc metalloprotease FTSH 8 and down-regulation of coproporphyrinogen III oxidase, protoporphyrinogen oxidase, and cytochrome f in leaves under alkali stress by western-blot analysis (Figure 4 and Supplementary Figure 3).

### Quantitative Proteomics Analysis of Control and Alkali-Stressed Roots

Compared to controls, 1291 differently expressed proteins (DEPs) were found in roots under alkali stress (Supplementary Table 4). GO enrichment analysis (Supplementary Figure 4) showed that these 1291 proteins are highly related to carbohydrate transmembrane transporter activity and ribosomes, etc. KEGG pathway enrichment analysis demonstrated that most of these proteins are widely related to ribosomes, oxidative phosphorylation, the citrate cycle (TCA cycle), starch and sucrose metabolism, fructose and mannose metabolism, etc. (Supplementary Figure 4). These 1291 proteins included 889 up-regulated and 402 down-regulated DEPs (Supplementary Table 4).

Among the 889 up-regulated DEPs, we found eight proteins involved in glycolysis, the tricarboxylic acid cycle, and the pentose phosphate pathway. This indicates that energy and carbohydrate metabolism in roots changed in response to alkali stress (Table 1). Seven proteins are related to sucrose synthesis decomposition and transportation, indicating that sucrose metabolism is involved in the processes responding to alkali stress (Table 1). We found some interesting proteins related to the improvement of alkali tolerance, which can be divided into two groups: (1) Ten proteins are involved in plant hormone synthesis; for example, 1-aminocyclopropane-1-carboxylate oxidase homolog 1-like is the precursor of ethylene, and nitrilase-like protein 2 and Putative flavin-containing monooxygenase 1 contributes to auxin synthesis. Putative aldehyde oxidase 2 and molybdenum cofactor sulfurase contribute to abscisic acid (ABA) biosynthesis, indicating that oats modulated the plant hormone mechanism in response to alkali stress. (2) Seven proteins, such as chitinase and heat shock protein, are involved in plant defense and have multiple functional adaptations that assist in resisting salt and drought stresses. In this study, we found that these proteins were induced in response to alkali stress, indicating that they might help enhance alkali tolerance (Table 1).

**TABLE 1:** Some differentially expressed proteins related to alkali tolerance in roots at AS vs. CK.

**Protein ID**	**Description**	**Up/down**	**Unique peptides number**	**Protein coverage (%)**	**Peptide sequences**
**TCA and pentose phosphate pathway (PPP)**					
TRINITY_DN51780_c0_g1_i1 _m.4463152	Phosphoenolpyruvate carboxykinase	Up	1	6	[K].TTLSADESR.[Y]
TRINITY_DN388294_c0_g1_i2 _m.1185887	Pyruvate dehydrogenase kinase	Up	1	4	[R].AVAEEVAR.[W]
TRINITY_DN312036_c0_g2_i1 _m.180279	2,3-Bisphosphoglycerate-independent phosphoglycerate mutase	Up	1	10	[R].TFACSETVK.[F]
TRINITY_DN393827_c2_g1_i5 _m.3300827	Fructose-1,6-bisphosphatase	Up	6	21	[K].KLDVLSNEVFVK.[A]
TRINITY_DN373968_c0_g3_i1 _m.2360392	Glucokinase	Up	1	5	[K].DVTAITEGK.[F]
TRINITY_DN282835_c0_g1 _i2_m.4051799	Succinate dehydrogenase flavoprotein subunit	Up	1	10	[K].ATVLATGGAGR.[I]
TRINITY_DN319879_c0_g1 _i1_m.3058361	6-Phosphogluconate dehydrogenase, decarboxylating 2	Up	1	11	[R].AAAEGGLPVLGHR.[D]
TRINITY_DN393860_c3_g2 _i1_m.3304521	Transaldolase	Up	1	5	[K].FAADAVTLK.[DS]
**Carbohydrate metabolism**					
TRINITY_DN378496_c1_g1 _i5_m.2242774	Inorganic pyrophosphatase	Up	1	3	[K].IPDGKPENQFAFTGECK.[N]
TRINITY_DN389710_c1_g1 _i1_m.993434	Sugar transport protein 13-like	Up	1	7	[R].GTDNVEPEFNEIVEASR.[I]
TRINITY_DN395511_c1_g2 _i2_m.3041349	Neutral/alkaline invertase 1	Up	1	2	[R].VDVQTGIK.[L]
TRINITY_DN390035_c0_g2 _i9_m.1197912	Probable sucrose-phosphate synthase 4	Up	14	15	[K].LIDKYDLYGQVAYPK.[H]
TRINITY_DN367766_c0_g1 _i8_m.1693602	Sucrose synthase type 3	Up	3	17	[R].VVHGIDVFDPK.[F]
TRINITY_DN573539_c0_g1 _i1_m.3616686	Sucrose synthase 5	Up	2	17	[-].STFQEIAGSK.[E]
TRINITY_DN388501_c0_g1 _i3_m.2462822	Sucrose synthase	Up	1	17	[R].NELIALLSK.[Y]
**Plant hormone**					
TRINITY_DN399120_c1_g1 _i4_m.913870	Tryptophan aminotransferase 1	Up	1	5	[R].GGEQFGGDAR.[C]
TRINITY_DN399649_c1_g1 _i9_m.3201863	Putative flavin-containing monooxygenase 1	Up	1	3	[R].VDEGSIVPK.[N]
TRINITY_DN366503_c0_g1 _i1_m.2637387	1-Aminocyclopropane-1-carboxylate oxidase homolog 1-like	Up	1	14	[R].GVAAEYTQQVR.[R]
TRINITY_DN399772_c4_g2 _i2_m.3141544	Putative aldehyde oxidase 2	Up	2	24	[R].NTATVGGNLIMAQR.[H]
TRINITY_DN321721_c0_g1 _i2_m.3148171	Methionine adenosyltransferase	Up	1	9	[K].QSPDINQGVDR.[K]
TRINITY_DN378428_c0_g1 _i3_m.2243308	Allene oxide synthase 4	Up	4	11	[K].DLPLEPIVTFTSLTK.[A]
TRINITY_DN397654_c2_g1 _i1_m.3162616	Cobalamin-independent methionine synthase	Up	2	63	[R].KYDEVKPALTNMVDAAK.[Q]
TRINITY_DN378840_c1_g1 _i2_m.3294150	Nitrilase-like protein 2	Up	2	6	[K].LAEPLDGPIMQR.[Y]
TRINITY_DN389157_c0_g2 _i1_m.1230902	Molybdenum cofactor sulfurase	Up	2	37	[K].KIEAVGSQSGQTK.[K]
TRINITY_DN388330_c0_g1 _i2_m.1977646	Acyl-coenzyme A oxidase 4	Up	1	23	[R].ELLGGNGILADFLVAK.[A]
**Water metabolism**					
TRINITY_DN397133_c1_g1 _i2_m.3278941	Aquaporin PIP2-5	Up	1	23	[R].YGGGANELSSR.[Y]
TRINITY_DN359969_c2_g3 _i4_m.2682956	Tonoplast intrinsic protein 1-1	Up	1	7	[R].IAIGAPGELSHPDTFR.[A]
TRINITY_DN385803_c0_g1 _i9_m.1142374	Tonoplast intrinsic protein-	Up	1	6	[K].LAFGSLGDSFSATSIK.[A]
TRINITY_DN380423_c1_g2 _i3_m.1296074	PIP aquaporin	Up	2	24	[R].YGGGANELSSGYTK.[G]
TRINITY_DN380423_c1_g1 _i1_m.1296055	Putative PIP-type aquaporin	Up	1	37	[K].GFQQTLYMSTGGGA NAVASGYTK.[G]
TRINITY_DN385803_c0_g1 _i15_m.1142393	Delta tonoplast intrinsic protein TIP2;2	Up	2	11	[R].YLYMCDDHTAVSSDY.[-]
TRINITY_DN385803_c0_g1 _i16_m.1142396	Aquaporin TIP2-3	Up	1	6	[K].LAIGSLGDSFSATSIR.[S]
TRINITY_DN375766_c0_g1 _i2_m.938610	Aquaporin PIP1-1	Up	2	19	[K].GFQQGLYMGNGGGANVVASGYTK.[G]
TRINITY_DN365230_c2_g1 _i4_m.2408520	Aquaporin PIP1-5	Up	1	31	[K].GFQTTLYMGNGGGANSVAPGYTK.[G]
**Resisting stress**					
TRINITY_DN395628_c2_g3 _i4_m.2553628	Heat shock protein Hsp88	Up	1	1	[K].TQEISNLK.[N]
TRINITY_DN390153_c0_g2 _i12_m.2090886	LEA-3 protein	Up	1	14	[K].ASEAAQYTQER.[S]
TRINITY_DN389810_c1_g1 _i13_m.2490263	Chitinase 1	Up	2	11	[K].ASFPNIATSIAPFER.[DG]
TRINITY_DN364677_c2_g3 _i7_m.2315861	Pathogenesis related protein 4	Up	1	27	[K].IDTDGQGYQR.[G]
TRINITY_DN368790_c0_g1 _i3_m.1346522	Cold regulated protein	Up	2	22	[R].LYALDDVLSLGNK.[V]
TRINITY_DN399320_c9_g2 _i1_m.1326337	17.9 kDa class I heat shock protein	Up	1	14	[R].TSSSETAAFAGAR.[I]
TRINITY_DN360622_c4_g6 _i1_m.1268910	Putative heat shock protein ssb1 protein	Up	1	37	[R].IINEPTAAAIAYGLGSGK.[ST]
**Root growth**					
TRINITY_DN397718_c3_g3 _i1_m.2923869	UDP-glycosyltransferase UGT93B9	Down	1	15	[R].ALEGEFVDVVAGHLAPDGK.[K]
TRINITY_DN328030_c2_g1 _i1_m.1319606	Tubulin alpha-1 chain	Down	1	29	[K].TVGGGDDAFNTFFSETGAGK.[H]
TRINITY_DN393329_c4_g1 _i2_m.2010303	WALI7	Down	1	50	[K].DLSGSFAFVVFDGK.[S]
TRINITY_DN397816_c1_g3 _i3_m.1949074	Villin-3-like	Down	22	35	[K].VSEGNEPSFFK.[T]
TRINITY_DN363133_c3_g3 _i1_m.2050947	Actin	Down	2	31	[K].LAYVALDYEQELETSR.[S]
TRINITY_DN395341_c8_g1 _i1_m.2575505	Actin-7-like	Down	1	46	[KR].DAYVGDEAQAK.[R]
TRINITY_DN741371_c0_g1 _i1_m.4247998	LIM domain-containing protein WLIM2b	Down	1	6	[K].AAGMFSGTQDK.[C]
TRINITY_DN295087_c0_g1 _i1_m.3899549	Tubulin beta-2 chain	Down	1	29	[-R].AVLMDLEPGTMDSLR.[ST]
TRINITY_DN387941_c2_g1 _i11_m.1829767	Actin-related protein 7	Down	5	20	[K].RFEIGGTDLTNLFAQELK.[K]
TRINITY_DN359620_c5_g1 _i2_m.3176306	Actin-3	Down	1	38	[R].VAPEEHPVLLTEAPMNPK.[A]
TRINITY_DN385905_c2_g1 _i1_m.3097369	Translationally controlled tumor protein	Down	3	87	[K].LTGDELLSDSFPYR.[E]
TRINITY_DN394237_c1_g1 _i8_m.2225294	Exocyst complex component 5	Down	10	13	[K].QQSDSTGSIGR.[A]
TRINITY_DN386725_c2_g1 _i1_m.2810910	MAP KINASE	Down	2	22	[R].HLDHENIVGLR.[D]
TRINITY_DN391562_c2_g3 _i3_m.2835610	Aspartate aminotransferase A	Down	1	37	[R].LAGATPVILPTSISDNFLLKPDSLASVITEK.[S]
TRINITY_DN389070_c0_g2 _i2_m.2984398	Adenosine kinase 2	Down	1	19	[K].RPENWTLVEK.[A]
TRINITY_DN389938_c1_g1 _i2_m.1920740	Alpha-L-arabinofuranosidase 1	Down	1	6	[K].DVVDGIEFAR.[G]
TRINITY_DN391424_c1_g1 _i5_m.3345662	Profilin	Down	2	70	[K].DFEEPGTLAPTGLFLGGTK.[Y]
TRINITY_DN342829_c2_g2 _i4_m.2093562	Beta tubulin 6	Down	1	76	[R].YVGTADLQLER.[V]
**Carbohydrate metabolism**					
TRINITY_DN399270_c7_g2 _i12_m.2322625	Starch excess 4	Down	1	11	[K].YVVDGNWLCNDHEMK.[T]
TRINITY_DN395260_c3_g4 _i2_m.1375019	Sucrose synthase 7	Down	3	27	[R].MNPGIWEYVK.[V]

*CK, control; AS, 35 mmol.L^–1^ Na_2_CO_3_:NaHCO_3_ = 1:1.*

Among the 402 down-regulated DEPs in alkali-stressed roots compared to controls, 18 proteins, such as profilin, tubulin alpha-1, and actin, play important roles in root growth and morphology and are particularly important in root elongation, lateral root formation, and root hair formation. This phenomenon may be one of the possible causes of root growth inhibition in response to alkali stress. Two proteins are involved in carbohydrate and energy metabolism, such as starch excess 4 and sucrose synthase 7. This indicates that carbohydrates may be involved in the alkali stress response, which warrants further study (Table 1).

We further verified the down-regulation of profilin under alkali stress in roots by western-blot analysis (Figure 4 and Supplementary Figure 3).

### Quantitative Analysis of Proteomics in Leaves in Alkali Stress and Alkali Stress + Spermine Treatments

Of the DEPs identified in oat leaves, 14 showed increases and 26 showed decreases in abundance after spermine treatment compared to alkali stress alone (Supplementary Table 5). GO enrichment analysis (Supplementary Figure 5) showed that these 40 proteins are related to the thiazole biosynthetic process and arginine catabolic process etc. KEGG pathway enrichment analysis demonstrated that these proteins are related to arginine and proline metabolism and thiamine metabolism etc. (Supplementary Figure 5).

Among the 14 up-regulated proteins, up-regulation of putative ornithine aminotransferase contributes to increased proline contents (Table 2). We further verified that adding spermine could increase the proline contents (Supplementary Figure 6). Cytochrome f and photosystem II 10 kDa polypeptide are important components of chloroplasts. Up-regulation of these two proteins can help stabilize the chloroplast structure. Putative invertase inhibitor controls the activity of invertase and plays a significant role in sucrose metabolic processes. Up-regulation of this protein indicates that spermine may have a role in regulating carbohydrate metabolism. Up-regulation of glutathione *S*-transferase lambda 1 can contribute to reducing (ROS) accumulation (Table 2).

**TABLE 2:** Some differentially expressed proteins related to alkali tolerance treated by exogenous spermine compared to alkali stress.

**Protein ID**	**Description**	**Up/down**	**Unique peptides number**	**Protein coverage (%)**	**Peptide sequences**	**Root/leaf**
TRINITY_DN395470_c1_g1 _i8_m.2730616	Arginine decarboxylase	Up	1	23	[R].YDVQHDISSVIEEWAR.[E]	Leaf
TRINITY_DN387562_c5_g1 _i10_m.2262265	Xylanase inhibitor protein 1-like	Up	1	6	[R].NKDEGSLAEACDTGR.[Y]	Leaf
TRINITY_DN397833_c2_g1 _i4_m.1946133	ATP-dependent 6-phosphofructokinase 6-like	Up	1	15	[K].SFGFDSAVEEAQR.[A]	Leaf
TRINITY_DN393592_c1_g1 _i4_m.2756609	Glutamyl-tRNA reductase	Up	1	27	[R].VPGGSSGGSASAVSAR.[Q]	Leaf
TRINITY_DN398938_c5_g4 _i3_m.1279814	3′-*N*-debenzoyl-2′-deoxytaxol *N*-benzoyltransferase-like	Up	1	5	[K].VASSTITDVVK.[I]	Leaf
TRINITY_DN387831_c0_g1 _i9_m.1640573	Serine/threonine-protein kinase STN8	Up	1	14	[K].AAGYDLNR.[W]	Leaf
TRINITY_DN386963_c0_g1 _i1_m.2664437	Nuclear-interacting partner of ALK	Up	1	3	[R].NMEEGGSTADKPINR.[L]	Leaf
TRINITY_DN367024_c0_g1 _i6_m.1971459	Photosystem II 10 kDa polypeptide	Up	5	41	[K].KIQTAQPYGPGGGADFK.[D]	Leaf
TRINITY_DN855066_c0_g1 _i1_m.3466289	Cold responsive protein	Up	2	14	[K].AADATEDAIEGAK.[G]	Leaf
TRINITY_DN399631_c1_g3 _i1_m.3206417	Light-induced protein 1-like	Up	2	12	[R].VTEEVEREYLSYDDAK.[T]	Leaf
TRINITY_DN263154_c0_g1 _i2_m.4123170	Cytochrome f -	Up	3	36	[K].KGGLNVGAVLILPEGFELAPPDR.[I]	Leaf
TRINITY_DN368820_c0_g1 _i4_m.3056111	Putative invertase inhibitor	Up	1	4	[R].CEALYDR.[M]	Leaf
TRINITY_DN771345_c0_g1 _i1_m.4363218	Putative ornithine aminotransferase	Up	2	41	[R].LAPPLSISSEELAEASK.[A]	Leaf
TRINITY_DN371528_c1_g1 _i12_m.1648156	Glutathione *S*-transferase lambda 1	Up	1	4	[K].NQPLILLDAAK.[R]	Leaf
TRINITY_DN355168_c0_g1 _i1_m.2929380	Abscisic stress-ripening protein 2	Up	1	31	[K].IEEEVAAAAAVGAGGFVFHEHHEK.[K]	Leaf
TRINITY_DN379801_c2_g1 _i3_m.2459734	Vacuolar-processing enzyme beta-isozyme 1-like	Down	1	2	[K].DYTGDEVTTK.[N]	Leaf
TRINITY_DN388501_c0_g1 _i3_m.2462822	Sucrose synthase	Up	1	17	[R].NELIALLSK.[Y]	Root
TRINITY_DN322423_c1_g2 _i1_m.1661457	Glycoside hydrolase family 7	Up	2	12	[K].LIGNPQSEIANNPGSSVTDSFCK.[A]	Root
TRINITY_DN367766_c0_g1 _i8_m.1693602	Sucrose synthase type 3	Up	3	17	[K].NITGLVEAYSK.[N]	Root
TRINITY_DN397008_c2_ g2_i8_m.2504541	G-type lectin *S*-receptor-like serine/threonine-protein kinase At2g19130	Up	1	5	[R].LKGDANPDELMK.[A]	Root
TRINITY_DN382045_c0_g1 _i6_m.3191285	Vesicle-associated membrane protein 721-like	Up	1	14	[R].TEQLNEQAR.[D]	Root
TRINITY_DN382438_c1_g1 _i6_m.2032609	Cellulose synthase-like protein E6	Up	1	4	[R].FTEEYKEDWDGGIK.[E]	Root
TRINITY_DN374351_c5_g1 _i7_m.1768499	1,3-Beta glucanase	Up	2	38	[R].IYFADGQALSALR.[N]	Root
TRINITY_DN355421_c3_g1 _i3_m.2825170	Glucan endo-1,3-beta-glucosidase, acidic isoform	Up	1	11	[K].YIAVGNEVSGGDTQLILPAMK.[N]	Root
TRINITY_DN388147_c2_g1 _i10_m.1808979	Monodehydroascorbate reductase	Up	4	39	[K-].IAAFYESYYTNK.[G]	Root
TRINITY_DN366217_c2_g2 _i9_m.2500472	Glutathione *S*-transferase GSTF1	Up	2	13	[R].NPFGQIPAFQDGDLLLFESR.[A]	Root
TRINITY_DN193217_c0_g1 _i1_m.531296	Polyphenol oxidase	Up	2	27	[R].VRPAAHLVDAQYLDK.[Y]	Root
TRINITY_DN389810_c1_g1 _i5_m.2490232	Chitinase	Up	2	45	[K].VSSYGFEYETR.[A]	Root

Among the 26 down-regulated proteins, down-regulation of vacuolar-processing enzyme beta-isozyme 1-like, which leads to vacuolar collapse and plasma membrane loss, could decrease the adverse effects of alkali stress on oats (Table 2).

We used western-blot analysis to further verify the up-regulation of cytochrome f and photosystem II 10 kDa polypeptide in leaves treated with spermine compared with untreated alkali-stressed leaves (Figure 4 and Supplementary Figure 3).

### Quantitative Proteomics Analysis of Alkali Stress and Alkali Stress + Spermine in Roots

A total of 192 differentially abundant proteins in roots were found to be affected by spermine treatment compared to alkali stress alone (Supplementary Table 6). GO enrichment analysis (Supplementary Figure 7) showed that these 192 proteins are highly related to the cellular carbohydrate metabolic process, glucan metabolic process, L-alanine catabolic process, etc. KEGG pathway enrichment analysis demonstrates that these proteins are related to starch and sucrose metabolism, glycolysis/gluconeogenesis, etc. These 192 proteins included 78 up-regulated and 114 down-regulated DEPs (Supplementary Figure 7).

Among these 78 up-regulated proteins, sucrose synthase type 3 functions in sucrose synthesis and glycoside hydrolase family 7 participates in glucose mechanism. This indicates that spermine may facilitate the accumulation of sucrose and affect carbohydrate metabolism in roots (Table 2). Up-regulation of G-type lectin *S*-receptor-like serine and vesicle-associated membrane protein 721-like could facilitate root growth and development. Cellulose synthase-like protein E6, glucan endo-1,3-beta-glucosidase, acidic isoform, and Beta-glucosidase 6 are involved in cell wall synthesis, which is linked to cell growth (Table 2). Glutathione *S*-transferase GSTF1, alternative oxidase, and monodehydroascorbate reductase are involved in the antioxidant system, protecting against oxidative damage. We further verified that adding spermine could improve the activities of some antioxidant enzymes (SOD and CAT) and decrease the MDA content in roots. This phenomenon indicates that spermine may help eliminate ROS and decrease membrane lipid peroxidation in roots (Supplementary Figure 1).

Polyphenol oxidase functions in the elimination of toxic substances. Chitinase is involved in responses to abiotic stress, so its up-regulation may help improve alkali tolerance (Table 2).

We used western-blot analysis to further verify the up-regulation of glucan endo-1,3-beta-glucosidase and polyphenol oxidase in roots treated with spermine compared with untreated alkali-stressed roots (Figure 4 and Supplementary Figure 3).

### Q-PCR Verification

The qPCR technique was used to verify the proteomic data. The qPCR results of some DEGs were consistent with the results of the proteomic analysis. However, we also found that some down-regulated (or up-regulated) DEPs, such as protoporphyrinogen oxidase in the leaves of groups AS vs. CK, exhibited relatively low differential expressions at the transcript level, indicating that the expressions of these DEPs were also modulated at post-transcriptional levels (Supplementary Figure 8).

### Contents of Sucrose, Glucose, Fructose, and Starch

In the oat leaves, alkali stress significantly increased the contents of sucrose, glucose, fructose, starch, and soluble sugar. The concentrations in the spermine-treated oat leaves were remarkably lower than those without added spermine under alkali stress. Alkali stress decreased the ratio of hexose to sucrose, which was increased by spermine addition in alkali-treated oats (Table 3).

**TABLE 3:** Effect of spermine application on the carbohydrate contents in leaves and roots of oats under alkali stress.

**Treatment**	**Sucrose (mg.g**^–^**^1^)**	**Glucose (μ molg**^–^**^1^)**	**Fructose (mg.g**^–^**^1^)**	**Starch (mg.g**^–^**^1^)**	**Soluble sugar (mg.g**^–^**^1^)**	**Hexose/sucrose**	**Soluble sugar in roots/soluble sugar in leaves**
				**Leaves**			

**CK**	27.12 ± 1.21c	18.32 ± 1.16c	19.03 ± 0.89c	43.18 ± 1.41c	91.82 ± 1.32c	1.38 ± 0.003a	–
**AS**	40.13 ± 2.51a	25.64 ± 1.12a	26.13 ± 0.73a	58.23 ± 1.83a	133.11 ± 2.61a	1.29 ± 0.007c	–
**AS + Spm**	33.68 ± 1.03b	22.18 ± 0.83b	23.31 ± 0.83b	49.18 ± 1.26b	110.37 ± 3.16b	1.35 ± 0.011b	–

				**Roots**			

**CK**	9.35 ± 1.12c	8.49 ± 1.37b	6.79 ± 0.37b	25.93 ± 2.42a	42.32 ± 0.63c	1.63 ± 0.008a	0.46 ± 0.017a
**AS**	16.22 ± 0.43b	13.57 ± 1.32a	8.98 ± 0.62a	16.35 ± 1.90c	48.28 ± 0.35a	1.38 ± 0.005b	0.36 ± 0.012c
**AS + Spm**	18.43 ± 0.69a	14.93 ± 1.71a	10.23 ± 0.51a	21.22 ± 2.3b	45.49 ± 0.29b	1.37 ± 0.010b	0.41 ± 0.013b

*CK, control; AS, 35 mmol.L^–1^ Na_2_CO_3_:NaHCO_3_ = 1:1; AS + Spm, 35 mmol.L^–1^ Na_2_CO_3_:NaHCO_3_ + 0.01 mmol.L^–1^ spermine.*

In the oat roots, alkali stress significantly increased the contents of sucrose, glucose, fructose, and soluble sugar, and decreased the starch contents. Spermine addition increased the sucrose and starch contents in alkali-stressed roots. Significant decreases in the hexose-to-sucrose ratio were observed in roots under alkali stress (Table 3).

The ratio of total carbohydrate content in roots to that in leaves was 21.74% lower in alkaline-stressed plants than in controls, and spermine addition inhibited stress-induced decreases in this ratio (Table 3).

### Plant Hormones

**FIGURE 5:**
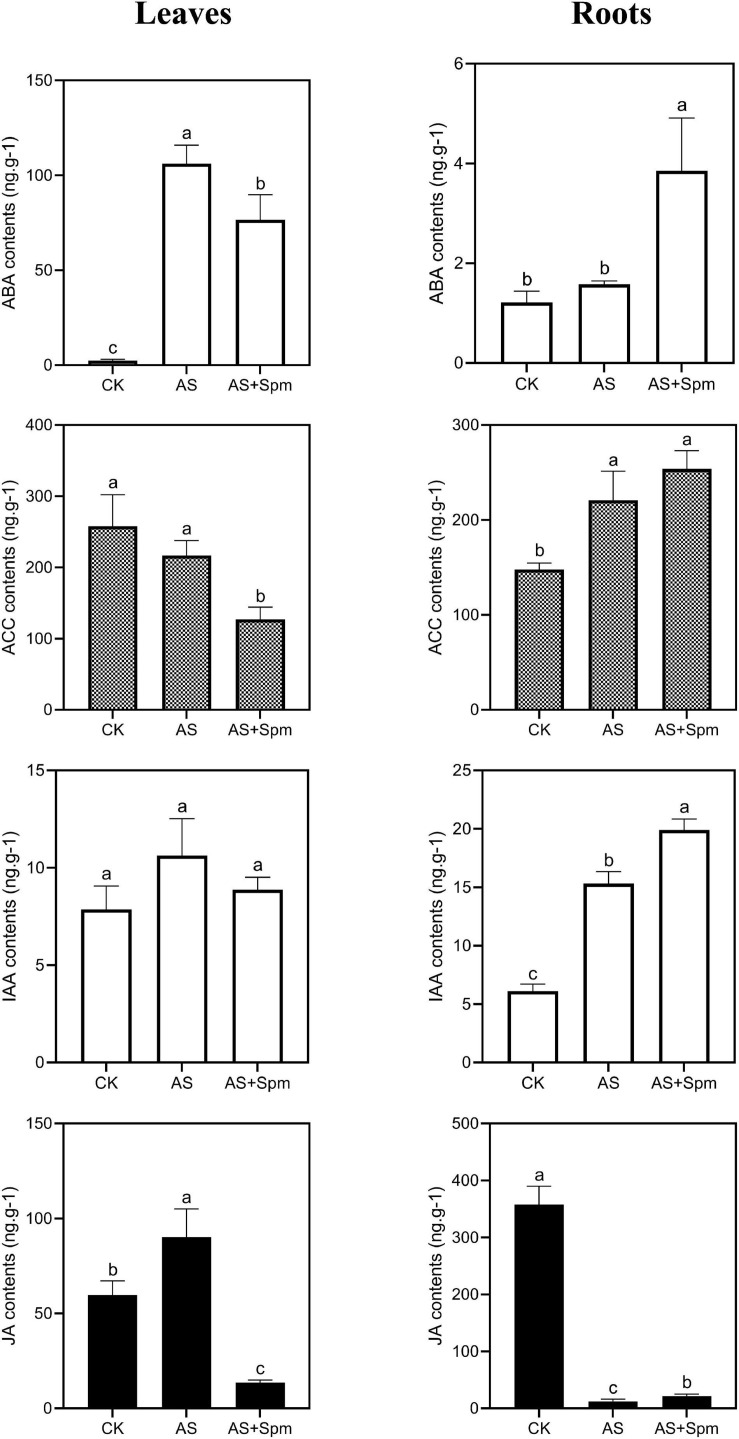
Effects of spermine on ABA, ACC, IAA, JA contents under alkali stress. CK, control; AS, alkali stress (35 mmol.L^–1^ Na_2_CO_3_:NaHCO_3_ = 1:1); AS + Spm, alkali stress + spermine (35 mmol.L^–1^ Na_2_CO_3_:NaHCO_3_ + 0.01 mmol.L^–1^ spermine).

Under alkali stress, the contents of ABA, JA in leaves and the contents of ACC (ethylene precursor), IAA in roots increased significantly, while the JA content in roots decreased. Compared to the single alkali stress group, the exogenous spermine increased the ABA, IAA, and JA contents in roots by 144.39%, 30.07%, and 73.54%, respectively. Adding spermine decreased the ACC contents in leaves compared to that in the untreated alkali-stressed group. This phenomenon is similar to the results of proteomic analysis (Figure 5).

## Discussion

### The Toxic Effects of Alkali Stress on Oats

Alkali stress inhibited crop growth and yield more severely than salt stress. However, the reasons remain unclear. It is has been reported that plant growth is driven directly by photosynthesis (Fatichi et al., 2014) and chlorophyll is the key pigment driving oxygenic photosynthesis (Schliep et al., 2013). Early reports revealed that severe saline-alkali stress decreases chlorophyll contents (Wang et al., 2013). In our previous study, alkali stress was found to cause yellow leaves and had a stronger inhibitory effect on chlorophyll accumulation than salt stress (Bai et al., 2018). However, the reasons for reductions in chlorophyll content due to alkalinity have not been investigated. In this study, we identified coproporphyrinogen III oxidase and protoporphyrinogen oxidase, which participate in the chlorophyll biosynthesis pathways. Coproporphyrinogen III oxidase (CPO) catalyzes the oxidative decarboxylation of coproporphyrinogen III to form protoporphyrinogen IX in heme biosynthesis and is shared in chlorophyll biosynthesis. Protoporphyrinogen oxidase catalyzes the oxidation of protoporphyrinogen IX to protoporphyrin IX. This is the last common step in the production of heme and chlorophyll. Down-regulation of these two proteins may explain the reduction in the accumulation of chlorophyll content due to alkali stress.

We found that alkali stress significantly decreased the root dry weight of oat plants. This is consistent with Wang et al. (2012), who found that alkalinity inhibited rice root growth. Root growth is maintained by coordinating cell proliferation and differentiation. We identified some root-growth-related proteins, such as actin, beta tubulin (Liu et al., 2016), and profilin. Plant microtubules are important components that play crucial roles in cell expansion and cell division. Microtubules are mainly composed of alpha and beta-tubulin (Whittaker and Triplett, 1999). Actins are involved in many important developmental processes, such as cell wall deposition, cell elongation, and root hair morphogenesis (Meagher et al., 1999). Profilin is an actin-binding protein involved in cell elongation and cell shape maintenance, and it has been reported that Arabidopsis seedlings overexpressing profilin have longer roots and root hairs than wild-type seedlings (Ramachandran et al., 2000). In this study, down-regulation of four actin proteins, three microtubule proteins, and profilin may be reasons why alkalinity limited root growth.

### Mechanisms Related to Alkali Tolerance in Oats

Early reports revealed that oats can tolerate alkali soils (pH > 9.0; Zhao et al., 2007). The detailed molecular mechanisms involved in the alkali tolerance of oats have not been explored clearly. In this study, several results were related to the tolerance improvement. (1) Firstly, the TMT-proteomic analysis identified that under alkali stress, Catalase, Monodehydroascorbate reductase, probable L-ascorbate peroxidase 7, 5′-adenylylsulfate reductase 1, cytosolic glutathione reductase, lethal leaf spot1, and quinone-oxidoreductase homologs were up-regulated, which contributed to the scavenging of toxic substances. Catalase, monodehydroascorbate reductase and, probably, L-ascorbate peroxidase 7 function in scavenging hydrogen peroxide. 5′-Adenylylsulfate reductase 1 and cytosolic glutathione reductase play important roles in glutathione synthesis, which scavenges reactive oxygen intermediates to combat oxidative stress (Table 2). Lethal leaf spot1 and putative quinone-oxidoreductase homologs are known to catalyze the metabolic detoxification of phenolics and quinones and protect cells from oxidative stress. Gene ontology (GO) enrichment analysis showed DEPs in leaves related to antioxidant activity. Further investigation using the spectrophotometric method indicated that oat leaves and roots decreased the adverse effects of ROS by increasing SOD, POD, and CAT activities in alkali conditions. (2) Secondly, RuBisCO activase beta form precursor, outer envelope pore protein 37, and NADH dehydrogenase [ubiquinone] 1 alpha subcomplex assembly factor 3 are components of chloroplasts. Fructokinase-1 is crucial for early chloroplast development (He L. et al., 2018). ATP-dependent zinc metalloprotease FTSH 8 plays an important role in the formation of thylakoid membranes during early chloroplast development and seems to be crucial for chloroplast differentiation (Miura et al., 2007; Kato and Sakamoto, 2018). In this study, these five proteins were up-regulated in leaves under alkali stress. These results suggest that oat can improve the expression of some chloroplast-related proteins to maintain the stability of chloroplasts under alkali stress. Ribulose bisphosphate carboxylase activase (RCA) participates in catalyzing the first reaction of photosynthetic CO_2_ assimilation. It has been reported that overexpression of RCA can increase photosynthetic efficiency (Chen et al., 2010). Here, we found that alkalinity increased the expression of RCA in leaves, which could help relieve the limitation on photosynthesis caused by alkali stress. Zhao et al. (2019) found that alkali stress decreased the expression of RCA in oat shoots, which is not consistent with our results, perhaps due to the different stress times used. (3) Thirdly, previous studies indicate there is an accumulation of proline, which may serve as an osmotic agent to increase permeability and maintain the balance of water metabolism. Transgenic plants overproducing proline show increased tolerance to osmotic stress (Verdoy et al., 2006). In this study, TMT-proteomic analysis identified the up-regulation of putative ornithine aminotransferase and putative delta-1-pyrroline-5-carboxylate 1, which function in proline synthesis. Moreover, using the spectrophotometric method, we verified that alkalinity can increase proline contents in leaves and roots to improve alkali tolerance. (4) Fourthly, heat shock proteins, which protect proteins from terminal aggregation, can improve the tolerance of plants to cold, heat, and drought stresses (Guo et al., 2014; Fragkostefanakis et al., 2015). Chitinases are noted to occur in response to cold, salt, and drought stresses (Grover, 2012). In this study, proteomic analysis showed that alkali stress significantly increased the expression levels of heat shock proteins and chitinases, indicating that heat shock proteins and chitinases may contribute to alkali tolerance.

Under alkali stress, TMT-proteomic analysis identified the up-regulation of JA synthesis-related protein histone deacetylase in leaves, and the up-regulation of ethylene precursor ACC and two IAA synthesis-related proteins—nitrilase-like protein 2 and putative flavin-containing monooxygenase 1—in roots. We hypothesized that oat hormones may respond to alkali stress. Further investigation using LC-MS/MS indicated that alkalinity increased the contents of ABA and JA in leaves and increased the ACC and IAA contents in roots. These results are similar to those of Wen et al. (2018), who found that alkali stress increased the IAA contents of roots of Malus rootstocks (Wen et al., 2018). JA can stimulate the activities of antioxidant enzymes and eliminate ROS. It has been reported that JA mitigates the toxic effects of alkalinity and facilitates the photosynthesis and growth of maize under alkali stress (Mir et al., 2018). ABA helps maintain water balance (Pye et al., 2018). Thus, increases in JA and ABA contents in oat leaves were observed to improve alkali tolerance in the present study. IAA plays an important role in the root system’s architecture by affecting cell division, expansion, and differentiation (He F. et al., 2018). Increases in IAA contents in oat roots can facilitate root growth in stressful environments.

### Effects of Exogenous Spermine on Oats Under Alkali Stress

To identify the mechanisms related to the improvement of alkali tolerance, we added spermine to an alkali solution. We found that alkali-stressed plants with Spm had higher chlorophyll contents and dry weights of shoots and roots than non-Spm plants. Similar results have been reported in Spm-treated *Malus hupehensis* Rehd seedlings (Gong et al., 2018). However, the mechanisms by which exogenous spermine increases alkali tolerance have not been investigated thoroughly. Cytochrome f is closely related to electron transport capacity in photosynthesis, and photosystem II 10 kDa polypeptide is associated with the oxygen-evolving complex of photosystem II. Here, we showed that spermine up-regulated the expressions of cytochrome f and photosystem II 10 kDa polypeptide in the leaves of alkali-stressed plants, thereby promoting photosynthesis under alkali stress (Smith et al., 2000). G-type lectin *S*-receptor-like serine protein kinase, a novel putative protein kinase, can facilitate root growth and plays important roles in plant responses to salt and drought stresses (Sun et al., 2013). VAMP721 is involved in plant growth and development (Yi et al., 2013). b-Glucosidase catalyzes the hydrolysis of the b-glucosidic bond between two carbohydrate moieties or between a carbohydrate and an aglucone moiety, and participates in the formation of intermediates in cell wall lignification (Morant et al., 2008). b-Glucosidases contribute to the maintenance of the structural integrity of cell walls, which is critical for cell growth (Rui and Dinneny, 2020). These three proteins contribute to root growth and, in this study, exogenous spermine increased their expressions under alkali stress. Glutathione *S*-transferase has been found to metabolize some toxic compounds (xenobiotics) via GSH conjugation (Edwards et al., 2000). Up-regulation of glutathione *S*-transferase lambda 1 via spermine application might contribute to the elimination of toxic compounds.

In alkali-stressed plants, the up-regulation of sucrose synthase was higher in plants treated with spermine than in non-treated plants. KEGG pathway enrichment analysis showed that some DEPs in roots at AS vs. AS + Spm are highly related to starch and sucrose metabolism. These results suggest that spermine plays an important role in regulating carbohydrate metabolism under alkali stress. We further measured the contents of sucrose, glucose, fructose, starch, and soluble sugar in roots and leaves. It has been reported that alkali stress increases the soluble sugar content of oats (Bai et al., 2013). A higher soluble sugar content can contribute to osmotic adjustment and stabilization of cellular membranes. However, it has also been demonstrated that an increase in soluble sugar may cause lower sugar utilization during leaf growth and alternating source/sink metabolism, which leads to feedback repression of photosynthesis (Khelil et al., 2007). In this study, spermine addition decreased the contents of sucrose, glucose, fructose, and soluble sugar in leaves, suggesting that it contributed to alleviation of the photosynthesis feedback repression caused by alkalinity. Sucrose is an important photosynthesis product and its main hydrolysis products are hexoses, such as glucose and fructose (Khelil et al., 2007). The hexose/sucrose ratio can be used to represent sucrose utilization (Khelil et al., 2007). Improvement in sucrose utilization can provide more energy for plant growth (Dong et al., 2011). In this study, alkali stress decreased the hexose/sucrose ratio, while adding spermine increased this ratio in leaves compared with those of non-treated plants, suggesting that spermine can improve sucrose utilization and facilitate leaf cell growth and development. The ratio of the total carbohydrate content in roots to that in leaves decreased under alkaline conditions while spermine addition increased it, suggesting that exogenous spermine promotes carbohydrate export from leaves to roots (Mittler, 2006). Thus, spermine addition increased the starch contents of roots compared with those of untreated plants. The accumulation of starch in roots may help store energy for root growth, water absorption, and increased alkali tolerance.

The effects of spermine on plant hormones in alkalinity have not been reported. Ethylene is known to be the promoter of plant senescence. High levels of ethylene accumulation inhibit primary root elongation and adversely affect salt tolerance (Zhang M. et al., 2016; Qin et al., 2019). In this study, compared to plants under alkali stress, the ethylene precursor ACC was down-regulated at the protein level in leaves treated with spermine. Further investigation using LC-MS/MS showed that spermine addition decreased the ACC contents of leaves compared to untreated plants, which could help delay plant senescence and maintain oat growth. In addition, exogenous spermine increased the JA and IAA contents of roots compared with plants under alkali stress, which could contribute to root growth.

## Conclusion

To conclude, (1) alkalinity down-regulated the expression levels of coproporphyrinogen III oxidase, protoporphyrinogen oxidase, and pheophorbide a oxygenase, which disturbed chlorophyll synthesis and contributed to the appearance of yellow leaves. (2) Oat plants improved their expression levels of 19 antioxidant proteins in leaves to eliminate ROS, and increased proline contents in leaves to maintain the balance of water metabolism. (3) Compared to single-alkali-stress plants, adding spermine increased chlorophyll contents and plant dry weight. Adding spermine up-regulated chitinase and some proteins involved in chloroplast structure, root growth, and the antioxidant system to improve alkali tolerance. Exogenous spermine increased the hexose/sucrose ratio to improve sucrose utilization efficiency in leaves and promoted carbohydrate export from leaves to roots to increase energy storage in roots. Adding spermine increased root IAA and JA contents, which facilitated root growth, and decreased leaf ACC contents, which delayed senescence.

## Data Availability Statement

The datasets presented in this study can be found in online repositories. The names of the repository/repositories and accession number(s) can be found in the article/Supplementary Material.

## Author Contributions

JB, KJ, and WQ designed the experiments. JB conducted the experiments and wrote the manuscript. YW and QY participated in the experiments. WQ helped to take photos involved in this experiments. KJ helped to revise this manuscript. All authors contributed to the article and approved the submitted version.

## Conflict of Interest

The authors declare that the research was conducted in the absence of any commercial or financial relationships that could be construed as a potential conflict of interest.

## Supplementary Material

Supplementary Figure 1 Effects of spermine on the MDA contents and SOD, POD, CAT activities under alkali stress. SOD, superoxide dismutase; POD, peroxidase; CAT, catalase; MDA, malondialdehyde; CK, control; AS, alkali stress (35 mmol.L^–1^ Na_2_CO_3_:NaHCO_3_ (=1:1),; AS (+Spm:, 35 mmol.L(-1 Na2CO3NaCO3:NaHCO3(1:1) (+0.01 mmol.L(1L-1 spermine.

Supplementary Figure 2 Effects of alkali stress on total flavone contents in leaves.

Supplementary Figure 3 Western blot analysis. **(A)** The expression levels of coproporphyrinogen III oxidase, protoporphyrinogen oxidase, ATP-dependent zinc metalloprotease FTSH 8, cytochrome f of leaves (L) at AS were normalized to CK. **(B)** The expression levels of cytochrome f and photosystem II 10 kDa polypeptide of leaves (L) at AS + Spm were normalized to AS. **(C)** The expression level of profilin of roots (R) at AS was normalized to CK. **(D)** The expression levels of glucan endo-1,3-beta-glucosidase, polyphenol oxidase of roots (R) at AS + Spm were normalized to AS. The results were presented as mean ± SD (*n* = 3). ^∗^*p* < 0.05, ^∗∗^*p* < 0.01. CK, control; AS, alkali stress (35 mmol.L^–1^ Na_2_CO_3_:NaHCO_3_ = 1:1); AS + Spm, alkali stress + spermine (35 mmol.L^–1^ Na_2_CO_3_:NaHCO_3_ + 0.01 mmol.L^–1^ spermine); R, roots; L, leaves.

Supplementary Figure 4 **(A)** GO and **(B)** KEGG analysis of differentially expressed proteins in roots at CK vs. AS. The color gradient indicated significance, ^∗∗∗^ indicated *P* < 0.001, ^∗∗^ indicated *P* < 0.01, ^∗^ indicated *P* < 0.05. CK, control; AS, alkali stress (35 mmol.L^–1^ Na_2_CO_3_:NaHCO_3_ = 1:1).

Supplementary Figure 5 **(A)** GO and **(B)** KEGG analysis of differentially expressed proteins in leaves at AS vs. Spm. The color gradient indicated significance, ^∗∗∗^ indicated *P* < 0.001, ^∗∗^ indicated *P* < 0.01, ^∗^ indicated *P* < 0.05. AS, alkali stress (35 mmol.L^–1^ Na_2_CO_3_:NaHCO_3_ = 1:1); AS + Spm, 35 mmol.L^–1^ Na_2_CO_3_:NaHCO_3_(1:1) + 0.01 mmol.L^–1^ spermine.

Supplementary Figure 6 Effects of spermine on proline contents under alkali stress. CK, control; AS, alkali stress (35 mmol.L^–1^ Na_2_CO_3_:NaHCO_3_ = 1:1); AS + Spm, 35 mmol.L^–1^ Na_2_CO_3_:NaHCO_3_(1:1) + 0.01 mmol.L^–1^ spermine.

Supplementary Figure 7 **(A)** GO and **(B)** KEGG analysis of differentially expressed proteins in roots at AS vs. AS + Spm. The color gradient indicated significance, ^∗∗∗^ indicated *P* < 0.001, ^∗∗^ indicated *P* < 0.01, ^∗^ indicated *P* < 0.05. AS, alkali stress (35 mmol.L^–1^ Na_2_CO_3_:NaHCO_3_ = 1:1); AS + Spm, 35 mmol.L^–1^ Na_2_CO_3_:NaHCO_3_ (1:1) + 0.01 mmol.L^–1^ spermine.

Supplementary Figure 8 The quantitative RT-PCR analysis of some genes. The values are the mean ± SE of three independent biological replicates normalized against the reference gene *Actin.* Varied letters: significant differences at the 0.05 level. *SUC*, sucrose synthase type 3; *RUB*, RuBisCO large subunit-binding protein subunit beta; *COP*, coproporphyrinogen III oxidase; *ADZM*, ATP-dependent zinc metalloprotease FTSH 8; *PROGO*, protoporphyrinogen oxidase; *CYT*, cytochrome f; *PHO10*, photosystem II 10 kDa polypeptide; *PIP1-1*, aquaporin PIP1-1; *POLYO*, polyphenol oxidase; *GLUEI*, glucan endo-1,3-beta-glucosidase, acidic isoform.
