# Supplementary figures and images for: Proteomic Responses to Alkali Stress in Oats and the Alleviatory Effects of Exogenous Spermine Application
Source: Front Plant Sci. 2021 Apr 1;12:627129. doi: 10.3389/fpls.2021.627129 (PMC8049610; doi:10.3389/fpls.2021.627129)

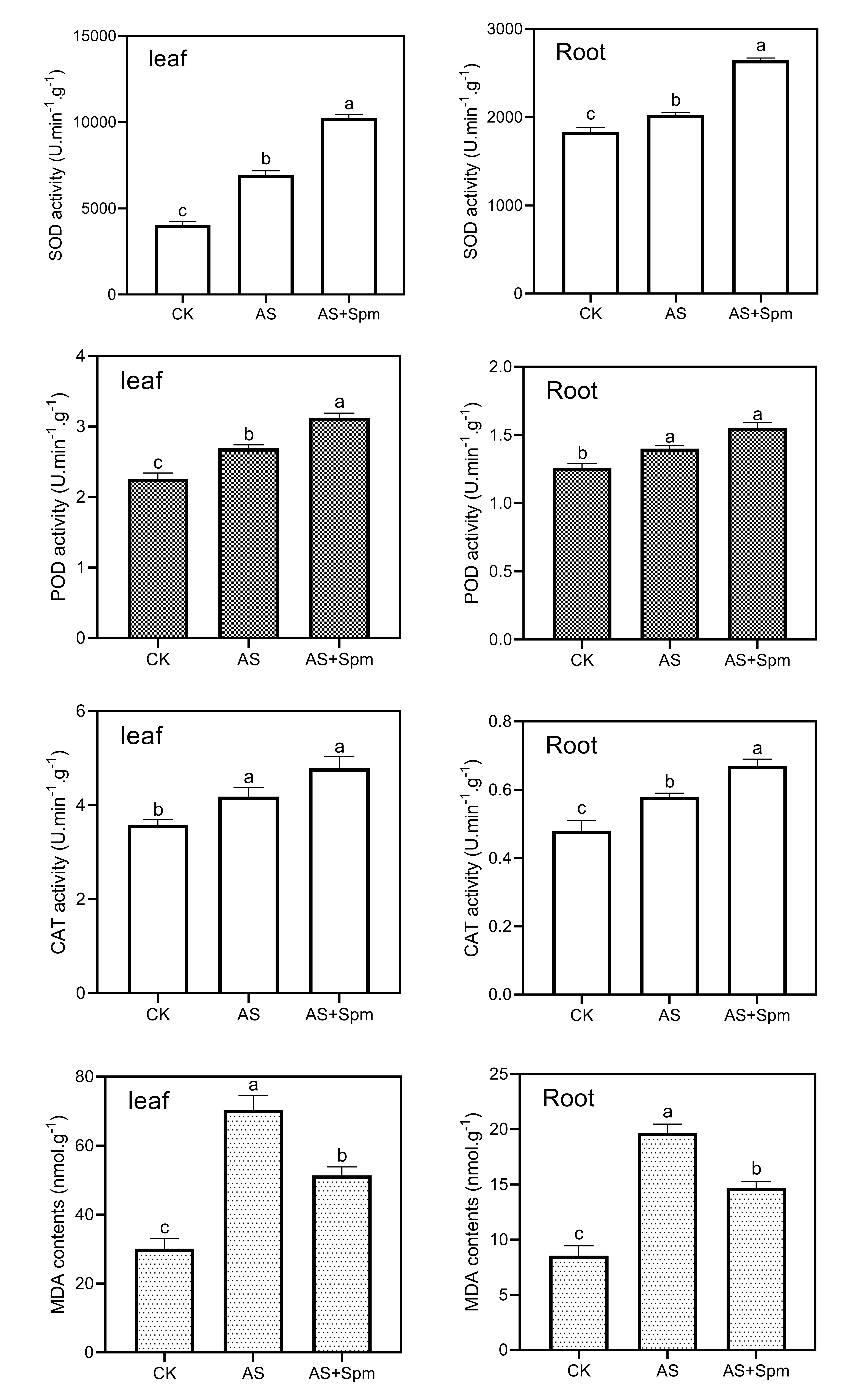

Supplement: Supplementary Figure 1 — Effects of spermine on the MDA contents and SOD, POD, CAT activities under alkali stress. SOD, superoxide dismutase; POD, peroxidase; CAT, catalase; MDA, malondialdehyde; CK, control; AS, alkali stress (35 mmol.L–1 Na2CO3:NaHCO3 (=1:1),; AS (+Spm:, 35 mmol.L(-1 Na2CO3NaCO3:NaHCO3(1:1) (+0.01 mmol.L(1L-1 spermine. [file Image_1.TIF]

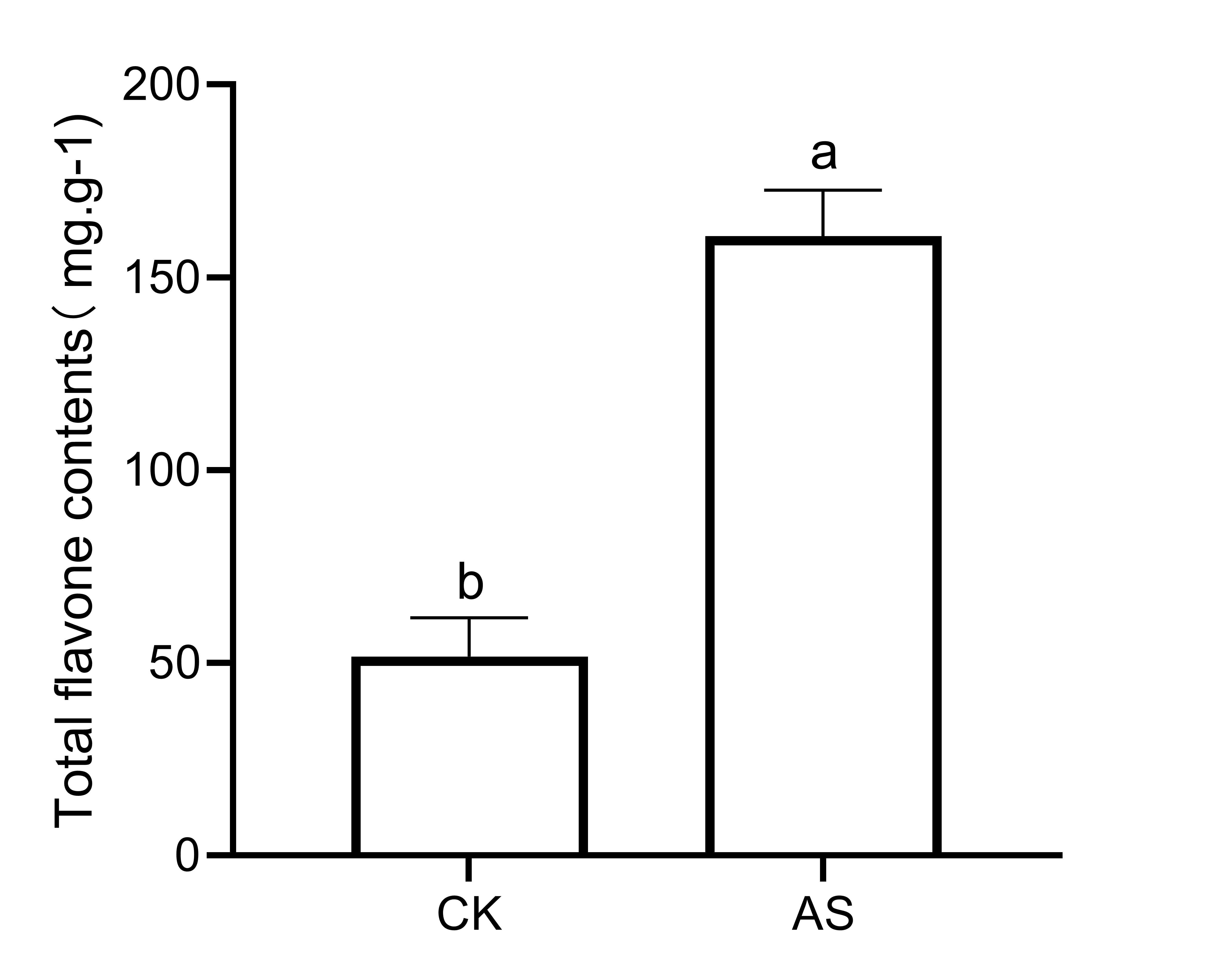

Supplement: Supplementary Figure 2 — Effects of alkali stress on total flavone contents in leaves. [file Image_2.TIF]

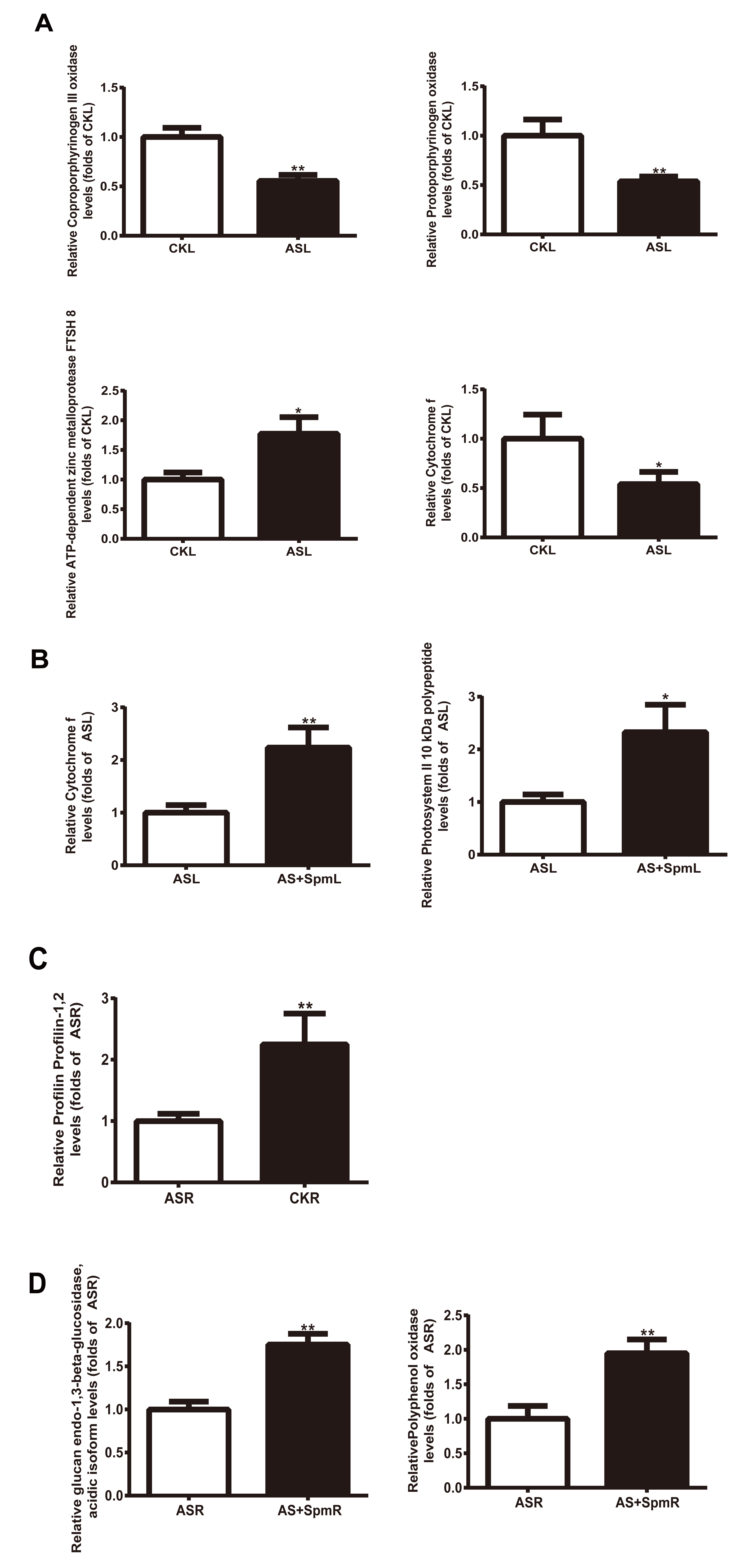

Supplement: Supplementary Figure 3 — Western blot analysis. (A) The expression levels of coproporphyrinogen III oxidase, protoporphyrinogen oxidase, ATP-dependent zinc metalloprotease FTSH 8, cytochrome f of leaves (L) at AS were normalized to CK. (B) The expression levels of cytochrome f and photosystem II 10 kDa polypeptide of leaves (L) at AS + Spm were normalized to AS. (C) The expression level of profilin of roots (R) at AS was normalized to CK. (D) The expression levels of glucan endo-1,3-beta-glucosidase, polyphenol oxidase of roots (R) at AS + Spm were normalized to AS. The results were presented as mean ± SD (n = 3). ∗p < 0.05, ∗∗p < 0.01. CK, control; AS, alkali stress (35 mmol.L–1 Na2CO3:NaHCO3 = 1:1); AS + Spm, alkali stress + spermine (35 mmol.L–1 Na2CO3:NaHCO3 + 0.01 mmol.L–1 spermine); R, roots; L, leaves. [file Image_3.TIF]

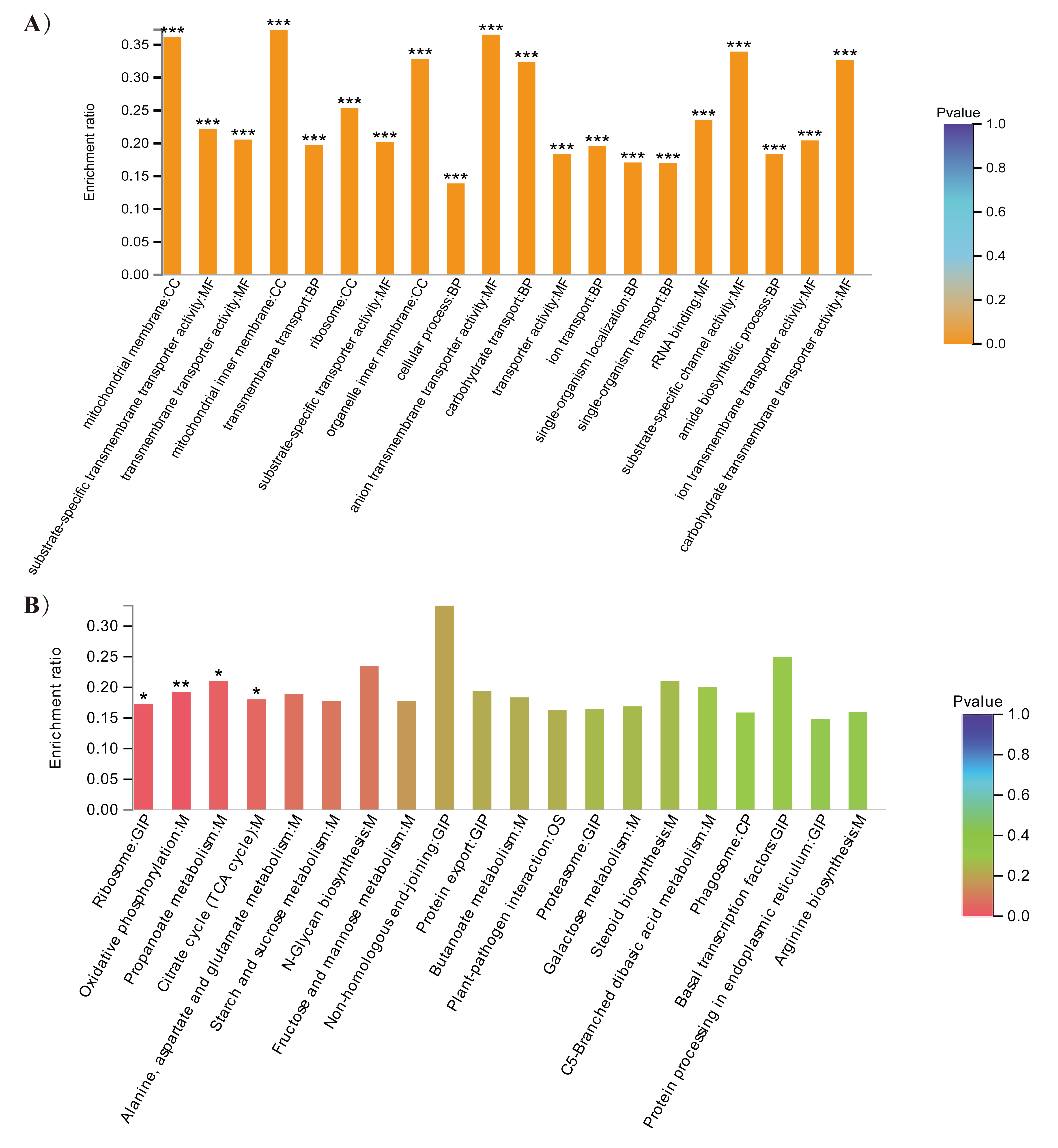

Supplement: Supplementary Figure 4 — (A) GO and (B) KEGG analysis of differentially expressed proteins in roots at CK vs. AS. The color gradient indicated significance, ∗∗∗ indicated P < 0.001, ∗∗ indicated P < 0.01, ∗ indicated P < 0.05. CK, control; AS, alkali stress (35 mmol.L–1 Na2CO3:NaHCO3 = 1:1). [file Image_4.TIF]

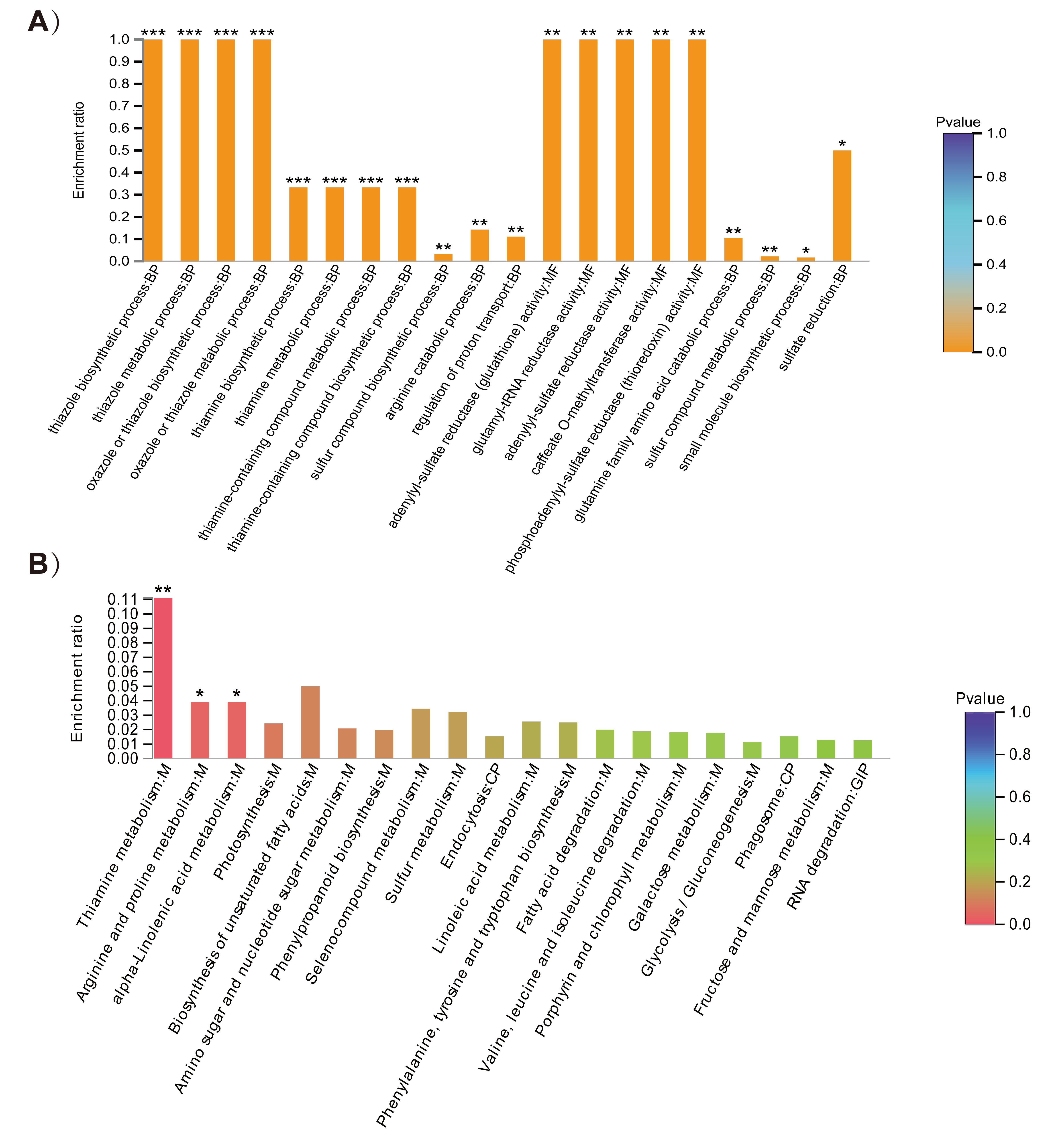

Supplement: Supplementary Figure 5 — (A) GO and (B) KEGG analysis of differentially expressed proteins in leaves at AS vs. Spm. The color gradient indicated significance, ∗∗∗ indicated P < 0.001, ∗∗ indicated P < 0.01, ∗ indicated P < 0.05. AS, alkali stress (35 mmol.L–1 Na2CO3:NaHCO3 = 1:1); AS + Spm, 35 mmol.L–1 Na2CO3:NaHCO3(1:1) + 0.01 mmol.L–1 spermine. [file Image_5.TIF]

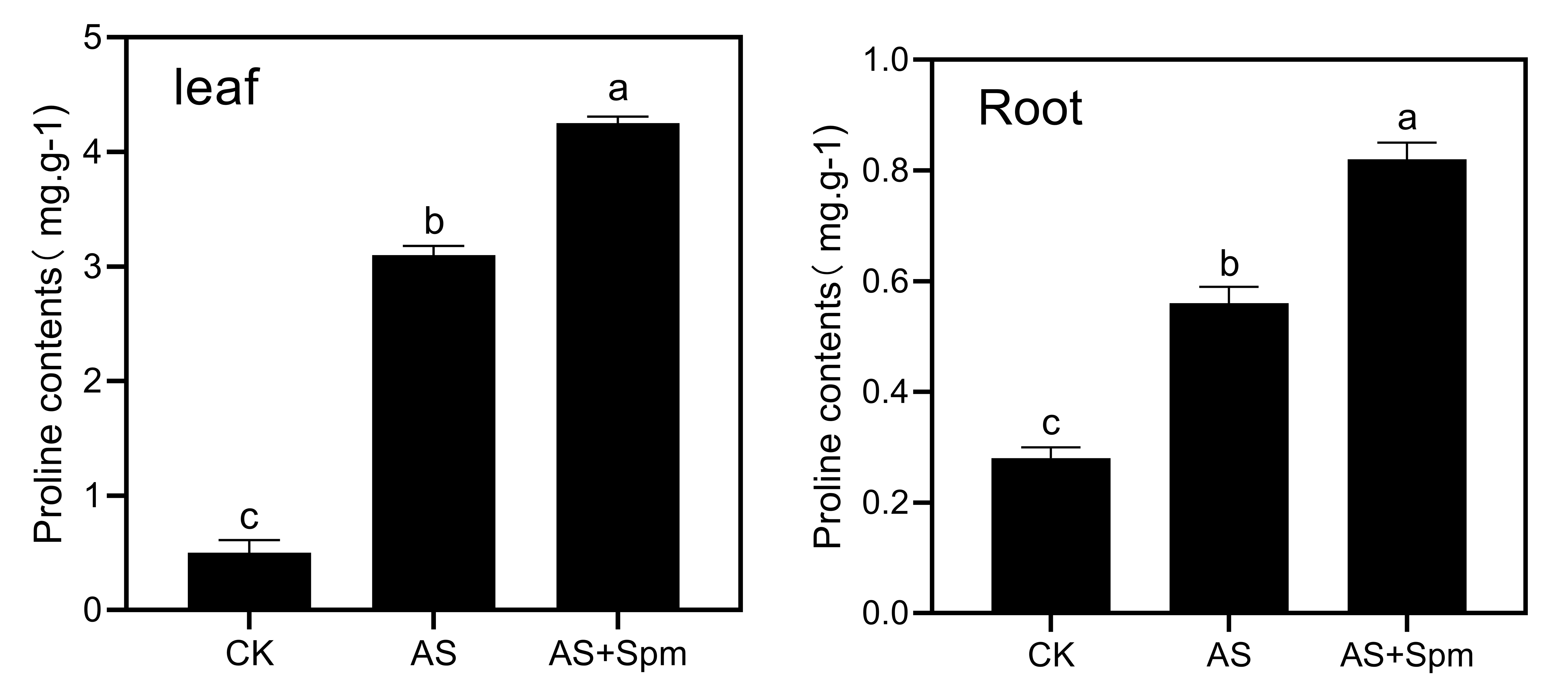

Supplement: Supplementary Figure 6 — Effects of spermine on proline contents under alkali stress. CK, control; AS, alkali stress (35 mmol.L–1 Na2CO3:NaHCO3 = 1:1); AS + Spm, 35 mmol.L–1 Na2CO3:NaHCO3(1:1) + 0.01 mmol.L–1 spermine. [file Image_6.TIF]

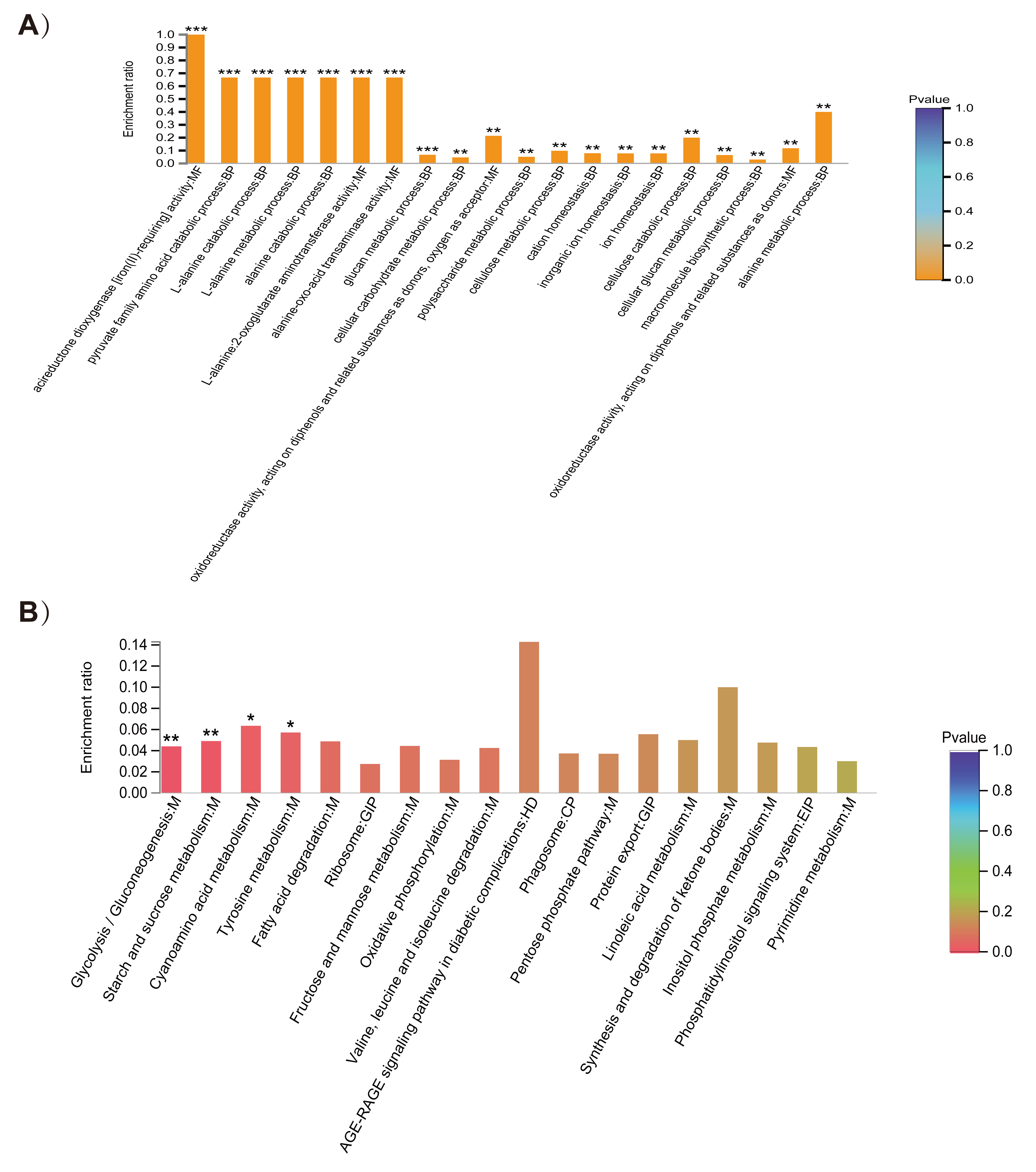

Supplement: Supplementary Figure 7 — (A) GO and (B) KEGG analysis of differentially expressed proteins in roots at AS vs. AS + Spm. The color gradient indicated significance, ∗∗∗ indicated P < 0.001, ∗∗ indicated P < 0.01, ∗ indicated P < 0.05. AS, alkali stress (35 mmol.L–1 Na2CO3:NaHCO3 = 1:1); AS + Spm, 35 mmol.L–1 Na2CO3:NaHCO3 (1:1) + 0.01 mmol.L–1 spermine. [file Image_7.TIF]

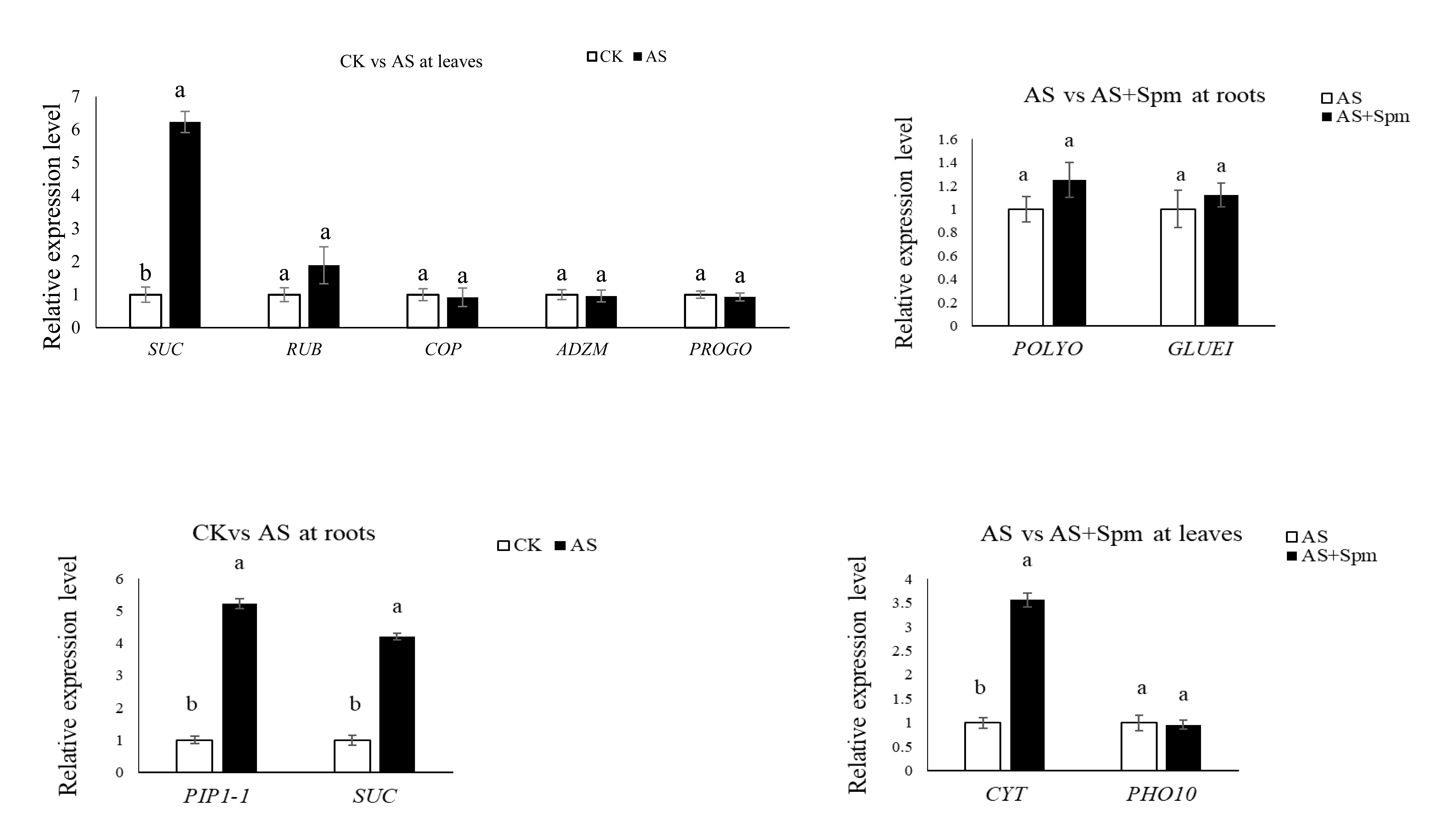

Supplement: Supplementary Figure 8 — The quantitative RT-PCR analysis of some genes. The values are the mean ± SE of three independent biological replicates normalized against the reference gene Actin. Varied letters: significant differences at the 0.05 level. SUC, sucrose synthase type 3; RUB, RuBisCO large subunit-binding protein subunit beta; COP, coproporphyrinogen III oxidase; ADZM, ATP-dependent zinc metalloprotease FTSH 8; PROGO, protoporphyrinogen oxidase; CYT, cytochrome f; PHO10, photosystem II 10 kDa polypeptide; PIP1-1, aquaporin PIP1-1; POLYO, polyphenol oxidase; GLUEI, glucan endo-1,3-beta-glucosidase, acidic isoform. [file Image_8.TIF]
