# Supplementary material for: Proteomic Responses to Alkali Stress in Oats and the Alleviatory Effects of Exogenous Spermine Application
Source: Front Plant Sci. 2021 Apr 1;12:627129. doi: 10.3389/fpls.2021.627129 (PMC8049610; doi:10.3389/fpls.2021.627129)
Supplement: Supplementary file 9 [file Table_1.pdf]

SUPPLEMENTAL TABLE 1 List of treatments used in the greenhouse study

Treatment	Abbreviation	Implementation
Control	Ck	Control (Hoagland solution)
Alkali stress	AS	Na_2CO_3:$NaHCO_3$ = 1:1, 35 mmol.L^{-1}
Alkali stress + spermine	AS+Spm	35mmol.L^{-1}(Na_2CO_3:$NaHCO_3$=1:1)+0.01mmol.L^{-1}spermine (Sigma-Aldrich Company, St. Louis, MO, USA)
