# Supplementary material for: Proteomic Responses to Alkali Stress in Oats and the Alleviatory Effects of Exogenous Spermine Application
Source: Front Plant Sci. 2021 Apr 1;12:627129. doi: 10.3389/fpls.2021.627129 (PMC8049610; doi:10.3389/fpls.2021.627129)
Supplement: Supplementary file 10 [file Table_2.pdf]

THE SUPPLEMENTAL TABLE 2: The DEPs of leaves at AS vs Ck

Protein ID	Description	Ratio	P-value	Go number
	**Up-regulation**			
TRINITY_DN399578_c7_g1_i1_m.2999402	thionin Asthi5	3.27	0.0142	GO:0006952;GO:0005576
TRINITY_DN399320_c9_g2_i1_m.1326337	17.9 kDa class I heat shock protein	4.37	0.0054	GO:0042542;GO:0045471;GO:0046688;GO:0046686;GO:0009408;GO:0046685;GO:0005737;GO:0005634
TRINITY_DN372719_c0_g1_i1_m.3121704	pathogenesis-related protein 1-like	7.04	0.0222	GO:0005576
TRINITY_DN879951_c0_g1_i1_m.3411043	Heat shock protein 83	5.86	0.0043	GO:0006950;GO:0006457;GO:0051082;GO:0005524
TRINITY_DN379065_c2_g3_i5_m.1429370	class IV endochitinase	6.72	0.0352	GO:0006032;GO:0005975;GO:0016998;GO:0004568;GO:0008061
TRINITY_DN370601_c5_g5_i1_m.958768	heat shock protein 82	5.03	0.0094	GO:0006950;GO:0006457;GO:0051082;GO:0005524
TRINITY_DN390772_c2_g1_i4_m.1675804	Thaumatin-like pathogenesis-related protein 1	8.65	0.0156	GO:0050832;GO:0031640;GO:0008152;GO:0052861;GO:0052862
TRINITY_DN368790_c0_g1_i4_m.1346525	cold regulated protein	4.75	0.0001	GO:0016021
TRINITY_DN399662_c4_g1_i1_m.3204749	beta-1,3-glucanase 2a	5.11	0.0174	GO:0005975;GO:0046658;GO:0030247;GO:0004553
TRINITY_DN372719_c1_g4_i2_m.3121728	PR-1a pathogenesis related protein	5.89	0.0238	GO:0006952;GO:0009607;GO:0005576
TRINITY_DN355421_c2_g2_i2_m.2825146	glucan endo-1,3-beta-glucosidase	4.49	0.0196	GO:0005975;GO:0046658;GO:0030247;GO:0004553
TRINITY_DN391252_c3_g1_i21_m.2777254	endonuclease 2-like	3.94	0.0010	GO:0006308;GO:0090305;GO:0004519;GO:0003676
TRINITY_DN390772_c2_g2_i1_m.1675786	Thaumatin-like pathogenesis-related protein 4	2.77	0.0126	GO:0050832;GO:0031640
TRINITY_DN235131_c0_g1_i1_m.4068132	heat shock cognate 70 kDa protein-like	3.50	0.0022	GO:0005524
TRINITY_DN390708_c1_g1_i1_m.1671723	Ribosome-inactivating protein 9	2.50	0.0007	GO:0016787
TRINITY_DN364677_c2_g1_i1_m.2315834	pathogenesis-related protein 4	4.22	0.0243	GO:0050832;GO:0042742
TRINITY_DN399662_c4_g1_i9_m.3204787	Glu1 protein	4.40	0.0258	GO:0005975;GO:0046658;GO:0030247;GO:0004553
TRINITY_DN374300_c3_g2_i1_m.2303058	heat shock protein 90	3.29	0.0077	GO:0009816;GO:0006457;GO:0051082;GO:0005524
TRINITY_DN396451_c2_g2_i8_m.2063441	aleurone ribonuclease	2.82	0.0000	GO:0090502;GO:0033897;GO:0003723
TRINITY_DN392222_c1_g2_i3_m.1911262	AF097363_1 heat shock protein 101	3.08	0.0021	GO:0019538;GO:0034605;GO:0005829;GO:0005634;GO:0005524
TRINITY_DN318416_c0_g1_i3_m.916773	AAA-ATPase At3g50940-like	4.16	0.0061	GO:0005524
TRINITY_DN84857_c0_g1_i1_m.4366601	AF251264_1 ribulose bisphosphate carboxylase activase B	3.49	0.0041	GO:0009507;GO:0004633;GO:0005524
TRINITY_DN395707_c2_g5_i2_m.1780277	heat-shock protein 70	3.09	0.0047	GO:0005524
TRINITY_DN359987_c1_g1_i1_m.2681357	Naringenin,2-oxoglutarate 3-dioxygenase	2.84	0.0193	GO:0055114;GO:0046872;GO:0051213
TRINITY_DN395589_c2_g1_i1_m.3039913	24.1 kDa heat shock protein, mitochondrial isoform X1	2.94	0.0044	GO:0009408;GO:0006970;GO:0005739
TRINITY_DN390708_c0_g1_i2_m.1671719	Ribosome-inactivating protein 9	2.07	0.0012	GO:0016787
TRINITY_DN399390_c2_g1_i5_m.1328649	chaperone protein ClpB1	2.44	0.0026	GO:0019538;GO:0034605;GO:0005829;GO:0005634;GO:0005524
TRINITY_DN383094_c0_g2_i9_m.2882453	putative 5'-adenylylsulfate reductase 1	2.56	0.0440	GO:0019419;GO:0019344;GO:0045454;GO:0009507;GO:0033741;GO:0051539;GO:0009973;GO:0004604;GO:0046872
TRINITY_DN395289_c5_g1_i1_m.1374412	70 kDa peptidyl-prolyl isomerase-like	2.06	0.0107	GO:0061077;GO:0000413;GO:0005789;GO:0005528;GO:0003755
TRINITY_DN395589_c2_g1_i2_m.3039972	24.1 kDa heat shock protein	1.88	0.0022	GO:0009408;GO:0006970;GO:0005739
TRINITY_DN396243_c2_g1_i3_m.1713069	serine carboxypeptidase-like 18	3.26	0.0167	GO:0051603;GO:0019748;GO:0016021;GO:0016747;GO:0004185
TRINITY_DN397454_c3_g2_i7_m.1055786	carotenoid cleavage dioxygenase	1.73	0.0050	GO:0016124;GO:0016121;GO:0055114;GO:0005794;GO:0005774;GO:0005886;GO:0009506;GO:0045549
TRINITY_DN388636_c1_g3_i8_m.1586781	putative delta-1-pyrroline-5-carboxylate 1	1.70	0.0053	GO:0006561;GO:0016310;GO:0055114;GO:0005737;GO:0004349;GO:0004350
TRINITY_DN371641_c2_g3_i2_m.2887268	NADH dehydrogenase subunit 6	1.56	0.0118	GO:0055114;GO:0016021;GO:0005747;GO:0008137
TRINITY_DN363572_c0_g1_i7_m.1172083	CTP synthase 1	2.07	0.0086	GO:0044210;GO:0006541;GO:0003883;GO:0005524
TRINITY_DN393816_c2_g1_i3_m.3302704	UDP-glucoronosyl and UDP-glucosyl transferase	1.57	0.0056	GO:0009813;GO:0052696;GO:0043231;GO:0080043;GO:0080044

TRINITY_DN399921_c10_g3_i3_m.1627378	Pleiotropic drug resistance protein 4	2.22	0.0106	GO:0055085;GO:0016021;GO:0005739;GO:0005886;GO:0042626;GO:0005524
TRINITY_DN399576_c1_g1_i1_m.3002630	lipid transfer protein 7a2b	1.50	0.0031	GO:0009751;GO:0042542;GO:0006869;GO:0009611;GO:0009723;GO:0008289
TRINITY_DN386347_c2_g4_i1_m.1128474	Acidic endochitinase	2.72	0.0103	GO:0005975;GO:0006032;GO:0005576;GO:0004568;GO:0008061
TRINITY_DN365517_c0_g1_i1_m.1183396	cell division AAA ATPase family protein	1.81	0.0209	GO:0051301;GO:0016021;GO:0005524
TRINITY_DN392303_c0_g1_i17_m.1910299	DNA topoisomerase 6 subunit B isoform X1	1.27	0.0355	GO:0007389;GO:0009741;GO:0042254;GO:0042023;GO:0006265;GO:0010026;GO:0006412;GO:0000902;GO:0015935;GO:0009330;GO:0005634;GO:0005829;GO:0003918;GO:0003677;GO:0042803;GO:0005524
TRINITY_DN381488_c4_g1_i1_m.1246590	glycine-rich RNA-binding protein 2	1.40	0.0039	GO:0003676;GO:0000166
TRINITY_DN264107_c0_g2_i1_m.3960027	ABC transporter, ATPbinding domain containing protein	1.89	0.0035	GO:0016021;GO:0005524;GO:0016887
TRINITY_DN384601_c2_g1_i12_m.2355839	L-ascorbate peroxidase 1	1.43	0.0030	GO:0098869;GO:0042744;GO:0006979;GO:0055114;GO:0005737;GO:0020037;GO:0016688;GO:0046872
TRINITY_DN151429_c0_g1_i1_m.650610	B12D protein	1.72	0.0009	GO:0016021
TRINITY_DN392960_c6_g2_i2_m.2522375	1,3-beta glucanase	1.51	0.0163	GO:0005975;GO:0042973
TRINITY_DN391482_c2_g1_i9_m.3348142	chitinase 2	1.57	0.0320	GO:0016998;GO:0031640;GO:0000272;GO:0050832;GO:0006032;GO:0005576;GO:0008061;GO:0004568
TRINITY_DN388813_c1_g1_i1_m.1730164	Thioredoxin H-type 4	1.33	0.0043	GO:0006662;GO:0006457;GO:0009414;GO:0045454;GO:0000103;GO:0034599;GO:0055114;GO:0009941;GO:0009570;GO:0015035;GO:0016671
TRINITY_DN376808_c1_g2_i3_m.1136526	actin depolymerization factor-like protein	1.39	0.0045	GO:0030042;GO:0015629;GO:0003779
TRINITY_DN368820_c0_g1_i4_m.3056111	Putative invertase inhibitor	1.63	0.0003	GO:0043086;GO:0004857
TRINITY_DN381333_c0_g2_i7_m.1333536	cardiolipin synthase	1.31	0.0104	GO:0046474;GO:0016020;GO:0005739;GO:0009941;GO:0008444
TRINITY_DN379801_c2_g1_i3_m.2459734	vacuolar-processing enzyme beta-isozyme 1-like	1.79	0.0120	GO:0006624;GO:0051603;GO:0005773;GO:0004197
TRINITY_DN396009_c6_g1_i1_m.1773691	Trans-cinnamate 4-monooxygenase	1.51	0.0041	GO:0009808;GO:0044550;GO:0055114;GO:0016020;GO:0020037;GO:0016710;GO:0005506
TRINITY_DN376353_c0_g1_i5_m.3133467	activator of 90 kDa heat shock protein ATPase homolog	1.50	0.0022	GO:0006457;GO:0042542;GO:0032781;GO:0009408;GO:0009644;GO:0034976;GO:0005829;GO:0051087;GO:0001671
TRINITY_DN351452_c2_g1_i11_m.2840857	glucose-6-phosphate 1-dehydrogenase	1.29	0.0163	GO:0006098;GO:0006006;GO:0055114;GO:0016021;GO:0050661;GO:0004345
TRINITY_DN373131_c1_g1_i8_m.2497947	cysteine proteinase inhibitor 1	1.42	0.0136	GO:0010951;GO:0006952;GO:0005576;GO:0004869;GO:0008233
TRINITY_DN387562_c5_g1_i10_m.2262265	xylanase inhibitor protein 1-like	1.74	0.0038	GO:0006032;GO:0045493;GO:0005576;GO:0004568;GO:0008061
TRINITY_DN395470_c1_g1_i3_m.2730581	Arginine decarboxylase	1.40	0.0039	GO:0033388;GO:0006527;GO:0008295;GO:0008792
TRINITY_DN398089_c1_g1_i4_m.947850	Gamma-glutamyltranspeptidase 1	1.81	0.0057	GO:0006749;GO:0016021;GO:0003840
TRINITY_DN389469_c2_g2_i6_m.1812626	Pathogenesis-related protein STH-21	2.68	0.0244	GO:0050896;GO:0050790;GO:0050794;GO:0044424
TRINITY_DN385076_c1_g1_i14_m.2193414	universal stress protein	2.06	0.0011	GO:0006950
TRINITY_DN395707_c2_g3_i1_m.1780257	probable mediator of RNA polymerase II transcription subunit 37c	1.77	0.0019	GO:0005524
TRINITY_DN398030_c2_g1_i9_m.953114	beta-glucosidase 4	1.31	0.0445	GO:1901657;GO:0030245;GO:0005829;GO:0102483;GO:0008422
TRINITY_DN364178_c2_g1_i14_m.3312599	fructosyltransferase	1.56	0.0033	GO:0005975;GO:0016021;GO:0016740;GO:0004553
TRINITY_DN387165_c0_g1_i11_m.1009884	NADH dehydrogenase [ubiquinone] 1 alpha subcomplex assembly factor 3	1.26	0.0087	GO:0006096;GO:0009570;GO:0016021;GO:0005739;GO:0030955;GO:0016301;GO:0000287;GO:0004743
TRINITY_DN365392_c0_g1_i2_m.1112484	Inositol-tetrakisphosphate 1-kinase 1	1.51	0.0250	GO:0010264;GO:0016310;GO:0032957;GO:0005622;GO:0000287;GO:0047325;GO:0052726;GO:0052725;GO:0005524
TRINITY_DN373400_c0_g3_i4_m.2732467	papain-like cysteine proteinase	1.51	0.0022	GO:0051603;GO:0005764;GO:0005615;GO:0004197
TRINITY_DN371370_c0_g2_i2_m.2330748	allene oxide cyclase 3	1.24	0.0122	GO:0009507;GO:0046423
TRINITY_DN355749_c0_g1_i6_m.1741158	ATP-dependent zinc metalloprotease FTSH 8	1.24	0.0089	GO:0045041;GO:0006461;GO:0008053;GO:0034982;GO:0042407;GO:0051301;GO:0009535;GO:0016021;GO:0005743;GO:0004176;GO:0004222;GO:0005524;GO:0008270
TRINITY_DN396243_c3_g1_i6_m.1713087	serine carboxypeptidase-like 18	2.20	0.0092	GO:0051603;GO:0019748;GO:0016021;GO:0016747;GO:0004185

TRINITY_DN393369_c6_g1_i5_m.2011168	non-specific lipid-transfer protein 2B	1.28	0.0234	GO:0006869;GO:0008289
TRINITY_DN395640_c3_g1_i10_m.2551466	Xylem cysteine proteinase 1	1.22	0.0144	GO:0006508;GO:0008234
TRINITY_DN394651_c0_g1_i11_m.1460781	L-galactose dehydrogenase	1.24	0.0081	GO:0019853;GO:0055114;GO:0005829;GO:0010349
TRINITY_DN370006_c2_g1_i3_m.2931555	Putative UDP-glucosyltransferase	1.75	0.0113	GO:0009813;GO:0052696;GO:0043231;GO:0080043;GO:0080044
TRINITY_DN375378_c1_g2_i12_m.2517118	basic blue protein-like	1.23	0.0136	GO:0046658;GO:0009055
TRINITY_DN218686_c0_g1_i1_m.3804922	Germin-like protein 5-1	1.57	0.0461	GO:0010497;GO:2000280;GO:0033609;GO:0005618;GO:0048046;GO:0009506;GO:0030145;GO:0045735;GO:0046564
TRINITY_DN351582_c0_g1_i1_m.1079127	Cytochrome P450 71D7	1.87	0.0028	GO:0044550;GO:0055114;GO:0016021;GO:0020037;GO:0016709;GO:0005506
TRINITY_DN383179_c0_g1_i4_m.2790891	deoxyhypusine hydroxylase-B	1.25	0.0389	GO:0008612;GO:0055114;GO:0019135;GO:0046872
TRINITY_DN364527_c0_g2_i3_m.2707292	multiple organellar RNA editing factor 9	1.21	0.0064	GO:0005739
TRINITY_DN393034_c3_g1_i2_m.1155727	protein disulfide isomerase family protein TaPDIL1-1gamma	1.30	0.0360	GO:0045454;GO:0034975;GO:0009960;GO:0005788;GO:0003756
TRINITY_DN369868_c0_g1_i3_m.2235934	probable uridine nucleosidase 2	1.38	0.0207	GO:0006152;GO:0005829;GO:0008477;GO:0046872;GO:0045437
TRINITY_DN393940_c2_g1_i2_m.1536269	Bifunctional dihydroflavonol 4-reductase/flavanone 4-reductase	1.50	0.0027	GO:0050662;GO:0003824
TRINITY_DN381735_c1_g3_i2_m.1787082	Zn-dependent hydrolases of the beta-lactamase fold	1.50	0.0004	GO:0009536;GO:0016787
TRINITY_DN377608_c2_g3_i1_m.3222128	tuliposide A-converting enzyme 2	2.45	0.0064	GO:0008152;GO:0016787
TRINITY_DN377472_c5_g1_i5_m.1100033	thioredoxin-like protein AAED1	1.24	0.0093	GO:0098869;GO:0055114;GO:0009570;GO:0004601;GO:0051920
TRINITY_DN397359_c8_g2_i1_m.2205001	heat shock cognate 70 kDa protein 2	1.32	0.0033	GO:0005524
TRINITY_DN375894_c0_g1_i3_m.1096464	electron transfer flavoprotein subunit beta	1.37	0.0007	GO:0015996;GO:0006552;GO:0019243;GO:0055114;GO:0005759;GO:0009055
TRINITY_DN370637_c1_g1_i21_m.2218331	Cytochrome c oxidase copper chaperone	1.33	0.0160	GO:0006825;GO:0005758;GO:0016531
TRINITY_DN355614_c0_g1_i2_m.1110037	RNA-binding protein 42-like isoform X2	1.43	0.0005	GO:0003676;GO:0000166
TRINITY_DN363420_c1_g1_i1_m.1301812	putative glutathione S-transferase GSTU6	1.55	0.0016	GO:0006749;GO:0009407;GO:0005737;GO:0004364
TRINITY_DN392532_c3_g1_i18_m.1491435	cathepsin B-like	1.49	0.0046	GO:0051603;GO:0050790;GO:0005764;GO:0005615;GO:0004197
TRINITY_DN350722_c0_g1_i4_m.972352	Heat shock 70 kDa protein 4L	1.28	0.0038	GO:0005524
TRINITY_DN367766_c0_g1_i8_m.1693602	sucrose synthase type 3	1.80	0.0015	GO:0005985;GO:0016157
TRINITY_DN387604_c2_g1_i17_m.2263455	chaperone protein dnaJ 15	1.28	0.0433	GO:0009958
TRINITY_DN383905_c1_g2_i2_m.1888647	receptor-like protein kinase FERONIA	1.26	0.0097	GO:0006468;GO:0016021;GO:0004674;GO:0005524
TRINITY_DN391088_c0_g1_i2_m.3322774	E3 ubiquitin-protein ligase KEG	1.26	0.0147	GO:0006468;GO:0010260;GO:0016021;GO:0009507;GO:0004674;GO:0016874;GO:0005524
TRINITY_DN399934_c13_g1_i5_m.1625881	translational activator GCN1 isoform X2	1.30	0.0022	GO:0033674;GO:0045859;GO:0033554;GO:0006417;GO:0019901;GO:0043022;GO:0019887
TRINITY_DN384096_c2_g1_i6_m.1266013	ERAD-associated E3 ubiquitin-protein ligase component HRD3	1.22	0.0056	GO:0016021;GO:0005739;GO:0005789
TRINITY_DN383994_c1_g1_i5_m.2788659	outer envelope pore protein 37	1.26	0.0004	GO:0009536;GO:0019031
TRINITY_DN392994_c7_g1_i6_m.2526673	cinnamoyl-CoA reductase 1-like	1.26	0.0232	GO:0016021;GO:0050662;GO:0003824
TRINITY_DN355884_c0_g1_i6_m.3318033	probable L-ascorbate peroxidase 7	1.27	0.0255	GO:0098869;GO:0042744;GO:0006979;GO:0055114;GO:0031969;GO:0009570;GO:0016021;GO:0020037;GO:0016688;GO:0046872
TRINITY_DN398551_c4_g4_i3_m.1119509	Methionine S-methyltransferase	1.32	0.0112	GO:0090502;GO:0009058;GO:0001887;GO:0032259;GO:0046500;GO:0005829;GO:0030732;GO:0004523;GO:0030170;GO:0003676
TRINITY_DN275333_c0_g1_i4_m.3878305	UDP-glycosyltransferase 74F2-like	1.53	0.0140	GO:0009813;GO:0052696;GO:0043231;GO:0080043;GO:0080044
TRINITY_DN385576_c1_g1_i4_m.2336667	Chalcone synthase 8	1.20	0.0202	GO:0009058;GO:0016747
TRINITY_DN329781_c0_g1_i1_m.3164828	1-aminocyclopropane-1-carboxylate oxidase-1-like protein	1.64	0.0027	GO:0055114;GO:0005506;GO:0051213
TRINITY_DN649927_c0_g1_i1_m.74896	ATP synthase subunit alpha	1.28	0.0023	GO:0015986;GO:0015991;GO:0045261;GO:0046933;GO:0046961;GO:0005524
TRINITY_DN393647_c1_g1_i7_m.1036534	protein-L-isoaspartate O-methyltransferase	1.41	0.0003	GO:0006479;GO:0009536;GO:0004719

TRINITY_DN398609_c1_g1_i1_m.2657042	Calcium-transporting ATPase 4	1.24	0.0158	GO:0098655;GO:0005783;GO:0005887;GO:0019829;GO:0015662;GO:0046872;GO:0005524
TRINITY_DN398155_c2_g3_i5_m.3074189	Calcium-transporting ATPase 1	1.46	0.0395	GO:0070588;GO:0043231;GO:0005887;GO:0005516;GO:0005388;GO:0046872;GO:0005524
TRINITY_DN235919_c0_g1_i1_m.4033131	enolase 2	1.30	0.0300	GO:0006096;GO:0000015;GO:0000287;GO:0004634
TRINITY_DN399738_c4_g1_i2_m.3143721	AP-2 complex subunit sigma-like isoform X1	1.30	0.0381	GO:0015031;GO:0008565
TRINITY_DN398923_c2_g3_i2_m.1280142	Dihydrolipoyllysine-residue acetyltransferase component of pyruvate dehydrogenase complex	1.52	0.0069	GO:0008152;GO:0009536;GO:0016746
TRINITY_DN389757_c0_g2_i1_m.996723	Farnesyl pyrophosphate synthase	1.71	0.0051	GO:0045337;GO:0005737;GO:0004337;GO:0004161
TRINITY_DN372888_c3_g1_i1_m.1411071	heat shock protein 81-1	1.24	0.0136	GO:0006950;GO:0006457;GO:0005737;GO:0051082;GO:0005524
TRINITY_DN393667_c4_g2_i4_m.1033327	Luminal-binding protein 4	1.26	0.0477	GO:0005788;GO:0005524
TRINITY_DN397454_c2_g2_i5_m.1055708	carotenoid 9,1	1.31	0.0006	GO:0016124;GO:0016121;GO:0055114;GO:0005794;GO:0016021;GO:0005774;GO:0005886;GO:0009506;GO:0045549
TRINITY_DN370841_c2_g1_i1_m.1168984	SAR-like protein	1.20	0.0155	GO:0006888;GO:0006886;GO:0005794;GO:0005783;GO:0005215;GO:0005525
TRINITY_DN394721_c1_g1_i9_m.2627326	vacuolar-sorting receptor 6	1.24	0.0022	GO:0016021;GO:0005509
TRINITY_DN396833_c1_g1_i7_m.2569603	protein argonaute 1C-like	1.23	0.0029	GO:0031047;GO:0005739;GO:0003676
TRINITY_DN398761_c4_g1_i1_m.1998555	DnaJ protein 2-like protein	1.38	0.0022	GO:0006457;GO:0009408;GO:0031072;GO:0051082;GO:0046872;GO:0005524
TRINITY_DN395511_c1_g2_i2_m.3041349	neutral/alkaline invertase 1	1.32	0.0115	GO:0005739;GO:0033926
TRINITY_DN449407_c0_g1_i1_m.808047	alpha-galactosidase A	2.29	0.0106	GO:0004553
TRINITY_DN397591_c3_g1_i8_m.2299305	LETM1 and EF-hand domain-containing protein 1	1.37	0.0035	GO:0016021;GO:0005739;GO:0043022;GO:0005509
TRINITY_DN397168_c1_g2_i2_m.3282508	phospholipase D	1.27	0.0011	GO:0016042;GO:0046470;GO:0016020;GO:0070290;GO:0005509;GO:0004630
TRINITY_DN388147_c2_g1_i9_m.1808976	Monodehydroascorbate reductase	1.30	0.0023	GO:0022900;GO:0045454;GO:0005623;GO:0050660;GO:0016656;GO:0015036
TRINITY_DN399896_c2_g1_i2_m.2042929	AF129479_1 HAK2	1.22	0.0123	GO:0071805;GO:0016021;GO:0015079
TRINITY_DN390007_c0_g1_i7_m.1201051	probable protein phosphatase 2C 70 isoform X1	1.35	0.0013	GO:0006470;GO:0046872;GO:0004722
TRINITY_DN202479_c1_g1_i1_m.3959788	catalase	1.41	0.0012	GO:0042542;GO:0098869;GO:0042744;GO:0055114;GO:0009514;GO:0020037;GO:0004096;GO:0046872
TRINITY_DN394946_c10_g1_i1_m.2429604	anthranilate synthase alpha 2 subunit	1.22	0.0416	GO:0000162;GO:0005950;GO:0009507;GO:0004049
TRINITY_DN369493_c1_g1_i1_m.1470675	probable phospholipid hydroperoxide glutathione peroxidase	1.35	0.0008	GO:0048831;GO:0055114;GO:2000280;GO:0098869;GO:0006508;GO:0006979;GO:0005739;GO:0004252;GO:0004602
TRINITY_DN362598_c0_g1_i16_m.2022289	stress-related protein	1.29	0.0125	GO:0016021
TRINITY_DN382230_c2_g1_i11_m.1497946	Acetylornithine deacetylase	1.23	0.0002	GO:0008152;GO:0016787
TRINITY_DN397656_c1_g2_i6_m.3161266	AF310160_1 sucrose-phosphate synthase	1.23	0.0040	GO:0005986;GO:0005886;GO:0046524
TRINITY_DN376173_c1_g1_i2_m.1049863	glutathione transferase GST 23-like isoform X2	1.56	0.0273	GO:0006952;GO:0006749;GO:0009407;GO:0005737;GO:0004364
TRINITY_DN397445_c4_g1_i1_m.1053704	heat shock cognate protein 80-like	1.46	0.0197	GO:0006950;GO:0006457;GO:0005737;GO:0051082;GO:0005524
TRINITY_DN393733_c0_g2_i1_m.3179912	endoplasmic reticulum-Golgi intermediate compartment protein 3-like	1.20	0.0091	GO:0016021
TRINITY_DN393034_c3_g1_i2_m.1155725	disulfide isomerase	1.22	0.0229	GO:0009960;GO:0045454;GO:0034975;GO:0034976;GO:0005788;GO:0003756
TRINITY_DN393314_c2_g3_i1_m.2012271	glutathione transferase	1.40	0.0013	GO:0006749;GO:0009407;GO:0005737;GO:0004364
TRINITY_DN397107_c2_g1_i1_m.3281948	outer envelope protein 80	1.23	0.0130	GO:0009658;GO:0019031;GO:0009707;GO:0005739
TRINITY_DN375474_c1_g1_i1_m.1512117	probable histone deacetylase 19	1.26	0.0088	GO:0006355;GO:0070932;GO:0005634;GO:0032041
TRINITY_DN342110_c0_g1_i1_m.3308010	alpha-glucosidase 2	1.31	0.0055	GO:0005975;GO:0009507;GO:0030246;GO:0004553
TRINITY_DN388550_c1_g1_i5_m.3153458	proline synthase co-transcribed bacterial homolog protein	1.27	0.0042	GO:0005622;GO:0030170
TRINITY_DN985487_c0_g1_i1_m.346269	presequence protease 2	1.31	0.0142	GO:0016485;GO:0004222;GO:0008270
TRINITY_DN362008_c0_g5_i1_m.1088163	Acetolactate synthase small subunit	1.21	0.0011	GO:0009082;GO:0009536;GO:0003984;GO:0016597
TRINITY_DN399508_c3_g1_i1_m.2998943	cytochrome P450	1.65	0.0171	GO:0055114;GO:0016021;GO:0004497;GO:0020037;GO:0016705;GO:0005506
TRINITY_DN375667_c0_g1_i6_m.1959100	: vacuolar-processing enzyme	1.21	0.0347	GO:0006624;GO:0051603;GO:0005773;GO:0004197

TRINITY_DN380334_c6_g1_i3_m.1209649	Leucoanthocyanidin reductase	1.42	0.0040	GO:0050662;GO:0003824
TRINITY_DN391562_c1_g1_i4_m.2835564	ruBisCO large subunit-binding protein subunit beta	1.26	0.0060	GO:0042026;GO:0009536;GO:0005524
TRINITY_DN395714_c2_g2_i5_m.1783572	Putative LRR receptor-like serine/threonine-protein kinase	1.77	0.0063	GO:0016310;GO:0016020;GO:0000166;GO:0004672
TRINITY_DN397800_c4_g1_i3_m.2924065	vacuolar ATPase subunit F	1.26	0.0345	GO:0015991;GO:0033180;GO:0046961
TRINITY_DN369780_c1_g4_i6_m.3257091	UDP-glycosyltransferase 88A1-like	2.28	0.0123	GO:0052696;GO:0009813;GO:0016021;GO:0043231;GO:0003676;GO:0080043;GO:0080044
TRINITY_DN382343_c0_g2_i3_m.2700105	Proteasome subunit beta type-4	1.26	0.0116	GO:0006511;GO:0005634;GO:0022626;GO:0005839;GO:0004298
TRINITY_DN378540_c1_g1_i8_m.3216452	serine carboxypeptidase-like 2	1.47	0.0080	GO:0051603;GO:0019748;GO:0016747;GO:0004185
TRINITY_DN399641_c1_g1_i1_m.3203499	plasma membrane ATPase 1-like	1.32	0.0112	GO:1902600;GO:0051453;GO:0006754;GO:0043231;GO:0005887;GO:0008553;GO:0046872;GO:0005524
TRINITY_DN374912_c0_g1_i8_m.931306	Catalase-1	1.26	0.0309	GO:0042542;GO:0098869;GO:0042744;GO:0055114;GO:0009514;GO:0020037;GO:0004096;GO:0046872
TRINITY_DN375552_c2_g1_i7_m.1634487	Putative fructose-bisphosphate aldolase 3	1.20	0.0253	GO:0006096;GO:0009651;GO:0006007;GO:0006979;GO:0009534;GO:0010287;GO:0005739;GO:0004332
TRINITY_DN396243_c3_g5_i1_m.1713091	Serine carboxypeptidase-like 6	1.43	0.0071	GO:0051603;GO:0019748;GO:0016021;GO:0016747;GO:0004185
TRINITY_DN390286_c1_g2_i2_m.2368890	protein TRI1-like	1.41	0.0312	GO:0005739
TRINITY_DN393761_c0_g1_i10_m.3180472	Phosphate carrier protein	1.71	0.0188	GO:0006810;GO:0009723;GO:0009651;GO:0006412;GO:0016021;GO:0003735
TRINITY_DN386879_c1_g3_i3_m.1227247	extracellular response kinase	1.26	0.0073	GO:0000165;GO:0005622;GO:0004707;GO:0005524
TRINITY_DN341340_c0_g1_i2_m.3021076	Putative phosphoribosylformylglycinamidine synthase	1.22	0.0206	GO:0006189;GO:0006541;GO:0009536;GO:0004642;GO:0005524
TRINITY_DN395746_c1_g1_i5_m.1782859	long chain acyl-CoA synthetase 4-like	1.28	0.0064	GO:0008152;GO:0009536;GO:0003824
TRINITY_DN382591_c2_g2_i1_m.3139076	Zinc finger protein	1.29	0.0070	GO:0005634;GO:0046872;GO:0003677
TRINITY_DN389070_c0_g2_i2_m.2984398	adenosine kinase 2	1.33	0.0067	GO:0016310;GO:0006167;GO:0006166;GO:0004001
TRINITY_DN396029_c1_g1_i7_m.1775954	uridine kinase-like protein 2	1.22	0.0004	GO:0044211;GO:0006206;GO:0016310;GO:0044206;GO:0005829;GO:0009507;GO:0016757;GO:0004849;GO:0005524
TRINITY_DN380445_c0_g2_i4_m.1296552	probable linoleate 9S-lipoxygenase 4	1.38	0.0259	GO:0031408;GO:0055114;GO:0046872;GO:0016702
TRINITY_DN362016_c1_g1_i4_m.1088179	Catalase-1	1.38	0.0036	GO:0042542;GO:0098869;GO:0042744;GO:0055114;GO:0009514;GO:0020037;GO:0004096;GO:0046872
TRINITY_DN399108_c2_g1_i2_m.912617	Presequence protease 1	1.24	0.0054	GO:0016485;GO:0005739;GO:0004222;GO:0008270
TRINITY_DN312338_c1_g1_i2_m.1402007	GTP-binding protein SAR1A	1.34	0.0025	GO:0006886;GO:0016192;GO:0005794;GO:0005783;GO:0005886;GO:0005525
TRINITY_DN397809_c1_g1_i4_m.1951808	BEL1-like homeodomain protein 7	1.35	0.0029	GO:0006355;GO:0005634;GO:0003700;GO:0003677
TRINITY_DN372799_c0_g1_i5_m.3121143	FGGY carbohydrate kinase domain-containing protein	1.32	0.0136	GO:0016310;GO:0005975;GO:0009507;GO:0016773;GO:0016301
TRINITY_DN394885_c0_g1_i7_m.1748055	putative clathrin assembly protein At2g01600	1.33	0.0346	GO:0048268;GO:0030136;GO:0030276;GO:0005545
TRINITY_DN393046_c2_g1_i1_m.1154309	Peroxidase 12	1.44	0.0132	GO:0009664;GO:0006979;GO:0098869;GO:0042744;GO:0055114;GO:0005576;GO:0009505;GO:0020037;GO:0046872;GO:0004601
TRINITY_DN399203_c7_g1_i13_m.915971	KH domain-containing protein At4g18375-like	1.21	0.0291	GO:0003723
TRINITY_DN388333_c1_g2_i1_m.1978299	AF487526_1 D1 protease-like protein precursor	1.30	0.0051	GO:0006508;GO:0031977;GO:0008236
TRINITY_DN96990_c0_g1_i1_m.4405674	NADP-dependent glyceraldehyde-3-phosphate dehydrogenase	1.21	0.0023	GO:0055114;GO:0004029;GO:0008886;GO:0009013;GO:0047100;GO:0004777
TRINITY_DN387674_c0_g1_i1_m.3108350	Putative disease resistance RPP13-like protein 3	1.31	0.0178	GO:0043531
TRINITY_DN370378_c1_g1_i6_m.2678716	peptide methionine sulfoxide reductase B1	1.32	0.0014	GO:0006979;GO:0055114;GO:0030091;GO:0009570;GO:0033743
TRINITY_DN393731_c4_g1_i7_m.3182505	Catalase isozyme 2	1.63	0.0211	GO:0042542;GO:0098869;GO:0042744;GO:0055114;GO:0009514;GO:0020037;GO:0004096;GO:0046872
TRINITY_DN394332_c0_g2_i1_m.2271943	root hair defective 3 GTP-binding protein	1.21	0.0017	GO:0016021;GO:0005789;GO:0005525;GO:0003924
TRINITY_DN393705_c0_g2_i4_m.1031238	CLP protease regulatory subunit CLPX1	1.27	0.0009	GO:0006457;GO:0030163;GO:0006508;GO:0005759;GO:0004176;GO:0051082;GO:0005524

TRINITY_DN385729_c1_g2_i8_m.1145747	phosphate transporter 6	1.50	0.0077	GO:0006817;GO:0055085;GO:0005887;GO:0022891;GO:0005315
TRINITY_DN360514_c0_g1_i2_m.1005080	L-ascorbate peroxidase 6	1.22	0.0015	GO:0098869;GO:0042744;GO:0006979;GO:0055114;GO:0009570;GO:0005739;GO:0020037;GO:0016688;GO:0046872
TRINITY_DN388983_c1_g1_i14_m.1611251	quinone oxidoreductase PIG3-like	1.79	0.0162	GO:0009610;GO:0055114;GO:0005829;GO:0004024;GO:0008270
TRINITY_DN388801_c1_g1_i17_m.2536925	pyrroline-5-carboxylate reductases	1.24	0.0037	GO:0055129;GO:0055114;GO:0005618;GO:0009536;GO:0004735
TRINITY_DN982239_c0_g1_i1_m.232546	GL22221	1.34	0.0274	GO:0006352;GO:0006334;GO:0005634;GO:0000786;GO:0003677;GO:0046982
TRINITY_DN222880_c0_g1_i2_m.3946675	Ca2+ binding protein cbp1	1.28	0.0495	GO:0016021;GO:0005739;GO:0043022;GO:0005509
TRINITY_DN397803_c1_g1_i6_m.2922870	Proteasome subunit alpha type-5	1.36	0.0035	GO:0006511;GO:0005737;GO:0019773;GO:0005634;GO:0004298
TRINITY_DN396075_c2_g1_i2_m.1774117	NADH-plastoquinone oxidoreductase subunit 1	1.23	0.0036	GO:0019684;GO:0055114;GO:0009535;GO:0016021;GO:0005886;GO:0016655;GO:0048038
TRINITY_DN388026_c0_g1_i1_m.2951443	Putative L-ascorbate peroxidase 4	1.29	0.0021	GO:0098869;GO:0042744;GO:0006979;GO:0055114;GO:0016021;GO:0020037;GO:0016688;GO:0046872
TRINITY_DN399015_c5_g2_i1_m.2650607	pyruvate decarboxylase	1.30	0.0069	GO:0000287;GO:0030976;GO:0004737
TRINITY_DN376441_c1_g1_i1_m.868690	GTP-binding protein SAR1A	1.27	0.0017	GO:0006888;GO:0006886;GO:0007264;GO:0005794;GO:0005783;GO:0005215;GO:0005525
TRINITY_DN371234_c0_g1_i6_m.1386789	flowering locus T protein	1.42	0.0047	GO:0048573;GO:0009909;GO:0008429
TRINITY_DN398914_c2_g1_i11_m.1277298	Anthocyanin 5-aromatic acyltransferase	1.53	0.0113	GO:0016747
TRINITY_DN389368_c1_g1_i6_m.2194143	UPF0061 protein azo1574	1.24	0.0021	GO:0009570
TRINITY_DN354705_c0_g1_i3_m.1683682	bisdemethoxycurcumin synthase-like	1.35	0.0057	GO:0009058;GO:0016747
TRINITY_DN397885_c2_g2_i8_m.1945623	glutamate decarboxylase 1-like	1.32	0.0376	GO:0006536;GO:0030170;GO:0004351
TRINITY_DN388026_c0_g3_i1_m.2951450	ascorbate peroxidase	1.31	0.0043	GO:0098869;GO:0042744;GO:0006979;GO:0055114;GO:0016021;GO:0020037;GO:0016688;GO:0046872
TRINITY_DN390397_c1_g1_i2_m.2620022	glyceraldehyde-3-phosphate dehydrogenase 1	1.26	0.0046	GO:0006006;GO:0006096;GO:0055114;GO:0005737;GO:0004365;GO:0051287;GO:0050661
TRINITY_DN388789_c0_g2_i1_m.2537028	folylpolyglutamate synthase-like isoform X1	1.27	0.0189	GO:0046901;GO:0006730;GO:0005739;GO:0004326;GO:0005524
TRINITY_DN391809_c1_g1_i1_m.2604206	claspin isoform X1	1.39	0.0303	GO:0003677
TRINITY_DN398835_c2_g3_i3_m.1561633	mitochondrial outer membrane protein porin 5	1.33	0.0180	GO:1903959;GO:0005741;GO:0046930;GO:0015288;GO:0008308
TRINITY_DN382354_c0_g1_i1_m.2699976	fructokinase-1	1.23	0.0155	GO:0046835;GO:0006014;GO:0019252;GO:0008865;GO:0004747;GO:0005524
TRINITY_DN394925_c4_g1_i5_m.2429950	putative mitochondrial chaperone BCS1-B	1.24	0.0263	GO:0016021;GO:0005524
TRINITY_DN379416_c0_g1_i2_m.2938809	glycine-rich RNA-binding protein RZ1C-like	1.28	0.0179	GO:0008270;GO:0003676;GO:0000166
TRINITY_DN396206_c2_g2_i11_m.1711897	ATPase family AAA domain-containing protein 2B	1.24	0.0147	GO:0031936;GO:0045944;GO:0005634;GO:0042393;GO:0016887;GO:0003682;GO:0005524
TRINITY_DN292501_c0_g1_i1_m.3773618	glutathione S-transferase GSTF1	1.22	0.0034	GO:0006749;GO:0005737;GO:0004364
TRINITY_DN371545_c0_g1_i1_m.1647135	temperature stress-induced lipocalin	1.23	0.0240	GO:0042538;GO:0009735;GO:0009416;GO:0009408;GO:0005794;GO:0005783;GO:0005739;GO:0005774;GO:0005886;GO:0009506
TRINITY_DN371545_c0_g1_i1_m.1647135	temperature stress-induced lipocalin	1.23	0.0240	GO:0042538;GO:0009735;GO:0009416;GO:0009408;GO:0005794;GO:0005783;GO:0005739;GO:0005774;GO:0005886;GO:0009506
TRINITY_DN350188_c4_g2_i4_m.1344143	guanosine nucleotide diphosphate dissociation inhibitor 2-like	1.34	0.0039	GO:0050790;GO:0015031;GO:0007264;GO:0055114;GO:0005622;GO:0005093;GO:0016491
TRINITY_DN373129_c0_g1_i12_m	mitochondrial outer membrane protein porin 3	1.23	0.0034	GO:1903959;GO:0009527;GO:0046930;GO:0005741;GO:0015288;GO:0008308
TRINITY_DN377065_c6_g2_i1_m.3007949	Beta-glucosidase 5	1.56	0.0150	GO:1901657;GO:0005975;GO:0102483;GO:0008422
TRINITY_DN382539_c1_g1_i6_m.3136170	putative 12-oxophytodienoate reductase 11	1.41	0.0081	GO:0055114;GO:0005622;GO:0003959;GO:0010181
TRINITY_DN384059_c0_g1_i4_m.1264285	putative quinone-oxidoreductase homolog	1.36	0.0222	GO:0055114;GO:0008270;GO:0016491
TRINITY_DN367632_c3_g1_i10_m.1318877	zinc finger CCCH domain-containing protein 11	1.21	0.0351	GO:0005739;GO:0046872;GO:0003677
TRINITY_DN140402_c0_g1_i1_m.461338	dihydrolipoyllysine-residue succinyltransferase	1.24	0.0232	GO:0006099;GO:0016021;GO:0045252;GO:0004149
TRINITY_DN379907_c0_g1_i10_m.2424353	cytochrome b5 isoform E	1.66	0.0179	GO:0016021;GO:0046872;GO:0020037
TRINITY_DN383511_c0_g1_i1_m.2719138	acetyl-coenzyme A synthetase	1.31	0.0055	GO:0019427;GO:0009536;GO:0003987;GO:0016208;GO:0005524
TRINITY_DN387735_c0_g1_i1_m.3235137	glutathione peroxidase W69 mutant	1.37	0.0085	GO:0098869;GO:0055114;GO:0006979;GO:0004602

TRINITY_DN396838_c12_g1_i2_m.2571383	Rubisco activase beta form precursor	1.92	0.0165	GO:0009570;GO:0003700;GO:0005524
TRINITY_DN368336_c5_g1_i3_m.2085270	H2A5_WHEAT	1.35	0.0142	GO:0005634;GO:0000786;GO:0046982;GO:0003677
TRINITY_DN392871_c1_g2_i1_m.2581201	Putative oxidoreductase	1.22	0.0057	GO:0055114;GO:0009536;GO:0016491
TRINITY_DN392947_c6_g1_i14_m.2522955	Nuclease S1	1.36	0.0111	GO:0006308;GO:0090305;GO:0004519;GO:0003676
TRINITY_DN396141_c3_g4_i3_m.3078747	leucine aminopeptidase 2	1.26	0.0066	GO:0006508;GO:0009507;GO:0004177;GO:0008235;GO:0030145
TRINITY_DN350158_c1_g1_i1_m.1343470	ABC transporter F family member 1	1.26	0.0243	GO:0009536;GO:0005524;GO:0016887
TRINITY_DN389163_c1_g1_i3_m.1231161	histone deacetylase 8	1.35	0.0098	GO:0016787
TRINITY_DN381625_c0_g2_i15_m.3290704	R1 protein	1.25	0.0214	GO:0006355;GO:0005634;GO:0016021;GO:0042393;GO:0008270
TRINITY_DN389472_c0_g1_i1_m.1811993	Peptide chain release factor 1	1.39	0.0352	GO:0006415;GO:0005737;GO:0043022;GO:0016149
TRINITY_DN390617_c1_g1_i10_m.1392908	papain-like cysteine proteinase	1.34	0.0199	GO:0051603;GO:0071805;GO:0042391;GO:0005615;GO:0005764;GO:0005887;GO:0005783;GO:0005249;GO:0004197
TRINITY_DN383339_c1_g1_i2_m.978717	sugar phosphatase YfbT-like	1.36	0.0134	GO:0008152;GO:0009536;GO:0016787
TRINITY_DN362523_c0_g1_i1_m.2022092	pheophorbide a oxygenase	1.75	0.0190	GO:0015996;GO:0010228;GO:0009816;GO:0016226;GO:0009965;GO:0009793;GO:0045893;GO:0048481;GO:0030154;GO:0008219;GO:0010027;GO:0055114;GO:0009941;GO:0009534;GO:0016021;GO:0051537;GO:0010277;GO:0016630;GO:0032441;GO:0046872
TRINITY_DN387853_c1_g1_i9_m.1638899	chlorophyll a/b-binding protein WCAB precursor	1.21	0.0081	GO:0018298;GO:0009768;GO:0009416;GO:0009535;GO:0009941;GO:0009522;GO:0016021;GO:0010287;GO:0016168;GO:0031409;GO:0046872
TRINITY_DN361246_c0_g1_i1_m.1858670	ruBisCO large subunit-binding protein subunit beta	1.23	0.0088	GO:0042026;GO:0009536;GO:0005524
TRINITY_DN365437_c4_g1_i3_m.1725817	bifunctional nitrilase/nitrile hydratase NIT4-like	1.20	0.0125	GO:0006807;GO:0016021;GO:0016810
TRINITY_DN381701_c0_g1_i1_m.3289476	alliin lyase	1.56	0.0209	GO:0016021;GO:0047654
TRINITY_DN376603_c1_g2_i16_m.3125948	Tropinone reductase-like protein	1.22	0.0166	GO:0055114;GO:0016021;GO:0005829;GO:0016491
TRINITY_DN394982_c1_g1_i3_m.2425838	methionine sulfoxide reductase MSRA4.1	1.26	0.0090	GO:0009651;GO:0006979;GO:0055114;GO:0030091;GO:0009507;GO:0008113
TRINITY_DN361544_c4_g1_i2_m.3005723	Iron-sulfur cluster assembly enzyme ISCU	1.33	0.0276	GO:0097428;GO:0006879;GO:0044571;GO:0005759;GO:0051539;GO:0051537;GO:0008198;GO:0036455
TRINITY_DN394104_c3_g1_i3_m.900462	Root phototropism protein 2	1.64	0.0228	GO:0009638;GO:0007165;GO:0005737;GO:0005634;GO:0004871
TRINITY_DN397920_c4_g1_i3_m.2061483	cytosolic glutathione reductase	1.23	0.0127	GO:0098869;GO:0022900;GO:0006749;GO:0045454;GO:0005737;GO:0050661;GO:0050660;GO:0004362
TRINITY_DN394186_c3_g1_i12_m.1899482	lysine histidine transporter 1-like	1.65	0.0445	GO:0003333;GO:0016021;GO:0009536;GO:0005886;GO:0015171
TRINITY_DN396226_c2_g4_i2_m.1711803	Mitogen activated protein kinase kinase 5-like	1.37	0.0179	GO:0023014;GO:0005739;GO:0005524;GO:0004702
TRINITY_DN321105_c0_g1_i1_m.2518459	UDP-glucose:sterol glucosyltransferase	1.37	0.0106	GO:0016125;GO:0048316;GO:0052696;GO:0009813;GO:0030259;GO:0043231;GO:0005886;GO:0051507
TRINITY_DN385603_c4_g1_i2_m.2340413	Disease resistance protein RPM1	1.30	0.0187	GO:0043531
TRINITY_DN385055_c0_g1_i1_m.2188669	Stromal 70 kDa heat shock-related protein	1.21	0.0148	GO:0006457;GO:0009536;GO:0051082;GO:0005524
TRINITY_DN324869_c0_g2_i1_m.1612319	Anthocyanin 5-O-glucosyltransferase	1.21	0.0317	GO:0009813;GO:0052696;GO:0043231;GO:0080043;GO:0080044
TRINITY_DN399772_c4_g1_i6_m.3141526	aldehyde oxidase 2	1.26	0.0206	GO:0009688;GO:0009851;GO:0009115;GO:0055114;GO:0005829;GO:0004031;GO:0050660;GO:0016614;GO:0051537;GO:0004854;GO:0005506;GO:0009055
TRINITY_DN394931_c1_g1_i2_m.2425637	Alanine aminotransferase 2	1.21	0.0155	GO:0009058;GO:0042853;GO:0030170;GO:0004021
TRINITY_DN395784_c2_g5_i1_m.1780725	lethal leaf spot1	1.88	0.0372	GO:0015996;GO:0010228;GO:0009816;GO:0016226;GO:0009965;GO:0009793;GO:0045893;GO:0048481;GO:0030154;GO:0008219;GO:0010027;GO:0055114;GO:0009941;GO:0009534;GO:0016021;GO:0051537;GO:0010277;GO:0016630;GO:0032441;GO:0046872
TRINITY_DN397056_c2_g2_i1_m.2503270	2,3-bisphosphoglycerate-independent phosphoglycerate mutase	1.25	0.0167	GO:0006007;GO:0006096;GO:0005737;GO:0004619;GO:0030145
TRINITY_DN399698_c7_g4_i8_m.3206930	probable pyruvate, phosphate dikinase regulatory protein	1.36	0.0100	GO:0006468;GO:0009507;GO:0004674;GO:0016776;GO:0005524

TRINITY_DN346344_c0_g2_i2_m.2947108	Nucleoside diphosphate kinase 1	1.49	0.0181	GO:0006228;GO:0006241;GO:0006183;GO:0006165;GO:0005622;GO:0004550;GO:0046872;GO:0005524
TRINITY_DN387233_c0_g1_i2_m.1307454	galactokinase	1.22	0.0228	GO:0046835;GO:0006012;GO:0005829;GO:0005524;GO:0004335
TRINITY_DN363721_c0_g1_i2_m.3274340	1,2-dihydroxy-3-keto-5-methylthiopentene dioxygenase 4	1.20	0.0114	GO:0019509;GO:0055114;GO:0005737;GO:0005634;GO:0010309;GO:0005506
TRINITY_DN396146_c2_g1_i1_m.3078262	AF467539_1 putative aldehyde dehydrogenase BIS1	1.23	0.0099	GO:0072593;GO:0010133;GO:0055114;GO:0005739;GO:0009507;GO:0004028;GO:0004029;GO:0050897;GO:0003842;GO:0008270
TRINITY_DN396565_c13_g1_i8_m.2308008	cysteine endopeptidase EP gamma	1.33	0.0177	GO:0051603;GO:0006955;GO:0005764;GO:0005615;GO:0004197
TRINITY_DN381698_c3_g1_i1_m.3290397	glutathione S-transferase zeta class	1.39	0.0209	GO:0006749;GO:0009072;GO:0005737;GO:0004364
TRINITY_DN399054_c2_g1_i9_m.2648666	Superoxide-generating NADPH oxidase heavy chain subunit C	1.28	0.0439	GO:0055114;GO:0016021;GO:0000293;GO:0005506
TRINITY_DN391876_c2_g1_i3_m.2605180	succinate-semialdehyde dehydrogenase	1.27	0.0133	GO:0072593;GO:0006081;GO:0006540;GO:0009450;GO:0009416;GO:0009408;GO:0055114;GO:0009570;GO:0005759;GO:0004030;GO:0051287;GO:0009013;GO:0004777;GO:0005507;GO:0004029
TRINITY_DN257560_c0_g1_i1_m.3979057	phosphoribosylformylglycinamidine synthase	1.20	0.0096	GO:0006541;GO:0006189;GO:0009536;GO:0004642;GO:0016740;GO:0005524
TRINITY_DN380935_c0_g2_i11_m.936991	phytochrome-associated serine/threonine-protein phosphatase 1	1.22	0.0457	GO:0006470;GO:0004721
TRINITY_DN362367_c0_g1_i10_m.1737261	universal stress protein 23267	1.35	0.0156	GO:0006950;GO:0002238
TRINITY_DN373443_c2_g2_i5_m.1235535	cinnamyl alcohol dehydrogenase	1.28	0.0253	GO:0009809;GO:0055114;GO:0045551;GO:0052747;GO:0008270
TRINITY_DN391288_c3_g1_i1_m.2774086	UDP-glycosyltransferase 73C6-like	1.52	0.0157	GO:0009813;GO:0052696;GO:0043231;GO:0080043;GO:0080044
TRINITY_DN399495_c11_g1_i1_m.1285746	isovaleryl-CoA dehydrogenase	1.34	0.0309	GO:0033539;GO:0006552;GO:0055088;GO:0005739;GO:0050660;GO:0052890;GO:0000062;GO:0008470;GO:0009055
TRINITY_DN368481_c0_g1_i6_m.1420042	probable plastid-lipid-associated protein 11	1.31	0.0303	GO:0009536
TRINITY_DN385558_c0_g2_i12_m.2340628	Flavin-containing monooxygenase FMO GS-OX-like 5	1.24	0.0263	GO:0022900;GO:0009536;GO:0050661;GO:0050660;GO:0004499;GO:0015036
TRINITY_DN385766_c1_g2_i3_m.1145477	Serine/threonine-protein kinase HT1	1.26	0.0374	GO:0006468;GO:0004674;GO:0005524
TRINITY_DN382294_c1_g2_i5_m.1497186	glyceraldehyde-3-phosphate dehydrogenase 2	1.24	0.0159	GO:0006006;GO:0006096;GO:0055114;GO:0005737;GO:0004365;GO:0051287;GO:0050661
TRINITY_DN283360_c0_g1_i2_m.3925980	phosphoenolpyruvate carboxykinase [ATP]-like	1.32	0.0488	GO:0006094;GO:0004612;GO:0005524
TRINITY_DN392732_c1_g6_i6_m.904894	ubiquinol oxidase 2	1.26	0.0339	GO:0055114;GO:0016021;GO:0005739;GO:0070469;GO:0009916;GO:0046872
TRINITY_DN359265_c1_g1_i21_m.1983442	12-oxophytodienoate reductase 2	1.30	0.0413	GO:0031408;GO:0055114;GO:0005739;GO:0003959;GO:0010181
TRINITY_DN399606_c4_g1_i2_m.3205972	lysine ketoglutarate reductase/saccharopine dehydrogenase	1.47	0.0322	GO:0055114;GO:0016021;GO:0016491
TRINITY_DN364278_c0_g1_i12_m.2512544	Ras-related protein RABD1	1.24	0.0424	GO:0006888;GO:0007264;GO:0005789;GO:0032588;GO:0005886;GO:0080115;GO:0030742;GO:0005525
TRINITY_DN399344_c6_g1_i1_m.1325958	Pyruvate, phosphate dikinase 1	1.29	0.0275	GO:0019252;GO:0006090;GO:0016310;GO:0009416;GO:0015979;GO:0008610;GO:0009507;GO:0005829;GO:0005634;GO:0016301;GO:0050242;GO:0046872;GO:0005524
TRINITY_DN391236_c2_g1_i2_m.2773175	Secologanin synthase	1.29	0.0359	GO:0055114;GO:0016021;GO:0004497;GO:0020037;GO:0016705;GO:0003677;GO:0005506
TRINITY_DN387677_c1_g1_i2_m.3106465	Dolichyldiphosphatase 1	1.40	0.0241	GO:0006651;GO:0006487;GO:0048868;GO:0016311;GO:0030176;GO:0009507;GO:0047874;GO:0008195
TRINITY_DN392537_c5_g2_i3_m.1492788	cysteine proteinase 1	1.50	0.0295	GO:0051603;GO:0005764;GO:0005615;GO:0004197
TRINITY_DN398577_c3_g1_i3_m.1121670	glutathionyl-hydroquinone reductase YqjG isoform X2	1.26	0.0242	GO:0016740
TRINITY_DN941973_c0_g1_i1_m.370801	phosphoglycerate mutase-like protein 1	1.22	0.0367	GO:0009507
TRINITY_DN379353_c0_g1_i3_m.1904644	magnesium transporter MRS2-A	1.29	0.0267	GO:1903830;GO:0015693;GO:0031969;GO:0016021;GO:0015095
TRINITY_DN398238_c1_g1_i8_m.1378798	protein CHUP1	1.35	0.0328	GO:0010207;GO:0045893;GO:0009902;GO:0034660;GO:0010027;GO:0035304;GO:0042793;GO:0009707
TRINITY_DN771345_c0_g1_i1_m.4363218	Putative ornithine aminotransferase	1.38	0.0321	GO:0006635;GO:0006593;GO:0009413;GO:0009414;GO:0055129;GO:0042538;GO:0009733;GO:

				0009737;GO:0009753;GO:0019544;GO:0010121;GO:0007031;GO:0051646;GO:0009408;GO:0006979;GO:0010260;GO:0009741;GO:0005759;GO:0030170;GO:0042802;GO:0004587;GO:0008270
TRINITY_DN379563_c5_g2_i4_m.2399372	Anthocyanidin 5,3-O-glucosyltransferase	1.38	0.0248	GO:0009813;GO:0052696;GO:0043231;GO:0080043;GO:0080044
TRINITY_DN387440_c0_g1_i5_m.2381371	probable serine protease EDA2	1.25	0.0317	GO:0006508;GO:0008239;GO:0004185
TRINITY_DN371754_c0_g2_i1_m.2598765	probable peroxygenase 5	1.51	0.0214	GO:0005509
TRINITY_DN394962_c1_g3_i3_m.2427971	Lipoxygenase 2.3	1.23	0.0317	GO:0031408;GO:0055114;GO:0009507;GO:0046872;GO:0016165
TRINITY_DN369194_c2_g1_i1_m.1548460	ATP synthase subunit beta	1.24	0.0287	GO:0015991;GO:0015986;GO:0045261;GO:0005524;GO:0046933
TRINITY_DN369263_c0_g2_i1_m.2472935	BURP domain-containing protein 3-like	1.31	0.0253	GO:0005802;GO:0005768
TRINITY_DN395659_c0_g2_i4_m.2554194	uncharacterized aarF domain-containing protein kinase At4g31390	1.29	0.0287	GO:0015996;GO:0006468;GO:0080183;GO:0010287;GO:0005739;GO:0004672;GO:0005524
TRINITY_DN385040_c0_g2_i1_m.2192508	putative protein phosphatase 2C 44	1.35	0.0388	GO:0006470;GO:0009536;GO:0046872;GO:0004722
TRINITY_DN376620_c0_g1_i1_m.1942519	vesicle-associated membrane protein 714	1.25	0.0364	GO:0006906;GO:0006887;GO:0009651;GO:0005794;GO:0016021;GO:0005773;GO:0031201;GO:0000149;GO:0005484
TRINITY_DN390832_c1_g4_i1_m.2289016	ubiquinone biosynthesis protein COQ9	1.32	0.0257	GO:0006744;GO:0005743;GO:0008289
TRINITY_DN382365_c1_g1_i5_m.2695644	universal stress protein A-like protein	1.24	0.0327	GO:0006950
TRINITY_DN387302_c0_g1_i3_m.1304699	probable aspartyl aminopeptidase	1.32	0.0265	GO:0006508;GO:0009570;GO:0005739;GO:0004177;GO:0008270;GO:0008237
TRINITY_DN395919_c0_g1_i3_m.2146613	multiple organellar RNA editing factor 2	1.33	0.0274	GO:0009536
TRINITY_DN391067_c0_g2_i12_m.3320632	Alcohol dehydrogenase 3	1.26	0.0357	GO:0055114;GO:0005737;GO:0004022;GO:0008270
TRINITY_DN374423_c0_g2_i12_m.1575516	AF442967_1 thaumatin-like protein	11.78	0.0380	------
TRINITY_DN374423_c0_g2_i4_m.1575469	permatin precursor	5.18	0.0207	------
TRINITY_DN374423_c0_g1_i3_m.1575457	permatin	1.75	0.0391	------
TRINITY_DN397238_c1_g1_i3_m.1024939	Hsp17.2	1.86	0.0025	------
TRINITY_DN381705_c2_g1_i2_m.3288861	jasmonate-induced protein homolog	1.98	0.0022	------
TRINITY_DN391566_c2_g2_i6_m.2837883	Tropinone reductase-like protein	1.47	0.0103	------
TRINITY_DN309613_c0_g1_i1_m.870427	aspartic proteinase nepenthesin-1-like	1.23	0.0132	------
		**Down-regulation**		
TRINITY_DN398835_c2_g4_i6_m.1561624	ribosomal protein S7	0.71	0.0461	GO:0000028;GO:0006412;GO:0015935;GO:0005739;GO:0009507;GO:0019843;GO:0003735;GO:0003729
TRINITY_DN395707_c1_g4_i1_m.1780210	Heat shock cognate 70 kDa protein 1	0.59	0.0483	GO:0016021;GO:0005524;GO:0003677
TRINITY_DN353955_c0_g2_i1_m.2165457	protein FLUORESCENT IN BLUE LIGHT	0.68	0.0441	GO:0016021;GO:0009536
TRINITY_DN396987_c2_g1_i2_m.2674707	probable beta-D-xylosidase 7	0.55	0.0390	GO:0031222;GO:0045493;GO:0016021;GO:0009505;GO:0046556;GO:0009044
TRINITY_DN349285_c0_g1_i2_m.1912909	beta-amylase 1	0.74	0.0388	GO:0008152;GO:0009536;GO:0016787
TRINITY_DN392690_c0_g2_i4_m.2117944	MAR-binding filament-like protein 1 isoform X1	0.73	0.0425	GO:0016021;GO:0009536
TRINITY_DN353380_c0_g1_i1_m.1509948	50S ribosomal protein L10	0.73	0.0444	GO:0042254;GO:0006412;GO:0009536;GO:0005840;GO:0003735;GO:0003676
TRINITY_DN366839_c2_g2_i1_m.2771195	40S ribosomal protein S15	0.74	0.0382	GO:0000028;GO:0006412;GO:0022627;GO:0003735;GO:0003723
TRINITY_DN340121_c0_g1_i1_m.2989461	50S ribosomal protein L21	0.72	0.0402	GO:0010207;GO:0009902;GO:0035304;GO:0009793;GO:0045893;GO:0010027;GO:0042793;GO:0006412;GO:0042742;GO:0009941;GO:0009570;GO:0005634;GO:0005762;GO:0003735;GO:0003723
TRINITY_DN399579_c0_g2_i3_m.3002181	rps8 gene product--	0.74	0.0484	GO:0006412;GO:0005840;GO:0009507;GO:0003735;GO:0019843
TRINITY_DN398747_c3_g2_i3_m.2001822	ribosomal protein L33	0.65	0.0477	GO:0006412;GO:0005840;GO:0009507;GO:0003735
TRINITY_DN371947_c0_g1_i3_m.2918547	single-stranded DNA-binding protein WHY1	0.70	0.0419	GO:0006281;GO:0006355;GO:0045910;GO:0009570;GO:0005634;GO:0003723;GO:0003677
TRINITY_DN247605_c0_g1_i1_m.3852356	glutathione S-transferase F11-like	0.75	0.0303	GO:0006749;GO:0005737;GO:0016021;GO:0004364
TRINITY_DN358470_c0_g5_i1_m.3334320	ribosomal protein S4	0.71	0.0500	GO:0045903;GO:0009507;GO:0015935;GO:0003735;GO:0019843

TRINITY_DN397087_c2_g1_i1_m.2508655	ribosomal protein S2	0.70	0.0484	GO:0006351;GO:0006412;GO:0015935;GO:0009507;GO:0003735;GO:0003677;GO:0003899
TRINITY_DN225147_c0_g1_i1_m.4155574	translation elongation factor 1 alpha--	0.76	0.0487	GO:0006468;GO:0006414;GO:0005737;GO:0003924;GO:0004672;GO:0003746;GO:0005524;GO:0005525
TRINITY_DN387620_c1_g2_i2_m.3106275	geranylgeranyl hydrogenase	0.69	0.0308	GO:0015995;GO:0055114;GO:0015979;GO:0010189;GO:0009507;GO:0016628;GO:0045550;GO:0071949
TRINITY_DN390544_c2_g1_i10_m.1823510	bifunctional epoxide hydrolase 2-like	0.73	0.0257	GO:0016787
TRINITY_DN370090_c0_g1_i2_m.2932503	2-hydroxyisoflavanone dehydratase	0.74	0.0275	GO:0008152;GO:0016787
TRINITY_DN383617_c0_g1_i3_m.3254791	LL-diaminopimelate aminotransferase	0.70	0.0225	GO:0009735;GO:0009862;GO:0009089;GO:0009570;GO:0010285;GO:0030170;GO:0005507
TRINITY_DN381300_c1_g1_i4_m.1253627	shikimate kinase family protein	0.76	0.0186	GO:0009965;GO:0006636;GO:0016310;GO:0030154;GO:0009658;GO:0019288;GO:0009108;GO:0009106;GO:0010027;GO:0015995;GO:0006546;GO:0006733;GO:0006766;GO:0019748;GO:0016226;GO:0009117;GO:0045893;GO:0009684;GO:0009073;GO:0019344;GO:0019632;GO:0009570;GO:0000287;GO:0004765
TRINITY_DN376734_c1_g1_i4_m.2860427	50S ribosomal protein L17	0.70	0.0335	GO:0019288;GO:0006412;GO:0009941;GO:0015934;GO:0009570;GO:0003735
TRINITY_DN392343_c1_g1_i5_m.2440572	Medium-chain-fatty-acid--CoA ligase	0.82	0.0347	GO:0008152;GO:0016874
TRINITY_DN372921_c1_g1_i6_m.1992436	Alkaline and neutral invertase	0.81	0.0384	GO:0010311;GO:0009555;GO:0080022;GO:0005987;GO:0048510;GO:0005829;GO:0004575;GO:0033926
TRINITY_DN391565_c1_g2_i6_m.2834650	ribosome-recycling factor	0.80	0.0381	GO:0002184;GO:0009658;GO:0009793;GO:0009817;GO:0032544;GO:0009579;GO:0009570;GO:0005829;GO:0043023;GO:0005507
TRINITY_DN362747_c0_g1_i6_m.2588137		0.74	0.0303	GO:0006412;GO:0009536;GO:0022625;GO:0016021;GO:0003735;GO:0003729
TRINITY_DN388927_c0_g1_i8_m.1608535	imidazoleglycerol-phosphate dehydratase	0.75	0.0230	GO:0000105;GO:0009536;GO:0004424
TRINITY_DN387042_c0_g3_i1_m.1413379	RNA recognition motif	0.80	0.0005	GO:0015995;GO:0000381;GO:0019288;GO:0009902;GO:0034660;GO:0010027;GO:0015979;GO:0042793;GO:0009579;GO:0009570;GO:0005634;GO:0019013;GO:0030529;GO:0000166;GO:0003729
TRINITY_DN366807_c0_g1_i14_m.2771165	Caleosin related protein	0.81	0.0362	GO:0016021;GO:0005509
TRINITY_DN392261_c2_g1_i19_m.1911164	Pyridoxamine 5'-phosphate oxidase	0.81	0.0352	GO:0055114;GO:0009536;GO:0010181;GO:0016491
TRINITY_DN395637_c0_g1_i3_m.2556763	tRNA synthetases class	0.82	0.0321	GO:0006450;GO:0006438;GO:0005829;GO:0005739;GO:0002161;GO:0004832;GO:0005524
TRINITY_DN391693_c1_g1_i9_m.3210734	Coproporphyrinogen III oxidase	0.57	0.0449	GO:0015995;GO:0006782;GO:0055114;GO:0016020;GO:0009507;GO:0004109;GO:0042803
TRINITY_DN372678_c0_g1_i9_m.2026810	Ribosomal protein L9	0.70	0.0473	GO:0006412;GO:0005840;GO:0005739;GO:0044435;GO:0009507;GO:0019843;GO:0003735
TRINITY_DN388617_c1_g2_i6_m.1587296	30S ribosomal protein S1	0.74	0.0342	GO:0015995;GO:0019288;GO:0009902;GO:0034660;GO:0010027;GO:0009773;GO:0009579;GO:0005840;GO:0016020;GO:0009507;GO:0003676
TRINITY_DN396144_c1_g2_i16_m.3082637	Metallopeptidase family M24	0.80	0.0152	GO:0009737;GO:0070084;GO:0006508;GO:0005739;GO:0009507;GO:0070006;GO:0046872
TRINITY_DN373286_c0_g1_i11_m.893566	chalcone isomerase I	0.76	0.0155	GO:0009813;GO:0045430
TRINITY_DN393413_c0_g1_i2_m.1687516	ribosomal protein L16	0.79	0.0287	GO:0032543;GO:0009507;GO:0005762;GO:0003735;GO:0019843
TRINITY_DN374787_c7_g3_i1_m.3186401	FAD binding domain;Glucose inhibited division protein A	0.52	0.0193	GO:0015995;GO:0055114;GO:0015979;GO:0010189;GO:0009507;GO:0016628;GO:0045550;GO:0071949
TRINITY_DN391565_c1_g2_i5_m.2834648	Ribosome recycling factor	0.79	0.0143	GO:0002184;GO:0009658;GO:0009793;GO:0009817;GO:0032544;GO:0009579;GO:0009570;GO:0005829;GO:0043023;GO:0005507
TRINITY_DN384752_c1_g1_i6_m.1760471	putative FIDDLEHEAD very long chain fatty acid condensing enzyme FDH;1	0.82	0.0103	GO:0006633;GO:0016021;GO:0005783;GO:0016747
TRINITY_DN391302_c2_g1_i1_m.2774990	SnRK1 gamma subunit	0.71	0.0207	GO:0016310;GO:0016301
TRINITY_DN391735_c1_g2_i2_m.2158020	Mg-por_mtran_C;Methyltransf_25	0.66	0.0202	GO:0015995;GO:0032259;GO:0009536;GO:0046406
TRINITY_DN388935_c4_g1_i1_m.1610391	Peroxidase 52	0.77	0.0174	GO:0098869;GO:0042744;GO:0006979;GO:0055114;GO:0005576;GO:0020037;GO:0004601;GO:0046872
TRINITY_DN353830_c6_g1_i1_m.1356770	Actin-3	0.79	0.0078	GO:0005856;GO:0005737;GO:0005524

TRINITY_DN398647_c0_g2_i2_m.2654559	divinyl chlorophyllide a 8-vinyl-reductase	0.67	0.0147	GO:0015995;GO:0055114;GO:0009941;GO:0009534;GO:0016020;GO:0033728;GO:0051744
TRINITY_DN393592_c1_g1_i4_m.2756609	glutamyl-tRN	0.76	0.0383	GO:0006655;GO:0070681;GO:0032543;GO:0030956;GO:0009570;GO:0005739;GO:0050567;GO:0016740;GO:0005524
TRINITY_DN382119_c3_g1_i9_m.1369400	protein STRICTOSIDINE SYNTHASE-LIKE 3-like	0.81	0.0226	GO:0009058;GO:0005783;GO:0016021;GO:0005739;GO:0016844;GO:0016788
TRINITY_DN160871_c0_g1_i1_m.519090	Major intrinsic protein	0.68	0.0384	GO:0006833;GO:0016021;GO:0005215
TRINITY_DN396624_c1_g1_i7_m.2176982	alanine--glyoxylate aminotransferase 2 homolog 1	0.74	0.0104	GO:0019544;GO:0005739;GO:0005774;GO:0030170;GO:0008453;GO:0042802;GO:0008270
TRINITY_DN357753_c0_g1_i2_m.2214680	protein YLS3	0.68	0.0476	GO:0006869;GO:0016021;GO:0008289
TRINITY_DN370281_c0_g3_i3_m.2482723	RNA polymerase beta subunit	0.73	0.0087	GO:0006351;GO:0009507;GO:0003899;GO:0032549;GO:0003677
TRINITY_DN381495_c0_g1_i7_m.1250579	superoxide dismutase [Fe] 1, chloroplastic-like	0.82	0.0049	GO:0019430;GO:0055114;GO:0009536;GO:0046872;GO:0004784
TRINITY_DN379241_c1_g1_i2_m.2131457	Ribosomal protein L1p/L10e family	0.78	0.0261	GO:0000470;GO:0006412;GO:0009536;GO:0022625;GO:0005739;GO:0003735;GO:0003723
TRINITY_DN397154_c1_g1_i9_m.3279147	ribonuclease J isoform X1	0.83	0.0176	GO:0044550;GO:0045333;GO:0015979;GO:0016020;GO:0009507;GO:0020037;GO:0016709;GO:0003723;GO:0003677;GO:0005506
TRINITY_DN321755_c0_g1_i1_m.3147984	proline-rich receptor-like protein kinase PERK2	0.77	0.0486	GO:0016020
TRINITY_DN396406_c1_g2_i2_m.2062379	Alpha-L-arabinofuranosidase 1	0.80	0.0195	GO:0006810;GO:0046373;GO:0016021;GO:0005215;GO:0046556
TRINITY_DN398652_c1_g1_i6_m.2659113	mitochondrial acid phosphatase	0.78	0.0141	GO:0055062;GO:0016311;GO:0005773;GO:0005829;GO:0009505;GO:0046872;GO:0003993
TRINITY_DN375609_c0_g1_i6_m.1955928	protoporphyrinogen oxidase, chloroplastic	0.80	0.0043	GO:0015995;GO:0006782;GO:0055114;GO:0009941;GO:0009534;GO:0004729
TRINITY_DN391571_c4_g1_i4_m.2838529	Sodium/calcium exchanger protein;EF-hand domain pair	0.78	0.0053	GO:0055074;GO:0035725;GO:0016021;GO:0005432;GO:0005509
TRINITY_DN744194_c0_g1_i1_m.4160115	branched-chain-amino-acid aminotransferase 5, chloroplastic	0.82	0.0012	GO:0009081;GO:0052654;GO:0052655;GO:0052656
TRINITY_DN369498_c0_g1_i2_m.1468569	protein CHLORORESPIRATORY REDUCTION 6-	0.82	0.0138	GO:0016226;GO:0010275;GO:0009535;GO:0009570;GO:0016021;GO:0005739
TRINITY_DN398547_c2_g2_i3_m.1124976	Leucyl-tRNA synthetase, cytoplasmic	0.75	0.0129	GO:0006450;GO:0006429;GO:0005829;GO:0016021;GO:0004823;GO:0002161;GO:0005524
TRINITY_DN263154_c0_g1_i2_m.4123170	cytochrome f	0.82	0.0017	GO:0015979;GO:0055114;GO:0009535;GO:0031361;GO:0020037;GO:0005506;GO:0009055
TRINITY_DN398480_c8_g3_i1_m.2873648	MYB-related protein	0.80	0.0007	GO:0003677
TRINITY_DN388014_c2_g1_i3_m.2951173	Chlorophyll a-b binding protein 1B-20	0.36	0.0080	GO:0018298;GO:0009768;GO:0019344;GO:0009416;GO:0070838;GO:0009535;GO:0009941;GO:0009522;GO:0016021;GO:0010287;GO:0016168;GO:0031409;GO:0046872
TRINITY_DN397730_c2_g1_i4_m.2923650	starch synthase 1, chloroplastic/amyloplastic	0.76	0.0179	GO:0010021;GO:0019252;GO:0009570;GO:0009501;GO:0033201;GO:0004373;GO:0009011
TRINITY_DN375098_c4_g1_i14_m.1929460	preprotein translocase subunit SECY	0.76	0.0277	GO:0015031;GO:0009535;GO:0016021
TRINITY_DN63723_c0_g1_i1_m.4541991	ribosome maturation protein SBDS	0.82	0.0119	GO:0006364;GO:0042256;GO:0005737;GO:0005634;GO:0003723
TRINITY_DN339270_c1_g1_i1_m.2285136	ankyrin repeat domain-containing protein 2A	0.78	0.0261	GO:0009536
TRINITY_DN332222_c2_g1_i2_m.1685807	patellin-3-like	0.52	0.0333	GO:0006810;GO:0016021
TRINITY_DN389835_c5_g1_i3_m.2490719	cold shock protein-1	0.65	0.0171	GO:0006355;GO:0008270;GO:0003677
TRINITY_DN399089_c2_g1_i7_m.2653761	calcium-dependent protein kinase	0.80	0.0229	GO:0018105;GO:0009738;GO:0035556;GO:0046777;GO:0005737;GO:0016020;GO:0005634;GO:0004683;GO:0009931;GO:0005509;GO:0005524;GO:0005516
TRINITY_DN386963_c0_g1_i1_m.2664437	Nuclear-interacting partner of ALK	0.71	0.0138	GO:0016021;GO:0005634;GO:0008270
TRINITY_DN384416_c4_g2_i6_m.1581026	14-3-3-like protein GF14-B	0.75	0.0355	GO:0005737;GO:0005634;GO:0019904
TRINITY_DN386382_c0_g3_i6_m.1129079	putative 20S proteasome subunit beta-4	0.70	0.0437	GO:0000502;GO:0016021;GO:0009536
TRINITY_DN397531_c0_g1_i5_m.2295465	Eukaryotic translation initiation factor 3 subunit D	0.79	0.0126	GO:0001731;GO:0006446;GO:0005852;GO:0016282;GO:0016021;GO:0033290;GO:0003743
TRINITY_DN383309_c0_g3_i3_m.980047	Ubiquitin carrier protein E2 27	0.79	0.0069	GO:0043161;GO:0016579;GO:0000209;GO:0005737;GO:0005634;GO:0031625;GO:0016874;GO:0061630;GO:0005524
TRINITY_DN386745_c0_g1_i12_m.2816100	11-beta-hydroxysteroid dehydrogenase 1B-like	0.71	0.0004	GO:0055114;GO:0016021;GO:0016491
TRINITY_DN392907_c0_g1_i3_m.2522557	2-phytyl-1,4-beta-naphthoquinone methyltransferase	0.69	0.0137	GO:0042372;GO:0032259;GO:0009507;GO:0008757;GO:0052624
TRINITY_DN353542_c1_g1_i4_m.3110530	ATP synthase alpha/beta family	0.76	0.0352	GO:0015991;GO:0015986;GO:0005754;GO:0005524;GO:0046933
TRINITY_DN377763_c0_g1_i2_m.2644043	Glyoxalase/Bleomycin resistance protein/Dioxygenase superfamily	0.80	0.0002	GO:0018105;GO:0006897;GO:0016055;GO:0008360;GO:0005634;GO:0005739;GO:0004674;GO:0004462;GO:0046872;GO:0005524

TRINITY_DN380425_c1_g1_i2_m.1295559	Putative glucose-6-phosphate 1-epimerase	0.74	0.0010	GO:0005975;GO:0048046;GO:0009570;GO:0030246;GO:0016853
TRINITY_DN392712_c0_g1_i1_m.903001	plastidic alpha 1,4-glucan phosphorylase	0.81	0.0238	GO:0005980;GO:0042742;GO:0009414;GO:0009409;GO:0019252;GO:0009570;GO:0030170;GO:0008184
TRINITY_DN395713_c3_g2_i4_m.1786010	glycine-rich RNA-binding protein GRP2A	0.54	0.0008	GO:0045087;GO:0000380;GO:0009735;GO:0010119;GO:0006406;GO:0010228;GO:0006970;GO:0010501;GO:0032508;GO:0005829;GO:0005777;GO:0005634;GO:0048046;GO:0009507;GO:0009506;GO:0003697;GO:0000166;GO:0003690;GO:0003729
TRINITY_DN398888_c3_g1_i6_m.1558931	NPH1-2	0.61	0.0000	GO:0046777;GO:0023014;GO:0009638;GO:0000160;GO:0009904;GO:0009903;GO:0018298;GO:0010119;GO:0009785;GO:0010362;GO:0010155;GO:0006355;GO:0009898;GO:0005773;GO:0005634;GO:0009986;GO:0004674;GO:0010181;GO:0005524;GO:0009882;GO:0000155;GO:0042802
TRINITY_DN399631_c1_g3_i1_m.3206417	light-induced protein 1-like	0.66	0.0158	GO:0005622
TRINITY_DN386076_c0_g2_i4_m.2971427	Sigma 54 modulation/S30EA ribosomal protein C terminus	0.64	0.0050	GO:0044238;GO:0009570;GO:0005840;GO:0009534
TRINITY_DN398836_c4_g1_i4_m.1560471	DNA topoisomerase 1	0.82	0.0005	GO:0006265;GO:0005694;GO:0003917;GO:0003677;GO:0004672
TRINITY_DN386076_c0_g2_i3_m.2971417	Sigma 54 modulation/S30EA ribosomal protein C terminus	0.70	0.0000	GO:0044238;GO:0009536;GO:0005840
TRINITY_DN399200_c1_g1_i1_m.911796	glutamine-dependent asparagine synthetase	0.30	0.0054	GO:0006529;GO:0006541;GO:0009063;GO:0009646;GO:0005829;GO:0004066;GO:0042803
TRINITY_DN399457_c6_g4_i1_m.1283861	30S ribosomal protein 2	0.74	0.0291	GO:0019288;GO:0009773;GO:0009579;GO:0009941;GO:0005840;GO:0000166;GO:0003676
TRINITY_DN387535_c2_g2_i1_m.2259200	3-dehydroquinate synthase;Iron-containing alcohol dehydrogenase	0.79	0.0412	GO:0033587;GO:0009423;GO:0009073;GO:0009507;GO:0051287;GO:0003856;GO:0046872;GO:0042802
TRINITY_DN389718_c2_g1_i7_m.997534	MOSC domain-containing protein 2	0.71	0.0141	GO:0003824;GO:0030170;GO:0030151
TRINITY_DN359020_c2_g1_i1_m.2563015	ubiquitin	0.73	0.0107	GO:0006412;GO:0005794;GO:0022627;GO:0005634;GO:0003735;GO:0046872
TRINITY_DN396119_c3_g1_i25_m.3077009	Asparagine synthase;Glutamine amidotransferase domain	0.50	0.0001	GO:0070981;GO:0006541;GO:0009646;GO:0005829;GO:0005524;GO:0004066;GO:0042803
TRINITY_DN386076_c0_g3_i6_m.2971431	Ribosomal_S30AE Description:Sigma 54 modulation protein / S30EA ribosomal protein	0.67	0.0022	GO:0044238;GO:0009536;GO:0005840
TRINITY_DN369697_c2_g1_i2_m.3026824	Lipoxygenase;PLAT/LH2 domain	0.72	0.0058	GO:0031408;GO:0055114;GO:0009507;GO:0046872;GO:0016165
TRINITY_DN397069_c0_g1_i15_m.2503948	Putative ribose-5-phosphate isomerase	0.69	0.0295	GO:0009735;GO:0009052;GO:0009535;GO:0009941;GO:0009570;GO:0004751
TRINITY_DN376554_c1_g1_i1_m.3127678	haloacid dehalogenase-like hydrolase domain-containing protein 3	0.81	0.0368	GO:0008152;GO:0016787
TRINITY_DN343760_c0_g1_i1_m.1177101	Globin	0.80	0.0403	GO:0019432;GO:0006631;GO:0046872;GO:0019825;GO:0020037
TRINITY_DN391257_c1_g1_i19_m.2777977	plasminogen activator inhibitor 1 RNA-binding protein-like	0.77	0.0332	------
TRINITY_DN855066_c0_g1_i1_m.3466289	cold responsive protein	0.50	0.0004	------
TRINITY_DN393114_c3_g1_i9_m.1553398	group3 late embryogenesis abundant protein	0.76	0.0292	------
TRINITY_DN565404_c0_g1_i1_m.3657538	Remorin	0.77	0.0450	------
TRINITY_DN392479_c1_g1_i5_m.1259193	protein EARLY RESPONSIVE TO DEHYDRATION 15-like isoform X1	0.47	0.0035	------
TRINITY_DN388490_c0_g1_i4_m.2462399	Rhodanese-like domain	0.64	0.0023	------

DEPs: Differentially expression proteins, CK:control, AS:alkali stress(35mmol.L^{-1} $NaCO_3$:$NaHCO_3$=1:1).
