# Supplementary material for: Proteomic Responses to Alkali Stress in Oats and the Alleviatory Effects of Exogenous Spermine Application
Source: Front Plant Sci. 2021 Apr 1;12:627129. doi: 10.3389/fpls.2021.627129 (PMC8049610; doi:10.3389/fpls.2021.627129)
Supplement: Supplementary file 11 [file Table_3.pdf]

SUPPLEMENTAL TABLE 3 Some differentially expressed proteins related to alkali tolerance in leaves at AS vs Ck

Protein ID	Description	Up/ down	Unique peptides number	Protein coverage (%)	Peptide sequences
**Chloroplast structure**					
TRINITY_DN283360_c0_g1_i2_m.3925980	phosphoenolpyruvate carboxykinase [ATP]-like	up	3	6	[K].FGTVLENVVFDEHTR.[E]
TRINITY_DN391562_c1_g1_i4_m.2835564	ruBisCO large subunit-binding protein subunit beta	up	1	16	[RK].IVNDGVTVAK.[E]
TRINITY_DN397107_c2_g1_i1_m.3281948	outer envelope protein 80	up	1	9	[-].HLDQVIK.[S]
TRINITY_DN84857_c0_g1_i1_m.4366601	AF251264_1 ribulose bisphosphate carboxylase activase B	up	4	70	[R].KYDFDNTMDGLYIAPAFMDK.[L]
TRINITY_DN387165_c0_g1_i11_m.1009884	NADH dehydrogenase [ubiquinone] 1 alpha subcomplex assembly factor 3	up	2	9	[K].TFADITAESLSIFK.[V]
TRINITY_DN355749_c0_g1_i6_m.1741158	ATP-dependent zinc metalloprotease FTSH 8	up	2	8	[K].GALLVGPPGTGK.[T]
TRINITY_DN383994_c1_g1_i5_m.2788659	outer envelope pore protein 37	up	5	19	[K].VPQDNIQNSTVVFR.[V]
TRINITY_DN96990_c0_g1_i1_m.4405674	NADP-dependent glyceraldehyde-3-phosphate dehydrogenase	up	1	20	[K].GATFCQEYR.[R]
TRINITY_DN396075_c2_g1_i2_m.1774117	NADH-plastoquinone oxidoreductase subunit 1	up	1	5	[R].MDQLLNLGWK.[F]
TRINITY_DN382354_c0_g1_i1_m.2699976	fructokinase-1	up	4	32	[R].ENGVDDSAVVFDAGAR.[T]
TRINITY_DN396838_c12_g1_i2_m.2571383	Rubisco activase beta form precursor	up	1	37	[K].GIVDSLFQAPMGDGTHEAVLSSYEYISQGLR.[K]
TRINITY_DN387853_c1_g1_i9_m.1638899	chlorophyll a/b-binding protein WCAB precursor	up	1	83	[K].AGSQIFSEGGLDYLGNPSLVHAQSILAIWACQVVLMGAVEGYR.[VI-]
TRINITY_DN361246_c0_g1_i1_m.1858670	ruBisCO large subunit-binding protein subunit beta	up	4	21	[K].SQYLDDIATLTGGTVIR.[E]
**Antioxidant system**					
TRINITY_DN381698_c3_g1_i1_m.3290397	glutathione S-transferase zeta class	up	6	32	[K].VGTGESLPWIQQQIDR.[G]
TRINITY_DN395784_c2_g5_i1_m.1780725	lethal leaf spot1	up	2	45	[K].ESSADVNQQYTK.[L]
TRINITY_DN393731_c4_g1_i7_m.3182505	Catalase isozyme 2	up	18	60	[R].EGNWDLLGNNFPVFFIR.[D]
TRINITY_DN370378_c1_g1_i6_m.2678716	peptide methionine sulfoxide reductase B1	up	4	20	[K].VDNASLSDEELK.[K]
TRINITY_DN374912_c0_g1_i8_m.931306	Catalase-1	up	1	52	[K].YRPSSSFNAPMWSTNSGAPVWNNDNSLTVGSR.[G]
TRINITY_DN388147_c2_g1_i9_m.1808976	Monodehydroascorbate reductase	up	3	60	[K].GIELILSTEIVK.[A]
TRINITY_DN398089_c1_g1_i4_m.947850	Gamma-glutamyltranspeptidase 1	up	1	6	[R].AGGVVTADDMR.[G]
TRINITY_DN384601_c2_g1_i12_m.2355839	L-ascorbate peroxidase 1	up	5	39	[K].FDNTYFTELLSGDKEGLLQLPSDK.[T]

Continue supplemental table 3

Protein ID	Description	Up/down	Unique peptides number	Protein coverage (%)	Peptide sequences
TRINITY_DN383094_c0_g2_i9_m.2882453	putative 5'-adenylylsulfate reductase 1	up	5	17	[K].GLFSFYEDGHQECCR.[V]
TRINITY_DN355884_c0_g1_i6_m.3318033	probable L-ascorbate peroxidase 7	up	2	31	[K].YAADQEAFFEDYAEAHAK.[L]
TRINITY_DN202479_c1_g1_i1_m.3959788	catalase	up	1	13	[R].WIDALSDPR.[L]
TRINITY_DN369493_c1_g1_i1_m.1470675	probable phospholipid hydroperoxide glutathione peroxidase	up	5	44	[K].VDVNGDNVAPIYK.[F]
TRINITY_DN360514_c0_g1_i2_m.1005080	L-ascorbate peroxidase 6	up	4	26	[K].YAEDQDTFFEDYAEAHAK.[L]
TRINITY_DN388026_c0_g1_i1_m.2951443	Putative L-ascorbate peroxidase 4	up	2	18	[R].LAWHDAGTYDVNTR.[T]
TRINITY_DN292501_c0_g1_i1_m.3773618	glutathione S-transferase GSTF1	up	3	13	[K].YKTEEVDLLR.[E]
TRINITY_DN384059_c0_g1_i4_m.1264285	putative quinone-oxidoreductase homolog	up	1	2	[K].YGGGAEGLK.[H]
TRINITY_DN397920_c4_g1_i3_m.2061483	cytosolic glutathione reductase	up	15	38	[K].VLGAAMCGPDAAEIMQGLAVALK.[A]
TRINITY_DN309613_c0_g1_i1_m.870427	aspartic proteinase nepenthesin-1-like	up	6	16	[K].GLMLVDSGCTYTHLDTK.[I]
TRINITY_DN391067_c0_g2_i12_m.3320632	Alcohol dehydrogenase 3	up	1	19	[R].GVMIGDGESR.[F]
**Proline synthesis**					
TRINITY_DN388636_c1_g3_i8_m.158678	putative delta-1-pyrroline-5-carboxylate 1	up	1	10	[K].LDDVIDLVIPR.[G]
TRINITY_DN771345_c0_g1_i1_m.4363218	Putative ornithine aminotransferase	up	2	41	[R].LAPPLSISSEELAEASK.[A]
**Carbohydrate metabolism**					
TRINITY_DN395511_c1_g2_i2_m.3041349	neutral/alkaline invertase 1	up	1	2	[R].AIAVAEEK.[L]
TRINITY_DN367766_c0_g1_i8_m.1693602	sucrose synthase type 3	up	1	7	[K].SKDREEIEEIEK.[M]
TRINITY_DN364178_c2_g1_i14_m.3312599	fructosyltransferase	up	1	7	[K].DDNQGSHAGIAIMFK.[T]
TRINITY_DN368820_c0_g1_i4_m.3056111	Putative invertase inhibitor	up	1	4	[R].CEALYDR.[M]
TRINITY_DN397656_c1_g2_i6_m.3161266	AF310160_1 sucrose-phosphate synthase	up	1	30	[K].LRPQGVQLPPLDPAPALAAEESSAAYNPTR.[Y]
**Plant hormone**					
TRINITY_DN379907_c0_g1_i10_m.2424353	cytochrome b5 isoform E	up	2	11	[K].DATADFEDIGHSESAK.[E]
TRINITY_DN329781_c0_g1_i1_m.3164828	1-aminocyclopropane-1-carboxylate oxidase-1-like protein	up	1	7	[K].GLDGTSALQR.[F]
TRINITY_DN397168_c1_g2_i2_m.3282508	phospholipase D	up	4	28	[R].TMEMMYTDIVQALQAK.[G]
TRINITY_DN375474_c1_g1_i1_m.1512117	probable histone deacetylase 19	up	1	4	[R].ESAGTETK.[D]
**Resisting stress**					
TRINITY_DN399320_c9_g2_i1_m.1326337	17.9 kDa class I heat shock protein	up	1	8	[R].TSSSETAAFAGAR.[I]
TRINITY_DN391482_c2_g1_i9_m.3348142	chitinase 2	up	2	35	[K].QEQGSPPSYCEPR.[Q]
TRINITY_DN382591_c2_g2_i1_m.3139076	Zinc finger protein	up	1	6	[R].NMAIASAR.[M]
TRINITY_DN394104_c3_g1_i3_m.900462	Root phototropism protein 2	up	1	7	[K].ADAALAQENEALR.[S]

Continue supplemental table 3

Protein ID	Description	Up/down	Unique peptides number	Protein coverage (%)	Peptide sequences
TRINITY_DN390772_c2_g1_i4_m.1675804	Thaumatin-like pathogenesis-related protein 1	up	2	28	[K].QSSNINVPAGTSAGR.[I]
**Flavonoid metabolism**					
TRINITY_DN393940_c2_g1_i2_m.1536269	Bifunctional dihydroflavonol 4-reductase/flavanone 4-reductase	up	2	11	[K].GELETVENK.[Y]
TRINITY_DN385576_c1_g1_i4_m.2336667	Chalcone synthase 8	up	1	5	[R].IDMADTAVPK.[L]
**Carbohydrate metabolism**					
TRINITY_DN397730_c2_g1_i4_m.2923650	starch synthase 1, chloroplastic/amyloplastic	Down	6	9	[K].KGEEGTGWAFSPLTVDK.[M]
**Chlorophyll metabolism**					
TRINITY_DN388014_c2_g1_i3_m.2951173	Chlorophyll a-b binding protein 1B-20	Down	3	31	[K].NPGSVNQDPIFK.[QSN]
TRINITY_DN392261_c2_g1_i19_m.1911164	Pyridoxamine 5'-phosphate oxidase	Down	5	10	[R].VTIFGDVYPLPAEEQEWAHK.[Q]
TRINITY_DN391693_c1_g1_i9_m.3210734	Coproporphyrinogen III oxidase	Down	1	44	[R].WEYDHKPEEGTEEFK.[L]
TRINITY_DN398647_c0_g2_i2_m.2654559	divinyl chlorophyllide a 8-vinyl-reductase	Down	12	31	[K].NSQEDVVADLAPAR.[V]
TRINITY_DN375609_c0_g1_i6_m.1955928	protoporphyrinogen oxidase, chloroplastic	Down	1	11	[K].ECLIDGELQGFGQLHPR.[S]
TRINITY_DN369498_c0_g1_i2_m.1468569	protein Chlororespiratory reduction 6	Down	1	9	[R].TLDLSPVQEALGDLNSLTTAQSK.[N]
TRINITY_DN398547_c2_g2_i3_m.1124976	Leucyl-tRNA synthetase, cytoplasmic	Down	1	19	[K].VFQAEPGNK.[VL]
TRINITY_DN263154_c0_g1_i2_m.4123170	cytochrome f	Down	3	36	[K].KGGLNVGAVLILPEGFELAPPDR.[I]
TRINITY_DN362523_c0_g1_i1_m.2022092	pheophorbide a oxygenase	up	1	37	[R].GAYSAFQTLQK.[V]

CK: control, AS: 35mmol.L^{-1} Na_2CO_3:$NaHCO_3$=1:1.
