# Supplementary material for: Proteomic Responses to Alkali Stress in Oats and the Alleviatory Effects of Exogenous Spermine Application
Source: Front Plant Sci. 2021 Apr 1;12:627129. doi: 10.3389/fpls.2021.627129 (PMC8049610; doi:10.3389/fpls.2021.627129)
Supplement: Supplementary file 12 [file Table_4.pdf]

# SUPPLEMENTAL TABLE 4 The DEPs of roots at AS vs Ck

| Protein ID | Description | Ratio | P-value | Go number |
|---|---|---|---|---|
| | **Up-regulation** | | | |
| TRINITY_DN399723_c2_g2_i2_m.3144297 | Sugar carrier protein C | 2.29 | 0.0474 | GO:0015992;GO:0046323;GO:1904659;GO:0005887;GO:0005355;GO:0005351 |
| TRINITY_DN739510_c0_g1_i1_m.4302853 | alpha glucoside ABC transporter substrate binding protein | 2.61 | 0.0150 | GO:0006810;GO:0005215 |
| TRINITY_DN359401_c0_g1_i1_m.2478511 | 30S ribosomal protein S16 | 3.25 | 0.0017 | GO:0006412;GO:0005840;GO:0003735 |
| TRINITY_DN384576_c4_g1_i3_m.2354173 | Subtilisin chymotrypsin inhibitor CI 1B | 2.60 | 0.0033 | GO:0010951;GO:0009611;GO:0004867 |
| TRINITY_DN387100_c1_g1_i2_m.1414091 | dihydrolipoyl dehydrogenase | 3.27 | 0.0006 | GO:0045454;GO:0006096;GO:0055114;GO:0005623;GO:0050660;GO:0004148 |
| TRINITY_DN366406_c0_g1_i2_m.2636647 | probable sugar phosphate/phosphate translocator At3g11320 | 2.11 | 0.0014 | GO:0016021 |
| TRINITY_DN377969_c0_g1_i1_m.2831558 | translation initiation factor IF 3 | 3.17 | 0.0058 | GO:0006413;GO:0005737;GO:0003743 |
| TRINITY_DN359412_c0_g1_i2_m.1989121 | ATP F0F1 synthase subunit B | 2.53 | 0.0187 | GO:0042777;GO:0016021;GO:0005886;GO:0045263;GO:0046933 |
| TRINITY_DN399120_c1_g1_i4_m.913870 | Tryptophan aminotransferase 1 | 1.74 | 0.0132 | GO:0016021;GO:0008483;GO:0016846 |
| TRINITY_DN386726_c1_g2_i1_m.2811208 | NADPH dependent FMN reductase | 3.04 | 0.0026 | GO:0055114;GO:0016491 |
| TRINITY_DN290195_c0_g1_i2_m.3944440 | TonB dependent receptor | 2.30 | 0.0024 | GO:0006810;GO:0009279;GO:0004872 |
| TRINITY_DN394643_c1_g1_i24_m.1459415 | Protein TRANSPARENT TESTA 12 | 1.99 | 0.0020 | GO:0006855;GO:0016021;GO:0015297;GO:0015238 |
| TRINITY_DN765027_c0_g1_i1_m.4204123 | nitrous oxide reductase | 2.06 | 0.0051 | GO:1902600;GO:0055114;GO:0016020;GO:0004129;GO:0050304;GO:0005509;GO:0005507 |
| TRINITY_DN375800_c1_g1_i11_m.938155 | probable FBA1 fructose bisphosphate aldolase | 2.30 | 0.0002 | GO:0006096;GO:0006094;GO:0005829;GO:0005739;GO:0005634;GO:0008270;GO:0004332 |
| TRINITY_DN280167_c0_g1_i1_m.3845377 | superoxide dismutase | 2.38 | 0.0103 | GO:0019430;GO:0055114;GO:0046872;GO:0004784 |
| TRINITY_DN298984_c0_g1_i1_m.3929424 | adenylosuccinate synthetase | 1.82 | 0.0109 | GO:0044208;GO:0005737;GO:0004019;GO:0000287;GO:0005525 |
| TRINITY_DN367449_c0_g1_i10_m.2615214 | peroxidase A2 | 1.90 | 0.0032 | GO:0098869;GO:0042744;GO:0006979;GO:0055114;GO:0005576;GO:0004497;GO:0020037;GO:0016705;GO:0004601;GO:0005506 |
| TRINITY_DN672645_c0_g1_i1_m.157038 | protease modulator HflC | 3.16 | 0.0106 | GO:0052547;GO:0016021;GO:0008233 |
| TRINITY_DN370127_c0_g2_i1_m.1205334 | Subtilisin chymotrypsin inhibitor 2A | 2.38 | 0.0139 | GO:0010951;GO:0009611;GO:0016021;GO:0004867 |
| TRINITY_DN309435_c0_g1_i2_m.2237711 | elongation factor Tu | 3.64 | 0.0003 | GO:0006414;GO:0005737;GO:0003746;GO:0003924;GO:0005525 |
| TRINITY_DN368009_c2_g1_i2_m.3297778 | 30S ribosomal protein S6 | 3.49 | 0.0137 | GO:0006412;GO:0005840;GO:0003735;GO:0019843 |
| TRINITY_DN379195_c0_g1_i3_m.1218852 | vacuolar protease A | 2.94 | 0.0017 | GO:0051603;GO:0016237;GO:0009267;GO:0004190 |
| TRINITY_DN365985_c13_g2_i1_m.1453592 | molecular chaperone DnaK | 3.22 | 0.0235 | GO:0006457;GO:0051082;GO:0005524 |
| TRINITY_DN151967_c0_g1_i1_m.558873 | ABC transporter ATP binding protein | 2.23 | 0.0235 | GO:0003333;GO:0015424;GO:0005524 |
| TRINITY_DN175458_c0_g1_i2_m.461269 | probable binding protein component of ABC sugar transporter | 2.92 | 0.0374 | GO:0008643;GO:0005215 |
| TRINITY_DN367449_c0_g1_i4_m.2615188 | Peroxidase 54 | 1.93 | 0.0086 | GO:0098869;GO:0042744;GO:0006979;GO:0055114;GO:0005576;GO:0004497;GO:0020037;GO:0016705;GO:0004601;GO:0005506 |
| TRINITY_DN778192_c0_g1_i1_m.4212009 | integration host factor subunit alpha | 3.07 | 0.0000 | GO:0006417;GO:0006355;GO:0006310;GO:0003677 |
| TRINITY_DN351172_c1_g1_i3_m.1872766 | porin | 2.49 | 0.0001 | GO:0016021;GO:0009279 |
| TRINITY_DN312566_c0_g2_i1_m.2432846 | elongation factor P | 2.54 | 0.0424 | GO:0006414;GO:0005737;GO:0003746 |
| TRINITY_DN373968_c0_g3_i1_m.2360392 | Glucokinase | 7.70 | 0.0216 | GO:0051156;GO:0006096;GO:0016021;GO:0004340;GO:0016874;GO:0005524 |
| TRINITY_DN275300_c0_g1_i2_m.4059446 | branched chain amino acid ABC transporter substrate binding protein | 4.17 | 0.0231 | GO:0006865 |
| TRINITY_DN356061_c2_g1_i2_m.2312333 | Ribosomal protein S9 | 1.89 | 0.0344 | GO:0006412;GO:0005840;GO:0003735 |
| TRINITY_DN380124_c1_g1_i16_m.3329408 | UDP arabinopyranose mutase 2 like | 2.55 | 0.0003 | GO:0009832;GO:0033356;GO:0030244;GO:0071555;GO:0005794;GO:0005829;GO:0052691;GO:0005515 |
| TRINITY_DN312401_c0_g1_i1_m.1401364 | Vacuolar processing enzyme | 2.43 | 0.0040 | GO:0006624;GO:0051603;GO:0005773;GO:0004197 |
| TRINITY_DN324572_c0_g3_i1_m.1041365 | 60 kDa jasmonate induced protein | 1.98 | 0.0065 | GO:0016787 |

| | | | | |
|---|---|---|---|---|
| TRINITY_DN346089_c2_g1_i8_m.3355310 | DNA directed RNA polymerase subunit beta' | 2.45 | 0.0078 | GO:0006351;GO:0003899;GO:0003677 |
| TRINITY_DN389816_c1_g2_i6_m.2487642 | 50S ribosomal protein L24 | 2.05 | 0.0015 | GO:0006412;GO:0005840;GO:0003735;GO:0019843 |
| TRINITY_DN360359_c3_g1_i1_m.1832144 | cytosolic nonspecific dipeptidase | 2.00 | 0.0083 | GO:0006508;GO:0005737;GO:0016805;GO:0008237;GO:0034701 |
| TRINITY_DN300717_c0_g1_i1_m.1069567 | Subtilisin like protease | 1.98 | 0.0053 | GO:0005985;GO:0016157 |
| TRINITY_DN312316_c0_g2_i2_m.1401502 | transcriptional regulator HU subunit alpha | 1.87 | 0.0280 | GO:0030261;GO:0003677 |
| TRINITY_DN87418_c0_g1_i2_m.4368525 | 30S ribosomal protein S14 | 1.99 | 0.0003 | GO:0006412;GO:0022627;GO:0003735;GO:0019843 |
| TRINITY_DN345321_c0_g2_i4_m.1612777 | elongation factor Ts | 1.94 | 0.0009 | GO:0006414;GO:0005737;GO:0003746 |
| TRINITY_DN348653_c0_g1_i2_m.2244557 | type I glutamate ammonia ligase | 2.05 | 0.0027 | GO:0009399;GO:0006542;GO:0005737;GO:0004356;GO:0005524 |
| TRINITY_DN394286_c3_g1_i9_m.2220290 | carrier protein | 1.57 | 0.0367 | GO:0055085;GO:0005743;GO:0016021;GO:0005215 |
| TRINITY_DN1009555_c0_g1_i1_m.4645346 | serine glyoxylate aminotransferase | 2.26 | 0.0241 | GO:0008483 |
| TRINITY_DN389448_c0_g2_i1_m.1814421 | 30S ribosomal protein S13 | 1.88 | 0.0093 | GO:0006412;GO:0005840;GO:0003735;GO:0000049;GO:0019843 |
| TRINITY_DN116390_c0_g1_i1_m.428861 | ATP binding protein | 2.06 | 0.0058 | GO:1990022;GO:0044376;GO:0034087;GO:0005737 |
| TRINITY_DN93173_c0_g1_i1_m.4395366 | pyruvate kinase | 1.91 | 0.0184 | GO:0006096;GO:0030955;GO:0000287;GO:0004743;GO:0016301 |
| TRINITY_DN392237_c3_g1_i6_m.1907371 | 50S ribosomal protein L9 | 2.43 | 0.0194 | GO:0006412;GO:0005840;GO:0003735;GO:0019843 |
| TRINITY_DN384784_c3_g2_i4_m.1760807 | 50S ribosomal protein L21 | 1.97 | 0.0338 | GO:0006412;GO:0005840;GO:0003735;GO:0019843 |
| TRINITY_DN273935_c0_g2_i1_m.3759547 | dihydrolipoamide acetyltransferase | 2.00 | 0.0003 | GO:0006096;GO:0045254;GO:0004742 |
| TRINITY_DN360622_c4_g6_i1_m.1268910 | putative heat shock protein ssb1 protein | 1.53 | 0.0427 | GO:0005829;GO:0005524 |
| TRINITY_DN1015537_c0_g1_i1_m.4592851 | alkyl hydroperoxide reductase | 1.68 | 0.0051 | GO:0098869;GO:0055114;GO:0051920 |
| TRINITY_DN389816_c1_g2_i6_m.2487634 | 50S ribosomal protein L6 | 1.67 | 0.0029 | GO:0006412;GO:0005840;GO:0003735;GO:0019843 |
| TRINITY_DN387814_c0_g1_i1_m.1640972 | 2,3 bisphosphoglycerate independent phosphoglycerate mutase like isoform X2 | 1.59 | 0.0054 | GO:0006007;GO:0006096;GO:0005737;GO:0004619;GO:0030145 |
| TRINITY_DN391530_c4_g4_i4_m.2833369 | ADP,ATP carrier protein, mitochondrial | 1.59 | 0.0003 | GO:0006412;GO:0055085;GO:0016021;GO:0005743;GO:0005215;GO:0003735 |
| TRINITY_DN385803_c0_g1_i9_m.1142374 | tonoplast intrinsic protein | 1.61 | 0.0004 | GO:0009992;GO:0034220;GO:0006833;GO:0015793;GO:0009705;GO:0005887;GO:0042807;GO:0015250;GO:0015254 |
| TRINITY_DN368790_c0_g1_i3_m.1346522 | cold regulated protein | 1.58 | 0.0005 | GO:0016021 |
| TRINITY_DN394135_c0_g2_i2_m.1897722 | cytochrome P450 monooxygenase CYP710A8d | 1.27 | 0.0215 | GO:0016126;GO:0022900;GO:0016021;GO:0004497;GO:0020037;GO:0000249;GO:0005506 |
| TRINITY_DN348962_c0_g5_i1_m.1696024 | cytochrome b c1 complex subunit 6 | 1.46 | 0.0003 | GO:1902600;GO:0006122;GO:0005750;GO:0045153;GO:0008121 |
| TRINITY_DN390569_c0_g1_i4_m.1824555 | transmembrane emp24 domain containing protein p24delta9 | 1.35 | 0.0077 | GO:0006810;GO:0016021;GO:0005739;GO:0005789 |
| TRINITY_DN398835_c2_g3_i2_m.1561605 | mitochondrial outer membrane protein porin 5 | 1.49 | 0.0002 | GO:1903959;GO:0005741;GO:0046930;GO:0015288;GO:0008308 |
| TRINITY_DN373129_c0_g1_i6_m.2496044 | mitochondrial outer membrane protein porin 3 | 1.37 | 0.0005 | GO:1903959;GO:0009527;GO:0046930;GO:0005741;GO:0015288;GO:0008308 |
| TRINITY_DN996309_c0_g1_i1_m.302198 | nitrate reductase subunit alpha | 2.29 | 0.0053 | GO:0042126;GO:0055114;GO:0009325;GO:0008940;GO:0051539;GO:0030151;GO:0009055 |
| TRINITY_DN381682_c0_g1_i4_m.3291682 | D mannose binding lectin domain related protein precursor | 1.37 | 0.0082 | GO:0016310;GO:0016020;GO:0030246;GO:0004672 |
| TRINITY_DN770500_c0_g1_i1_m.4339256 | malate dehydrogenase | 1.79 | 0.0026 | GO:0006099;GO:0005975;GO:0006108;GO:0030060 |
| TRINITY_DN383389_c1_g2_i4_m.979842 | Succinate/fumarate mitochondrial transporter | 1.42 | 0.0033 | GO:0071422;GO:0006412;GO:0009536;GO:0016021;GO:0003735;GO:0015141 |
| TRINITY_DN394578_c2_g3_i7_m.2966802 | Putative membrane protein | 1.55 | 0.0000 | GO:0009941;GO:0016021;GO:0005774;GO:0005886;GO:0009506;GO:0008270 |
| TRINITY_DN373129_c0_g1_i12_m.2496060 | mitochondrial outer membrane protein porin 3 | 1.34 | 0.0046 | GO:1903959;GO:0009527;GO:0046930;GO:0005741;GO:0015288;GO:0008308 |
| TRINITY_DN382856_c1_g1_i9_m.2542095 | putative glutathione specific gamma glutamylcyclotransferase 2 | 1.35 | 0.0142 | GO:0005829 |
| TRINITY_DN336786_c0_g1_i1_m.1271200 | Hyoscyamine 6 dioxygenase | 1.68 | 0.0139 | GO:0055114;GO:0046872;GO:0051213 |
| TRINITY_DN312036_c0_g2_i1_m.1802793 | 2,3 bisphosphoglycerate independent phosphoglycerate mutase | 1.29 | 0.0176 | GO:0006007;GO:0005737;GO:0004619;GO:0030145 |
| TRINITY_DN399649_c1_g1_i9_m.3201863 | Putative flavin containing monooxygenase 1 | 1.20 | 0.0479 | GO:0055114;GO:0050661;GO:0050660;GO:0004499 |
| TRINITY_DN390114_c0_g1_i14_m.2089684 | threonine tRNA ligase, mitochondrial 1 | 1.38 | 0.0062 | GO:0006435;GO:0005739;GO:0005524;GO:0003723;GO:0004829 |
| TRINITY_DN385640_c1_g1_i11_m.3361640 | Urease | 1.60 | 0.0003 | GO:0043419;GO:0016151;GO:0009039 |
| TRINITY_DN382828_c0_g1_i6_m.2542287 | scopoletin glucosyltransferase like | 1.39 | 0.0034 | GO:0052696;GO:0009813;GO:0043231;GO:0016021;GO:0080043;GO:0080044 |
| TRINITY_DN391190_c0_g1_i10_m.2106641 | probable mitochondrial processing peptidase subunit beta | 1.34 | 0.0073 | GO:0006122;GO:0009060;GO:0016485;GO:0005750;GO:0004222;GO:0008270 |

| | | | | |
|---|---|---|---|---|
| TRINITY_DN234234_c0_g1_i2_m.3915865 | Ribose phosphate pyrophosphokinase 4 | 1.43 | 0.0119 | GO:0006783;GO:0016310;GO:0009220;GO:0009116;GO:0005829;GO:0009506;GO:0005886;GO:0016301;GO:0000287;GO:0004749;GO:0005524 |
| TRINITY_DN360449_c0_g1_i3_m.1215511 | probable leucine rich repeat receptor like protein kinase At5g49770 | 1.34 | 0.0073 | GO:0006468;GO:0005739;GO:0016021;GO:0004674;GO:0005524 |
| TRINITY_DN388825_c0_g1_i11_m.1729777 | probable small nuclear ribonucleoprotein G | 1.40 | 0.0033 | GO:0000398;GO:0071004;GO:0043186;GO:0005732;GO:0071011;GO:0071013;GO:0097526;GO:0019013;GO:0005689;GO:0005687;GO:0005686;GO:0005685;GO:0034719;GO:0005682;GO:0003723 |
| TRINITY_DN282835_c0_g1_i2_m.4051799 | succinate dehydrogenase flavoprotein subunit | 1.28 | 0.0063 | GO:0022900;GO:0006099;GO:0005886;GO:0050660;GO:0008177 |
| TRINITY_DN399784_c4_g1_i2_m.3143531 | ABC transporter C family member 2 | 1.44 | 0.0001 | GO:0055085;GO:0016021;GO:0042626;GO:0005524 |
| TRINITY_DN310538_c0_g1_i2_m.1174961 | ras related protein RABA2a like | 1.28 | 0.0009 | GO:0007264;GO:0005768;GO:0005525 |
| TRINITY_DN388279_c1_g1_i3_m.1185799 | probable glutamate carboxypeptidase 2 | 1.40 | 0.0030 | GO:0006508;GO:0016021;GO:0004180 |
| TRINITY_DN387938_c0_g2_i2_m.1831045 | vacuole membrane protein KMS1 | 1.21 | 0.0068 | GO:0007030;GO:0007029;GO:0006887;GO:0010506;GO:0005783;GO:0016021;GO:0000407 |
| TRINITY_DN237878_c0_g1_i2_m.3853245 | alternative oxidase | 1.33 | 0.0090 | GO:0055114;GO:0016021;GO:0005739;GO:0070469;GO:0009916;GO:0046872 |
| TRINITY_DN398623_c1_g1_i2_m.2656491 | 5 methyltetrahydropteroyltriglutamate homocysteine methyltransferase | 1.40 | 0.0056 | GO:0032259;GO:0050667;GO:0009086;GO:0005829;GO:0005576;GO:0008705;GO:0008270;GO:0003871 |
| TRINITY_DN301992_c0_g2_i1_m.1403757 | Cortical cell delineating protein | 1.38 | 0.0005 | GO:0006869;GO:0008289 |
| TRINITY_DN378758_c0_g2_i6_m.1483539 | E3 ubiquitin protein ligase listerin | 1.23 | 0.0475 | GO:0000911;GO:0006486;GO:0010162;GO:0050826;GO:0009793;GO:0009933;GO:0009845;GO:0010228;GO:0009630;GO:0000226;GO:0010182;GO:0009909;GO:0009640;GO:0005829;GO:0008270 |
| TRINITY_DN365158_c2_g1_i2_m.1643858 | carbamoyl phosphate synthase large subunit | 1.27 | 0.0087 | GO:0044205;GO:0006526;GO:0004088;GO:0046872;GO:0005524 |
| TRINITY_DN390918_c0_g1_i11_m.2250373 | omega amidase | 1.41 | 0.0017 | GO:0006107;GO:0006807;GO:0006108;GO:0009570;GO:0005829;GO:0016746;GO:0008270;GO:0050152 |
| TRINITY_DN390361_c3_g1_i5_m.2622544 | Flavoprotein wrbA | 1.24 | 0.0011 | GO:0045892;GO:0055114;GO:0010181;GO:0016491 |
| TRINITY_DN376885_c1_g2_i2_m.1133559 | | 1.33 | 0.0003 | GO:0016021;GO:0005747 |
| TRINITY_DN161797_c0_g1_i2_m.536473 | Dolichyl diphosphooligosaccharide protein glycosyltransferase subunit 1 | 1.28 | 0.0097 | GO:0000394;GO:0006499;GO:0030244;GO:0009086;GO:0018279;GO:0016021;GO:0008250;GO:0004579 |
| TRINITY_DN378026_c0_g1_i9_m.3114861 | Annexin D4 | 1.29 | 0.0131 | GO:0009737;GO:0006970;GO:0016021;GO:0009506;GO:0005544;GO:0005509 |
| TRINITY_DN72586_c0_g1_i1_m.4554410 | putative germinal histone H4 protein | 1.27 | 0.0087 | GO:0006352;GO:0006334;GO:0005634;GO:0000786;GO:0003677;GO:0046982 |
| TRINITY_DN381520_c0_g2_i6_m.1503614 | Putative chloride channel like protein CLC g | 1.33 | 0.0334 | GO:0006821;GO:1903959;GO:0016021;GO:0005247 |
| TRINITY_DN220160_c0_g2_i1_m.3812871 | cellulose synthase A catalytic subunit 1 [UDP forming] | 1.32 | 0.0008 | GO:0030244;GO:0071555;GO:0016021;GO:0005886;GO:0016760;GO:0008270 |
| TRINITY_DN398030_c3_g1_i13_m.953156 | Beta glucosidase 6 | 1.45 | 0.0395 | GO:1901657;GO:0030245;GO:0005829;GO:0102483;GO:0008422 |
| TRINITY_DN350942_c0_g1_i4_m.1016905 | GDT1 like protein 5 isoform X1 | 1.36 | 0.0033 | GO:0016021 |
| TRINITY_DN328631_c0_g1_i1_m.3111884 | Aldose reductase | 1.23 | 0.0406 | GO:0055114;GO:0016491 |
| TRINITY_DN382230_c2_g1_i6_m.1497916 | Acetylornithine deacetylase | 1.32 | 0.0087 | GO:0008152;GO:0016787 |
| TRINITY_DN396593_c2_g2_i1_m.2308052 | Puromycin sensitive aminopeptidase | 1.38 | 0.0001 | GO:0006869;GO:0043171;GO:0006508;GO:0031090;GO:0005886;GO:0005783;GO:0005319;GO:0042277;GO:0070006;GO:0008270 |
| TRINITY_DN384497_c3_g1_i6_m.1583173 | ER membrane protein complex subunit 10 | 1.46 | 0.0002 | GO:0005783;GO:0005774 |
| TRINITY_DN395863_c1_g1_i8_m.1523481 | Putative DNA binding protein ESCAROLA | 1.21 | 0.0269 | GO:0003677 |
| TRINITY_DN369554_c2_g1_i8_m.3039639 | Endo 1,3;1,4 beta D glucanase | 1.71 | 0.0144 | GO:0016787 |
| TRINITY_DN399588_c0_g1_i2_m.3002124 | nucleoprotein TPR isoform X2 | 1.26 | 0.0059 | GO:0009536 |
| TRINITY_DN348432_c2_g1_i1_m.2982814 | 30S ribosomal protein S8 | 1.30 | 0.0409 | GO:0006412;GO:0005840;GO:0003735;GO:0019843 |
| TRINITY_DN374537_c0_g1_i1_m.2412134 | ubiquinol cytochrome c reductase core subunit 2 | 1.35 | 0.0220 | GO:1902600;GO:0006122;GO:0009060;GO:0005750;GO:0030061;GO:0008121;GO:0046872 |
| TRINITY_DN602880_c0_g1_i1_m.132796 | glycoside hydrolase family 7 | 1.39 | 0.0376 | GO:0030245;GO:0005576;GO:0016162;GO:0030248 |
| TRINITY_DN221724_c0_g1_i1_m.3933092 | ADP,ATP carrier protein 2 | 1.65 | 0.0040 | GO:0006412;GO:0055085;GO:0016021;GO:0005743;GO:0005215;GO:0003735 |

| | | | | |
|---|---|---|---|---|
| TRINITY_DN396532_c0_g1_i16_m.2309986 | calcium transporting ATPase 3, endoplasmic reticulum type isoform X2 | 1.28 | 0.0278 | GO:0071421;GO:0070588;GO:0055071;GO:0048364;GO:0016021;GO:0005802;GO:0005768;GO:0005388;GO:0046872;GO:0005524;GO:0015410 |
| TRINITY_DN165962_c0_g1_i1_m.647487 | 50S ribosomal protein L11 | 1.37 | 0.0014 | GO:0006412;GO:0022625;GO:0003735;GO:0070180 |
| TRINITY_DN385049_c0_g3_i1_m.2191310 | pathogenesis related protein 1 like | 1.30 | 0.0005 | GO:0005576 |
| TRINITY_DN393274_c0_g1_i3_m.1879295 | venom phosphodiesterase 2 like | 1.39 | 0.0043 | GO:0000394;GO:0090305;GO:0009086;GO:0009536;GO:0005773;GO:0016021;GO:0004528;GO:0035529 |
| TRINITY_DN376484_c0_g2_i1_m.866747 | B cell receptor associated protein 29 | 1.28 | 0.0169 | GO:0006886;GO:0016021;GO:0005783 |
| TRINITY_DN399115_c5_g1_i12_m.915497 | Protein SRG1 | 1.33 | 0.0096 | GO:0055114;GO:0005506;GO:0051213 |
| TRINITY_DN365955_c1_g1_i1_m.1450806 | signal peptidase complex subunit 3B | 1.54 | 0.0016 | GO:0006465;GO:0005787;GO:0016021;GO:0008233 |
| TRINITY_DN394613_c0_g1_i3_m.1457879 | phospholipid:diacylglycerol acyltransferase 1 | 1.31 | 0.0207 | GO:0006629;GO:0005773;GO:0016021;GO:0005783;GO:0008374 |
| TRINITY_DN396817_c1_g2_i2_m.2568543 | glycerophosphodiester phosphodiesterase GDPDL3 | 1.24 | 0.0013 | GO:0006629;GO:0016020;GO:0008889 |
| TRINITY_DN329113_c0_g1_i2_m.1974517 | alpha glucosidase like protein | 1.35 | 0.0132 | GO:0005975;GO:0016021;GO:0009507;GO:0030246;GO:0004553 |
| TRINITY_DN168325_c0_g2_i1_m.471454 | Calmodulin | 1.43 | 0.0095 | GO:0005509 |
| TRINITY_DN397733_c2_g2_i5_m.2928829 | transmembrane 9 superfamily member 2 like | 1.27 | 0.0016 | GO:0006412;GO:0016021;GO:0005840;GO:0003735 |
| TRINITY_DN386086_c0_g1_i1_m.2970675 | histone deacetylase complex subunit SAP18 | 1.28 | 0.0062 | GO:0009651;GO:0005730 |
| TRINITY_DN396092_c0_g1_i4_m.1777895 | Glycerol 3 phosphate dehydrogenase SDP6 | 1.26 | 0.0309 | GO:0019761;GO:0019563;GO:0006127;GO:0006355;GO:0015824;GO:0006007;GO:0009331;GO:0005739;GO:0052591 |
| TRINITY_DN388026_c0_g1_i1_m.2951443 | Putative L ascorbate peroxidase 4 | 1.37 | 0.0219 | GO:0098869;GO:0042744;GO:0006979;GO:0055114;GO:0016021;GO:0020037;GO:0016688;GO:0046872 |
| TRINITY_DN392651_c0_g1_i4_m.2118712 | alpha 1,3 mannosyl glycoprotein 2 beta N acetylglucosaminyltransferase isoform X1 | 1.23 | 0.0005 | GO:0006491;GO:0006972;GO:0005802;GO:0005768;GO:0016262;GO:0003827 |
| TRINITY_DN398003_c2_g1_i8_m.2058598 | 1 acyl sn glycerol 3 phosphate acyltransferase 1 | 1.23 | 0.0458 | GO:0006655;GO:0015995;GO:0006636;GO:0010228;GO:0009793;GO:0019288;GO:0016226;GO:0048481;GO:0016117;GO:0010027;GO:0009941;GO:0016021;GO:0003841 |
| TRINITY_DN291676_c0_g1_i1_m.3793600 | GDT1 like protein 5 | 1.67 | 0.0087 | GO:0016021 |
| TRINITY_DN377316_c1_g1_i2_m.2688105 | transmembrane protein 87B | 1.38 | 0.0080 | GO:0016021 |
| TRINITY_DN390294_c1_g1_i3_m.2367474 | vacuolar sorting receptor 1 like | 1.22 | 0.0007 | GO:0016021;GO:0005509 |
| TRINITY_DN316120_c0_g1_i6_m.969805 | Zinc metallopeptidase | 1.23 | 0.0187 | GO:0051603;GO:0043171;GO:0016485;GO:0005739;GO:0004222;GO:0008270 |
| TRINITY_DN360174_c0_g1_i2_m.955313 | Cytochrome b c1 complex subunit Rieske | 1.39 | 0.0102 | GO:1902600;GO:0055114;GO:0016021;GO:0005743;GO:0070469;GO:0051537;GO:0008121;GO:0046872 |
| TRINITY_DN395572_c1_g1_i10_m.3044912 | LysM domain containing GPI anchored protein 2 | 1.29 | 0.0006 | GO:0006468;GO:0045087;GO:0051707;GO:0010033;GO:0016020;GO:0097367;GO:0043167;GO:0004672 |
| TRINITY_DN374278_c1_g1_i6_m.2300628 | inositol monophosphatase 3 | 1.25 | 0.0124 | GO:0019853;GO:0007165;GO:0046855;GO:0046854;GO:0009409;GO:0006021;GO:0005829;GO:0005886;GO:0008934;GO:0000287;GO:0010347 |
| TRINITY_DN397541_c1_g1_i3_m.2299318 | glucose 6 phosphate isomerase | 1.24 | 0.0092 | GO:0009744;GO:0009817;GO:0009813;GO:0006096;GO:0010224;GO:0006094;GO:0005829;GO:0004347 |
| TRINITY_DN399718_c3_g1_i4_m.3146475 | trehalose 6 phosphate synthase | 1.27 | 0.0424 | GO:0005992;GO:0005739;GO:0003825 |
| TRINITY_DN68481_c0_g1_i1_m.4467248 | DNA directed RNA polymerase II subunit RPB11 | 1.35 | 0.0008 | GO:0006351;GO:0003899;GO:0046983;GO:0003677 |
| TRINITY_DN385510_c2_g2_i7_m.2341342 | NADH dehydrogenase subunit 4 | 1.43 | 0.0014 | GO:0042773;GO:0016021;GO:0070469;GO:0031966;GO:0008137 |
| TRINITY_DN399179_c6_g2_i3_m.912343 | protein CIA1 | 1.24 | 0.0204 | GO:0016226;GO:0097361;GO:0016746 |
| TRINITY_DN389251_c0_g3_i2_m.3170670 | secretory carrier associated membrane protein 6 | 1.38 | 0.0157 | GO:0015031;GO:0030658;GO:0016021;GO:0005886 |
| TRINITY_DN319879_c0_g1_i1_m.3058361 | 6 phosphogluconate dehydrogenase, decarboxylating 2 | 1.27 | 0.0072 | GO:0009737;GO:0019521;GO:0006098;GO:0009414;GO:0009409;GO:0009651;GO:0055114;GO:0009507;GO:0004616 |
| TRINITY_DN379237_c0_g1_i3_m.2131322 | EF hand family protein | 1.38 | 0.0040 | GO:0006508;GO:0005737;GO:0004198;GO:0005509 |
| TRINITY_DN392909_c1_g2_i5_m.2523614 | sugar transporter ERD6 like 4 可以的 | 1.28 | 0.0064 | GO:0015992;GO:0046323;GO:1904659;GO:0005887;GO:0005355;GO:0005351 |

| | | | | |
|---|---|---|---|---|
| TRINITY_DN394159_c0_g1_i6_m.1895295 | subtilisin like protease SBT1.7 | 1.33 | 0.0072 | GO:0006508;GO:0005618;GO:0004252 |
| TRINITY_DN387359_c0_g1_i2_m.1543120 | aspartate aminotransferase P2 | 1.22 | 0.0192 | GO:0006520;GO:0009058;GO:0009941;GO:0009570;GO:0010319;GO:0048046;GO:0005739;GO:0080130;GO:0004069;GO:0030170;GO:0042802 |
| TRINITY_DN394978_c1_g2_i6_m.2428405 | putative inosine 5' monophosphate dehydrogenase | 1.36 | 0.0237 | GO:0006177;GO:0055114;GO:0005737;GO:0046872;GO:0003938;GO:0000166 |
| TRINITY_DN393136_c1_g1_i1_m.1551992 | hypersensitive induced response protein 1 | 1.25 | 0.0250 | GO:0016020 |
| TRINITY_DN379719_c0_g1_i18_m.2462092 | ABC transporter B family member 20 | 1.21 | 0.0143 | GO:0055085;GO:0016021;GO:0042626;GO:0005524 |
| TRINITY_DN367210_c0_g1_i2_m.1107712 | 2,3 dimethylmalate lyase | 1.33 | 0.0118 | GO:0006097;GO:0009536;GO:0046912;GO:0016829 |
| TRINITY_DN261030_c0_g1_i2_m.3894600 | prepilin type N terminal cleavage/methylation domain containing protein | 4.77 | 0.0124 | GO:0016020 |
| TRINITY_DN136836_c0_g1_i1_m.617031 | ABC transporter substrate binding protein | 4.41 | 0.0002 | GO:0006810;GO:0005215 |
| TRINITY_DN95618_c0_g1_i2_m.4499333 | multifunctional fatty acid oxidation complex subunit alpha | 4.35 | 0.0005 | GO:0006635;GO:0036125;GO:0004165;GO:0008692;GO:0004300;GO:0003857 |
| TRINITY_DN378494_c1_g2_i4_m.2240858 | 30S ribosomal protein S10 | 2.92 | 0.0019 | GO:0006412;GO:0005840;GO:0003735;GO:0000049 |
| TRINITY_DN251781_c0_g1_i1_m.4078044 | ATP F0F1 synthase subunit gamma | 3.36 | 0.0013 | GO:0042777;GO:0045261;GO:0005886;GO:0046933;GO:0046961;GO:0005524 |
| TRINITY_DN287399_c0_g1_i1_m.3912434 | cytochrome c550 | 2.83 | 0.0436 | GO:0020037;GO:0009055 |
| TRINITY_DN368224_c3_g1_i4_m.1448338 | 30S ribosomal protein S1 | 2.00 | 0.0184 | GO:0006412;GO:0005840;GO:0003735;GO:0003723 |
| TRINITY_DN231492_c0_g1_i2_m.3791112 | C4 dicarboxylate ABC transporter | 2.37 | 0.0149 | GO:0006810;GO:0030288 |
| TRINITY_DN348643_c0_g1_i4_m.2244734 | 50S ribosomal protein L27 | 2.84 | 0.0059 | GO:0006412;GO:0005840;GO:0003735 |
| TRINITY_DN362714_c0_g3_i1_m.2587606 | cytochrome c | 2.86 | 0.0195 | GO:0055114;GO:0042597;GO:0005506;GO:0020037;GO:0009055 |
| TRINITY_DN399318_c3_g2_i2_m.1325840 | 30S ribosomal protein S7 | 2.63 | 0.0022 | GO:0006412;GO:0015935;GO:0003735;GO:0000049;GO:0019843 |
| TRINITY_DN389816_c1_g2_i6_m.2487633 | 50S ribosomal protein L5 | 1.68 | 0.0287 | GO:0006412;GO:0005840;GO:0003735;GO:0000049;GO:0019843 |
| TRINITY_DN986201_c0_g1_i1_m.365011 | NADP dependent malic enzyme MaeB | 2.05 | 0.0047 | GO:0006108;GO:0055114;GO:0051287;GO:0004471;GO:0046872 |
| TRINITY_DN399320_c9_g2_i1_m.1326337 | 17.9 kDa class I heat shock protein | 1.79 | 0.0156 | GO:0042542;GO:0045471;GO:0046688;GO:0046686;GO:0009408;GO:0046685;GO:0005737;GO:0005634 |
| TRINITY_DN296129_c0_g1_i1_m.3967255 | isocitrate dehydrogenase | 2.03 | 0.0003 | GO:0006099;GO:0006097;GO:0051287;GO:0000287;GO:0004450 |
| TRINITY_DN389810_c1_g1_i5_m.2490232 | chitinase | 1.81 | 0.0121 | GO:0005975;GO:0006032;GO:0005576;GO:0004568;GO:0008061 |
| TRINITY_DN180077_c0_g1_i1_m.578215 | ATP synthase subunit alpha | 1.75 | 0.0410 | GO:0015991;GO:0042777;GO:0045261;GO:0005886;GO:0046933;GO:0046961;GO:0005524 |
| TRINITY_DN374391_c1_g1_i1_m.1768076 | Cysteine proteinase inhibitor | 1.78 | 0.0008 | GO:0010951;GO:0006952;GO:0009536;GO:0004869;GO:0042802 |
| TRINITY_DN360717_c1_g6_i2_m.1664850 | 60S ribosomal protein L26 1 | 1.59 | 0.0379 | GO:0006412;GO:0016021;GO:0015934;GO:0003735;GO:0005507 |
| TRINITY_DN166798_c0_g1_i1_m.482243 | 40S ribosomal protein S7 | 1.71 | 0.0159 | GO:0042274;GO:0006357;GO:0006364;GO:0006412;GO:0032040;GO:0022627;GO:0005730;GO:0030686;GO:0003735;GO:0000981;GO:0008270 |
| TRINITY_DN359527_c0_g1_i1_m.1043819 | probable glutathione S transferase GSTU6 | 1.77 | 0.0011 | GO:0006749;GO:0009407;GO:0005739;GO:0004364 |
| TRINITY_DN309296_c0_g1_i1_m.2876981 | 50S ribosomal protein L22 | 1.65 | 0.0017 | GO:0006412;GO:0022625;GO:0003735;GO:0019843 |
| TRINITY_DN335617_c0_g1_i1_m.1598774 | cell division protein FtsA | 1.87 | 0.0055 | GO:0008360;GO:0043093;GO:0032153;GO:0009898;GO:0005524 |
| TRINITY_DN395945_c3_g1_i1_m.2147741 | CSC1 like protein HYP1 | 1.81 | 0.0001 | GO:0016021 |
| TRINITY_DN545404_c0_g1_i1_m.3737745 | phosphopyruvate hydratase | 1.79 | 0.0055 | GO:0006096;GO:0000015;GO:0009986;GO:0005576;GO:0000287;GO:0004634 |
| TRINITY_DN396497_c2_g1_i12_m.2064499 | 50S ribosomal protein L4 | 1.69 | 0.0080 | GO:0006412;GO:0005840;GO:0003735;GO:0019843 |
| TRINITY_DN378494_c1_g2_i11_m.2240885 | 30S ribosomal protein S3 | 1.90 | 0.0007 | GO:0006412;GO:0015935;GO:0003735;GO:0019843;GO:0003729 |
| TRINITY_DN389835_c5_g1_i4_m.2490728 | cold shock protein 1 | 1.68 | 0.0009 | GO:0006355;GO:0008270;GO:0003677 |
| TRINITY_DN389810_c1_g1_i9_m.2490247 | chitinase 2 like | 1.56 | 0.0008 | GO:0005975;GO:0006032;GO:0005576;GO:0004568;GO:0008061 |
| TRINITY_DN366503_c0_g1_i1_m.2637387 | 1 aminocyclopropane 1 carboxylate oxidase homolog 1 like | 1.88 | 0.0006 | GO:0046872;GO:0051213 |
| TRINITY_DN339110_c0_g1_i4_m.2960644 | endo 1,3;1,4 beta D glucanase like isoform X2 | 2.07 | 0.0042 | GO:0016787 |
| TRINITY_DN359412_c1_g1_i2_m.1989139 | F0F1 ATP synthase subunit B | 1.83 | 0.0459 | GO:0042777;GO:0016021;GO:0005886;GO:0045263;GO:0046933 |
| TRINITY_DN640026_c0_g1_i1_m.92338 | peptidylprolyl isomerase | 1.67 | 0.0002 | GO:0000413;GO:0006457;GO:0003755 |
| TRINITY_DN382154_c0_g1_i1_m.1368984 | cyanate hydratase | 1.83 | 0.0006 | GO:0009440;GO:0005829;GO:0008824;GO:0042802;GO:0003677 |

| | | | | |
|---|---|---|---|---|
| TRINITY_DN399772_c4_g2_i2_m.3141544 | Putative aldehyde oxidase 2 | 1.58 | 0.0030 | GO:0009115;GO:0055114;GO:0005829;GO:0050660;GO:0016614;GO:0051537;GO:0016903;GO:0004854;GO:0005506;GO:0009055 |
| TRINITY_DN393733_c0_g2_i1_m.3179912 | endoplasmic reticulum Golgi intermediate compartment protein 3 like | 1.34 | 0.0024 | GO:0016021 |
| TRINITY_DN370175_c2_g1_i9_m.1203295 | probable ribosomal protein L12 | 1.31 | 0.0240 | GO:0006888;GO:0006886;GO:0006412;GO:0005840;GO:0030127;GO:0003735;GO:0008270 |
| TRINITY_DN388455_c2_g1_i6_m.2466409 | NADH dehydrogenase subunit 1 | 1.38 | 0.0011 | GO:0006120;GO:0016021;GO:0005886;GO:0005747;GO:0008137 |
| TRINITY_DN275277_c0_g1_i2_m.4059257 | 60S ribosomal protein L10a | 1.40 | 0.0010 | GO:0006412;GO:0015934;GO:0003735;GO:0003723 |
| TRINITY_DN377479_c0_g1_i7_m.1098779 | Cytochrome c1 1, heme protein | 1.54 | 0.0000 | GO:0020037;GO:0009055 |
| TRINITY_DN393034_c3_g1_i12_m.1155792 | protein disulfide isomerase family protein 1 2 | 1.48 | 0.0087 | GO:0045454;GO:0034975;GO:0009960;GO:0005788;GO:0003756 |
| TRINITY_DN340678_c1_g1_i4_m.2751393 | protein NUCLEAR FUSION DEFECTIVE 2 | 1.63 | 0.0065 | GO:0090502;GO:0010197;GO:0006396;GO:0005773;GO:0004525;GO:0003725 |
| TRINITY_DN397828_c2_g1_i20_m.1951515 | UDP glycosyltransferase | 1.39 | 0.0345 | GO:0009813;GO:0052696;GO:0043231;GO:0080043;GO:0080044 |
| TRINITY_DN397828_c2_g1_i20_m.1951515 | Niemann Pick C1 protein isoform X1 | 1.39 | 0.0345 | GO:0009813;GO:0052696;GO:0043231;GO:0080043;GO:0080044 |
| TRINITY_DN378540_c1_g1_i8_m.3216452 | serine carboxypeptidase like 2 | 1.34 | 0.0231 | GO:0051603;GO:0019748;GO:0016747;GO:0004185 |
| TRINITY_DN240950_c0_g1_i1_m.3853985 | capsular biosynthesis protein | 1.37 | 0.0072 | GO:0015774;GO:0016020;GO:0015159 |
| TRINITY_DN380423_c1_g2_i3_m.1296074 | PIP aquaporin | 1.72 | 0.0041 | GO:0009992;GO:0034220;GO:0006833;GO:0015793;GO:0005773;GO:0005887;GO:0009506;GO:0015250;GO:0015254 |
| TRINITY_DN464903_c0_g1_i1_m.728505 | type 4 pili major subunit | 1.27 | 0.0102 | GO:0015628;GO:0007155;GO:0016021;GO:0015627;GO:0009289;GO:0008565 |
| TRINITY_DN385663_c1_g1_i7_m.3359872 | mitochondrial phosphate carrier protein 3 | 1.48 | 0.0008 | GO:0006810;GO:0006412;GO:0016021;GO:0005743;GO:0003735;GO:0005515 |
| TRINITY_DN397546_c1_g2_i11_m.2299064 | cycloartenol synthase | 1.32 | 0.0000 | GO:0005739;GO:0016871 |
| TRINITY_DN368437_c1_g1_i2_m.1419694 | prohibitin 3 | 1.27 | 0.0019 | GO:0016020 |
| TRINITY_DN398089_c1_g1_i6_m.947873 | Gamma glutamyltranspeptidase 1 | 1.29 | 0.0147 | GO:0006749;GO:0016021;GO:0003840 |
| TRINITY_DN384399_c0_g5_i2_m.2378298 | 50S ribosomal protein L19 | 1.96 | 0.0037 | GO:0006412;GO:0005840;GO:0003735 |
| TRINITY_DN350923_c7_g1_i8_m.1017031 | translation elongation factor 1 alpha | 3.43 | 0.0038 | GO:0006414;GO:0005737;GO:0003746;GO:0003924;GO:0005525 |
| TRINITY_DN321721_c0_g1_i2_m.3148171 | methionine adenosyltransferase | 4.38 | 0.0054 | GO:0006556;GO:0006730;GO:0005737;GO:0004478;GO:0000287;GO:0005524 |
| TRINITY_DN378496_c1_g1_i5_m.2242774 | inorganic pyrophosphatase | 3.08 | 0.0177 | GO:0006796;GO:0005737;GO:0005634;GO:0000287;GO:0004427 |
| TRINITY_DN371682_c2_g1_i3_m.2886792 | putative protease inhibitor | 3.10 | 0.0253 | GO:0010951;GO:0005576;GO:0004867 |
| TRINITY_DN223611_c0_g1_i1_m.3915565 | Citrate | 3.68 | 0.0008 | GO:0006099;GO:0005737;GO:0004108 |
| TRINITY_DN297451_c2_g1_i1_m.3825965 | amino acid ABC transporter substrate bindnig protein | 2.93 | 0.0173 | GO:0006810;GO:0030288;GO:0005215 |
| TRINITY_DN242970_c1_g1_i2_m.3943454 | lipoprotein | 3.17 | 0.0272 | GO:0043165;GO:0009279 |
| TRINITY_DN397057_c1_g3_i2_m.2506770 | 50S ribosomal protein L10 | 1.86 | 0.0079 | GO:0006412;GO:0042254;GO:0005840;GO:0003735;GO:0070180 |
| TRINITY_DN499678_c0_g1_i1_m.762711 | catalase HPII | 2.80 | 0.0354 | GO:0098869;GO:0042744;GO:0006979;GO:0055114;GO:0020037;GO:0004096;GO:0046872 |
| TRINITY_DN370287_c3_g1_i3_m.2480526 | 30S ribosomal protein S20 | 2.18 | 0.0165 | GO:0006412;GO:0005840;GO:0003735;GO:0019843 |
| TRINITY_DN382051_c2_g1_i20_m.3191987 | dehydrin 7 | 2.19 | 0.0019 | GO:0050896 |
| TRINITY_DN293295_c0_g1_i4_m.4046936 | general secretion pathway protein | 2.86 | 0.0014 | GO:0043107;GO:0016021;GO:0044096;GO:0009986;GO:0005829 |
| TRINITY_DN399318_c3_g2_i3_m.1325852 | 30S ribosomal protein S12 | 2.36 | 0.0001 | GO:0006412;GO:0015935;GO:0003735;GO:0000049;GO:0019843 |
| TRINITY_DN374186_c0_g1_i3_m.1697530 | myo inositol 1 phosphate synthase | 1.70 | 0.0060 | GO:0006021;GO:0008654;GO:0005737;GO:0004512 |
| TRINITY_DN395872_c1_g1_i2_m.1523351 | endoplasmic reticulum oxidoreductin 1 like | 1.46 | 0.0298 | GO:0051604;GO:0055114;GO:0009536;GO:0016021;GO:0005789;GO:0016671;GO:0003756 |
| TRINITY_DN308699_c0_g1_i3_m.1042511 | GTP binding protein ypt1 | 1.63 | 0.0229 | GO:0016236;GO:0070317;GO:0032258;GO:0048194;GO:0090114;GO:1990261;GO:0006461;GO:0048211;GO:0007131;GO:0007264;GO:0034498;GO:0006890;GO:0000730;GO:0032456;GO:1900101;GO:0035494;GO:0005795;GO:0005789;GO:0031410;GO:0005739;GO:0000407;GO:0035861;GO:0005829;GO:0005802;GO:0000139;GO:0005634;GO:0003677;GO:0000149;GO:0005524;GO:0005525;GO:0003924;GO:0008094 |
| TRINITY_DN396659_c1_g1_i3_m.2180219 | alpha mannosidase 2 | 1.24 | 0.0015 | GO:0042538;GO:0006491;GO:0006013;GO:0006517;GO:0009536;GO:0016021;GO:0005802;GO:0000139;GO:0005768;GO:0030246;GO:0008270;GO:0004559 |

| | | | | |
|---|---|---|---|---|
| TRINITY_DN332233_c0_g1_i1_m.1685212 | protein YLS9 like | 1.30 | 0.0310 | GO:0006952;GO:0007165;GO:0016021;GO:0046658;GO:0009506;GO:0004871 |
| TRINITY_DN363148_c0_g1_i1_m.2052021 | tobamovirus multiplication protein 2A | 1.39 | 0.0039 | GO:0016021 |
| TRINITY_DN383646_c3_g1_i3_m.3249952 | transmembrane 9 superfamily member 8 like | 1.54 | 0.0044 | GO:0016021 |
| TRINITY_DN378030_c0_g4_i1_m.3114353 | germin like protein | 1.63 | 0.0463 | GO:0033609;GO:0005618;GO:0005576;GO:0030145;GO:0045735;GO:0046564 |
| TRINITY_DN399065_c2_g1_i14_m.2654090 | glutamate synthase 1 [NADH] | 1.42 | 0.0358 | GO:0019676;GO:0048589;GO:0097054;GO:0060359;GO:0055114;GO:0009507;GO:0050660;GO:0051538;GO:0010181;GO:0016040;GO:0005506 |
| TRINITY_DN379065_c2_g3_i12_m.1429421 | class IV endochitinase | 1.85 | 0.0131 | GO:0006032;GO:0005975;GO:0016998;GO:0004568;GO:0008061 |
| TRINITY_DN398406_c4_g1_i4_m.2869809 | 50S ribosomal protein L4 | 1.89 | 0.0001 | GO:0006412;GO:0005840;GO:0003735;GO:0019843 |
| TRINITY_DN345321_c0_g2_i4_m.1612778 | 30S ribosomal protein S2 | 2.08 | 0.0004 | GO:0006412;GO:0015935;GO:0003735 |
| TRINITY_DN306866_c1_g2_i4_m.2981091 | GMP synthetase | 1.63 | 0.0056 | GO:0006177;GO:0006541;GO:0016462;GO:0003922;GO:0005524 |
| TRINITY_DN391155_c2_g1_i11_m.2103034 | elongation factor Tu | 1.98 | 0.0084 | GO:0006414;GO:0005737;GO:0003746;GO:0003924;GO:0005525 |
| TRINITY_DN237908_c0_g2_i1_m.4108644 | pyruvate dehydrogenase | 1.54 | 0.0291 | GO:0055114;GO:0004739 |
| TRINITY_DN395217_c2_g2_i4_m.1370521 | germin like protein 8 11 isoform X2 | 1.43 | 0.0261 | GO:0033609;GO:0005618;GO:0048046;GO:0030145;GO:0045735;GO:0046564 |
| TRINITY_DN391957_c1_g1_i9_m.1853404 | high affinity nitrate transporter activating protein 2.1 like | 1.64 | 0.0009 | GO:0010167;GO:0015706;GO:0016021 |
| TRINITY_DN390441_c2_g1_i1_m.3226047 | non functional NADPH dependent codeinone reductase 2 like | 1.28 | 0.0342 | GO:0055114;GO:0005739;GO:0016491 |
| TRINITY_DN776893_c0_g1_i1_m.4181332 | trigger factor | 1.40 | 0.0062 | GO:0000413;GO:0006457;GO:0051301;GO:0015031;GO:0007049;GO:0005737;GO:0003755 |
| TRINITY_DN375087_c1_g1_i1_m.1930439 | Sterol 3 beta glucosyltransferase | 1.59 | 0.0116 | GO:0010214;GO:0016125;GO:0052696;GO:0009813;GO:0009845;GO:0030259;GO:0055114;GO:0005774;GO:0008270;GO:0016906;GO:0016491 |
| TRINITY_DN375176_c1_g3_i1_m.3262970 | small nuclear ribonucleoprotein Sm D1 | 1.56 | 0.0092 | GO:0000387;GO:0000245;GO:0000243;GO:0071011;GO:0071010;GO:0071013;GO:0034715;GO:0097526;GO:0005689;GO:0005687;GO:0005686;GO:0005685;GO:0034719;GO:0005682;GO:0003723 |
| TRINITY_DN170506_c0_g1_i2_m.525141 | peptidoglycan associated lipoprotein | 1.39 | 0.0227 | GO:0016021;GO:0009279 |
| TRINITY_DN393649_c1_g1_i6_m.1032397 | DNA directed RNA polymerases I and III subunit rpac1 | 1.20 | 0.0150 | GO:0006360;GO:0032259;GO:0006383;GO:0005736;GO:0005666;GO:0001056;GO:0001054;GO:0003677;GO:0008168;GO:0046983 |
| TRINITY_DN361841_c0_g1_i5_m.1571518 | TP dependent RNA helicase p62 isoform X1 | 1.87 | 0.0164 | GO:0005524;GO:0003676;GO:0004386 |
| TRINITY_DN361841_c0_g1_i5_m.1571518 | ATP dependent RNA helicase p62 isoform X1 | 1.87 | 0.0164 | GO:0005524;GO:0003676;GO:0004386 |
| TRINITY_DN345672_c0_g1_i5_m.872963 | AF112964_1 small GTP binding protein | 1.40 | 0.0138 | GO:0007264;GO:0030139;GO:0016021;GO:0005768;GO:0005525 |
| TRINITY_DN371588_c0_g1_i7_m.1647514 | development related protein kinase | 1.36 | 0.0340 | GO:0006468;GO:0035556;GO:0005737;GO:0005634;GO:0004674;GO:0005524 |
| TRINITY_DN397983_c0_g1_i2_m.2061080 | Voltage dependent anion selective channel protein | 1.68 | 0.0001 | GO:0009058;GO:1903959;GO:0046930;GO:0005741;GO:0015288;GO:0008308 |
| TRINITY_DN25779_c0_g1_i1_m.4548655 | succinate dehydrogenase iron sulfur subunit [Pseudomonas fluorescens] | 1.62 | 0.0003 | GO:0006099;GO:0051536;GO:0008177;GO:0009055 |
| TRINITY_DN863545_c0_g1_i1_m.3405861 | aminopeptidase | 1.97 | 0.0022 | GO:0006508;GO:0004177 |
| TRINITY_DN389448_c0_g6_i1_m.1814443 | 30S ribosomal protein S4 | 1.49 | 0.0320 | GO:0006412;GO:0015935;GO:0003735;GO:0019843 |
| TRINITY_DN138746_c0_g1_i1_m.555730 | ribosomal subunit interface protein | 1.73 | 0.0001 | GO:0044238 |
| TRINITY_DN382319_c2_g2_i2_m.2695576 | gamma carbonic anhydrase 2 | 1.72 | 0.0002 | GO:0070207;GO:2000377;GO:0009901;GO:0005747;GO:0009507;GO:0042802 |
| TRINITY_DN387645_c2_g3_i6_m.3109112 | putative protease Do like 14 isoform X1 | 1.40 | 0.0018 | GO:0006508;GO:0004252 |
| TRINITY_DN391530_c4_g4_i5_m.2833377 | ATP carrier protein 1 | 1.64 | 0.0008 | GO:0006412;GO:0055085;GO:0016021;GO:0005743;GO:0005215;GO:0003735 |
| TRINITY_DN395120_c0_g1_i9_m.2846159 | putative amidase AmiD isoform X2 | 1.28 | 0.0044 | GO:0006891;GO:0006487;GO:0006635;GO:0006869;GO:0016558;GO:0010351;GO:0005622;GO:0016740;GO:0016884 |
| TRINITY_DN390261_c2_g1_i3_m.2367284 | receptor protein kinase TMK1 like | 1.48 | 0.0001 | GO:0006468;GO:0005739;GO:0016021;GO:0004674;GO:0005524 |
| TRINITY_DN366217_c3_g1_i8_m.2500510 | glutathione S transferase 3 | 1.22 | 0.0186 | GO:0006749;GO:0005737;GO:0004364 |
| TRINITY_DN378428_c0_g1_i3_m.2243308 | allene oxide synthase 4 | 1.38 | 0.0122 | GO:0031408;GO:0016125;GO:0055114;GO:0020037;GO:0016705;GO:0004497;GO:0047987;GO:0005506 |
| TRINITY_DN171808_c1_g1_i1_m.432874 | elongation factor 2 | 1.70 | 0.0271 | GO:0006414;GO:0003746;GO:0003924;GO:0005525 |

| | | | | |
|---|---|---|---|---|
| TRINITY_DN390648_c3_g2_i9_m.1393228 | dihydrolipoyllysine residue acetyltransferase component 1 of pyruvate dehydrogenase complex | 1.45 | 0.0034 | GO:0006090;GO:0045254;GO:0005759;GO:0004742 |
| TRINITY_DN386922_c2_g1_i3_m.2667615 | class III peroxidase | 1.66 | 0.0039 | GO:0098869;GO:0042744;GO:0006979;GO:0055114;GO:0005576;GO:0020037;GO:0004601;GO:0046872 |
| TRINITY_DN395470_c1_g1_i8_m.2730616 | Arginine decarboxylase | 1.37 | 0.0174 | GO:0033388;GO:0006527;GO:0008295;GO:0008792 |
| TRINITY_DN283322_c0_g1_i2_m.3926355 | UTP glucose 1 phosphate uridylyltransferase isoform X1 | 1.40 | 0.0076 | GO:0006011;GO:0003983 |
| TRINITY_DN397549_c1_g2_i6_m.2299926 | probable calcium transporting ATPase 6 | 1.23 | 0.0120 | GO:0070588;GO:0043231;GO:0005887;GO:0005516;GO:0005388;GO:0046872;GO:0005524 |
| TRINITY_DN397454_c2_g2_i5_m.1055708 | carotenoid 9,1 | 1.24 | 0.0053 | GO:0016124;GO:0016121;GO:0055114;GO:0005794;GO:0016021;GO:0005774;GO:0005886;GO:0009506;GO:0045549 |
| TRINITY_DN383256_c0_g2_i6_m.3054025 | Acyl CoA binding domain containing protein 4 | 1.31 | 0.0006 | GO:0000062 |
| TRINITY_DN388744_c0_g2_i9_m.2537201 | Lactoylglutathione lyase | 1.48 | 0.0006 | GO:0046872;GO:0004462 |
| TRINITY_DN399943_c11_g1_i3_m.1625950 | ABC transporter C family member 3 | 1.24 | 0.0141 | GO:0045454;GO:0055085;GO:0055114;GO:0043190;GO:0042626;GO:0005524;GO:0015035;GO:0009055 |
| TRINITY_DN387949_c0_g1_i1_m.1827067 | glycosyl hydrolase family 10 | 1.47 | 0.0209 | GO:0009405;GO:0045493;GO:0005615;GO:0031176 |
| TRINITY_DN377881_c0_g1_i2_m.1085700 | Transmembrane 9 superfamily member 4 | 1.35 | 0.0006 | GO:0016021;GO:0005802;GO:0005768;GO:0009506;GO:0005774 |
| TRINITY_DN384309_c3_g1_i1_m.2379487 | exodeoxyribonuclease III | 1.35 | 0.0021 | GO:0006284;GO:0005634;GO:0003906;GO:0005488;GO:0008311 |
| TRINITY_DN354341_c0_g1_i10_m.2231930 | calcium transporting ATPase 8 | 1.49 | 0.0023 | GO:0070588;GO:0043231;GO:0005887;GO:0005516;GO:0005388;GO:0046872;GO:0005524 |
| TRINITY_DN372518_c3_g2_i1_m.2762227 | cyclophilin B D | 1.41 | 0.0177 | GO:0000413;GO:0006457;GO:0005795;GO:0005783;GO:0005771;GO:0016020;GO:0005829;GO:0003755 |
| TRINITY_DN133778_c0_g1_i1_m.474029 | nucleotide exchange factor GrpE | 1.38 | 0.0378 | GO:0050790;GO:0006457;GO:0005737;GO:0051087;GO:0000774;GO:0042803 |
| TRINITY_DN398652_c1_g1_i6_m.2659114 | ER membrane protein complex subunit 7 homolog | 1.35 | 0.0016 | GO:0016021;GO:0030246 |
| TRINITY_DN394952_c1_g1_i6_m.2428788 | Receptor like protein kinase 2 | 1.29 | 0.0117 | GO:0006468;GO:0052696;GO:0009813;GO:0043231;GO:0016021;GO:0004672;GO:0016758;GO:0008194;GO:0005524 |
| TRINITY_DN382832_c0_g1_i6_m.2541523 | probable mitochondrial saccharopine dehydrogenase like oxidoreductase At5g39410 | 1.22 | 0.0005 | GO:0055114;GO:0009941;GO:0016021;GO:0005739;GO:0005774;GO:0005886;GO:0016491 |
| TRINITY_DN398531_c1_g2_i3_m.1123989 | PTI1 like tyrosine protein kinase 3 | 1.32 | 0.0397 | GO:0018108;GO:0004713;GO:0005524 |
| TRINITY_DN369004_c0_g3_i1_m.3150683 | cerevisin | 1.26 | 0.0284 | GO:0006508;GO:0004252 |
| TRINITY_DN380423_c1_g1_i1_m.1296055 | putative PIP type aquaporin | 1.76 | 0.0231 | GO:0009992;GO:0034220;GO:0006833;GO:0015793;GO:0005773;GO:0005887;GO:0009506;GO:0015250;GO:0015254 |
| TRINITY_DN368681_c1_g2_i2_m.1040120 | GST | 1.33 | 0.0305 | GO:0006749;GO:0009407;GO:0005737;GO:0004364 |
| TRINITY_DN370625_c0_g1_i2_m.2216853 | peroxisome biogenesis protein 7 | 1.31 | 0.0095 | GO:0006625;GO:0005622 |
| TRINITY_DN395565_c1_g2_i3_m.3046310 | subtilisin protease | 1.22 | 0.0112 | GO:0006508;GO:0005618;GO:0004252 |
| TRINITY_DN395089_c0_g1_i4_m.2278264 | cystathionine beta lyase | 1.20 | 0.0133 | GO:0000394;GO:0019279;GO:0009536;GO:0004121;GO:0030170 |
| TRINITY_DN364637_c0_g1_i2_m.2316710 | vesicle associated membrane 1 3 like protein | 1.35 | 0.0213 | GO:0016021;GO:0005789 |
| TRINITY_DN373681_c1_g1_i2_m.1756631 | ubiquitin carboxyl terminal hydrolase 12 like isoform X1 | 1.36 | 0.0195 | GO:0006511;GO:0016579;GO:0036459 |
| TRINITY_DN387596_c0_g1_i1_m.2261520 | long chain alcohol O fatty acyltransferase like | 1.24 | 0.0336 | GO:0016021;GO:0008374 |
| TRINITY_DN168674_c0_g1_i1_m.598604 | proteasome subunit alpha type 3 | 1.22 | 0.0284 | GO:0010499;GO:0043161;GO:0019773;GO:0005634;GO:0034515;GO:0004298;GO:0003729 |
| TRINITY_DN385576_c3_g1_i2_m.2336706 | Stilbene synthase 2 | 1.23 | 0.0288 | GO:0006012;GO:0009813;GO:0008108;GO:0016747;GO:0042802 |
| TRINITY_DN375639_c3_g1_i4_m.1957212 | 30S ribosomal protein S9 | 3.39 | 0.0114 | GO:0006412;GO:0022627;GO:0003735 |
| TRINITY_DN385803_c0_g1_i15_m.1142393 | delta tonoplast intrinsic protein TIP2;2 | 2.59 | 0.0006 | GO:0009992;GO:0007030;GO:0006816;GO:0072489;GO:0006833;GO:0015793;GO:0009651;GO:0009941;GO:0005794;GO:0000326;GO:0009705;GO:0005887;GO:0042807;GO:0009505;GO:0009506;GO:0015200;GO:0015250;GO:0015254 |
| TRINITY_DN845716_c0_g1_i1_m.3374669 | serine hydroxymethyltransferase | 2.11 | 0.0031 | GO:0035999;GO:0019264;GO:0032259;GO:0005737;GO:0004372;GO:0030170;GO:0008168 |
| TRINITY_DN335247_c0_g1_i3_m.1543467 | branched chain amino acid ABC transporter substrate binding | 2.49 | 0.0132 | GO:0016021 |

| | | | | |
|---|---|---|---|---|
| | protein | | | |
| TRINITY_DN368209_c0_g1_i2_m.1447816 | 60S acidic ribosomal protein P2B | 3.05 | 0.0062 | GO:0002181;GO:0006414;GO:0022625;GO:0030687;GO:0070180;GO:0003735 |
| TRINITY_DN142821_c0_g1_i1_m.583280 | polyamine ABC transporter substrate binding protein | 2.84 | 0.0250 | GO:0015846;GO:0042597;GO:0019808 |
| TRINITY_DN180129_c0_g1_i1_m.600510 | two component system response regulator | 2.84 | 0.0164 | GO:0000160;GO:0005622;GO:0003677 |
| TRINITY_DN389710_c1_g1_i1_m.993434 | sugar transport protein 13 like | 1.77 | 0.0198 | GO:0015992;GO:0046323;GO:1904659;GO:0005887;GO:0005355;GO:0005351 |
| TRINITY_DN84984_c0_g1_i1_m.4487465 | phage tail protein | 2.00 | 0.0059 | GO:0005198 |
| TRINITY_DN317547_c0_g1_i2_m.1476287 | D 3 phosphoglycerate dehydrogenase | 3.00 | 0.0001 | GO:0006564;GO:0055114;GO:0051287;GO:0004617;GO:0016597 |
| TRINITY_DN350813_c0_g1_i5_m.2547938 | 50S ribosomal protein L28 | 2.69 | 0.0052 | GO:0006412;GO:0005840;GO:0003735 |
| TRINITY_DN215564_c0_g1_i3_m.3775699 | ATP dependent metalloprotease | 1.59 | 0.0366 | GO:0051301;GO:0030163;GO:0006508;GO:0016021;GO:0005886;GO:0004222;GO:0016887;GO:0005524;GO:0008270 |
| TRINITY_DN380876_c1_g3_i3_m.1351369 | ATP dependent 6 phosphofructokinase 6 like | 1.93 | 0.0060 | GO:0006002;GO:0006508;GO:0061615;GO:0005737;GO:0004222;GO:0003872;GO:0046872;GO:0005524 |
| TRINITY_DN332695_c1_g4_i1_m.1944361 | molecular chaperone GroEL | 2.11 | 0.0003 | GO:0042026;GO:0005737;GO:0051082;GO:0005524 |
| TRINITY_DN62801_c0_g1_i1_m.4470172 | nucleoside diphosphate kinase | 1.54 | 0.0149 | GO:0006228;GO:0006241;GO:0006165;GO:0006183;GO:0005737;GO:0004550;GO:0005524;GO:0046872 |
| TRINITY_DN392960_c6_g2_i2_m.2522375 | 1,3 beta glucanase | 1.46 | 0.0386 | GO:0005975;GO:0042973 |
| TRINITY_DN386027_c2_g1_i4_m.2970640 | 60S ribosomal protein L11 | 1.83 | 0.0037 | GO:0006412;GO:0005840;GO:0003735 |
| TRINITY_DN383618_c0_g1_i4_m.3250740 | ABC transporter B family member 4 | 1.47 | 0.0130 | GO:0055085;GO:0016021;GO:0042626;GO:0005524 |
| TRINITY_DN178004_c0_g1_i2_m.403651 | succinate dehydrogenase | 1.50 | 0.0077 | GO:0022900;GO:0006099;GO:0005886;GO:0050660;GO:0008177 |
| TRINITY_DN380124_c1_g1_i5_m.3329369 | UDP arabinose mutase 1 | 1.56 | 0.0006 | GO:0009832;GO:0030244;GO:0071555;GO:0033356;GO:0005794;GO:0005618;GO:0005829;GO:0005576;GO:0009506;GO:0052691;GO:0005515;GO:0016757 |
| TRINITY_DN363751_c0_g2_i1_m.3273283 | NADH dehydrogenase [ubiquinone] 1 beta subcomplex subunit 7 | 1.69 | 0.0000 | GO:0055114;GO:0009536;GO:0005739;GO:0008137 |
| TRINITY_DN375995_c0_g2_i1_m.1102879 | mitochondrial import receptor subunit TOM40 1 like | 1.57 | 0.0016 | GO:0030150;GO:0005742;GO:0015266 |
| TRINITY_DN389416_c0_g1_i2_m.1817008 | pathogen related protein | 1.63 | 0.0037 | GO:0006952;GO:0009607 |
| TRINITY_DN382318_c1_g2_i1_m.2699240 | probable pyridoxal 5' phosphate synthase subunit PDX1.1 | 1.49 | 0.0013 | GO:0042823;GO:0042819;GO:0003824 |
| TRINITY_DN379329_c0_g2_i4_m.1905873 | aarF domain containing protein kinase 4 | 1.46 | 0.0075 | GO:0005739 |
| TRINITY_DN319627_c0_g1_i1_m.2442470 | PRA1 family protein B2 | 1.47 | 0.0003 | GO:0016021;GO:0009536 |
| TRINITY_DN387648_c1_g1_i11_m.3106792 | cytochrome b561 and DOMON domain containing protein At4g17280 like | 1.34 | 0.0160 | GO:0055114;GO:0016021 |
| TRINITY_DN363982_c0_g1_i11_m.2693155 | Proactivator polypeptide | 1.55 | 0.0016 | GO:0043085;GO:0006629;GO:0005737;GO:0001664;GO:0008047 |
| TRINITY_DN358760_c4_g1_i3_m.3015694 | 40S ribosomal protein S2 3 like | 1.27 | 0.0389 | GO:0045903;GO:0006407;GO:0032040;GO:0022627;GO:0070181;GO:0003735 |
| TRINITY_DN387293_c0_g1_i12_m.1306299 | lipoamide acyltransferase component of branched chain alpha keto acid dehydrogenase complex | 1.44 | 0.0018 | GO:0009750;GO:0008152;GO:0005739;GO:0016407;GO:0008270;GO:0004147 |
| TRINITY_DN398902_c0_g3_i3_m.1561414 | ferritin 2A | 1.58 | 0.0009 | GO:0006879;GO:0006826;GO:0055114;GO:0005623;GO:0004322;GO:0008199 |
| TRINITY_DN385803_c0_g1_i16_m.1142396 | Aquaporin TIP2 3 | 1.52 | 0.0013 | GO:0009992;GO:0034220;GO:0006833;GO:0015793;GO:0009705;GO:0005887;GO:0042807;GO:0015250;GO:0015254 |
| TRINITY_DN377811_c0_g1_i13_m.1087158 | Arginine/serine rich splicing factor RSP31 | 1.27 | 0.0487 | GO:0003676;GO:0000166 |
| TRINITY_DN383649_c0_g1_i12_m.3250829 | Uricase | 1.36 | 0.0087 | GO:0007031;GO:0006635;GO:0019628;GO:0006144;GO:0005777;GO:0005739;GO:0004846 |
| TRINITY_DN17000_c0_g1_i1_m.4372993 | cytochrome c oxidase subunit 5C 2 | 1.60 | 0.0009 | GO:0016021;GO:0005746;GO:0005634 |
| TRINITY_DN394626_c0_g1_i14_m.1462836 | Histone acetyltransferase GCN5 | 1.30 | 0.0000 | GO:0006355;GO:0016573;GO:0005634;GO:0009536;GO:0004402 |
| TRINITY_DN399135_c3_g1_i5_m.910882 | ammonium transporter | 1.28 | 0.0239 | GO:0019740;GO:0015695;GO:0072488;GO:0005887;GO:0008519 |
| TRINITY_DN382620_c1_g1_i5_m.920656 | nuclear transcription factor Y subunit C 4 like | 1.49 | 0.0234 | GO:0005634;GO:0046982 |
| TRINITY_DN387970_c0_g1_i8_m.1827634 | 3 oxoacyl [acyl carrier protein] synthase I/Beta ketoacyl ACP synthase I | 1.49 | 0.0018 | GO:0006633;GO:0009507;GO:0004315 |

| | | | | |
|---|---|---|---|---|
| TRINITY_DN386748_c1_g1_i1_m.2814509 | heme oxygenase 1 | 1.47 | 0.0017 | GO:0048573;GO:0006788;GO:0010229;GO:0015979;GO:0009507;GO:0004392;GO:0003700;GO:0046872 |
| TRINITY_DN397654_c2_g1_i1_m.3162616 | cobalamin independent methionine synthase | 1.26 | 0.0202 | GO:0032259;GO:0050667;GO:0009086;GO:0005829;GO:0005576;GO:0008705;GO:0008270;GO:0003871 |
| TRINITY_DN397374_c0_g1_i11_m.2210866 | Rhomboid family member 1 | 1.23 | 0.0033 | GO:0016485;GO:0016021;GO:0004252 |
| TRINITY_DN368529_c1_g1_i8_m.1506948 | Mn superoxide dismutase | 1.22 | 0.0145 | GO:0019430;GO:0055114;GO:0016021;GO:0046872;GO:0004784 |
| TRINITY_DN394817_c0_g1_i24_m.1746220 | probable acyl CoA dehydrogenase IBR3 isoform X3 | 1.30 | 0.0219 | GO:0048767;GO:0033539;GO:0009610;GO:0055088;GO:0050660;GO:0052890;GO:0000062;GO:0003995;GO:0009055 |
| TRINITY_DN372114_c0_g1_i4_m.2098663 | isoaspartyl peptidase/L asparaginase 1 | 1.26 | 0.0043 | GO:0005829;GO:0016787 |
| TRINITY_DN391529_c0_g1_i1_m.2833717 | protein TRIGALACTOSYLDIACYLGLYCEROL 3 | 1.28 | 0.0346 | GO:0008152;GO:0006869;GO:0009507;GO:0005524;GO:0016887 |
| TRINITY_DN378840_c1_g1_i2_m.3294150 | nitrilase like protein 2 | 1.24 | 0.0065 | GO:0006807;GO:0009536;GO:0016810 |
| TRINITY_DN392975_c1_g1_i2_m.2524776 | E3 ubiquitin protein ligase At1g12760 like | 1.56 | 0.0206 | GO:0043161;GO:0016567;GO:0009536;GO:0016021;GO:0008270;GO:0061630 |
| TRINITY_DN393724_c0_g1_i20_m.3181807 | protein fluG | 1.21 | 0.0471 | GO:0000723;GO:0010311;GO:0009737;GO:0048829;GO:0006281;GO:0006542;GO:0032508;GO:0005829;GO:0008017;GO:0004356;GO:0005524;GO:0003678;GO:0043015;GO:0043621 |
| TRINITY_DN391653_c2_g2_i1_m.3213098 | PRA1 family protein F3 | 1.25 | 0.0236 | GO:0016021;GO:0009536 |
| TRINITY_DN330198_c0_g1_i1_m.3256080 | probable RPL17A ribosomal protein L17.e | 5.83 | 0.0062 | GO:0006412;GO:0015934;GO:0003735 |
| TRINITY_DN399492_c1_g1_i9_m.1284618 | 30S ribosomal protein S5 | 5.32 | 0.0001 | GO:0006412;GO:0015935;GO:0003735;GO:0019843 |
| TRINITY_DN307235_c0_g1_i1_m.2821824 | 60 kDa chaperonin、GroEL protein，Heat shock protein 60 | 4.20 | 0.0061 | GO:0042026;GO:0005737;GO:0051082;GO:0005524 |
| TRINITY_DN371389_c3_g1_i4_m.2330238 | sodium coupled neutral amino acid transporter 1 like | 3.74 | 0.0092 | GO:0003333;GO:0016021;GO:0015171 |
| TRINITY_DN377371_c0_g1_i4_m.2690183 | serine/threonine protein kinase STY46 | 2.00 | 0.0039 | GO:0006468;GO:0009536;GO:0016597;GO:0005524;GO:0004672 |
| TRINITY_DN511611_c0_g1_i1_m.3684712 | BMP family ABC transporter substrate binding protein | 2.06 | 0.0015 | GO:0005886 |
| TRINITY_DN376098_c4_g1_i1_m.3352493 | 40S ribosomal protein S6 | 2.41 | 0.0007 | GO:0006412;GO:0005840;GO:0003735 |
| TRINITY_DN372752_c4_g1_i9_m.3119834 | calmodulin | 3.54 | 0.0186 | GO:0007264;GO:0005622;GO:0005509;GO:0005525 |
| TRINITY_DN541743_c0_g1_i1_m.3677158 | formate dehydrogenase O, Fe S subunit | 2.00 | 0.0044 | GO:0045333;GO:0015944;GO:0016021;GO:0046872;GO:0051539 |
| TRINITY_DN345055_c1_g1_i1_m.2108365 | succinate CoA ligase subunit alpha | 2.00 | 0.0003 | GO:0008152;GO:0048037;GO:0004775;GO:0016746;GO:0005524 |
| TRINITY_DN239575_c0_g1_i3_m.3967693 | ribonuclease E | 2.03 | 0.0016 | GO:0090502;GO:0008033;GO:0006402;GO:0006364;GO:0005737;GO:0009898;GO:0008995;GO:0004521;GO:0000287;GO:0008270;GO:0003723 |
| TRINITY_DN383910_c0_g1_i2_m.2786394 | acid phosphatase 1 | 1.95 | 0.0009 | GO:0016311;GO:0005739;GO:0003993 |
| TRINITY_DN290027_c1_g1_i4_m.3896828 | Porin D precursor | 1.96 | 0.0344 | GO:0055085;GO:0016021;GO:0015288 |
| TRINITY_DN378494_c1_g2_i1_m.2240831 | 50S ribosomal protein L23 | 1.95 | 0.0009 | GO:0006412;GO:0005840;GO:0003735;GO:0000166;GO:0019843 |
| TRINITY_DN224720_c0_g1_i1_m.4157158 | porin | 2.16 | 0.0380 | GO:0055085;GO:0016021;GO:0015288 |
| TRINITY_DN274183_c0_g1_i1_m.3911519 | ATP synthase, subunit E | 1.78 | 0.0187 | GO:0015991;GO:0033178;GO:0046961 |
| TRINITY_DN393062_c0_g1_i2_m.1151980 | short chain enoyl CoA hydratase | 1.60 | 0.0346 | GO:0008152;GO:0009536;GO:0005777;GO:0016853 |
| TRINITY_DN360717_c1_g8_i1_m.1664836 | mitochondrial import receptor subunit TOM7 1 like | 1.72 | 0.0040 | GO:0030150;GO:0005742;GO:0016021 |
| TRINITY_DN394030_c0_g1_i11_m.898405 | Peroxidase 2 | 1.67 | 0.0114 | GO:0009664;GO:0006979;GO:0098869;GO:0042744;GO:0055114;GO:0005576;GO:0009505;GO:0020037;GO:0046872;GO:0004601 |
| TRINITY_DN392109_c1_g1_i9_m.2200573 | probable trehalase | 1.57 | 0.0285 | GO:0005993;GO:0005886;GO:0004555 |
| TRINITY_DN381126_c0_g2_i8_m.2747832 | coiled coil domain containing protein 90B | 1.74 | 0.0000 | GO:0016021;GO:0005739 |
| TRINITY_DN599182_c0_g1_i1_m.3669002 | glutamate dehydrogenase | 1.82 | 0.0013 | GO:0006520;GO:0055114;GO:0004353 |
| TRINITY_DN357843_c0_g1_i3_m.3314007 | putative carboxylesterase 15 | 2.16 | 0.0051 | GO:0008152;GO:0016787 |
| TRINITY_DN317321_c0_g1_i2_m.2780888 | elongation factor G | 1.52 | 0.0492 | GO:0006414;GO:0005737;GO:0003746;GO:0003924;GO:0005525 |
| TRINITY_DN369253_c1_g1_i6_m.2472066 | Serpin ZX | 1.70 | 0.0000 | GO:0010951;GO:0005615;GO:0004867 |
| TRINITY_DN183748_c0_g1_i1_m.415686 | | 1.60 | 0.0038 | GO:0000160;GO:0006810;GO:0030288;GO:0005622 |
| TRINITY_DN96575_c0_g2_i1_m.4510205 | ubiquinol cytochrome c reductase iron sulfur subunit | 1.66 | 0.0006 | GO:1902600;GO:0055114;GO:0016021;GO:0051537;GO:0008121;GO:0046872 |

| | | | | |
|---|---|---|---|---|
| TRINITY_DN396711_c1_g1_i5_m.964336 | glutathione reductase | 1.70 | 0.0005 | GO:0009658;GO:0006626;GO:0098869;GO:0022900;GO:0006749;GO:0048481;GO:0045454;GO:0009570;GO:0005739;GO:0050661;GO:0050660;GO:0004362;GO:0005507;GO:0005524 |
| TRINITY_DN398923_c2_g3_i2_m.1280142 | Dihydrolipoyllysine residue acetyltransferase component of pyruvate dehydrogenase complex | 1.47 | 0.0007 | GO:0008152;GO:0009536;GO:0016746 |
| TRINITY_DN385729_c1_g2_i8_m.1145747 | phosphate transporter 6 | 1.58 | 0.0077 | GO:0006817;GO:0055085;GO:0005887;GO:0022891;GO:0005315 |
| TRINITY_DN396160_c0_g2_i22_m.3077944 | Mitochondrial uncoupling protein 3 | 1.46 | 0.0014 | GO:0006355;GO:0006839;GO:0006412;GO:0016021;GO:0005743;GO:0005634;GO:0043565;GO:0003700;GO:0005215;GO:0003735 |
| TRINITY_DN395505_c1_g5_i2_m.2728656 | NADH dehydrogenase subunit 2 | 1.51 | 0.0001 | GO:0042773;GO:0016021;GO:0070469;GO:0005743;GO:0008137 |
| TRINITY_DN388966_c0_g1_i1_m.1606215 | mitochondrial outer membrane protein porin 6 | 1.43 | 0.0023 | GO:1903959;GO:0046930;GO:0005741;GO:0005886;GO:0015288;GO:0008308 |
| TRINITY_DN377851_c0_g2_i3_m.1085480 | protein RETICULATA RELATED 4 | 1.45 | 0.0025 | GO:0009706;GO:0005739 |
| TRINITY_DN373516_c1_g1_i1_m.1435596 | endoglucanase type F | 1.80 | 0.0175 | GO:0030245;GO:0045493;GO:0005576;GO:0030248;GO:0008810;GO:0031176 |
| TRINITY_DN391357_c0_g1_i1_m.2958120 | pullulanase 1 | 1.55 | 0.0001 | GO:0005975;GO:0046872;GO:0051060 |
| TRINITY_DN387192_c0_g3_i5_m.1005256 | pyridoxal kinase | 1.52 | 0.0001 | GO:0016310;GO:0042816;GO:0009443;GO:0005829;GO:0008478 |
| TRINITY_DN382773_c0_g1_i6_m.3063958 | STE24 | 1.35 | 0.0003 | GO:0071586;GO:0005773;GO:0016021;GO:0005789;GO:0004222;GO:0046872 |
| TRINITY_DN391531_c1_g1_i12_m.2835218 | Dihydrolipoyllysine residue acetyltransferase component 3 of pyruvate dehydrogenase complex | 1.60 | 0.0003 | GO:0006090;GO:0045254;GO:0005759;GO:0004742 |
| TRINITY_DN380106_c1_g1_i14_m.3328866 | transmembrane emp24 domain containing protein p24delta3 like | 1.46 | 0.0003 | GO:0006810;GO:0016021;GO:0005789 |
| TRINITY_DN383719_c1_g11_i1_m.1705252 | prohibitin 1 | 1.48 | 0.0022 | GO:0016020 |
| TRINITY_DN378892_c0_g2_i5_m.3295662 | putative zinc transporter | 1.34 | 0.0058 | GO:0010167;GO:0015706;GO:0035434;GO:0006826;GO:0071577;GO:0010106;GO:0016021;GO:0005886;GO:0005385;GO:0005375;GO:0016491 |
| TRINITY_DN389248_c1_g1_i1_m.3171630 | serpin ZX like | 1.61 | 0.0007 | GO:0010951;GO:0016021;GO:0005615;GO:0004867 |
| TRINITY_DN396880_c0_g1_i9_m.2565829 | Endoplasmic reticulum metallopeptidase 1 | 1.27 | 0.0043 | GO:0016021;GO:0005783 |
| TRINITY_DN395619_c0_g1_i1_m.2557047 | putative phospholipid transporting ATPase 9 isoform X1 | 1.29 | 0.0004 | GO:0048194;GO:0045332;GO:0005802;GO:0016021;GO:0005739;GO:0000139;GO:0005886;GO:0000287;GO:0004012;GO:0005524 |
| TRINITY_DN398798_c2_g1_i3_m.1995725 | RING finger protein 160 | 1.22 | 0.0034 | GO:0000911;GO:0006486;GO:0010162;GO:0050826;GO:0009793;GO:0009933;GO:0009845;GO:0010228;GO:0009630;GO:0000226;GO:0010182;GO:0009909;GO:0009640;GO:0005829;GO:0008270 |
| TRINITY_DN379320_c1_g2_i6_m.1905422 | Glycogenin 2 | 1.24 | 0.0332 | GO:0006012;GO:0047216 |
| TRINITY_DN376289_c1_g1_i16_m.2945698 | beta glucosidase 7 like | 1.24 | 0.0001 | GO:0005975;GO:1901657;GO:0005576;GO:0047701;GO:0080079;GO:0004567;GO:0047668;GO:0102483;GO:0004565;GO:0033907;GO:0042803;GO:0080083;GO:0042973;GO:0050224 |
| TRINITY_DN347661_c0_g3_i2_m.2749352 | Cytokinin O glucosyltransferase 2 | 1.44 | 0.0074 | GO:0009813;GO:0052696;GO:0043231;GO:0080043;GO:0080044 |
| TRINITY_DN353050_c2_g1_i1_m.3031684 | S adenosylmethionine synthase 4、Methionine adenosyltransferase 4 | 1.23 | 0.0089 | GO:0006556;GO:0006730;GO:0005829;GO:0004478;GO:0046872;GO:0005524 |
| TRINITY_DN387086_c0_g1_i2_m.1413299 | ABC transporter D family member 1 | 1.24 | 0.0037 | GO:0055085;GO:0005739;GO:0016021;GO:0042626;GO:0005524 |
| TRINITY_DN385003_c4_g3_i1_m.973512 | thioredoxin like protein | 2.31 | 0.0407 | GO:0045454;GO:0006457;GO:0034976;GO:0005783;GO:0003756 |
| TRINITY_DN399529_c1_g1_i9_m.3002969 | protein RRP6 like 3 | 1.35 | 0.0126 | GO:0090305;GO:0005622;GO:0008408;GO:0000166;GO:0003676 |
| TRINITY_DN371921_c4_g1_i3_m.2918588 | adenine phosphoribosyltransferase 2 like | 1.26 | 0.0122 | GO:0006168;GO:0009116;GO:0005737;GO:0003999 |
| TRINITY_DN386437_c2_g2_i3_m.1440692 | PI PLC X domain containing protein At5g67130 like | 1.21 | 0.0005 | GO:0006891;GO:0009954;GO:0006635;GO:0016558;GO:0016310;GO:0010227;GO:0048439;GO:0016021;GO:0031463;GO:0008081;GO:0016301 |
| TRINITY_DN367833_c1_g1_i4_m.2169632 | probable small nuclear ribonucleoprotein F | 1.29 | 0.0073 | GO:0000398;GO:0005732;GO:0071013;GO:0019013;GO:0005685;GO:0003723 |
| TRINITY_DN391911_c0_g1_i3_m.1856800 | pyruvate dehydrogenase E1 component subunit alpha 3 | 1.24 | 0.0188 | GO:0006086;GO:0006096;GO:0055114;GO:0009941;GO:0009570;GO:0004739 |
| TRINITY_DN378729_c1_g1_i8_m.1483609 | glutathione S transferase 1 | 1.21 | 0.0045 | GO:0006749;GO:0005737;GO:0004364 |
| TRINITY_DN373621_c6_g1_i6_m.1755940 | 2 Cys peroxiredoxin BAS1 | 1.24 | 0.0042 | GO:0098869;GO:0006355;GO:0042744;GO:0055114;GO:0005634;GO:0005667;GO:0009507;GO:0005515;GO:0003700;GO:0051920;GO:0003677;GO:0004601 |

| | | | | |
|---|---|---|---|---|
| TRINITY_DN394569_c0_g1_i3_m.2962272 | probable L ascorbate peroxidase 7 | 1.22 | 0.0343 | GO:0098869;GO:0042744;GO:0006979;GO:0055114;GO:0009570;GO:0016021;GO:0020037;GO:0016688;GO:0046872 |
| TRINITY_DN312028_c1_g1_i4_m.1803056 | flagellar motor protein MotA | 3.04 | 0.0004 | GO:0015031;GO:0016021;GO:0008565 |
| TRINITY_DN171701_c0_g1_i1_m.577450 | vacuolar ATP synthase catalytic subunit a | 3.52 | 0.0050 | GO:0044267;GO:0015991;GO:0046034;GO:0007035;GO:0090463;GO:0090465;GO:0090464;GO:0055114;GO:0005829;GO:0000329;GO:0033180;GO:0050661;GO:0050660;GO:0004499;GO:0005524;GO:0046961 |
| TRINITY_DN515652_c0_g1_i1_m.3712056 | serine/threonine protein kinase | 1.91 | 0.0042 | GO:0006468;GO:0004674 |
| TRINITY_DN636289_c0_g1_i1_m.7732 | chemotaxis protein CheW | 2.44 | 0.0000 | GO:0007165;GO:0006935;GO:0016874;GO:0004871 |
| TRINITY_DN394721_c1_g1_i10_m.2627345 | vacuolar sorting receptor 6 | 2.15 | 0.0038 | GO:0016021;GO:0005509 |
| TRINITY_DN354304_c0_g2_i1_m.1065560 | cold shock protein | 2.21 | 0.0059 | GO:0006355;GO:0005737;GO:0003677 |
| TRINITY_DN378494_c1_g2_i4_m.2240860 | 30S ribosomal protein S19 | 2.18 | 0.0001 | GO:0006412;GO:0015935;GO:0003735;GO:0019843 |
| TRINITY_DN364110_c0_g1_i2_m.3311012 | phenylalanine ammonia lyase | 1.60 | 0.0383 | GO:0006559;GO:0009800;GO:0005737;GO:0016021;GO:0045548;GO:0052883 |
| TRINITY_DN370407_c0_g4_i2_m.3085047 | polynucleotide phosphorylase/polyadenylase | 1.71 | 0.0073 | GO:0090503;GO:0006402;GO:0006396;GO:0005737;GO:0004654;GO:0000287;GO:0000175;GO:0003723 |
| TRINITY_DN321266_c0_g1_i1_m.2866314 | type II citrate synthase | 1.93 | 0.0148 | GO:0006099;GO:0005737;GO:0004108 |
| TRINITY_DN384399_c0_g2_i3_m.2378263 | 30S ribosomal protein S16 | 2.00 | 0.0444 | GO:0006412;GO:0005840;GO:0003735 |
| TRINITY_DN772166_c0_g1_i1_m.4231719 | transcription elongation factor NusA | 2.05 | 0.0024 | GO:0031564;GO:0006414;GO:0005737;GO:0003700;GO:0000166;GO:0003746 |
| TRINITY_DN398748_c1_g2_i4_m.1995298 | putative endo 1 | 1.78 | 0.0161 | GO:0006076;GO:0009986;GO:0052861;GO:0042973 |
| TRINITY_DN376991_c0_g5_i1_m.1059960 | protein phosphatase 2C 45 like protein | 2.32 | 0.0272 | GO:0006470;GO:0016021;GO:0046872;GO:0004722 |
| TRINITY_DN200878_c0_g1_i1_m.3949870 | molecular chaperone SurA | 2.08 | 0.0103 | GO:0000413;GO:0060274;GO:0051085;GO:0043165;GO:0050821;GO:0015031;GO:0030288;GO:0042277;GO:0051082;GO:0003755 |
| TRINITY_DN352233_c0_g1_i3_m.1700897 | cytochrome b c1 complex subunit 9 | 1.77 | 0.0014 | GO:1902600;GO:0006122;GO:0009060;GO:0034551;GO:0005750;GO:0008121 |
| TRINITY_DN394817_c0_g1_i33_m.1746261 | Acyl CoA dehydrogenase family member 10 | 1.69 | 0.0010 | GO:0048767;GO:0033539;GO:0009610;GO:0031122;GO:0055088;GO:0005634;GO:0050660;GO:0052890;GO:0008568;GO:0000062;GO:0003995;GO:0005524;GO:0009055 |
| TRINITY_DN366217_c2_g2_i9_m.2500472 | probable glutathione S transferase GSTF1 | 1.65 | 0.0002 | GO:0006749;GO:0005737;GO:0004364 |
| TRINITY_DN389810_c1_g1_i13_m.2490263 | Chitinase 1 | 1.36 | 0.0299 | GO:0005975;GO:0006032;GO:0005576;GO:0004568;GO:0008061 |
| TRINITY_DN398018_c1_g3_i2_m.952403 | NADH dehydrogenase subunit 3 | 1.90 | 0.0006 | GO:0055114;GO:0016021;GO:0005747;GO:0008137 |
| TRINITY_DN94578_c0_g1_i1_m.4429038 | PrkA serine protein kinase | 1.68 | 0.0006 | GO:0006468;GO:0004674 |
| TRINITY_DN376173_c1_g1_i3_m.1049873 | glutathione transferase GST 23 like isoform X2 | 1.43 | 0.0058 | GO:0006952;GO:0006749;GO:0009407;GO:0005737;GO:0004364 |
| TRINITY_DN380600_c1_g1_i1_m.2743447 | BI1 like protein | 1.58 | 0.0236 | GO:0006355;GO:0016021;GO:0003700;GO:0003677 |
| TRINITY_DN391227_c2_g1_i1_m.2778159 | Acyl CoA binding protein | 1.98 | 0.0002 | GO:0000062 |
| TRINITY_DN277836_c0_g1_i1_m.3891312 | 12 oxophytodienoate reductase 1 | 1.50 | 0.0008 | GO:0055114;GO:0010181;GO:0016491 |
| TRINITY_DN233816_c0_g1_i1_m.3799411 | ATP synthase subunit beta | 1.67 | 0.0011 | GO:0015991;GO:0042777;GO:0045261;GO:0005886;GO:0046933;GO:0005524 |
| TRINITY_DN381254_c2_g1_i12_m.1252520 | Dihydroneopterin aldolase | 1.59 | 0.0007 | GO:0032981;GO:0046654;GO:0008380;GO:0046656;GO:0009536;GO:0005739;GO:0003723;GO:0004150 |
| TRINITY_DN381714_c6_g1_i3_m.1788831 | ABC transporter F family member 1 | 1.46 | 0.0166 | GO:0005524;GO:0016887 |
| TRINITY_DN876352_c0_g1_i1_m.3434074 | 50S ribosomal protein L13 | 1.27 | 0.0427 | GO:0006412;GO:0022625;GO:0003735;GO:0003729 |
| TRINITY_DN397133_c1_g1_i2_m.3278941 | Aquaporin PIP2 5 | 2.02 | 0.0024 | GO:0009992;GO:0034220;GO:0006833;GO:0015793;GO:0005773;GO:0005887;GO:0009506;GO:0015250;GO:0015254 |
| TRINITY_DN388751_c0_g1_i13_m.2540918 | adenine nucleotide transporter BT1 | 1.48 | 0.0025 | GO:0055085;GO:0006412;GO:0016021;GO:0003735 |
| TRINITY_DN399461_c3_g1_i1_m.1289269 | nudix hydrolase 23 | 1.47 | 0.0247 | GO:0042726;GO:0016021;GO:0005739;GO:0009507;GO:0047884 |
| TRINITY_DN366159_c0_g1_i2_m.2363510 | mediator of RNA polymerase II transcription subunit 20a like isoform X1 | 1.62 | 0.0441 | GO:0006357;GO:0016592;GO:0001104;GO:0003713 |
| TRINITY_DN399132_c9_g1_i11_m.912864 | High affinity nitrate transporter 2.1 | 1.32 | 0.0309 | GO:0055085;GO:0016021 |

| | | | | |
|---|---|---|---|---|
| TRINITY_DN383478_c3_g1_i4_m.2610653 | transmembrane 9 superfamily member 7 like | 1.38 | 0.0005 | GO:0016021;GO:0005739 |
| TRINITY_DN396946_c0_g3_i2_m.2675040 | callose synthase 12 like | 1.33 | 0.0212 | GO:0006075;GO:0016021;GO:0000148;GO:0003843 |
| TRINITY_DN399467_c2_g1_i7_m.1289826 | arginase | 1.27 | 0.0256 | GO:0000050;GO:0006527;GO:0005739;GO:0009507;GO:0004053;GO:0050897;GO:0030145 |
| TRINITY_DN396212_c1_g2_i3_m.1714946 | phospholipase D delta | 1.24 | 0.0309 | GO:0016042;GO:0012501;GO:0090333;GO:0009409;GO:0009789;GO:0046473;GO:0046470;GO:0005773;GO:0005886;GO:0009506;GO:0070290;GO:0005509;GO:0004630 |
| TRINITY_DN395596_c1_g1_i9_m.3045909 | V type proton ATPase subunit a3 | 1.56 | 0.0002 | GO:0015986;GO:0007035;GO:0015991;GO:0070072;GO:0000325;GO:0016021;GO:0000220;GO:0051117;GO:0046961 |
| TRINITY_DN387054_c1_g2_i2_m.1416466 | V type proton ATPase subunit C | 1.45 | 0.0089 | GO:0015991;GO:0033180;GO:0005774;GO:0015078 |
| TRINITY_DN388838_c0_g1_i11_m.1730580 | membrane protein of ER body 2 like isoform X1 | 1.62 | 0.0023 | GO:0000041;GO:0046916;GO:0098662;GO:0043231;GO:0016020;GO:0046915 |
| TRINITY_DN388866_c0_g1_i1_m.1728151 | aminotransferase | 1.27 | 0.0345 | GO:0005739;GO:0008483;GO:0042802;GO:0030170 |
| TRINITY_DN370223_c3_g1_i3_m.2481065 | proteasome subunit beta type 1 like | 1.29 | 0.0432 | GO:0006511;GO:0005737;GO:0005634;GO:0005839;GO:0004298 |
| TRINITY_DN371370_c0_g2_i2_m.2330748 | allene oxide cyclase 3 | 1.25 | 0.0027 | GO:0009507;GO:0046423 |
| TRINITY_DN397837_c1_g1_i12_m.1949955 | NADH dehydrogenase [ubiquinone] 1 alpha subcomplex subunit 13 B | 1.27 | 0.0013 | GO:0016021 |
| TRINITY_DN389426_c1_g2_i3_m.1814488 | uric acid degradation bifunctional protein TTL isoform X1 | 1.40 | 0.0030 | GO:0001560;GO:0009742;GO:0051289;GO:0019428;GO:0006144;GO:0005829;GO:0005777;GO:0031234;GO:0033971;GO:0051997 |
| TRINITY_DN398783_c1_g1_i12_m.1998846 | dolichol phosphate mannosyltransferase subunit 1 | 1.32 | 0.0047 | GO:0006487;GO:0006506;GO:0035269;GO:0019348;GO:0005789;GO:0004169;GO:0004582 |
| TRINITY_DN379806_c0_g3_i3_m.1243734 | hypersensitive induced response protein 4 | 1.37 | 0.0027 | GO:0005794;GO:0005886;GO:0009506;GO:0005774 |
| TRINITY_DN380549_c4_g1_i5_m.2743242 | proteasome subunit beta type 2 | 1.29 | 0.0257 | GO:0006511;GO:0005737;GO:0005634;GO:0005839;GO:0004298 |
| TRINITY_DN393456_c1_g1_i8_m.1687680 | cytosolic purine 5' nucleotidase isoform X1 | 1.25 | 0.0014 | GO:0016311;GO:0055114;GO:0016020;GO:0005739;GO:0051539;GO:0008253;GO:0016651;GO:0046872 |
| TRINITY_DN399310_c5_g1_i8_m.1326756 | putative 4 hydroxy 4 methyl 2 oxoglutarate aldolase 2 | 1.23 | 0.0211 | GO:0032259;GO:0051252;GO:0043086;GO:0008428;GO:0008948;GO:0008168;GO:0047443;GO:0046872 |
| TRINITY_DN397462_c0_g1_i3_m.1056453 | 3' N debenzoyl 2' deoxytaxol N benzoyltransferase like | 1.22 | 0.0051 | GO:0016747 |
| TRINITY_DN399940_c10_g2_i1_m.1628550 | LRR receptor like serine/threonine protein kinase | 1.22 | 0.0298 | GO:0006468;GO:0016021;GO:0004674;GO:0005524 |
| TRINITY_DN359075_c3_g1_i10_m.2564126 | translation elongation factor aef 2 | 3.92 | 0.0223 | GO:0006414;GO:0005622;GO:0003746;GO:0003924;GO:0005525 |
| TRINITY_DN305459_c0_g1_i1_m.3049105 | catalase/peroxidase HPI | 3.16 | 0.0394 | GO:0098869;GO:0042744;GO:0006979;GO:0055114;GO:0020037;GO:0004096;GO:0046872 |
| TRINITY_DN272429_c0_g1_i1_m.3893530 | aspartate semialdehyde dehydrogenase | 3.26 | 0.0019 | GO:0071266;GO:0019877;GO:0009089;GO:0009088;GO:0009097;GO:0055114;GO:0005737;GO:0050661;GO:0051287;GO:0003942;GO:0046983;GO:0004073 |
| TRINITY_DN203902_c0_g1_i1_m.3856591 | organic hydroperoxide resistance protein | 4.10 | 0.0021 | GO:0006979 |
| TRINITY_DN389157_c0_g2_i1_m.1230902 | Molybdenum cofactor sulfurase | 3.34 | 0.0191 | GO:0006777;GO:0000413;GO:0006457;GO:0016829;GO:0030151;GO:0016740;GO:0008265;GO:0030170;GO:0003755 |
| TRINITY_DN263877_c0_g1_i3_m.3960912 | insertase | 2.48 | 0.0035 | GO:0015031;GO:0051205;GO:0016021;GO:0005886 |
| TRINITY_DN352636_c0_g1_i2_m.1146915 | Reticuline oxidase like protein | 1.97 | 0.0087 | GO:0006355;GO:0043401;GO:0055114;GO:0005634;GO:0050660;GO:0016614;GO:0003677;GO:0005496;GO:0030284 |
| TRINITY_DN122558_c0_g1_i1_m.399937 | 3 isopropylmalate dehydratase small subunit | 1.46 | 0.0361 | GO:0009098;GO:0009316;GO:0003861;GO:0016853 |
| TRINITY_DN358454_c3_g1_i2_m.3333167 | DNA directed RNA polymerase subunit alpha | 1.65 | 0.0158 | GO:0006351;GO:0003899;GO:0046983;GO:0003677 |
| TRINITY_DN374297_c0_g1_i6_m.2302749 | pyrophosphate energized vacuolar membrane proton pump | 1.52 | 0.0277 | GO:0015992;GO:0055085;GO:0016021;GO:0005774;GO:0004427;GO:0009678;GO:0046872 |
| TRINITY_DN372813_c3_g1_i5_m.1410520 | Cucumisin | 1.56 | 0.0221 | GO:0006508;GO:0005618;GO:0016021;GO:0004252 |
| TRINITY_DN211387_c0_g1_i1_m.3763193 | succinate CoA ligase subunit beta | 1.96 | 0.0105 | GO:0006099;GO:0000287;GO:0030145;GO:0016746;GO:0004775;GO:0005524 |
| TRINITY_DN347579_c6_g1_i5_m.2548372 | small subunit ribosomal protein S20e | 1.92 | 0.0005 | GO:0002181;GO:0000462;GO:0022627;GO:0003735;GO:0003723 |
| TRINITY_DN368040_c0_g1_i9_m.3299901 | probable prefoldin subunit 5 | 2.06 | 0.0000 | GO:0006457;GO:0016272;GO:0005829;GO:0051082 |
| TRINITY_DN369264_c5_g1_i1_m.2472817 | mitochondrial import inner membrane translocase subunit Tim13 | 1.71 | 0.0007 | GO:0015031;GO:0005758;GO:0005743;GO:0005507;GO:0015450 |
| TRINITY_DN391943_c3_g1_i6_m.1854614 | Calreticulin | 1.67 | 0.0044 | GO:0006457;GO:0051208;GO:0007275;GO:0005788;GO:0030246;GO:0051082;GO:0005509 |

| | | | | |
|---|---|---|---|---|
| TRINITY_DN387677_c1_g1_i2_m.3106465 | Dolichyldiphosphatase 1 | 1.69 | 0.0008 | GO:0006651;GO:0006487;GO:0048868;GO:0016311;GO:0030176;GO:0009507;GO:0047874;GO:0008195 |
| TRINITY_DN377544_c1_g1_i8_m.1934963 | fosmidomycin resistance protein | 1.43 | 0.0051 | GO:0055085;GO:0016021 |
| TRINITY_DN389865_c0_g2_i4_m.2491231 | putative beta xylosidase | 1.44 | 0.0050 | GO:0031222;GO:0045493;GO:0009505;GO:0046556;GO:0009044 |
| TRINITY_DN383621_c1_g1_i9_m.3251076 | eukaryotic translation initiation factor 6 2 | 1.86 | 0.0042 | GO:0000460;GO:0000054;GO:0042256;GO:1902626;GO:0000470;GO:0006413;GO:0007229;GO:0005730;GO:0005829;GO:0030687;GO:0043023;GO:0043022;GO:0003743 |
| TRINITY_DN377479_c0_g1_i2_m.1098771 | cytochrome c1 2, heme protein | 1.51 | 0.0014 | GO:0020037;GO:0009055 |
| TRINITY_DN399606_c4_g1_i2_m.3205972 | lysine ketoglutarate reductase/saccharopine dehydrogenase | 1.51 | 0.0458 | GO:0055114;GO:0016021;GO:0016491 |
| TRINITY_DN392281_c0_g1_i2_m.1911054 | tRNA pseudouridine synthase A | 1.23 | 0.0128 | GO:0001522;GO:0009536;GO:0003723;GO:0009982 |
| TRINITY_DN375766_c0_g1_i2_m.938610 | aquaporin PIP1 1 | 1.60 | 0.0018 | GO:0009992;GO:0034220;GO:0006833;GO:0015793;GO:0005773;GO:0005887;GO:0009506;GO:0015250;GO:0015254 |
| TRINITY_DN395511_c1_g2_i2_m.3041349 | neutral/alkaline invertase 1 | 1.54 | 0.0146 | GO:0005739;GO:0033926 |
| TRINITY_DN362466_c3_g3_i2_m.1862740 | ABC transporter C family member 14 | 1.36 | 0.0038 | GO:0055085;GO:0009536;GO:0016021;GO:0042626;GO:0005524 |
| TRINITY_DN390892_c2_g3_i1_m.2291739 | eukaryotic elongation factor 1 gamma | 1.42 | 0.0066 | GO:0006749;GO:0006414;GO:0005853;GO:0005634;GO:0004364;GO:0003746 |
| TRINITY_DN339719_c0_g1_i2_m.2766316 | phosphatidylinositol 3 and 4 kinase | 1.65 | 0.0028 | GO:0048015;GO:0046854;GO:0005622;GO:0005886;GO:0004430 |
| TRINITY_DN351152_c0_g1_i1_m.1872693 | U6 snRNA associated Sm like protein LSm6 | 1.47 | 0.0022 | GO:0030490;GO:0000398;GO:0046540;GO:0005732;GO:0005730;GO:0000932;GO:0019013;GO:0005688;GO:0003723 |
| TRINITY_DN397038_c0_g2_i1_m.2504707 | NADH dehydrogenase [ubiquinone] 1 alpha subcomplex subunit 1 | 1.31 | 0.0002 | GO:0051788;GO:0009853;GO:0006511;GO:0016021;GO:0005747 |
| TRINITY_DN389801_c4_g1_i3_m.997451 | probable aspartyl aminopeptidase | 1.45 | 0.0014 | GO:0006508;GO:0008270;GO:0008237;GO:0004177 |
| TRINITY_DN390035_c0_g2_i9_m.1197912 | probable sucrose phosphate synthase 4 | 1.28 | 0.0010 | GO:0005986;GO:0046524;GO:0016157 |
| TRINITY_DN388294_c0_g1_i2_m.1185887 | Pyruvate dehydrogenase kinase | 1.25 | 0.0147 | GO:0016310;GO:0016301 |
| TRINITY_DN368389_c3_g1_i4_m.2083606 | nudix hydrolase 2 | 1.50 | 0.0010 | GO:0009536;GO:0016787 |
| TRINITY_DN375199_c2_g1_i4_m.3260894 | pantoate beta alanine ligase isoform X1 | 1.29 | 0.0431 | GO:0015940;GO:0009793;GO:0005829;GO:0004592;GO:0005524;GO:0042803 |
| TRINITY_DN393922_c1_g2_i2_m.1537823 | phosphoenolpyruvate/phosphate translocator 1 | 1.24 | 0.0010 | GO:0089722;GO:0035436;GO:0034219;GO:0031969;GO:0016021;GO:0009670;GO:0015121 |
| TRINITY_DN388330_c0_g1_i2_m.1977646 | Acyl coenzyme A oxidase 4 | 1.22 | 0.0423 | GO:0007033;GO:0007030;GO:0044265;GO:0016192;GO:0016558;GO:0033539;GO:0006623;GO:0055088;GO:0009793;GO:0046459;GO:0005777;GO:0052890;GO:0000062;GO:0050660;GO:0003995;GO:0003997;GO:0009055 |
| TRINITY_DN383094_c0_g2_i3_m.2882414 | putative 5' adenylylsulfate reductase 1 | 1.51 | 0.0099 | GO:0019419;GO:0019344;GO:0045454;GO:0009507;GO:0033741;GO:0051539;GO:0009973;GO:0004604;GO:0046872 |
| TRINITY_DN372281_c0_g1_i5_m.3090457 | UDP glycosyltransferase 89B1 | 1.28 | 0.0045 | GO:0009813;GO:0052696;GO:0043231;GO:0080043;GO:0080044 |
| TRINITY_DN389203_c3_g2_i3_m.1228205 | long chain base biosynthesis protein 1a | 1.21 | 0.0071 | GO:0030148;GO:0016049;GO:0043067;GO:0046686;GO:0016021;GO:0005789;GO:0004758;GO:0030170 |
| TRINITY_DN398783_c1_g1_i7_m.1998813 | dolichol phosphate mannosyltransferase subunit 1 | 1.49 | 0.0019 | GO:0006487;GO:0006506;GO:0035269;GO:0019348;GO:0005789;GO:0004169;GO:0004582 |
| TRINITY_DN393827_c2_g1_i5_m.3300827 | fructose 1,6 bisphosphatase | 1.28 | 0.0364 | GO:0016311;GO:0005975;GO:0005737;GO:0042132;GO:0046872 |
| TRINITY_DN379421_c0_g2_i1_m.2939760 | porphobilinogen deaminase | 1.27 | 0.0021 | GO:0015995;GO:0006782;GO:0019684;GO:0009965;GO:0018160;GO:0019344;GO:0009409;GO:0019288;GO:0009697;GO:0045893;GO:0030154;GO:0006744;GO:0009814;GO:0009941;GO:0048046;GO:0004418 |
| TRINITY_DN382173_c1_g1_i16_m.1368166 | uracil phosphoribosyltransferase | 1.28 | 0.0227 | GO:0006222;GO:0043097;GO:0006223;GO:0005829;GO:0009507;GO:0004849;GO:0004845 |
| TRINITY_DN378339_c0_g1_i11_m.861871 | serine/arginine rich splicing factor RSZ23 isoform X1 | 1.31 | 0.0167 | GO:0008270;GO:0003676;GO:0000166 |
| TRINITY_DN388026_c0_g3_i1_m.2951450 | ascorbate peroxidase | 1.32 | 0.0361 | GO:0098869;GO:0042744;GO:0006979;GO:0055114;GO:0016021;GO:0020037;GO:0016688;GO:0046872 |
| TRINITY_DN389672_c3_g1_i2_m.2350270 | elongation factor Tu GTP binding domain containing protein 1 | 1.23 | 0.0234 | GO:0042256;GO:0006414;GO:0005622;GO:0003924;GO:0003746;GO:0005525 |
| TRINITY_DN386666_c2_g3_i1_m.987892 | synaptotagmin 5 like | 1.23 | 0.0191 | GO:0016020;GO:0005783;GO:0008289 |
| TRINITY_DN391718_c0_g1_i13_m.2157129 | 3 hydroxyisobutyryl CoA hydrolase like protein 3 | 1.28 | 0.0080 | GO:0003860 |

| | | | | |
|---|---|---|---|---|
| TRINITY_DN390612_c1_g2_i2_m.1393354 | probable hydroxyacylglutathione hydrolase 2 | 1.30 | 0.0120 | GO:0019243;GO:0009072;GO:0005737;GO:0016021;GO:0004416 |
| TRINITY_DN316963_c0_g1_i1_m.970527 | 60S ribosomal protein L38 | 1.40 | 0.0277 | GO:0022618;GO:0006412;GO:0022625;GO:0016021;GO:0003735;GO:0003723 |
| TRINITY_DN360434_c0_g1_i4_m.1214073 | Leucine aminopeptidase 2 | 1.31 | 0.0190 | GO:0006508;GO:0009507;GO:0004177;GO:0008235;GO:0030145 |
| TRINITY_DN398531_c1_g2_i2_m.1123981 | PTI1 like tyrosine protein kinase 1 | 1.31 | 0.0222 | GO:0018108;GO:0004713;GO:0005524 |
| TRINITY_DN409090_c0_g1_i1_m.746638 | succinyl CoA synthetase subunit beta | 3.34 | 0.0007 | GO:0006099;GO:0004775;GO:0000287;GO:0030145;GO:0005524 |
| TRINITY_DN316161_c0_g1_i2_m.969260 | ribosome recycling factor | 4.57 | 0.0115 | GO:0006415;GO:0005737 |
| TRINITY_DN384248_c0_g1_i2_m.1843807 | iron/phytosiderophore transporter | 2.48 | 0.0072 | GO:0055072;GO:0006811;GO:0055085;GO:0016021;GO:0005886 |
| TRINITY_DN318959_c0_g1_i4_m.3197878 | spheroplast protein y | 3.28 | 0.0346 | GO:0042597 |
| TRINITY_DN380694_c0_g1_i1_m.3247452 | putative endo beta 1,4 glucanase D | 3.89 | 0.0080 | GO:0016787 |
| TRINITY_DN362189_c1_g1_i1_m.2079582 | 30S ribosomal protein S18 | 2.49 | 0.0167 | GO:0006412;GO:0005840;GO:0003735;GO:0019843 |
| TRINITY_DN268850_c0_g1_i2_m.3981394 | RraA family protein | 2.96 | 0.0182 | GO:0051252;GO:0043086;GO:0008428;GO:0008948;GO:0047443;GO:0046872 |
| TRINITY_DN482420_c0_g1_i1_m.800645 | rod shape determining protein MreB | 1.90 | 0.0128 | GO:0000902 |
| TRINITY_DN323848_c1_g1_i2_m.1481390 | sugar ABC transporter | 2.30 | 0.0186 | GO:0008643 |
| TRINITY_DN311822_c0_g1_i2_m.2976388 | H NS histone family protein | 1.83 | 0.0095 | GO:0046983 |
| TRINITY_DN361855_c1_g1_i3_m.1571914 | GEM like protein 5 | 1.72 | 0.0021 | GO:0009793;GO:0010286;GO:0010029;GO:0098755 |
| TRINITY_DN5557_c1_g1_i1_m.4442544 | endopeptidase La | 1.94 | 0.0004 | GO:0033554;GO:0006515;GO:0005737;GO:0004252;GO:0004176;GO:0043565;GO:0005524 |
| TRINITY_DN390936_c0_g1_i20_m.2251132 | 40S ribosomal protein S0 | 1.64 | 0.0095 | GO:0000028;GO:0000461;GO:0000447;GO:0006412;GO:0006407;GO:0022627;GO:0030686;GO:0003735 |
| TRINITY_DN108115_c0_g1_i1_m.450055 | chemotaxis protein | 1.64 | 0.0053 | GO:0007165;GO:0016021;GO:0004871 |
| TRINITY_DN47265_c0_g1_i1_m.4501394 | molecular chaperone GroES | 1.59 | 0.0451 | GO:0006457;GO:0005737;GO:0005524 |
| TRINITY_DN399495_c11_g1_i1_m.1285746 | isovaleryl CoA dehydrogenase | 1.72 | 0.0250 | GO:0033539;GO:0006552;GO:0055088;GO:0005739;GO:0050660;GO:0052890;GO:0000062;GO:0008470;GO:0009055 |
| TRINITY_DN372103_c4_g1_i2_m.2898424 | aspartyl tRNA synthetase | 1.67 | 0.0036 | GO:0006422;GO:0005829;GO:0005634;GO:0004815;GO:0003723;GO:0005524 |
| TRINITY_DN338356_c0_g1_i1_m.2268268 | 60S ribosomal protein L22 2 | 1.72 | 0.0000 | GO:0002181;GO:0022625;GO:0003735 |
| TRINITY_DN208430_c0_g1_i1_m.4027339 | adenosine nucleotide translocator | 1.45 | 0.0347 | GO:0006412;GO:0055085;GO:0016021;GO:0005743;GO:0005215;GO:0003735 |
| TRINITY_DN357369_c1_g2_i1_m.1293546 | germin like protein 8 6 | 1.42 | 0.0108 | GO:0033609;GO:0005618;GO:0005576;GO:0030145;GO:0045735;GO:0046564 |
| TRINITY_DN377698_c2_g4_i3_m.3221200 | endo 1,3;1,4 beta D glucanase isoform X1 | 1.48 | 0.0061 | GO:0016787 |
| TRINITY_DN382452_c0_g1_i1_m.2034020 | succinate dehydrogenase subunit 4 | 1.77 | 0.0111 | GO:0055114;GO:0045273;GO:0016021;GO:0005739 |
| TRINITY_DN398893_c5_g1_i2_m.1563254 | pentatricopeptide repeat containing protein At3g02650 | 1.36 | 0.0095 | GO:0006412;GO:0005840;GO:0005739;GO:0003735 |
| TRINITY_DN338356_c0_g1_i1_m.2268268 | 60S ribosomal protein L22 2 | 1.72 | 0.0000 | GO:0002181;GO:0022625;GO:0003735 |
| TRINITY_DN381233_c0_g3_i3_m.1253245 | 30S ribosomal protein S11 | 1.64 | 0.0003 | GO:0000028;GO:0000462;GO:0006412;GO:0022627;GO:0070181;GO:0016740;GO:0003735;GO:0048027 |
| TRINITY_DN357369_c1_g2_i1_m.1293546 | germin like protein 8 6 | 1.42 | 0.0108 | GO:0033609;GO:0005618;GO:0005576;GO:0030145;GO:0045735;GO:0046564 |
| TRINITY_DN383879_c0_g1_i8_m.1892269 | putative ER lumen protein retaining receptor C28H8.4 | 1.32 | 0.0036 | GO:0006621;GO:0016021;GO:0005739;GO:0005783;GO:0046923 |
| TRINITY_DN364178_c2_g1_i15_m.3312603 | beta fructofuranosidase 1 like | 1.37 | 0.0164 | GO:0005975;GO:0016021;GO:0004575 |
| TRINITY_DN324154_c0_g1_i1_m.2706442 | phosphopyruvate hydratase family protein | 1.99 | 0.0298 | GO:0006096;GO:0000015;GO:0000287;GO:0004634 |
| TRINITY_DN378494_c1_g2_i4_m.2240853 | 50S ribosomal protein L2 | 1.83 | 0.0031 | GO:0006412;GO:0015934;GO:0003735;GO:0016740;GO:0019843 |
| TRINITY_DN364115_c0_g2_i5_m.3311187 | 40s ribosomal protein s28 | 1.33 | 0.0259 | GO:0006407;GO:0022627;GO:0003735 |
| TRINITY_DN371105_c1_g4_i2_m.2070771 | UBP1 associated protein 2B like | 1.28 | 0.0035 | GO:0003676;GO:0000166 |
| TRINITY_DN367925_c0_g1_i1_m.1770215 | 50S ribosomal protein L12 2 | 1.51 | 0.0030 | GO:0006354;GO:0006412;GO:0005840;GO:0005739;GO:0003735 |
| TRINITY_DN393940_c2_g1_i2_m.1536269 | Bifunctional dihydroflavonol 4 reductase/flavanone 4 reductase | 1.43 | 0.0002 | GO:0050662;GO:0003824 |
| TRINITY_DN375570_c1_g1_i3_m.1633653 | translocon associated protein subunit beta | 1.65 | 0.0057 | GO:0009627;GO:0034976;GO:0005794;GO:0016021;GO:0005774;GO:0005789;GO:0009506 |
| TRINITY_DN393281_c1_g1_i3_m.1876357 | signal peptide peptidase 2 like | 1.61 | 0.0004 | GO:0009555;GO:0033619;GO:0006465;GO:0009846;GO:0030660;GO:0005765;GO:0071556;GO:0071458;GO:0042500 |

| | | | | |
|---|---|---|---|---|
| TRINITY_DN394076_c3_g1_i4_m.899051 | protein GPR107 | 1.30 | 0.0086 | GO:0016021 |
| TRINITY_DN369975_c2_g2_i3_m.2128679 | Reticuline oxidase like protein | 1.53 | 0.0070 | GO:0055114;GO:0050660;GO:0016614 |
| TRINITY_DN379806_c0_g2_i5_m.1243753 | hypersensitive induced reaction protein 4 | 1.40 | 0.0001 | GO:0005794;GO:0005886;GO:0009506;GO:0005774 |
| TRINITY_DN380966_c4_g2_i13_m.933881 | dolichyl diphosphooligosaccharide protein glycosyltransferase 48 kDa subunit | 1.49 | 0.0046 | GO:0009826;GO:0009664;GO:0001510;GO:0030244;GO:0018279;GO:0005794;GO:0005730;GO:0005774;GO:0016021;GO:0008250;GO:0009505;GO:0009506;GO:0016757 |
| TRINITY_DN364475_c1_g1_i8_m.3168579 | Putative inorganic phosphate transporter 1 8 | 1.42 | 0.0079 | GO:0006817;GO:0055085;GO:0005887;GO:0022891;GO:0015293;GO:0005315 |
| TRINITY_DN388587_c1_g1_i2_m.3154222 | glucan endo 1,3 beta glucosidase 8 like | 1.30 | 0.0123 | GO:0005975;GO:0046658;GO:0016021;GO:0030247;GO:0004553 |
| TRINITY_DN325686_c0_g1_i1_m.1602828 | dihydrolipoyllysine residue acetyltransferase component 4 of pyruvate dehydrogenase complex | 1.46 | 0.0015 | GO:0008152;GO:0009941;GO:0022626;GO:0009534;GO:0009570;GO:0016020;GO:0004742 |
| TRINITY_DN394548_c2_g2_i6_m.2967381 | cation chloride cotransporter 1 like isoform X4 | 1.31 | 0.0056 | GO:0071805;GO:1902476;GO:0055075;GO:0055064;GO:0016021;GO:0015379;GO:0022820 |
| TRINITY_DN385785_c0_g1_i1_m.1145765 | alpha mannosidase | 1.33 | 0.0002 | GO:0006013;GO:0006517;GO:0008270;GO:0004559;GO:0030246 |
| TRINITY_DN342110_c0_g1_i1_m.3308010 | alpha glucosidase 2 | 1.28 | 0.0000 | GO:0005975;GO:0009507;GO:0030246;GO:0004553 |
| TRINITY_DN313097_c0_g1_i1_m.1362957 | V type proton ATPase subunit c1 | 1.21 | 0.0478 | GO:0015991;GO:0007035;GO:0006364;GO:0016021;GO:0000220;GO:0046961 |
| TRINITY_DN771345_c0_g1_i1_m.4363218 | Putative ornithine aminotransferase | 1.26 | 0.0294 | GO:0006635;GO:0006593;GO:0009413;GO:0009414;GO:0055129;GO:0042538;GO:0009733;GO:0009737;GO:0009753;GO:0019544;GO:0010121;GO:0007031;GO:0051646;GO:0009408;GO:0006979;GO:0010260;GO:0009741;GO:0005759;GO:0030170;GO:0042802;GO:0004587;GO:0008270 |
| TRINITY_DN385954_c1_g1_i9_m.1076210 | amylogenin | 1.35 | 0.0034 | GO:0009832;GO:0030244;GO:0071555;GO:0033356;GO:0005794;GO:0005829;GO:0009506;GO:0052691;GO:0005515;GO:0016740 |
| TRINITY_DN369513_c2_g1_i1_m.3037915 | la related protein 1A like | 1.36 | 0.0263 | GO:0005739 |
| TRINITY_DN390951_c2_g1_i1_m.2248420 | lipoate protein ligase A | 1.37 | 0.0396 | GO:0006464;GO:0005739;GO:0016874 |
| TRINITY_DN16407_c0_g1_i1_m.4415582 | RNA polymerase sigma factor RpoD | 1.20 | 0.0443 | GO:0001123;GO:0006355;GO:0005737;GO:0003700;GO:0016987;GO:0003677 |
| TRINITY_DN377737_c0_g3_i1_m.2645185 | 14 3 3 like protein GF14 E isoform X2 | 1.22 | 0.0031 | GO:0005737;GO:0005634;GO:0019904 |
| TRINITY_DN363784_c0_g1_i7_m.3273579 | dihydrolipoyllysine residue succinyltransferase component of 2 oxoglutarate dehydrogenase complex 1 | 1.28 | 0.0029 | GO:0006099;GO:0005739;GO:0016021;GO:0045252;GO:0004149 |
| TRINITY_DN339665_c0_g1_i2_m.2109730 | 2 hydroxyisoflavanone dehydratase | 1.43 | 0.0089 | GO:0008152;GO:0016787 |
| TRINITY_DN347152_c0_g1_i1_m.2826704 | Ferredoxin NADP reductase | 1.20 | 0.0097 | GO:0022900;GO:0015979;GO:0009507;GO:0004324 |
| TRINITY_DN371641_c2_g3_i2_m.2887268 | NADH dehydrogenase subunit 6 | 1.40 | 0.0086 | GO:0055114;GO:0016021;GO:0005747;GO:0008137 |
| TRINITY_DN384057_c2_g1_i7_m.1263311 | chaperone protein ClpC2 | 1.22 | 0.0009 | GO:0006508;GO:0009507;GO:0008233;GO:0005524 |
| TRINITY_DN383994_c1_g1_i5_m.2788659 | outer envelope pore protein 37 | 1.27 | 0.0132 | GO:0009536;GO:0019031 |
| TRINITY_DN369613_c1_g1_i15_m.3025965 | Putative ATP synthase protein YMF19、18 kDa membrane bound protein | 1.23 | 0.0043 | GO:0015992;GO:0006754;GO:0055085;GO:0045263;GO:0016021;GO:0031966;GO:0016820;GO:0005524 |
| TRINITY_DN750734_c0_g1_i1_m.4330939 | nitrate reductase A subunit gamma | 6.88 | 0.0005 | GO:0055114;GO:0016021;GO:0009325;GO:0008940 |
| TRINITY_DN263918_c0_g1_i2_m.4011175 | sodium:solute symporter | 4.84 | 0.0084 | GO:0055085;GO:0016021;GO:0005215 |
| TRINITY_DN399492_c1_g1_i11_m.1284646 | 50S ribosomal protein L29 | 5.34 | 0.0045 | GO:0006412;GO:0005840;GO:0003735 |
| TRINITY_DN383296_c3_g2_i1_m.3052650 | 40S ribosomal protein S5 | 3.63 | 0.0058 | GO:0000028;GO:0006412;GO:0022627;GO:0019843;GO:0003735;GO:0003729 |
| TRINITY_DN712761_c0_g1_i1_m.4362057 | nitrate reductase subunit beta | 3.04 | 0.0041 | GO:0042126;GO:0055114;GO:0009325;GO:0008940 |
| TRINITY_DN291200_c0_g1_i1_m.3801164 | biopolymer transporter ExbD | 3.86 | 0.0030 | GO:0015031;GO:0016021;GO:0005886;GO:0005215 |
| TRINITY_DN388896_c2_g2_i4_m.1728299 | 30S ribosomal protein S2 | 2.85 | 0.0000 | GO:0006412;GO:0015935;GO:0003735 |
| TRINITY_DN80453_c0_g1_i1_m.4491666 | thiol reductase thioredoxin | 2.61 | 0.0053 | GO:0045454;GO:0006662;GO:0055114;GO:0005623;GO:0015035 |
| TRINITY_DN380374_c0_g5_i4_m.1212237 | Protein synthesis inhibitor I | 2.44 | 0.0011 | GO:0006952;GO:0065007;GO:0051704;GO:0016787 |
| TRINITY_DN338374_c0_g1_i2_m.2268329 | MATE efflux family protein 5 | 1.90 | 0.0124 | GO:0006855;GO:0016021;GO:0015297;GO:0015238 |
| TRINITY_DN222186_c0_g1_i1_m.4097449 | DNA binding protein HRL18 | 1.99 | 0.0274 | GO:0030261;GO:0003677 |
| TRINITY_DN205512_c0_g1_i3_m.4049583 | aconitate hydratase | 1.74 | 0.0110 | GO:0008152;GO:0003994;GO:0051539 |

| | | | | |
|---|---|---|---|---|
| TRINITY_DN386743_c0_g1_i7_m.2813844 | Inactive rhomboid protein 1 | 2.10 | 0.0493 | GO:0016485;GO:0016021;GO:0004252 |
| TRINITY_DN754757_c0_g1_i1_m.4248475 | dihydrolipoamide succinyltransferase | 2.13 | 0.0008 | GO:0006099;GO:0033512;GO:0045252;GO:0004149 |
| TRINITY_DN399318_c1_g1_i5_m.1325816 | elongation factor G | 2.14 | 0.0119 | GO:0006414;GO:0005737;GO:0003746;GO:0003924;GO:0005525 |
| TRINITY_DN321571_c0_g1_i1_m.2865647 | F0F1 ATP synthase subunit alpha | 1.80 | 0.0105 | GO:0015991;GO:0042777;GO:0045261;GO:0005886;GO:0046933;GO:0046961;GO:0005524 |
| TRINITY_DN392237_c3_g1_i7_m.1907378 | 50S ribosomal protein L9 | 1.71 | 0.0252 | GO:0006412;GO:0005840;GO:0003735;GO:0019843 |
| TRINITY_DN859512_c0_g1_i1_m.3426681 | pilin | 2.38 | 0.0085 | GO:0007155;GO:0016021;GO:0009289 |
| TRINITY_DN163667_c0_g1_i2_m.486161 | fructose 1,6 bisphosphate aldolase | 2.19 | 0.0008 | GO:0006096;GO:0008270;GO:0004332 |
| TRINITY_DN398969_c2_g1_i2_m.1280326 | Protein kinase dsk1 | 1.67 | 0.0033 | GO:0006468;GO:0016021;GO:0004674;GO:0005524 |
| TRINITY_DN363392_c1_g2_i3_m.2258146 | protein disulfide isomerase | 1.94 | 0.0003 | GO:0045454;GO:0055114;GO:0005783;GO:0003756;GO:0010181;GO:0016491 |
| TRINITY_DN399750_c0_g2_i5_m.3142007 | ABC transporter G family member 48 isoform X1 | 1.46 | 0.0095 | GO:0006810;GO:0016021;GO:0005524;GO:0016887 |
| TRINITY_DN396940_c2_g1_i4_m.2669880 | Lipoxygenase 2 | 1.64 | 0.0300 | GO:0031408;GO:0009611;GO:0051707;GO:0055114;GO:0005737;GO:0016702;GO:0008270;GO:0003676 |
| TRINITY_DN393045_c0_g1_i4_m.1152347 | isocitrate dehydrogenase, NADP dependent | 1.44 | 0.0311 | GO:0055114;GO:0016616 |
| TRINITY_DN374268_c1_g1_i5_m.2300984 | Peroxidase 4 | 1.54 | 0.0012 | GO:0098869;GO:0042744;GO:0006979;GO:0055114;GO:0016021;GO:0005576;GO:0020037;GO:0004601;GO:0046872 |
| TRINITY_DN609325_c0_g1_i1_m.77502 | TonB dependent receptor | 1.62 | 0.0000 | GO:0006810;GO:0009279;GO:0004872 |
| TRINITY_DN379745_c3_g2_i3_m.2458333 | 60S ribosomal protein L20 | 1.51 | 0.0060 | GO:0006412;GO:0005840;GO:0003735 |
| TRINITY_DN376787_c3_g1_i6_m.2860675 | beta glucosidase 22 like | 1.42 | 0.0301 | GO:1901657;GO:0005975;GO:0102483;GO:0008422 |
| TRINITY_DN608310_c0_g1_i1_m.86749 | DNA J chaperone | 1.59 | 0.0247 | GO:0006457;GO:0009408;GO:0031072;GO:0051082;GO:0046872;GO:0005524 |
| TRINITY_DN322101_c0_g1_i1_m.3003833 | phosphatidylinositol binding clathrin assembly protein LAP isoform X8 | 1.34 | 0.0388 | GO:0048268;GO:0030136;GO:0030276;GO:0005545 |
| TRINITY_DN376048_c0_g1_i4_m.3351653 | Cell division protease ftsH like protein | 1.34 | 0.0113 | GO:0051301;GO:0006508;GO:0016020;GO:0005739;GO:0004176;GO:0004222;GO:0046872;GO:0005524 |
| TRINITY_DN399176_c3_g3_i1_m.912716 | protein SAND isoform X1 | 1.50 | 0.0042 | GO:0016192;GO:0012505 |
| TRINITY_DN395646_c1_g1_i12_m.2553863 | putative glycoprotein 3 alpha L fucosyltransferase | 1.22 | 0.0343 | GO:0036065;GO:0006486;GO:0016021;GO:0005739;GO:0032580;GO:0018392 |
| TRINITY_DN399750_c0_g1_i8_m.3141995 | PDR type ABC transporter | 1.42 | 0.0001 | GO:0006810;GO:0016021;GO:0005524;GO:0016887 |
| TRINITY_DN237735_c0_g1_i1_m.4121687 | D xylose ABC transporter substrate binding protein | 3.71 | 0.0196 | GO:0015753;GO:0048029 |
| TRINITY_DN57125_c0_g1_i1_m.4573711 | Alcohol dehydrogenase 3 | 3.00 | 0.0002 | GO:0032774;GO:0008643;GO:0055114;GO:0008270;GO:0003899;GO:0016491 |
| TRINITY_DN277343_c0_g1_i1_m.3940051 | pyruvate dehydrogenase | 2.39 | 0.0208 | GO:0055114;GO:0004739 |
| TRINITY_DN370407_c0_g4_i4_m.3085052 | polyribonucleotide nucleotidyltransferase | 2.16 | 0.0002 | GO:0090503;GO:0006402;GO:0006396;GO:0005737;GO:0004654;GO:0000287;GO:0000175;GO:0003723 |
| TRINITY_DN551556_c0_g1_i1_m.3599743 | histone fold containing protein | 2.11 | 0.0159 | GO:0005634;GO:0000786;GO:0046982;GO:0003677 |
| TRINITY_DN126350_c0_g1_i1_m.475281 | NADH:ubiquinone reductase | 2.42 | 0.0259 | GO:0006814;GO:0055114;GO:0016021;GO:0005886;GO:0051537;GO:0016655;GO:0046872;GO:0009055 |
| TRINITY_DN395614_c0_g1_i4_m.2552703 | GDP mannose transporter GONST3 like isoform X1 | 1.66 | 0.0109 | GO:0016021 |
| TRINITY_DN49452_c0_g1_i1_m.4583186 | serine peptidase | 2.21 | 0.0008 | GO:0006508;GO:0042597;GO:0004252 |
| TRINITY_DN393860_c3_g2_i1_m.3304521 | Transaldolase | 1.92 | 0.0039 | GO:0005975;GO:0006098;GO:0005634;GO:0005829;GO:0004801 |
| TRINITY_DN294444_c0_g1_i3_m.3842490 | UTP glucose 1 phosphate uridylyltransferase | 2.20 | 0.0128 | GO:0009058;GO:0006011;GO:0003983 |
| TRINITY_DN364475_c1_g1_i7_m.3168576 | phosphate transporter HvPT4 | 2.14 | 0.0002 | GO:0006817;GO:0055085;GO:0005887;GO:0022891;GO:0015293;GO:0005315 |
| TRINITY_DN385023_c0_g1_i3_m.2190530 | abscisic acid insensitive 8 homologue | 1.45 | 0.0021 | GO:0016021 |
| TRINITY_DN373634_c0_g2_i10_m.1758595 | Ubiquitin conjugating enzyme E2 5 | 1.32 | 0.0004 | GO:0016567;GO:0006511;GO:0005737;GO:0031625;GO:0061630;GO:0005524 |
| TRINITY_DN378703_c0_g1_i3_m.3271898 | CASP like protein 4B2 | 1.50 | 0.0054 | GO:0016021;GO:0005886 |
| TRINITY_DN393526_c0_g1_i9_m.2755326 | caffeic acid O methyltransferase | 1.31 | 0.0037 | GO:0032259;GO:0009809;GO:0009813;GO:0008171;GO:0046983 |
| TRINITY_DN394186_c3_g1_i12_m.1899482 | lysine histidine transporter 1 like | 1.43 | 0.0000 | GO:0003333;GO:0016021;GO:0009536;GO:0005886;GO:0015171 |

| | | | | |
|---|---|---|---|---|
| TRINITY_DN392651_c0_g2_i7_m.2118737 | alpha 1,3 mannosyl glycoprotein | 1.44 | 0.0001 | GO:0006491;GO:0006972;GO:0005802;GO:0005768;GO:0016262;GO:0003827 |
| TRINITY_DN374781_c0_g6_i1_m.3184776 | Cytokinin O glucosyltransferase 3 | 1.34 | 0.0001 | GO:0009813;GO:0052696;GO:0043231;GO:0080043;GO:0080044 |
| TRINITY_DN369415_c0_g2_i5_m.1469279 | Serine carboxypeptidase like 50 | 1.38 | 0.0007 | GO:0051603;GO:0004185 |
| TRINITY_DN379497_c2_g1_i2_m.2938296 | transmembrane 9 superfamily member 1 like | 1.20 | 0.0137 | GO:0016021 |
| TRINITY_DN396146_c2_g3_i7_m.3078320 | delta 1 pyrroline 5 carboxylate dehydrogenase | 1.36 | 0.0192 | GO:0072593;GO:0010133;GO:0055114;GO:0005739;GO:0009507;GO:0004028;GO:0004029;GO:0050897;GO:0003842;GO:0008270 |
| TRINITY_DN399807_c2_g3_i1_m.2045177 | Clathrin heavy chain 1 | 1.22 | 0.0251 | GO:0006886;GO:0016192;GO:0030130;GO:0030132;GO:0005198 |
| TRINITY_DN398766_c3_g3_i2_m.1997034 | NADH ubiquinone oxidoreductase chain 1 | 1.30 | 0.0058 | GO:0006120;GO:0016021;GO:0005886;GO:0005747;GO:0008137 |
| TRINITY_DN368029_c0_g1_i2_m.3298254 | probable 6 phosphogluconolactonase 4 | 1.26 | 0.0000 | GO:0006098;GO:0005975;GO:0009536;GO:0017057 |
| TRINITY_DN368908_c0_g1_i2_m.3149258 | Leucyl tRNA synthetase | 1.42 | 0.0025 | GO:0006450;GO:0006429;GO:0005829;GO:0016021;GO:0004823;GO:0002161;GO:0005524 |
| TRINITY_DN368055_c0_g1_i2_m.3300700 | CuZnSOD | 1.39 | 0.0095 | GO:0071486;GO:0019430;GO:0055114;GO:0009579;GO:0009570;GO:0048046;GO:0008270;GO:0004784;GO:0005507 |
| TRINITY_DN308541_c0_g1_i1_m.3342875 | GTP cyclohydrolase I | 1.55 | 0.0233 | GO:0006730;GO:0035998;GO:0046654;GO:0005737;GO:0003934;GO:0008270;GO:0005525 |
| TRINITY_DN393303_c0_g1_i11_m.1874174 | Mitochondrial carnitine/acylcarnitine carrier like protein | 1.67 | 0.0017 | GO:0015822;GO:0006839;GO:0006412;GO:0016021;GO:0009507;GO:0003735 |
| TRINITY_DN565099_c0_g1_i1_m.3674562 | methylisocitrate lyase | 1.69 | 0.0036 | GO:0019629;GO:0046421;GO:0000287 |
| TRINITY_DN334568_c0_g2_i1_m.2510635 | DNA gyrase subunit A | 1.51 | 0.0096 | GO:0006265;GO:0006261;GO:0005737;GO:0005694;GO:0003677;GO:0003918;GO:0005524 |
| TRINITY_DN370888_c0_g1_i3_m.1166718 | DNA directed RNA polymerases I and III subunit RPAC2 | 1.66 | 0.0083 | GO:0006360;GO:0006383;GO:0005736;GO:0005666;GO:0001056;GO:0001054;GO:0003677;GO:0046983 |
| TRINITY_DN385695_c0_g1_i1_m.3357995 | Aspartic proteinase nepenthesin 1 | 1.47 | 0.0298 | GO:0006508;GO:0030163;GO:0004190 |
| TRINITY_DN345743_c3_g3_i2_m.3287144 | Ubiquitin conjugating enzyme E2 28 | 1.40 | 0.0276 | GO:0016043;GO:0016567;GO:0006511;GO:0044763;GO:0004842;GO:0005524 |
| TRINITY_DN397920_c4_g1_i3_m.2061483 | cytosolic glutathione reductase | 1.78 | 0.0011 | GO:0098869;GO:0022900;GO:0006749;GO:0045454;GO:0005737;GO:0050661;GO:0050660;GO:0004362 |
| TRINITY_DN395768_c0_g1_i5_m.1786031 | MAP kinase phosphatase | 1.28 | 0.0073 | GO:0010374;GO:0006888;GO:0043090;GO:0006487;GO:0035335;GO:0006499;GO:0000188;GO:0048481;GO:0009409;GO:0009651;GO:0010225;GO:0010224;GO:0005829;GO:0004725;GO:0016301;GO:0017017 |
| TRINITY_DN390361_c3_g5_i2_m.2622548 | Flavoprotein wrbA | 1.31 | 0.0009 | GO:0045892;GO:0055114;GO:0010181;GO:0016491 |
| TRINITY_DN328827_c0_g2_i3_m.2914543 | putative glycine rich protein | 1.44 | 0.0402 | GO:0006355;GO:0008270;GO:0003677 |
| TRINITY_DN385301_c0_g5_i1_m.2392061 | small nuclear ribonucleoprotein SmD3b | 1.30 | 0.0072 | GO:0000387;GO:0000245;GO:0000243;GO:0071011;GO:0071010;GO:0071013;GO:0034715;GO:0097526;GO:0019013;GO:0005689;GO:0005687;GO:0005686;GO:0005685;GO:0034719;GO:0005682;GO:0003723 |
| TRINITY_DN398801_c0_g1_i3_m.1996422 | histidine tRNA ligase | 1.26 | 0.0472 | GO:0006427;GO:0032543;GO:0009536;GO:0005829;GO:0005739;GO:0016021;GO:0004821;GO:0005524 |
| TRINITY_DN392404_c0_g1_i2_m.2439221 | peptidyl prolyl cis trans isomerase PASTICCINO1 | 1.45 | 0.0017 | GO:0000911;GO:0000413;GO:0030010;GO:0009880;GO:0009735;GO:0009826;GO:0048513;GO:0030154;GO:0010048;GO:0000226;GO:0048527;GO:0042761;GO:0061077;GO:0005829;GO:0005789;GO:0005528;GO:0003755 |
| TRINITY_DN388303_c3_g1_i11_m.1186915 | Peroxisomal multifunctional enzyme type 2 | 1.40 | 0.0216 | GO:0007031;GO:0010260;GO:0043161;GO:0033542;GO:0033387;GO:0051788;GO:0005737;GO:0080023;GO:0004586 |
| TRINITY_DN384091_c0_g1_i4_m.1264926 | gamma aminobutyrate transaminase 1 | 1.33 | 0.0169 | GO:0005739;GO:0008483;GO:0042802;GO:0030170 |
| TRINITY_DN366088_c0_g1_i3_m.944466 | tonoplast intrinsic protein 1 2 | 1.55 | 0.0002 | GO:0009992;GO:0034220;GO:0006833;GO:0015793;GO:0009705;GO:0005887;GO:0042807;GO:0015250;GO:0015254 |
| TRINITY_DN395680_c0_g2_i1_m.2555171 | periodic tryptophan protein 2 homolog | 1.36 | 0.0066 | GO:0009553;GO:0000028;GO:0000462;GO:0034388;GO:0032040;GO:0030515 |
| TRINITY_DN10588_c0_g1_i1_m.4445470 | protein transport protein Sec61 subunit alpha like | 1.47 | 0.0022 | GO:0015031;GO:0016021 |
| TRINITY_DN395732_c1_g2_i4_m.1782370 | la related protein 6B like | 1.23 | 0.0021 | GO:0006396;GO:0009536;GO:0005634;GO:0030529;GO:0000166;GO:0003723 |
| TRINITY_DN396607_c1_g1_i4_m.2177108 | E3 ubiquitin protein ligase RNF14 | 1.24 | 0.0046 | GO:0032436;GO:0042787;GO:0000209;GO:0005737;GO:0000151;GO:0031624;GO:0016874;G |

| | | | | |
|---|---|---|---|---|
| | | | | O:0008270;GO:0061630 |
| TRINITY_DN341087_c0_g1_i4_m.3335345 | Glucan endo 1,3 beta glucosidase GV | 1.35 | 0.0248 | GO:0005975;GO:0006952;GO:0005737;GO:0046658;GO:0030247;GO:0043531;GO:0042973 |
| TRINITY_DN367766_c0_g1_i8_m.1693602 | sucrose synthase type 3 | 1.42 | 0.0005 | GO:0005985;GO:0016157 |
| TRINITY_DN376831_c1_g1_i9_m.1135646 | mitochondrial pyruvate carrier 4 | 1.46 | 0.0004 | GO:0006850;GO:0005743 |
| TRINITY_DN386551_c1_g1_i12_m.2904080 | Peroxidase 5 | 1.35 | 0.0036 | GO:0009664;GO:0006979;GO:0098869;GO:0042744;GO:0055114;GO:0005576;GO:0009505;GO:0020037;GO:0046872;GO:0004601 |
| TRINITY_DN391876_c2_g1_i3_m.2605180 | succinate semialdehyde dehydrogenase | 1.33 | 0.0013 | GO:0072593;GO:0006081;GO:0006540;GO:0009450;GO:0009416;GO:0009408;GO:0055114;GO:0009570;GO:0005759;GO:0004030;GO:0051287;GO:0009013;GO:0004777;GO:0005507;GO:0004029 |
| TRINITY_DN390317_c1_g1_i12_m.2622833 | GTPase activating protein gyp1 | 1.22 | 0.0420 | GO:0031338;GO:0006886;GO:0090630;GO:0005622;GO:0012505;GO:0005096;GO:0017137 |
| TRINITY_DN397146_c2_g1_i14_m.3281829 | probable staphylococcal like nuclease CAN1 | 1.34 | 0.0053 | GO:0090305;GO:0006418;GO:0005886;GO:0004812;GO:0004519;GO:0003676;GO:0046872;GO:0005524 |
| TRINITY_DN347493_c0_g2_i1_m.3103870 | Methylcrotonoyl CoA carboxylase subunit alpha | 1.47 | 0.0025 | GO:0009744;GO:0009750;GO:0006552;GO:0022626;GO:0005759;GO:0004485;GO:0050897;GO:0004075;GO:0005524 |
| TRINITY_DN399200_c1_g1_i1_m.911796 | glutamine dependent asparagine synthetase | 1.38 | 0.0362 | GO:0006529;GO:0006541;GO:0009063;GO:0009646;GO:0005829;GO:0004066;GO:0042803 |
| TRINITY_DN382298_c0_g1_i6_m.1495078 | probable NAD kinase 1 | 1.24 | 0.0416 | GO:0016310;GO:0006741;GO:0019674;GO:0003951 |
| TRINITY_DN369089_c0_g1_i7_m.3020671 | malate dehydrogenase 1 | 1.51 | 0.0018 | GO:0005975;GO:0006099;GO:0006108;GO:0005739;GO:0009505;GO:0005886;GO:0030060 |
| TRINITY_DN375283_c0_g1_i10_m.999811 | Proteasome subunit alpha type 2 | 1.41 | 0.0011 | GO:0006511;GO:0005737;GO:0019773;GO:0005634;GO:0004298 |
| TRINITY_DN370815_c0_g3_i1_m.1169124 | Proteasome subunit alpha type 7 A | 1.35 | 0.0176 | GO:0006511;GO:0005737;GO:0019773;GO:0005634;GO:0004298 |
| TRINITY_DN393538_c2_g1_i7_m.2758034 | | 1.32 | 0.0484 | GO:0009813;GO:0052696;GO:0043231;GO:0080043;GO:0080044 |
| TRINITY_DN375358_c0_g1_i6_m.2516405 | hexokinase 2 | 1.21 | 0.0137 | GO:0046835;GO:0051156;GO:0001678;GO:0006096;GO:0005829;GO:0008865;GO:0004340;GO:0019158;GO:0005524;GO:0005536 |
| TRINITY_DN341458_c0_g1_i3_m.1467007 | phosphonate ABC transporter ATP binding protein | 4.03 | 0.0106 | GO:0015716;GO:0015748;GO:0015416;GO:0005524 |
| TRINITY_DN317504_c0_g1_i2_m.1870540 | Bacterioferritin | 3.01 | 0.0120 | GO:0006879;GO:0006826;GO:0055114;GO:0005623;GO:0004322;GO:0008199 |
| TRINITY_DN725541_c0_g1_i1_m.4301928 | Ribosomal protein 59 of the small subunit | 2.03 | 0.0065 | GO:0000028;GO:0000462;GO:0006412;GO:0022627;GO:0070181;GO:0003735;GO:0048027 |
| TRINITY_DN369279_c1_g2_i1_m.2470829 | ran specific gtpase activating protein | 2.06 | 0.0066 | GO:0006606;GO:0006511;GO:0006405;GO:0000082;GO:0005737;GO:0005634;GO:0008536 |
| TRINITY_DN359424_c2_g4_i1_m.1988494 | 40S ribosomal protein S25 | 2.38 | 0.0003 | GO:0005840 |
| TRINITY_DN363984_c2_g1_i2_m.2693919 | 60S ribosomal protein L6 | 2.34 | 0.0014 | GO:0006412;GO:0005829;GO:0005840;GO:0005730;GO:0003735 |
| TRINITY_DN398308_c5_g2_i10_m.2797875 | 50S ribosomal protein L1 | 2.00 | 0.0112 | GO:0006417;GO:0015934;GO:0003735;GO:0000049;GO:0019843 |
| TRINITY_DN243421_c0_g1_i1_m.3800879 | ATP dependent RNA helicase DDX19A | 2.06 | 0.0007 | GO:0005524;GO:0003676;GO:0004386 |
| TRINITY_DN327314_c1_g2_i1_m.1395927 | argininosuccinate synthase | 2.11 | 0.0014 | GO:0006526;GO:0005737;GO:0004055;GO:0005524 |
| TRINITY_DN355508_c0_g1_i2_m.3117014 | 60S ribosomal protein L30 | 2.28 | 0.0032 | GO:0006364;GO:0048025;GO:0006412;GO:0005840;GO:0030627;GO:0003735 |
| TRINITY_DN538966_c0_g1_i1_m.3669472 | Amidohydrolase | 2.17 | 0.0036 | GO:0016810 |
| TRINITY_DN18526_c0_g1_i1_m.4534325 | Vtype ATPase, C subunit | 1.76 | 0.0031 | GO:0015991;GO:0007035;GO:0016021;GO:0000220;GO:0046961 |
| TRINITY_DN379285_c0_g1_i7_m.2135180 | mitochondrial import inner membrane translocase subunit TIM10 | 1.65 | 0.0092 | GO:0045039;GO:0005743;GO:0005758;GO:0008565;GO:0046872 |
| TRINITY_DN108653_c0_g1_i2_m.609869 | acetyl coenzyme A synthetase | 1.98 | 0.0146 | GO:0019427;GO:0003987;GO:0016208;GO:0046872;GO:0005524 |
| TRINITY_DN882399_c0_g1_i1_m.3398292 | osmotically inducible protein OsmC | 1.80 | 0.0056 | GO:0098869;GO:0006979;GO:0004601 |
| TRINITY_DN389058_c0_g1_i1_m.2984756 | serine/arginine rich SC35 like splicing factor SCL30 | 2.04 | 0.0005 | GO:0070217;GO:0016480;GO:0005737;GO:0005634;GO:0000166;GO:0003676;GO:0000994 |
| TRINITY_DN364677_c2_g3_i7_m.2315861 | pathogenesis related protein 4 | 1.76 | 0.0033 | GO:0050832;GO:0042742 |
| TRINITY_DN573539_c0_g1_i1_m.3616686 | sucrose synthase 5 | 1.66 | 0.0108 | GO:0005985;GO:0016021;GO:0016157 |
| TRINITY_DN380703_c0_g4_i2_m.3247042 | probable sodium/metabolite cotransporter BASS2 | 2.37 | 0.0002 | GO:0019761;GO:0006849;GO:0000023;GO:0043085;GO:0019252;GO:0009941;GO:0009534;GO:0005887;GO:0015293;GO:0050833 |
| TRINITY_DN182300_c0_g1_i1_m.538169 | transcriptional regulator Crp | 1.61 | 0.0402 | GO:0006355;GO:0005622;GO:0003700;GO:0003677 |
| TRINITY_DN388801_c1_g1_i9_m.2536894 | pyrroline 5 carboxylate reductases | 1.54 | 0.0002 | GO:0055129;GO:0055114;GO:0005618;GO:0009536;GO:0004735 |

| | | | | |
|---|---|---|---|---|
| TRINITY_DN391308_c0_g3_i11_m.2956092 | receptor kinase | 1.54 | 0.0154 | GO:0006468;GO:0016021;GO:0004674;GO:0030247;GO:0005524 |
| TRINITY_DN396448_c0_g1_i10_m.2063080 | sodium coupled neutral amino acid transporter 2 like | 1.61 | 0.0012 | GO:0003333;GO:0016021;GO:0015171 |
| TRINITY_DN387004_c0_g2_i2_m.2665083 | Anthocyanidin 3 O glucosyltransferase 2 like | 1.48 | 0.0009 | GO:0009813;GO:0052696;GO:0043231;GO:0080043;GO:0080044 |
| TRINITY_DN365870_c0_g1_i2_m.1018928 | ferulic acid esterase A | 1.80 | 0.0275 | GO:0005975;GO:0006508;GO:0005576;GO:0030248;GO:0004553;GO:0008236 |
| TRINITY_DN393034_c3_g1_i3_m.1155736 | protein disulfide isomerase | 1.62 | 0.0051 | GO:0045454;GO:0034975;GO:0009960;GO:0005788;GO:0003756 |
| TRINITY_DN275969_c0_g1_i2_m.4106718 | probable methionine tRNA ligase | 1.23 | 0.0200 | GO:0006431;GO:0022900;GO:0005829;GO:0004825;GO:0005524;GO:0009055 |
| TRINITY_DN387621_c6_g3_i4_m.3107270 | mitochondrial processing peptidase alpha chain precursor | 1.54 | 0.0003 | GO:0006627;GO:0046835;GO:0051156;GO:0001678;GO:0006096;GO:0005829;GO:0005743;GO:0004222;GO:0008865;GO:0004340;GO:0019158;GO:0008270;GO:0005524;GO:0005536 |
| TRINITY_DN382107_c0_g1_i6_m.1365974 | probable galacturonosyltransferase 11 | 1.27 | 0.0331 | GO:0045489;GO:0071555;GO:0016021;GO:0000139;GO:0047262 |
| TRINITY_DN323353_c1_g2_i1_m.1831373 | calcium dependent protein kinase 26 like | 1.33 | 0.0151 | GO:0018105;GO:0009738;GO:0035556;GO:0046777;GO:0005634;GO:0005739;GO:0005886;GO:0004683;GO:0009931;GO:0005509;GO:0005524;GO:0005516 |
| TRINITY_DN382882_c2_g1_i3_m.2543690 | probable 3 beta hydroxysteroid Delt | 1.65 | 0.0010 | GO:0016126;GO:0060964;GO:0016021;GO:0005789;GO:0005886;GO:0000247;GO:0047750 |
| TRINITY_DN392428_c2_g3_i2_m.1259554 | uridine 5 monophosphate synthase | 1.32 | 0.0114 | GO:0043547;GO:0044205;GO:0006207;GO:0005096;GO:0004588;GO:0004590 |
| TRINITY_DN384839_c0_g1_i3_m.2592096 | cytosolic Cu/Zn superoxide dismutase | 1.47 | 0.0176 | GO:0019430;GO:0055114;GO:0005739;GO:0008270;GO:0004784;GO:0005507 |
| TRINITY_DN378108_c2_g1_i1_m.2684580 | Trans 2,3 enoyl CoA reductase | 1.23 | 0.0345 | GO:0016126;GO:0007030;GO:0046520;GO:0006816;GO:0010025;GO:0009651;GO:0055114;GO:0016021;GO:0009923;GO:0005886;GO:0009922;GO:0019166 |
| TRINITY_DN388638_c0_g1_i2_m.1584792 | Glucan endo 1,3 beta glucosidase 3 | 1.48 | 0.0002 | GO:0005975;GO:0046658;GO:0030247;GO:0004553 |
| TRINITY_DN394329_c2_g7_i1_m.2275197 | prohibitin 2 | 1.27 | 0.0052 | GO:0016020 |
| TRINITY_DN342090_c0_g1_i8_m.2019729 | cytochrome b c1 complex subunit 7 | 1.44 | 0.0003 | GO:1902600;GO:0006122;GO:0009060;GO:0034551;GO:0009536;GO:0005750;GO:0008121 |
| TRINITY_DN392960_c6_g4_i2_m.2522403 | beta 1,3 glucanase | 1.62 | 0.0018 | GO:0005975;GO:0046658;GO:0030247;GO:0004553 |
| TRINITY_DN376801_c1_g3_i4_m.2857303 | protein RER1B | 1.29 | 0.0008 | GO:0006890;GO:0016021;GO:0005622 |
| TRINITY_DN311563_c0_g1_i1_m.1753174 | Geranylgeranyl transferase type 2 subunit alpha | 1.28 | 0.0082 | GO:0018344;GO:0005968;GO:0004663 |
| TRINITY_DN349764_c0_g1_i1_m.2373307 | L type lectin domain containing receptor kinase IV.1 nitrogen regulatory protein P | 1.42 | 0.0022 | GO:0006468;GO:0016021;GO:0004674;GO:0030246;GO:0005524 |
| TRINITY_DN369006_c0_g1_i11_m.3018241 | nitrogen regulatory protein P II homolog | 1.41 | 0.0030 | GO:0042325;GO:0009718;GO:0050790;GO:0006355;GO:0042304;GO:0006808;GO:0009534;GO:0005829;GO:0000287;GO:0010307;GO:0005524 |
| TRINITY_DN397485_c0_g1_i3_m.1058220 | Cleavage and polyadenylation specificity factor subunit 3 | 1.23 | 0.0005 | GO:0005634 |
| TRINITY_DN379685_c0_g1_i4_m.1593759 | pentatricopeptide repeat containing protein At1g60770 | 1.20 | 0.0180 | GO:0005739 |
| TRINITY_DN368264_c0_g1_i5_m.1448508 | ER membrane protein complex subunit 3 | 1.40 | 0.0055 | GO:0016049;GO:0034975;GO:0000902;GO:0072546;GO:0005739 |
| TRINITY_DN359781_c0_g1_i2_m.1514591 | protein argonaute 1B isoform X1 | 1.28 | 0.0259 | GO:0031047;GO:0003676 |
| TRINITY_DN383727_c1_g1_i2_m.1705455 | indole 3 acetaldehyde oxidase like | 1.41 | 0.0030 | GO:0009688;GO:0009851;GO:0009115;GO:0055114;GO:0005829;GO:0043546;GO:0016614;GO:0051537;GO:0071949;GO:0004854;GO:0005506;GO:0050302;GO:0009055 |
| TRINITY_DN343950_c0_g1_i2_m.2625884 | RNA recognition motif domain containing protein | 1.20 | 0.0045 | GO:0000398;GO:0017069;GO:0000166 |
| TRINITY_DN391235_c0_g1_i1_m.2774401 | peptidyl prolyl cis trans isomerase CYP59 | 1.25 | 0.0086 | GO:0000413;GO:0006457;GO:0003676;GO:0000166;GO:0008270;GO:0003755 |
| TRINITY_DN390760_c1_g2_i4_m.1675428 | Secologanin synthase | 1.55 | 0.0483 | GO:0055114;GO:0004497;GO:0016705;GO:0005506;GO:0020037 |
| TRINITY_DN385759_c1_g2_i5_m.1144187 | sugar transport protein 14 | 1.39 | 0.0299 | GO:0015992;GO:0046323;GO:1904659;GO:0005887;GO:0005355;GO:0005351 |
| TRINITY_DN391867_c0_g4_i1_m.2604824 | Squamous cell carcinoma antigen recognized by T cells 3 | 1.23 | 0.0375 | GO:0009560;GO:0006397;GO:0005634;GO:0000166;GO:0003676 |
| TRINITY_DN518207_c0_g1_i1_m.3724539 | acetyl CoA carboxylase biotin carboxylase subunit | 6.06 | 0.0348 | GO:0004075;GO:0046872;GO:0005524;GO:0003989 |
| TRINITY_DN905798_c0_g1_i1_m.286776 | chaperone protein dnaJ 13 | 6.48 | 0.0002 | GO:0055122;GO:0010228;GO:0016021;GO:0009536 |
| TRINITY_DN301902_c0_g2_i1_m.1302254 | peptidyl prolyl cis trans isomerase | 4.23 | 0.0160 | GO:0000413;GO:0006457;GO:0003755 |
| TRINITY_DN388501_c0_g1_i3_m.2462822 | sucrose synthase | 2.77 | 0.0058 | GO:0005985;GO:0016157 |
| TRINITY_DN323871_c2_g1_i3_m.1481350 | ketol acid reductoisomerase | 4.09 | 0.0003 | GO:0009097;GO:0055114;GO:0009099;GO:0004455;GO:0016853 |
| TRINITY_DN244469_c0_g3_i1_m.4041162 | electron transfer flavoprotein subunit beta | 2.39 | 0.0059 | GO:0050660;GO:0009055 |
| TRINITY_DN221649_c0_g1_i2_m.4040849 | molybdenum cofactor biosynthesis protein | 3.79 | 0.0105 | GO:0006777 |

| | | | | |
|---|---|---|---|---|
| TRINITY_DN974725_c0_g1_i1_m.229849 | UDP 3 O [3 hydroxymyristoyl] N acetylglucosamine deacetylase | 2.08 | 0.0144 | GO:0009245;GO:0046872;GO:0016746;GO:0103117;GO:0008759 |
| TRINITY_DN178778_c0_g1_i2_m.529235 | flagellar motor protein MotB | 3.17 | 0.0001 | GO:0016020 |
| TRINITY_DN152553_c0_g1_i1_m.583868 | protease modulator HflK | 3.04 | 0.0020 | GO:0006508;GO:0016021;GO:0008233 |
| TRINITY_DN291138_c0_g1_i1_m.3801372 | aconitate hydratase B | 2.21 | 0.0025 | GO:0006099;GO:0005829;GO:0051539;GO:0047456;GO:0003994;GO:0046872 |
| TRINITY_DN369495_c1_g1_i1_m.1468840 | ammonium transporter 1 member 1 | 2.83 | 0.0018 | GO:0019740;GO:0015695;GO:0072488;GO:0005887;GO:0008519 |
| TRINITY_DN587219_c0_g1_i1_m.3752731 | translation elongation factor TU | 1.85 | 0.0038 | GO:0006414;GO:0005737;GO:0003746;GO:0003924;GO:0005525 |
| TRINITY_DN262848_c1_g1_i1_m.4108185 | enoyl ACP reductase | 2.30 | 0.0009 | GO:0006633;GO:0055114;GO:0004318 |
| TRINITY_DN361228_c0_g1_i2_m.1860987 | mitochondrial import inner membrane translocase subunit TIM23 1 | 1.91 | 0.0048 | GO:0030150;GO:0031305;GO:0005744;GO:0015266 |
| TRINITY_DN398826_c1_g2_i4_m.1563885 | phosphate transporter | 1.77 | 0.0067 | GO:0006817;GO:0055085;GO:0016021;GO:0009536;GO:0005315 |
| TRINITY_DN391110_c2_g1_i16_m.2102147 | 2 oxoglutarate dehydrogenase subunit E1 | 2.01 | 0.0025 | GO:0006099;GO:0004591;GO:0030976 |
| TRINITY_DN383010_c2_g1_i9_m.2879560 | Glucan endo 1,3 beta glucosidase | 1.77 | 0.0109 | GO:0006952;GO:0005975;GO:0046658;GO:0030247;GO:0042973 |
| TRINITY_DN232991_c0_g1_i2_m.3880774 | elongation factor P | 2.22 | 0.0000 | GO:0006414;GO:0005737;GO:0003746 |
| TRINITY_DN390506_c5_g1_i8_m.1824882 | 20 alpha hydroxysteroid dehydrogenase | 1.85 | 0.0281 | GO:0042843;GO:0055114;GO:0047006;GO:0050571 |
| TRINITY_DN768992_c0_g1_i1_m.4303658 | biopolymer transporter TonB | 1.61 | 0.0386 | GO:0015031;GO:0016021;GO:0008565 |
| TRINITY_DN399491_c4_g1_i1_m.1283504 | chemotaxis protein CheA | 1.66 | 0.0084 | GO:0018106;GO:0006935;GO:0000160;GO:0023014;GO:0005737;GO:0005524;GO:0000155 |
| TRINITY_DN793261_c0_g1_i1_m.4287420 | peptidase S41 | 1.90 | 0.0010 | GO:0006508;GO:0008236 |
| TRINITY_DN302256_c0_g1_i1_m.2922035 | soluble pyridine nucleotide transhydrogenase | 2.18 | 0.0019 | GO:0006739;GO:1902600;GO:0045454;GO:0055114;GO:0005737;GO:0050660;GO:0003957 |
| TRINITY_DN344255_c1_g1_i2_m.3286662 | trypsin | 1.70 | 0.0491 | GO:0006508;GO:0005576;GO:0004252 |
| TRINITY_DN369485_c5_g2_i6_m.1469095 | 14 3 3 family protein | 1.83 | 0.0121 | GO:0055114;GO:0004497;GO:0019904 |
| TRINITY_DN388119_c2_g1_i3_m.1810263 | syntaxin 43 like | | | GO:0006886;GO:0006906;GO:0048278;GO:0016021;GO:0012505;GO:0031201;GO:0000149;G |
| | | 1.77 | 0.0001 | O:0005484 |
| TRINITY_DN393707_c1_g3_i6_m.3184328 | apocytochrome b | 1.79 | 0.0000 | GO:1902600;GO:0006122;GO:0045275;GO:0005743;GO:0008121;GO:0046872;GO:0009055 |
| TRINITY_DN396008_c5_g1_i9_m.1775281 | tetraspanin 8 like | 1.30 | 0.0289 | GO:0016021 |
| TRINITY_DN383240_c4_g1_i10_m.3052908 | hydroxyanthranilate hydroxycinnamoyltransferase 1 | 1.31 | 0.0188 | GO:0005739;GO:0016747 |
| TRINITY_DN376808_c1_g2_i8_m.1136538 | actin depolymerization factor like protein | 1.33 | 0.0106 | GO:0030042;GO:0015629;GO:0003779 |
| TRINITY_DN386558_c1_g2_i4_m.2901370 | methylcrotonoyl CoA carboxylase beta chain | 1.35 | 0.0199 | GO:0010050;GO:0006552;GO:0005739;GO:0050897;GO:0008270;GO:0016874 |
| TRINITY_DN387720_c1_g1_i5_m.3236536 | mitochondrial adenine nucleotide transporter ADNT1 | 1.40 | 0.0003 | GO:0055085;GO:0006412;GO:0016021;GO:0005634;GO:0003735 |
| TRINITY_DN51780_c0_g1_i1_m.4463152 | phosphoenolpyruvate carboxykinase | 1.49 | 0.0007 | GO:0016310;GO:0006094;GO:0005737;GO:0016301;GO:0004612;GO:0046872;GO:0005524 |
| TRINITY_DN359969_c2_g3_i4_m.2682956 | tonoplast intrinsic protein 1 1 | | | GO:0009992;GO:0034220;GO:0006833;GO:0015793;GO:0009705;GO:0005887;GO:0042807;G |
| | | 1.78 | 0.0110 | O:0015250;GO:0015254 |
| TRINITY_DN365230_c2_g1_i4_m.2408520 | Aquaporin PIP1 5 | | | GO:0009992;GO:0034220;GO:0006833;GO:0015793;GO:0005773;GO:0005887;GO:0009506;G |
| | | 1.46 | 0.0014 | O:0015250;GO:0015254 |
| TRINITY_DN398341_c0_g1_i2_m.2795683 | translin | 1.41 | 0.0008 | GO:0005634;GO:0043565 |
| TRINITY_DN365605_c0_g2_i6_m.1184177 | phenylalanine ammonia lyase | 1.23 | 0.0414 | GO:0009800;GO:0006559;GO:0005737;GO:0045548 |
| TRINITY_DN392428_c2_g1_i1_m.1259539 | Uridine 5' monophosphate synthase | | | GO:0006468;GO:0006207;GO:0044205;GO:0006508;GO:0005829;GO:0004588;GO:0004672;G |
| | | 1.31 | 0.0156 | O:0004590;GO:0008234;GO:0005524 |
| TRINITY_DN324788_c0_g1_i1_m.2017128 | Plastidic ATP/ADP transporter | 1.41 | 0.0018 | GO:0015866;GO:0015867;GO:0009536;GO:0016021;GO:0005471;GO:0005524 |
| TRINITY_DN369472_c0_g1_i2_m.1469400 | hexokinase 9 like | | | GO:0046835;GO:0051156;GO:0001678;GO:0006096;GO:0009527;GO:0005829;GO:0009507;G |
| | | 1.27 | 0.0479 | O:0046872;GO:0008865;GO:0004340;GO:0019158;GO:0005536;GO:0005524 |
| TRINITY_DN362334_c1_g1_i2_m.1737402 | protein disulfide isomerase like 5 1 wild type haplotype I | 1.23 | 0.0031 | GO:0045454;GO:0006457;GO:0034976;GO:0005783;GO:0003756 |
| TRINITY_DN396146_c2_g3_i2_m.3078273 | AF467542_1 putative aldehyde dehydrogenase WIS1 | | | GO:0072593;GO:0010133;GO:0055114;GO:0005739;GO:0009507;GO:0004028;GO:0004029;G |
| | | 1.35 | 0.0050 | O:0050897;GO:0003842;GO:0008270 |
| TRINITY_DN397558_c0_g1_i7_m.2298566 | xanthine dehydrogenase | | | GO:0009115;GO:0055114;GO:0005829;GO:0050660;GO:0016614;GO:0051537;GO:0016903;G |
| | | 1.34 | 0.0029 | O:0004854;GO:0005506;GO:0009055 |

| | | | | |
|---|---|---|---|---|
| TRINITY_DN399201_c1_g1_i5_m.916392 | probable serine protease EDA2 | 1.26 | 0.0206 | GO:0006508;GO:0016021;GO:0008239;GO:0004185 |
| TRINITY_DN399755_c4_g1_i11_m.3141259 | internal alternative NA | 1.25 | 0.0055 | GO:0055114;GO:0005739;GO:0016491 |
| TRINITY_DN559761_c0_g1_i1_m.3667100 | Probable cytoplasmic aconitate hydratase | 1.23 | 0.0022 | GO:0008152;GO:0003994;GO:0051539 |
| TRINITY_DN380857_c0_g1_i11_m.1349355 | ubiquitin carboxyl terminal hydrolase 6 | 1.27 | 0.0098 | GO:0006511;GO:0016579;GO:0036459 |
| TRINITY_DN214454_c0_g1_i1_m.4026166 | histone H3 like | 1.28 | 0.0028 | GO:0006334;GO:0006096;GO:0000786;GO:0005634;GO:0003677;GO:0004619;GO:0046982 |
| TRINITY_DN323215_c0_g1_i2_m.2995889 | Clathrin heavy chain 1 | 1.21 | 0.0119 | GO:0006886;GO:0016192;GO:0030130;GO:0030132;GO:0005198 |
| TRINITY_DN398030_c3_g1_i2_m.953129 | beta glucosidase 4 | 1.30 | 0.0154 | GO:1901657;GO:0030245;GO:0005829;GO:0102483;GO:0008422 |
| TRINITY_DN366745_c0_g1_i6_m.2326147 | UDP galactose:fucoside alpha 3 galactosyltransferase | 1.21 | 0.0049 | GO:0071555;GO:0016021;GO:0000139;GO:0016757 |
| TRINITY_DN395110_c0_g1_i8_m.2843907 | cytochrome b561, DM13 and DOMON domain containing protein At5g54830 | 1.27 | 0.0426 | GO:0055114;GO:0016021 |
| TRINITY_DN384843_c0_g1_i4_m.2592006 | thiamine thiazole synthase 2 | 1.28 | 0.0422 | GO:0055114;GO:0010155;GO:0009228;GO:0042742;GO:0009637;GO:0046777;GO:0006974;GO:0052837;GO:0000023;GO:0043085;GO:0019252;GO:0009941;GO:0009570;GO:0010319;GO:0009579;GO:0005829;GO:0016491;GO:0008270 |
| TRINITY_DN364974_c0_g2_i3_m.3336554 | heme peroxidase | 3.46 | 0.0210 | GO:0006979;GO:0098869;GO:0055114;GO:0016021;GO:0020037;GO:0004601 |
| TRINITY_DN338942_c0_g2_i1_m.1637329 | zinc dependent alcohol dehydrogenase | 2.55 | 0.0184 | GO:0008643;GO:0055114;GO:0008270;GO:0016491 |
| TRINITY_DN349925_c2_g3_i1_m.1338358 | transcription termination/antitermination protein NusA | 3.17 | 0.0001 | GO:0031564;GO:0006414;GO:0005737;GO:0003700;GO:0000166;GO:0003746 |
| TRINITY_DN351305_c0_g1_i12_m.2091547 | Putative xylosidase/arabinosidase | 2.48 | 0.0107 | GO:0005975;GO:0009044 |
| TRINITY_DN390708_c0_g1_i2_m.1671719 | Ribosome inactivating protein 9 | 1.83 | 0.0245 | GO:0016787 |
| TRINITY_DN395027_c0_g1_i8_m.2282867 | Sugar transport protein 1 | 2.11 | 0.0004 | GO:0015992;GO:0046323;GO:1904659;GO:0005887;GO:0005355;GO:0005351 |
| TRINITY_DN399858_c4_g4_i1_m.2043153 | 30S ribosomal protein S7 | 2.55 | 0.0028 | GO:0006412;GO:0015935;GO:0003735;GO:0000049;GO:0019843 |
| TRINITY_DN195888_c0_g1_i1_m.616367 | 50S ribosomal protein L31 | 1.90 | 0.0214 | GO:0006412;GO:0005840;GO:0003735 |
| TRINITY_DN355426_c0_g1_i1_m.2826318 | Glycosyl hydrolase family 61 | 2.18 | 0.0029 | GO:0005975;GO:0005576;GO:0016162;GO:0008810;GO:0030248 |
| TRINITY_DN321878_c0_g1_i1_m.2443346 | phosphoenolpyruvate synthase | 2.05 | 0.0043 | GO:0006090;GO:0016310;GO:0006094;GO:0008986;GO:0046872;GO:0005524 |
| TRINITY_DN359430_c0_g1_i7_m.1988598 | mitochondrial pyruvate carrier 1 | 1.70 | 0.0002 | GO:0006850;GO:0005743;GO:0016021 |
| TRINITY_DN393731_c4_g1_i7_m.3182505 | Catalase isozyme 2 | 1.39 | 0.0213 | GO:0042542;GO:0098869;GO:0042744;GO:0055114;GO:0009514;GO:0020037;GO:0004096;GO:0046872 |
| TRINITY_DN247115_c0_g1_i1_m.3778585 | 30S ribosomal protein S15 | 1.80 | 0.0059 | GO:0006412;GO:0005840;GO:0003735;GO:0019843 |
| TRINITY_DN382119_c3_g1_i6_m.1369396 | Adipocyte plasma membrane associated protein | 1.56 | 0.0031 | GO:0009058;GO:0005783;GO:0016021;GO:0005739;GO:0016844;GO:0016788 |
| TRINITY_DN325433_c0_g1_i1_m.2212558 | bifunctional aconitate hydratase 2 and 2 methylisocitrate dehydratase | 1.89 | 0.0347 | GO:0006099;GO:0005829;GO:0051539;GO:0047456;GO:0003994;GO:0046872 |
| TRINITY_DN380648_c0_g3_i4_m.3244965 | Protein vip1 | 1.50 | 0.0064 | GO:0003676;GO:0000166 |
| TRINITY_DN214207_c0_g1_i1_m.3927010 | aconitate hydratase 2 | 1.55 | 0.0317 | GO:0006099;GO:0005829;GO:0051539;GO:0047456;GO:0003994;GO:0046872 |
| TRINITY_DN395447_c0_g1_i11_m.2726789 | High affinity cationic amino acid transporter 1 | 1.45 | 0.0006 | GO:1902475;GO:0005739;GO:0005887;GO:0015297;GO:0015179 |
| TRINITY_DN392171_c0_g1_i9_m.2203412 | DNA topoisomerase 1 | 1.43 | 0.0259 | GO:0006338;GO:0006265;GO:0006260;GO:0007059;GO:0005730;GO:0031298;GO:0003917;GO:0003677;GO:0003918 |
| TRINITY_DN359386_c1_g1_i5_m.2480159 | triose phosphate isomerase | 1.48 | 0.0003 | GO:0006096;GO:0006098;GO:0006094;GO:0005739;GO:0004807 |
| TRINITY_DN390163_c0_g1_i5_m.2088964 | 1,4 alpha D glucan maltohydrolase，Beta amylase | 1.53 | 0.0325 | GO:0000272;GO:0016161 |
| TRINITY_DN237824_c0_g1_i1_m.3853196 | glutamine synthetase | 1.27 | 0.0246 | GO:0009399;GO:0006542;GO:0005737;GO:0004356;GO:0005524 |
| TRINITY_DN398609_c1_g1_i2_m.2657061 | calcium transporting ATPase 4 | 1.34 | 0.0017 | GO:0098655;GO:0005783;GO:0005887;GO:0019829;GO:0015662;GO:0046872;GO:0005524 |
| TRINITY_DN384780_c1_g2_i18_m.1761099 | somatic embryogenesis receptor kinase 1 isoform X1 | 1.35 | 0.0057 | GO:0016310;GO:0016301 |
| TRINITY_DN376678_c0_g1_i1_m.1940265 | mitochondrial dicarboxylate/tricarboxylate transporter DTC like | 1.47 | 0.0001 | GO:0006835;GO:0035674;GO:0006412;GO:0009941;GO:0005618;GO:0016021;GO:0005739;GO:0005774;GO:0009506;GO:0005310;GO:0003735;GO:0015142 |
| TRINITY_DN397541_c1_g3_i1_m.2299342 | cytosolic phosphoglucose isomerase | 1.32 | 0.0174 | GO:0006096;GO:0006094;GO:0004347 |
| TRINITY_DN372022_c3_g1_i4_m.2897774 | beta galactosidase | 4.56 | 0.0237 | GO:1901657;GO:0030245;GO:0008422 |

| | | | | |
|---|---|---|---|---|
| TRINITY_DN347888_c2_g1_i1_m.2386817 | 50s ribosomal protein l14e | 3.11 | 0.0000 | GO:0006412;GO:0005634;GO:0005829;GO:0005840;GO:0003735 |
| TRINITY_DN306151_c1_g1_i1_m.1360498 | branched chain alpha keto acid dehydrogenase subunit E2 | 2.65 | 0.0006 | GO:0044781;GO:0071973;GO:0005576;GO:0009420;GO:0005198 |
| TRINITY_DN55044_c0_g1_i1_m.4397729 | dihydrolipoamide dehydrogenase | 2.46 | 0.0006 | GO:0045454;GO:0006096;GO:0055114;GO:0005623;GO:0050660;GO:0004148 |
| TRINITY_DN366601_c0_g2_i2_m.1117656 | UFG1 | 1.77 | 0.0043 | GO:0016021 |
| TRINITY_DN395628_c2_g3_i4_m.2553628 | Heat shock protein Hsp88 | 2.44 | 0.0405 | GO:0005829;GO:0005634;GO:0005524 |
| TRINITY_DN366187_c0_g1_i2_m.2365772 | Ras related protein RHA1 | 1.38 | 0.0005 | GO:0007264;GO:0030139;GO:0005768;GO:0005525 |
| TRINITY_DN398889_c3_g4_i1_m.1559557 | 40S ribosomal protein S27 | 1.36 | 0.0042 | GO:0000028;GO:0006412;GO:0022627;GO:0003735;GO:0046872 |
| TRINITY_DN390036_c2_g4_i1_m.1199787 | protein ENHANCED DISEASE RESISTANCE 2 like | 1.31 | 0.0042 | GO:0008289 |
| TRINITY_DN371213_c1_g1_i5_m.1386572 | protein YIPF1 homolog | 1.59 | 0.0005 | GO:0016021 |
| TRINITY_DN398943_c3_g4_i2_m.1281033 | cytochrome c oxidase subunit 2 | 1.25 | 0.0115 | GO:1902600;GO:0022900;GO:0016021;GO:0005743;GO:0070469;GO:0004129;GO:0005507 |
| TRINITY_DN345172_c0_g1_i1_m.2267630 | 6 phosphofructokinase 2 | 1.28 | 0.0121 | GO:0006002;GO:0061615;GO:0009536;GO:0005524;GO:0003872;GO:0046872 |
| TRINITY_DN376116_c0_g1_i6_m.1049287 | dnaJ protein P58IPK homolog isoform X1 | 1.21 | 0.0175 | GO:0044794;GO:0005788;GO:0005886 |
| TRINITY_DN383930_c1_g1_i16_m.2787009 | signal peptidase complex catalytic subunit SEC11A | 1.27 | 0.0002 | GO:0006465;GO:0016021;GO:0008233 |
| TRINITY_DN371931_c0_g1_i10_m.2919356 | peroxisomal membrane protein 11 5 | 1.40 | 0.0034 | GO:0044375;GO:0016559;GO:0005779 |
| TRINITY_DN399012_c4_g2_i21_m.2652813 | probable acyl activating enzyme 18 | 1.26 | 0.0099 | GO:0008152;GO:0016021;GO:0003824 |
| TRINITY_DN392406_c1_g1_i6_m.1256275 | mitochondrial processing peptidase subunit alpha like | 1.37 | 0.0007 | GO:0006627;GO:0006002;GO:0061615;GO:0005743;GO:0004222;GO:0005524;GO:0008270;GO:0003872 |
| TRINITY_DN392774_c0_g1_i11_m.903916 | L lactate dehydrogenase B | 1.27 | 0.0176 | GO:0006096;GO:0055114;GO:0005829;GO:0005886;GO:0004459 |
| TRINITY_DN395473_c6_g1_i3_m.2725524 | pyrophosphate energized membrane proton pump 3 | 1.66 | 0.0171 | GO:0015992;GO:0055085;GO:0016021;GO:0009678;GO:0004427 |
| TRINITY_DN362714_c0_g1_i1_m.2587596 | cytochrome c4 | 2.43 | 0.0413 | GO:0055114;GO:0042597;GO:0005506;GO:0020037;GO:0009055 |
| TRINITY_DN395459_c6_g10_i2_m.2724387 | 6 phosphogluconate dehydrogenase | 2.18 | 0.0065 | GO:0009051;GO:0055114;GO:0005829;GO:0050661;GO:0004616 |
| TRINITY_DN922163_c0_g1_i1_m.295010 | RNA binding protein Hfq | 1.66 | 0.0220 | GO:0006355;GO:0003723 |
| TRINITY_DN380511_c0_g1_i16_m.2740531 | Lysyl tRNA synthetase | 1.98 | 0.0070 | GO:0006430;GO:0005737;GO:0004824;GO:0005524;GO:0003676 |
| TRINITY_DN382781_c0_g1_i4_m.3062220 | Flavonol 4' sulfotransferase | 2.38 | 0.0131 | GO:0008146 |
| TRINITY_DN356214_c2_g1_i10_m.874721 | beta hydroxydecanoyl ACP dehydratase | 1.65 | 0.0040 | GO:0006636;GO:0005737;GO:0047451;GO:0008693;GO:0034017 |
| TRINITY_DN390483_c0_g2_i2_m.3225540 | enoyl [acyl carrier protein] reductase [NADH] | 1.21 | 0.0018 | GO:0006633;GO:0055114;GO:0009507;GO:0004318 |
| TRINITY_DN377614_c0_g1_i4_m.3219945 | Anthocyanidin 3 O glucosyltransferase 1 | 1.24 | 0.0028 | GO:0009813;GO:0052696;GO:0043231;GO:0080043;GO:0080044 |
| TRINITY_DN385088_c0_g2_i1_m.2191883 | protein cornichon homolog 4 | 1.24 | 0.0175 | GO:0006810;GO:0016021 |
| TRINITY_DN370252_c0_g1_i7_m.2483572 | Formate dehydrogenase | 1.27 | 0.0024 | GO:0042183;GO:0009070;GO:0055114;GO:0005739;GO:0051287;GO:0008863;GO:0004617 |
| TRINITY_DN367049_c0_g1_i8_m.1969980 | GDSL esterase/lipase At5g62930 | 1.23 | 0.0068 | GO:0005829;GO:0016788 |
| TRINITY_DN370815_c0_g1_i2_m.1169118 | proteasome subunit alpha type 7 B | 1.36 | 0.0093 | GO:0006511;GO:0005737;GO:0019773;GO:0005634;GO:0004298 |
| TRINITY_DN340655_c0_g2_i4_m.2751095 | 5 methyltetrahydropteroyltriglutamate homocysteine methyltransferase | 1.63 | 0.0026 | GO:0032259;GO:0019280;GO:0046084;GO:0005829;GO:0005634;GO:0008270;GO:0003871 |
| TRINITY_DN373530_c1_g2_i6_m.1435364 | signal recognition particle receptor subunit alpha | 1.36 | 0.0337 | GO:0006614;GO:0005785;GO:0005047;GO:0003924;GO:0005525 |
| TRINITY_DN396279_c1_g4_i12_m.1711378 | fumarylacetoacetase | 1.53 | 0.0002 | GO:0006572;GO:0008219;GO:1902000;GO:0005829;GO:0004334 |
| TRINITY_DN388927_c0_g1_i6_m.1608526 | imidazoleglycerol phosphate dehydratase | 1.45 | 0.0090 | GO:0000105;GO:0009536;GO:0004424 |
| TRINITY_DN394125_c2_g2_i1_m.1898221 | 22.3 kDa class VI heat shock protein | 1.45 | 0.0369 | GO:0009408;GO:0009644;GO:0042542;GO:0005739 |
| TRINITY_DN397562_c4_g5_i1_m.2294644 | Putative gibberellin receptor GID1L3 | 1.38 | 0.0068 | GO:0008152;GO:0016787 |
| TRINITY_DN351445_c0_g1_i1_m.2840086 | argininosuccinate lyase | 1.40 | 0.0112 | GO:0006526;GO:0005737;GO:0016829 |
| TRINITY_DN389770_c0_g1_i4_m.993871 | dihydrolipoyl dehydrogenase 2 | 1.85 | 0.0006 | GO:0007020;GO:0031122;GO:0045454;GO:0055114;GO:0009536;GO:0000930;GO:0005874;GO:0003924;GO:0050660;GO:0004148;GO:0005525 |
| TRINITY_DN395530_c3_g1_i8_m.3040712 | UDP rhamnose:rhamnosyltransferase 1 | 1.23 | 0.0385 | GO:0009813;GO:0052696;GO:0043231;GO:0080043;GO:0080044 |
| TRINITY_DN382232_c0_g1_i16_m.1493987 | probable UMP/CMP kinase 2 | 1.24 | 0.0319 | GO:0006221;GO:0006207;GO:0046939;GO:0006354;GO:0005634;GO:0005739;GO:0004127;GO:0009041;GO:0005524 |

| | | | | |
|---|---|---|---|---|
| TRINITY_DN381461_c0_g2_i11_m.1250805 | Voltage dependent anion channel | 1.52 | 0.0001 | GO:1903959;GO:0005741;GO:0046930;GO:0015288;GO:0008308 |
| TRINITY_DN398476_c3_g1_i2_m.2870585 | protein CutA 1 | 1.43 | 0.0053 | GO:0048573;GO:0070207;GO:0010038;GO:0016021;GO:0009507;GO:0005507 |
| TRINITY_DN394759_c4_g1_i3_m.2628906 | Cysteine synthase | 1.40 | 0.0000 | GO:0006535;GO:0005739;GO:0016740;GO:0030170;GO:0004124 |
| TRINITY_DN393449_c3_g2_i15_m.1692264 | UDP glycosyltransferase 85A3 | 1.21 | 0.0451 | GO:0033587;GO:0052696;GO:0009423;GO:0009813;GO:0009536;GO:0080043;GO:0080044 |
| TRINITY_DN398315_c1_g2_i2_m.2796625 | Two pore calcium channel protein 1 | 1.32 | 0.0047 | GO:0007033;GO:0052543;GO:0009556;GO:0080141;GO:0019722;GO:0070588;GO:0007030;GO:0006952;GO:0010119;GO:0034765;GO:0009651;GO:0086010;GO:0009845;GO:0000325;GO:0005794;GO:0005886;GO:0016021;GO:0005509;GO:0005245 |
| TRINITY_DN378579_c0_g1_i2_m.3215202 | probable membrane associated 30 kDa protein | 1.42 | 0.0130 | GO:0016050;GO:0016032;GO:0010027;GO:0009535;GO:0009570;GO:0016021;GO:0009706;GO:0009508;GO:0005829 |
| TRINITY_DN1001_c0_g1_i1_m.4571486 | bifunctional 3 demethylubiquinone 3 O methyltransferase/2 octaprenyl 6 hydroxy phenol methylase | 2.08 | 0.0078 | GO:0006744;GO:0032259;GO:0008689;GO:0102004;GO:0008425 |
| TRINITY_DN386500_c4_g2_i19_m.1442740 | phosphoglycerate kinase | 1.44 | 0.0060 | GO:0006096;GO:0009570;GO:0005829;GO:0005634;GO:0005774;GO:0048046;GO:0009506;GO:0005886;GO:0004618;GO:0005524 |
| TRINITY_DN380447_c0_g3_i4_m.1294613 | Putative selenium binding protein | 1.26 | 0.0124 | GO:0008430 |
| TRINITY_DN389412_c0_g1_i4_m.1813610 | Sorting and assembly machinery component 50 A like protein | 1.20 | 0.0060 | GO:0016021;GO:0019867 |
| TRINITY_DN366008_c0_g1_i4_m.944331 | calcium binding protein CML13 | 1.20 | 0.0203 | GO:0007067;GO:0051301;GO:0005737;GO:0005815;GO:0005509;GO:0003676 |
| TRINITY_DN441989_c0_g1_i1_m.665861 | putative 40s ribosomal protein s1 | 1.49 | 0.0264 | GO:0007264;GO:0006412;GO:0022627;GO:0003735;GO:0005525 |
| TRINITY_DN399715_c4_g2_i1_m.3143215 | phosphatidate cytidylyltransferase 1 like | 1.30 | 0.0120 | GO:0006655;GO:0016024;GO:0016021;GO:0005789;GO:0004605 |
| TRINITY_DN399547_c5_g1_i14_m.2999804 | calcium dependent protein kinase 7 like | 1.25 | 0.0091 | GO:0018105;GO:0009738;GO:0035556;GO:0046777;GO:0005737;GO:0005634;GO:0005886;GO:0004683;GO:0009931;GO:0005509;GO:0005524;GO:0005516 |
| TRINITY_DN310692_c2_g1_i1_m.1598229 | thiamine thiazole synthase 1 | 1.36 | 0.0189 | GO:0009228;GO:0006950;GO:0052837;GO:0055114;GO:0009570;GO:0005829;GO:0046872;GO:0016491 |
| TRINITY_DN397803_c1_g1_i6_m.2922870 | Proteasome subunit alpha type 5 | 1.24 | 0.0197 | GO:0006511;GO:0005737;GO:0019773;GO:0005634;GO:0004298 |
| TRINITY_DN365396_c0_g1_i2_m.1112239 | probable prefoldin subunit 3 | 1.27 | 0.0223 | GO:0007021;GO:0007017;GO:0006457;GO:0005844;GO:0016272;GO:0005829;GO:0009536;GO:0015631 |
| TRINITY_DN397833_c2_g2_i3_m.1946147 | 6 phosphofructokinase 3 | 1.21 | 0.0390 | GO:0006002;GO:0006508;GO:0061615;GO:0005737;GO:0004222;GO:0003872;GO:0046872;GO:0005524 |
| TRINITY_DN49355_c0_g1_i1_m.4554508 | iron ABC transporter substrate binding protein | 1.68 | 0.0451 | ------ |
| TRINITY_DN444123_c0_g1_i1_m.791332 | 14 3 3 protein 6 like | 1.95 | 0.0012 | ------ |
| TRINITY_DN394726_c1_g1_i6_m.2630438 | THO complex subunit 5A like isoform X1 | 1.23 | 0.0230 | ------ |
| TRINITY_DN289482_c0_g1_i1_m.4060641 | cell surface protein | 4.07 | 0.0024 | ------ |
| TRINITY_DN385076_c1_g2_i1_m.2193347 | Universal stress protein A like protein | 2.17 | 0.0000 | ------ |
| TRINITY_DN49365_c0_g1_i1_m.4554557 | NADH dehydrogenase [ubiquinone] 1 beta subcomplex subunit 10 B | 1.73 | 0.0001 | ------ |
| TRINITY_DN370665_c0_g1_i12_m.2217018 | HMG1/2 like protein | 1.60 | 0.0038 | ------ |
| TRINITY_DN383294_c2_g2_i11_m.3050402 | PR17d precursor | 2.17 | 0.0076 | ------ |
| TRINITY_DN53480_c0_g1_i1_m.4483409 | phospholipid binding protein | 1.48 | 0.0022 | ------ |
| TRINITY_DN390153_c0_g2_i12_m.2090886 | LEA 3 protein | 2.55 | 0.0003 | ------ |
| TRINITY_DN348486_c0_g1_i2_m.2982925 | carbonyl reductase [NADPH] 2 | 1.42 | 0.0252 | ------ |
| TRINITY_DN299195_c0_g1_i1_m.3907415 | ubiquinol cytochrome c reductase complex assembly factor 1 isoform X1 | 2.45 | 0.0022 | ------ |
| TRINITY_DN333254_c0_g1_i2_m.2228931 | hemerythrin | 1.29 | 0.0078 | ------ |
| TRINITY_DN382929_c1_g1_i2_m.2185107 | vacuolar protein sorting associated protein 9A isoform X1 | 1.21 | 0.0077 | ------ |
| TRINITY_DN315238_c0_g1_i2_m.2228121 | poly granule associated protein | 1.50 | 0.0247 | ------ |

| | | | | |
|---|---|---|---|---|
| TRINITY_DN386326_c0_g1_i2_m.1129005 | ER membrane protein complex subunit 8/9 homolog | 1.23 | 0.0047 | ------ |
| TRINITY_DN381705_c2_g2_i2_m.3288870 | jasmonate induced protein homolog | 1.82 | 0.0008 | ------ |
| TRINITY_DN376172_c1_g3_i4_m.1047084 | selT like protein isoform X1 | 1.34 | 0.0029 | ------ |
| TRINITY_DN324023_c0_g1_i3_m.1426329 | ribosome inactivating protein like | 1.39 | 0.0323 | ------ |
| TRINITY_DN386362_c3_g2_i9_m.1127977 | guanine nucleotide binding protein subunit beta like protein | 3.27 | 0.0142 | ------ |
| TRINITY_DN192059_c0_g1_i1_m.650903 | peptidase M4 | 1.80 | 0.0050 | ------ |
| TRINITY_DN539335_c0_g1_i1_m.3631311 | C4 dicarboxylate ABC transporter substrate binding protein | 1.44 | 0.0047 | ------ |
| TRINITY_DN874908_c0_g1_i1_m.3524274 | ABC transporter permease | 1.95 | 0.0232 | ------ |
| TRINITY_DN771205_c0_g1_i1_m.4205945 | hemolysin D | 2.44 | 0.0022 | ------ |
| TRINITY_DN388453_c0_g2_i3_m.2467916 | probable aldo keto reductase 1 | 1.26 | 0.0032 | ------ |
| TRINITY_DN919049_c0_g1_i1_m.335129 | general stress protein | 5.90 | 0.0104 | ------ |
| TRINITY_DN1019447_c0_g1_i1_m.4643872 | Major outer membrane lipoprotein | 4.44 | 0.0041 | ------ |
| TRINITY_DN901498_c0_g1_i1_m.302556 | Phasin protein | 1.47 | 0.0039 | ------ |
| TRINITY_DN392666_c0_g1_i20_m.2118108 | chloroplast low molecular weight heat shock protein HSP26.7b | 1.95 | 0.0066 | ------ |
| TRINITY_DN340839_c0_g1_i1_m.1359471 | TPA: plasma membrane intrinsic protein2 | 1.57 | 0.0030 | ------ |
| TRINITY_DN409766_c0_g1_i1_m.769468 | lipase | 1.70 | 0.0157 | ------ |
| TRINITY_DN399627_c1_g1_i2_m.3204017 | mitochondrial fission protein ELM1 | 1.25 | 0.0041 | ------ |
| TRINITY_DN392027_c0_g2_i2_m.2009201 | Programmed cell death protein 4 | 1.22 | 0.0053 | ------ |
| TRINITY_DN365307_c0_g1_i4_m.1113832 | kelch domain containing protein 3 | 1.34 | 0.0052 | ------ |
| **Down-regulation** | | | | |
| TRINITY_DN389354_c2_g3_i3_m.2197190 | serine hydroxymethyltransferase 4 | 0.77 | 0.0341 | GO:0006563;GO:0006544;GO:0035999;GO:0032259;GO:0004372;GO:0030170;GO:0008168 |
| TRINITY_DN361922_c3_g1_i1_m.1652164 | putative protein phosphatase 2C 52 | 0.66 | 0.0106 | GO:0006470;GO:0046872;GO:0004722 |
| TRINITY_DN399453_c3_g3_i2_m.1286908 | probable alpha,alpha trehalose phosphate synthase [UDP forming] | 0.79 | 0.0024 | GO:0016311;GO:0005992;GO:0004805 |
| TRINITY_DN398581_c1_g1_i10_m.1119070 | CTP synthase | 0.72 | 0.0016 | GO:0044210;GO:0006541;GO:0003883;GO:0005524 |
| TRINITY_DN399270_c7_g2_i12_m.2322625 | starch excess 4 | 0.78 | 0.0354 | GO:0007623;GO:0016310;GO:0005983;GO:0042352;GO:0030244;GO:0006470;GO:0005739;GO:0009570;GO:0050201;GO:0019203;GO:0008138;GO:0030247;GO:0047341;GO:0005524 |
| TRINITY_DN392537_c5_g2_i3_m.1492788 | cysteine proteinase 1 | 0.78 | 0.0145 | GO:0051603;GO:0005764;GO:0005615;GO:0004197 |
| TRINITY_DN391429_c0_g1_i2_m.3345097 | ATP synthase subunit delta | 0.77 | 0.0024 | GO:0007030;GO:0015986;GO:0009060;GO:0006972;GO:0009853;GO:0006833;GO:0006511;GO:0009266;GO:0046686;GO:0051788;GO:0006096;GO:0005754;GO:0046933;GO:0008270;GO:0046961 |
| TRINITY_DN396914_c1_g1_i5_m.2674201 | clustered mitochondria protein isoform X2 | 0.75 | 0.0006 | GO:0048312;GO:0006413;GO:0005737;GO:0003743 |
| TRINITY_DN386323_c0_g1_i5_m.1128079 | putative acyl transferase 3 | 0.81 | 0.0158 | GO:0016747 |
| TRINITY_DN396321_c1_g2_i8_m.2802225 | Alcohol dehydrogenase | 0.82 | 0.0373 | GO:0055114;GO:0050897;GO:0008270;GO:0016491 |
| TRINITY_DN388675_c0_g1_i16_m.1584430 | kynurenine formamidase | 0.77 | 0.0301 | GO:0019441;GO:0004061 |
| TRINITY_DN646459_c0_g1_i1_m.69564 | heat shock protein 90 | 0.70 | 0.0057 | GO:0006950;GO:0006457;GO:0051082;GO:0005524 |
| TRINITY_DN380417_c1_g3_i2_m.1297098 | vacuolar protein sorting associated protein 11 homolog | 0.76 | 0.0100 | GO:0007032;GO:0006904;GO:0006886;GO:0006816;GO:0035542;GO:0007040;GO:0009651;GO:0007030;GO:0030897;GO:0009705;GO:0005768;GO:0030674;GO:0008270 |
| TRINITY_DN375343_c0_g5_i1_m.2515318 | ATP synthase F0 subunit 1 | 0.77 | 0.0030 | GO:0015986;GO:0015991;GO:0045261;GO:0005743;GO:0046933;GO:0046961;GO:0005524 |
| TRINITY_DN389485_c2_g1_i4_m.1813411 | NADPH cytochrome P450 reductase | 0.76 | 0.0038 | GO:0055114;GO:0016021;GO:0005789;GO:0030586;GO:0003958;GO:0010181 |
| TRINITY_DN394502_c1_g3_i6_m.1163791 | Tryptophan synthase beta chain 2 | 0.69 | 0.0214 | GO:0000162;GO:0016021;GO:0009536;GO:0052684;GO:0030170;GO:0004834 |
| TRINITY_DN354587_c4_g1_i2_m.2187225 | heat shock protein 81 1 | 0.61 | 0.0136 | GO:0006950;GO:0006457;GO:0051082;GO:0005524 |
| TRINITY_DN392457_c0_g2_i2_m.1259454 | NADH dehydrogenase [ubiquinone] iron sulfur protein 1 | 0.76 | 0.0139 | GO:0006979;GO:0042773;GO:0005747;GO:0009507;GO:0008137;GO:0051536;GO:0009055 |
| TRINITY_DN377771_c4_g1_i6_m.2643385 | huntingtin interacting protein K | 0.78 | 0.0150 | GO:0005829 |

| | | | | |
|---|---|---|---|---|
| TRINITY_DN376397_c1_g1_i4_m.3129487 | peroxidase 73 like | 0.48 | 0.0022 | GO:0098869;GO:0042744;GO:0006979;GO:0055114;GO:0005576;GO:0020037;GO:0004601;GO:0046872 |
| TRINITY_DN668787_c0_g1_i1_m.50366 | threonine tRNA ligase | 0.66 | 0.0049 | GO:0090502;GO:0006435;GO:0006388;GO:0016021;GO:0005739;GO:0000213;GO:0004829;GO:0003723;GO:0005524 |
| TRINITY_DN394057_c2_g1_i3_m.897315 | GDP mannose 3,5 epimerase 2 | 0.71 | 0.0169 | GO:0019853;GO:0050662;GO:0047918 |
| TRINITY_DN397816_c1_g3_i3_m.1949074 | villin 3 like | 0.70 | 0.0116 | GO:0051017;GO:0051015 |
| TRINITY_DN376401_c1_g1_i10_m.3131112 | Dynamin 2B | 0.68 | 0.0077 | GO:0003924;GO:0005525 |
| TRINITY_DN51272_c0_g1_i1_m.4500000 | 50S ribosomal protein L25 | 0.23 | 0.0332 | GO:0006412;GO:0005840;GO:0003735;GO:0008097 |
| TRINITY_DN397885_c2_g2_i2_m.1945593 | Glutamate decarboxylase 1 | 0.81 | 0.0037 | GO:0006536;GO:0030170;GO:0004351 |
| TRINITY_DN396258_c1_g1_i10_m.1709921 | zinc binding alcohol dehydrogenase domain containing protein 2 like | 0.80 | 0.0262 | GO:0055114;GO:0008270;GO:0016491 |
| TRINITY_DN380445_c0_g2_i2_m.1296537 | probable linoleate 9S lipoxygenase 4 | 0.79 | 0.0455 | GO:0031408;GO:0055114;GO:0046872;GO:0016702 |
| TRINITY_DN376059_c0_g1_i2_m.3351256 | dirigent protein 21 | 0.82 | 0.0102 | GO:0009699;GO:0042349 |
| TRINITY_DN385791_c1_g1_i15_m.1142901 | Late embryogenesis abundant protein Lea14 A | 0.63 | 0.0405 | GO:0009735;GO:0009269;GO:0005794;GO:0005829;GO:0005886;GO:0009506 |
| TRINITY_DN389164_c0_g1_i11_m.1232371 | leucine carboxyl methyltransferase 1 | 0.37 | 0.0122 | GO:0032259;GO:0005829;GO:0008757 |
| TRINITY_DN384470_c1_g2_i1_m.1578266 | probable ATP dependent RNA helicase DHX35 | 0.79 | 0.0029 | GO:0006396;GO:0005737;GO:0005681;GO:0044822;GO:0004004;GO:0005524 |
| TRINITY_DN995155_c0_g1_i2_m.392919 | sodium translocating pyrophosphatase | 0.39 | 0.0426 | GO:0015992;GO:0055085;GO:0005887;GO:0004427;GO:0000287;GO:0009678 |
| TRINITY_DN393329_c4_g1_i2_m.2010303 | WALI7 | 0.71 | 0.0041 | GO:0006529;GO:0006541;GO:0005829;GO:0004066;GO:0042803 |
| TRINITY_DN394633_c3_g1_i2_m.1462102 | Protein grpE | 0.81 | 0.0111 | GO:0050790;GO:0006457;GO:0016021;GO:0005759;GO:0051087;GO:0000774;GO:0042803 |
| TRINITY_DN252474_c0_g1_i2_m.3783073 | enolase like | 0.79 | 0.0061 | GO:0006096;GO:0000015;GO:0000287;GO:0004634 |
| TRINITY_DN395888_c0_g2_i2_m.1519798 | TGW 7A | 0.72 | 0.0047 | GO:0003824 |
| TRINITY_DN307770_c0_g1_i1_m.2139460 | PHD finger protein | 0.77 | 0.0045 | GO:0006355;GO:0005634;GO:0016021;GO:0042393;GO:0008270 |
| TRINITY_DN371306_c0_g1_i1_m.2330797 | nitronate monooxygenase | 0.78 | 0.0154 | GO:0055114;GO:0046482;GO:0005829;GO:0051213;GO:0018580 |
| TRINITY_DN394181_c1_g1_i9_m.1902166 | hydroxymethylglutaryl CoA synthase like | 0.69 | 0.0116 | GO:0019287;GO:0005829;GO:0009506;GO:0004421 |
| TRINITY_DN397718_c3_g4_i1_m.2923889 | cis zeatin O glucosyltransferase 1 | 0.80 | 0.0130 | GO:0009813;GO:0052696;GO:0043231;GO:0016758;GO:0008194 |
| TRINITY_DN372840_c1_g1_i5_m.1410955 | Branched chain amino acid aminotransferase 5 | 0.82 | 0.0112 | GO:0009081;GO:0052654;GO:0052655;GO:0052656 |
| TRINITY_DN284563_c0_g1_i1_m.4008628 | importin subunit beta 1 | 0.78 | 0.0042 | GO:0000060;GO:0006607;GO:0000059;GO:0006610;GO:0034399;GO:0031965;GO:0009507;GO:0008565;GO:0008139;GO:0008536 |
| TRINITY_DN395260_c3_g4_i2_m.1375019 | sucrose synthase 7 | 0.82 | 0.0145 | GO:0005985;GO:0005737;GO:0016020;GO:0016157 |
| TRINITY_DN393003_c1_g1_i12_m.2522878 | probable methylenetetrahydrofolate reductase | 0.68 | 0.0022 | GO:0006555;GO:0035999;GO:0055114;GO:0005829;GO:0004489 |
| TRINITY_DN378668_c4_g1_i1_m.3270454 | Elongation factor 1 delta | 0.81 | 0.0418 | GO:0006414;GO:0005853;GO:0003746 |
| TRINITY_DN321755_c0_g1_i1_m.3147984 | proline rich receptor like protein kinase PERK2 | 0.68 | 0.0003 | GO:0016020 |
| TRINITY_DN399461_c3_g5_i3_m.1289336 | Tetratricopeptide repeat protein 1 | 0.75 | 0.0035 | GO:0009536;GO:0051879 |
| TRINITY_DN370073_c0_g1_i2_m.2930966 | 30S ribosomal protein S7 | 0.38 | 0.0280 | GO:0006412;GO:0015935;GO:0003735;GO:0000049;GO:0019843 |
| TRINITY_DN396152_c2_g2_i1_m.3083191 | heat shock 70 kDa protein | 0.79 | 0.0172 | GO:0006457;GO:0005739;GO:0051082;GO:0005524 |
| TRINITY_DN87570_c0_g1_i2_m.4533022 | dehydrogenase | 0.24 | 0.0138 | GO:0055114;GO:0016020;GO:0030288;GO:0052935;GO:0052936;GO:0018468;GO:0005509 |
| TRINITY_DN391302_c2_g2_i2_m.2775006 | SNF1 related protein kinase regulatory subunit gamma 1 | 0.76 | 0.0102 | GO:0016310;GO:0016301 |
| TRINITY_DN392682_c1_g1_i11_m.2119489 | Calreticulin 3 | 0.64 | 0.0385 | GO:0009697;GO:0010204;GO:0006995;GO:0006457;GO:0046283;GO:0055074;GO:0009627;GO:0009626;GO:0042742;GO:0034976;GO:0016021;GO:0005789;GO:0051082;GO:0005509 |
| TRINITY_DN378352_c1_g1_i1_m.862410 | aminopeptidase M1 B | 0.78 | 0.0245 | GO:0043171;GO:0006508;GO:0009536;GO:0031090;GO:0005886;GO:0005783;GO:0042277;GO:0070006;GO:0008270 |
| TRINITY_DN389264_c0_g1_i2_m.3175954 | Transcription elongation factor A protein 2 | 0.73 | 0.0009 | GO:0032784;GO:0006357;GO:0006414;GO:0005634;GO:0008270;GO:0003677;GO:0003746 |
| TRINITY_DN398155_c2_g3_i5_m.3074189 | Calcium transporting ATPase 1 | 0.69 | 0.0004 | GO:0070588;GO:0043231;GO:0005887;GO:0005516;GO:0005388;GO:0046872;GO:0005524 |
| TRINITY_DN310821_c0_g1_i1_m.2738112 | purple acid phosphatase 2 like | 0.61 | 0.0019 | GO:0016311;GO:0016021;GO:0046872;GO:0003993 |

| | | | | |
|---|---|---|---|---|
| TRINITY_DN370757_c0_g2_i1_m.2475175 | macro domain containing protein VPA0103 | 0.78 | 0.0112 | GO:0015979;GO:0005829;GO:0009654;GO:0019898;GO:0005509 |
| TRINITY_DN363133_c3_g3_i1_m.2050947 | actin | 0.82 | 0.0247 | GO:0005524 |
| TRINITY_DN397670_c0_g1_i2_m.3160374 | AP 2 complex subunit alpha 2 like | 0.80 | 0.0056 | GO:0006886;GO:0016192;GO:0030131;GO:0008565 |
| TRINITY_DN399846_c2_g1_i3_m.2046450 | Casein kinase I isoform delta like protein | 0.80 | 0.0232 | GO:0018105;GO:0006897;GO:0016055;GO:0008360;GO:0005737;GO:0005634;GO:0004674;GO:0005524 |
| TRINITY_DN398110_c0_g1_i3_m.3073999 | cytochrome c oxidase subunit 6b 1 | 0.75 | 0.0491 | GO:1902600;GO:0006123;GO:0009853;GO:0009060;GO:0009535;GO:0005751;GO:0004129;GO:0005507 |
| TRINITY_DN372819_c0_g1_i2_m.1411420 | histone deacetylase | 0.69 | 0.0046 | GO:0006355;GO:0070932;GO:0005634;GO:0032041 |
| TRINITY_DN392082_c0_g1_i1_m.2009482 | ATP dependent zinc metalloprotease FTSH 1 | 0.75 | 0.0145 | GO:0010304;GO:0010205;GO:0010206;GO:0051301;GO:0006508;GO:0010027;GO:0007049;GO:0009941;GO:0009535;GO:0016021;GO:0005739;GO:0004176;GO:0004222;GO:0005524;GO:0046872 |
| TRINITY_DN399288_c2_g1_i7_m.2319956 | probable LRR receptor like serine/threonine protein kinase At1g06840 isoform X1 | 0.82 | 0.0305 | GO:0006468;GO:0016021;GO:0004674;GO:0005524 |
| TRINITY_DN388405_c4_g10_i1_m.1980415 | beta tubulin 2 | 0.63 | 0.0243 | GO:0007017;GO:0005737;GO:0005874;GO:0003924;GO:0005200;GO:0005525 |
| TRINITY_DN396009_c6_g1_i1_m.1773691 | Trans cinnamate 4 monooxygenase | 0.46 | 0.0009 | GO:0009808;GO:0044550;GO:0055114;GO:0016020;GO:0020037;GO:0016710;GO:0005506 |
| TRINITY_DN388621_c0_g2_i3_m.1588955 | Glucan endo 1,3 beta glucosidase 12 | 0.63 | 0.0020 | GO:0005975;GO:0006177;GO:0046658;GO:0016462;GO:0003922;GO:0030247;GO:0004553;GO:0005524 |
| TRINITY_DN396009_c6_g1_i1_m.1773691 | Trans cinnamate 4 monooxygenase | 0.46 | 0.0009 | GO:0009808;GO:0044550;GO:0055114;GO:0016020;GO:0020037;GO:0016710;GO:0005506 |
| TRINITY_DN15193_c0_g1_i1_m.4497342 | membrane protein | 0.20 | 0.0470 | GO:0016021;GO:0009279;GO:0005198 |
| TRINITY_DN266316_c0_g1_i1_m.3917957 | enolase 2 | 0.56 | 0.0082 | GO:0006096;GO:0006108;GO:0055114;GO:0000015;GO:0004634;GO:0016615;GO:0016616;GO:0000287 |
| TRINITY_DN377195_c0_g1_i4_m.3242412 | probable ubiquitin receptor RAD23 | 0.60 | 0.0447 | GO:0043161;GO:0006289;GO:0005634;GO:0003684 |
| TRINITY_DN397531_c0_g1_i9_m.2295482 | Eukaryotic translation initiation factor 3 subunit D | 0.47 | 0.0336 | GO:0001731;GO:0006446;GO:0005852;GO:0016282;GO:0016021;GO:0033290;GO:0003743 |
| TRINITY_DN391831_c0_g1_i3_m.2604992 | Af10 protein | 0.40 | 0.0151 | GO:0006623;GO:0009579;GO:0005794;GO:0005884;GO:0030276;GO:0002020 |
| TRINITY_DN382694_c1_g1_i1_m.917509 | P type H+ ATPase | 0.33 | 0.0169 | GO:1902600;GO:0006754;GO:0016021;GO:0008553;GO:0046872;GO:0005524 |
| TRINITY_DN396810_c0_g1_i1_m.2568454 | protein HIRA isoform X1 | 0.83 | 0.0445 | GO:0006355;GO:0005634;GO:0005515 |
| TRINITY_DN370839_c0_g1_i2_m.1168029 | histidine protein methyltransferase 1 homolog isoform X2 | 0.78 | 0.0386 | GO:0009536 |
| TRINITY_DN398387_c3_g3_i6_m.2795115 | Phosphoribosylamine glycine ligase | 0.75 | 0.0026 | GO:0009113;GO:0009536;GO:0046872;GO:0005524;GO:0004637 |
| TRINITY_DN371759_c0_g1_i3_m.2596592 | probable succinyl CoA ligase [ADP forming] subunit alpha | 0.77 | 0.0258 | GO:0006105;GO:0006104;GO:0006099;GO:0009142;GO:0009536;GO:0005829;GO:0005759;GO:0048037;GO:0004775;GO:0004776;GO:0005524 |
| TRINITY_DN372558_c1_g1_i2_m.2763835 | S adenosylmethionine synthase 3/Methionine adenosyltransferase 3 | 0.55 | 0.0006 | GO:0006556;GO:0006730;GO:0005829;GO:0004478;GO:0046872;GO:0005524 |
| TRINITY_DN328628_c0_g1_i1_m.3111598 | COP9 signalosome complex subunit 2 | 0.66 | 0.0079 | GO:0010388;GO:0030163;GO:0009640;GO:0005829 |
| TRINITY_DN369394_c2_g1_i1_m.2150045 | Glutamate 1 semialdehyde 2,1 aminomutase/Glutamate 1 semialdehyde aminotransferase | 0.58 | 0.0238 | GO:0015995;GO:0006782;GO:0009507;GO:0042286;GO:0008483;GO:0030170;GO:0042802 |
| TRINITY_DN391068_c1_g1_i12_m.3324213 | S norcoclaurine synthase 1 | 0.71 | 0.0156 | GO:0055114;GO:0046872;GO:0051213 |
| TRINITY_DN397838_c0_g1_i8_m.1949756 | Pollen specific protein SF3 | 0.63 | 0.0037 | GO:0008270 |
| TRINITY_DN363011_c0_g1_i4_m.2456666 | Serine carboxypeptidase like 51 | 0.66 | 0.0189 | GO:0051603;GO:0004185 |
| TRINITY_DN397952_c5_g1_i5_m.2060299 | 50S ribosomal protein L7/L12 | 0.32 | 0.0158 | GO:0006412;GO:0005840;GO:0003735 |
| TRINITY_DN399347_c2_g1_i1_m.1326443 | transcription initiation protein SPT3 homolog | 0.64 | 0.0251 | GO:0006366;GO:0009536;GO:0003723 |
| TRINITY_DN377402_c2_g2_i2_m.2689083 | 50S ribosomal protein L17 | 0.70 | 0.0218 | GO:0006412;GO:0015934;GO:0005739;GO:0003735 |
| TRINITY_DN364276_c0_g1_i1_m.2511846 | 70 kDa peptidyl prolyl isomerase | 0.74 | 0.0058 | GO:0000413;GO:0061077;GO:0016021;GO:0005789;GO:0005528;GO:0003755 |
| TRINITY_DN395800_c0_g2_i11_m.1783357 | pantothenate kinase 1 isoform X2 | 0.74 | 0.0009 | GO:0016310;GO:0015937;GO:0004594;GO:0005524 |
| TRINITY_DN325101_c0_g1_i2_m.1105182 | tyrosine biosynthesis bifunctional enzyme | 0.82 | 0.0042 | GO:0006571;GO:0009094;GO:0055114;GO:0008977;GO:0008483;GO:0004664;GO:0004665;G |

| | | | | |
|---|---|---|---|---|
| | | | | O:0030170 |
| TRINITY_DN395528_c0_g2_i7_m.3045280 | Hsp90 like protein | 0.69 | 0.0463 | GO:0006457;GO:0010075;GO:0009306;GO:0009414;GO:0009651;GO:0009934;GO:0005788;GO:0005739;GO:0005774;GO:0048046;GO:0005886;GO:0009507;GO:0005634;GO:0009506;GO:0051082;GO:0005524 |
| TRINITY_DN393259_c2_g3_i4_m.1874676 | chromatin assembly factor 1 subunit A isoform X2 | 0.80 | 0.0099 | GO:0046872 |
| TRINITY_DN398801_c0_g1_i5_m.1996431 | bifunctional protein FolD 2 | 0.81 | 0.0036 | GO:0009396;GO:0006730;GO:0055114;GO:0005829;GO:0004477;GO:0004488 |
| TRINITY_DN321220_c0_g1_i1_m.2866852 | Peroxidase 12 | 0.68 | 0.0020 | GO:0098869;GO:0042744;GO:0006979;GO:0055114;GO:0005576;GO:0020037;GO:0004601;GO:0046872 |
| TRINITY_DN382343_c0_g2_i8_m.2700119 | Proteasome subunit beta type 4 | 0.79 | 0.0028 | GO:0006511;GO:0005634;GO:0022626;GO:0005839;GO:0004298 |
| TRINITY_DN354281_c1_g2_i2_m.1066233 | Adenosylhomocysteinase | 0.60 | 0.0229 | GO:0006730;GO:0004013 |
| TRINITY_DN382354_c1_g1_i5_m.2699991 | fructokinase 2 | 0.69 | 0.0275 | GO:0046835;GO:0019252;GO:0008865;GO:0005524 |
| TRINITY_DN397547_c2_g1_i1_m.2296516 | heat shock protein 90 alpha | 0.74 | 0.0206 | GO:0006950;GO:0006457;GO:0051082;GO:0005524 |
| TRINITY_DN394025_c0_g1_i3_m.896994 | pyruvate dehydrogenase E1 component subunit alpha 1 | 0.78 | 0.0262 | GO:0006086;GO:0006096;GO:0055114;GO:0005759;GO:0004739 |
| TRINITY_DN361761_c7_g5_i3_m.2403903 | Heat shock cognate 70 kDa protein | 0.82 | 0.0213 | GO:0005524 |
| TRINITY_DN398980_c0_g1_i7_m.1277474 | NEDD8 activating enzyme E1 catalytic subunit | 0.71 | 0.0013 | GO:0045116;GO:0005634;GO:0005829;GO:0019781;GO:0016881;GO:0005524 |
| TRINITY_DN253914_c0_g1_i1_m.4121019 | protein GrpE like | 0.80 | 0.0017 | GO:0050790;GO:0006457;GO:0005759;GO:0051087;GO:0000774;GO:0042803 |
| TRINITY_DN388147_c2_g1_i14_m.1809008 | monodehydroascorbate reductase | 0.73 | 0.0195 | GO:0022900;GO:0045454;GO:0005623;GO:0050660;GO:0016656;GO:0015036 |
| TRINITY_DN398946_c6_g5_i1_m.1279612 | SOS1 | 0.83 | 0.0166 | GO:1902600;GO:0016021;GO:0015299 |
| TRINITY_DN395531_c0_g1_i11_m.3040268 | probable zinc protease PqqL | 0.79 | 0.0049 | GO:0051603;GO:0043171;GO:0016485;GO:0005739;GO:0009507;GO:0004222;GO:0008270 |
| TRINITY_DN398430_c1_g1_i4_m.2873885 | Coatomer subunit alpha 3 | 0.71 | 0.0001 | GO:0006891;GO:0006890;GO:0006888;GO:0006886;GO:0005739;GO:0000139;GO:0030126;GO:0005198 |
| TRINITY_DN54903_c0_g1_i1_m.4463657 | peptidyl prolyl cis trans isomerase | 0.72 | 0.0006 | GO:0000413;GO:0006457;GO:0003755 |
| TRINITY_DN383275_c2_g1_i1_m.3049520 | 50S ribosomal protein L16 | 0.44 | 0.0237 | GO:0006412;GO:0005840;GO:0003735;GO:0000049;GO:0019843 |
| TRINITY_DN386103_c1_g1_i3_m.2973155 | probable mannose 1 phosphate guanylyltransferase 1 | 0.65 | 0.0002 | GO:0009298;GO:0004475;GO:0005525 |
| TRINITY_DN399051_c1_g1_i1_m.2650290 | Cysteine rich receptor like protein kinase 6 | 0.69 | 0.0426 | GO:0006468;GO:0006952;GO:0009506;GO:0005886;GO:0030246;GO:0004674;GO:0005524 |
| TRINITY_DN388245_c1_g3_i13_m.1185477 | anthocyanidin reductase like | 0.79 | 0.0415 | GO:0050662;GO:0003824 |
| TRINITY_DN394346_c0_g5_i3_m.2270638 | 5' nucleotidase domain containing protein DDB_G0275467 | 0.77 | 0.0263 | GO:0016311;GO:0005739;GO:0046872;GO:0008253 |
| TRINITY_DN389699_c0_g1_i2_m.2350966 | T complex protein 1 subunit gamma | 0.73 | 0.0391 | GO:0006457;GO:0005829;GO:0051082;GO:0005524 |
| TRINITY_DN391809_c1_g1_i1_m.2604206 | claspin isoform X1 | 0.71 | 0.0243 | GO:0003677 |
| TRINITY_DN392233_c2_g1_i5_m.1908574 | Transketolase | 0.72 | 0.0104 | GO:0019253;GO:0009535;GO:0004802;GO:0046872 |
| TRINITY_DN376353_c0_g1_i2_m.3133451 | activator of 90 kDa heat shock protein ATPase homolog | 0.70 | 0.0484 | GO:0006457;GO:0042542;GO:0032781;GO:0009408;GO:0009644;GO:0034976;GO:0005829;GO:0051087;GO:0001671 |
| TRINITY_DN395341_c8_g1_i1_m.2575505 | actin 7 like | 0.69 | 0.0188 | GO:0005856;GO:0005737;GO:0005524 |
| TRINITY_DN397980_c0_g1_i1_m.2056303 | probable 26S proteasome non ATPase regulatory subunit 3 | 0.82 | 0.0114 | GO:0042176;GO:0006511;GO:0050790;GO:0008541;GO:0030234 |
| TRINITY_DN174218_c0_g1_i1_m.545002 | probable fructokinase 6 | 0.47 | 0.0201 | GO:0046835;GO:0006014;GO:0009570;GO:0008865;GO:0004747 |
| TRINITY_DN388274_c0_g1_i8_m.1185682 | cycloartenol C 24 methyltransferase 1 | 0.80 | 0.0314 | GO:0016126;GO:0032259;GO:0016021;GO:0005802;GO:0005774;GO:0005768;GO:0003838 |
| TRINITY_DN395150_c1_g1_i3_m.2843095 | exocyst complex component SEC6 | 0.73 | 0.0016 | GO:0006355;GO:0006887;GO:0051601;GO:0000145;GO:0000149 |
| TRINITY_DN383484_c1_g2_i2_m.2611496 | malate synthase | 0.74 | 0.0059 | GO:0006099;GO:0006097;GO:0004474 |
| TRINITY_DN383227_c4_g2_i2_m.3051200 | S adenosylmethionine synthase 2 | 0.67 | 0.0016 | GO:0006556;GO:0006730;GO:0009651;GO:0005829;GO:0004478;GO:0046872;GO:0005524 |
| TRINITY_DN379487_c1_g2_i1_m.2940001 | calcium dependent protein kinase SK5 | 0.73 | 0.0224 | GO:0018105;GO:0009738;GO:0035556;GO:0046777;GO:0005737;GO:0005634;GO:0005886;GO:0004683;GO:0009931;GO:0005509;GO:0005524;GO:0005516 |
| TRINITY_DN273784_c0_g1_i2_m.3923600 | acyl carrier protein [Candidatus Accumulibacter phosphatis | 0.23 | 0.0267 | GO:0006633;GO:0005737;GO:0000036 |
| TRINITY_DN394088_c2_g1_i2_m.895649 | endotransglucosylase/hydrolase XTH1 | 0.79 | 0.0436 | GO:0042546;GO:0071555;GO:0010411;GO:0005618;GO:0048046;GO:0016762;GO:0004553 |
| TRINITY_DN368125_c0_g1_i10_m.888385 | universal stress protein 930 | 0.73 | 0.0045 | GO:0006950 |

| | | | | |
|---|---|---|---|---|
| TRINITY_DN372408_c0_g2_i2_m.1590273 | momilactone A synthase like | 0.62 | 0.0304 | GO:0051504;GO:0055114;GO:0016491 |
| TRINITY_DN736151_c0_g1_i1_m.4198061 | DNA binding protein | 0.26 | 0.0044 | GO:0030261;GO:0003677 |
| TRINITY_DN392546_c2_g1_i3_m.1489197 | 26S proteasome non ATPase regulatory subunit 1 homolog A like | 0.55 | 0.0232 | GO:0043161;GO:0050790;GO:0042176;GO:0016021;GO:0005634;GO:0034515;GO:0008540;GO:0004175;GO:0030234 |
| TRINITY_DN395119_c0_g5_i6_m.2842134 | signal recognition particle subunit SRP68 | 0.74 | 0.0357 | GO:0006614;GO:0005786;GO:0005783;GO:0005829;GO:0030942;GO:0005047;GO:0008312 |
| TRINITY_DN351452_c2_g1_i11_m.2840857 | glucose 6 phosphate 1 dehydrogenase | 0.55 | 0.0039 | GO:0006098;GO:0006006;GO:0055114;GO:0016021;GO:0050661;GO:0004345 |
| TRINITY_DN328030_c2_g1_i1_m.1319606 | tubulin alpha 1 chain | 0.32 | 0.0186 | GO:0007017;GO:0005737;GO:0005874;GO:0003924;GO:0005200;GO:0005525 |
| TRINITY_DN397718_c3_g3_i1_m.2923869 | UDP glycosyltransferase UGT93B9 | 0.70 | 0.0379 | GO:0009813;GO:0052696;GO:0043231;GO:0016758;GO:0008194 |
| TRINITY_DN222880_c0_g1_i2_m.3946675 | Ca2+ binding protein cbp1 | 0.83 | 0.0158 | GO:0016021;GO:0005739;GO:0043022;GO:0005509 |
| TRINITY_DN392611_c0_g2_i5_m.2121542 | serine carboxypeptidase like | 0.71 | 0.0214 | GO:0051603;GO:0005773;GO:0016021;GO:0005777;GO:0005789;GO:0005829;GO:0004185 |
| TRINITY_DN364589_c1_g1_i20_m.2707447 | NADH cytochrome b5 reductase 1 | 0.79 | 0.0186 | GO:0022900;GO:0005783;GO:0016021;GO:0005886;GO:0009505;GO:0004128 |
| TRINITY_DN385273_c1_g1_i4_m.2391219 | 3 hydroxyisobutyryl CoA hydrolase | 0.79 | 0.0064 | GO:0009220;GO:0034968;GO:0010388;GO:0009909;GO:0009640;GO:0005739;GO:0003860 |
| TRINITY_DN389000_c0_g4_i5_m.1611123 | V type proton ATPase subunit B 2 | 0.83 | 0.0254 | GO:0046034;GO:0015991;GO:0033180;GO:0016820;GO:0005524 |
| TRINITY_DN398219_c0_g1_i8_m.1378720 | pirin like protein isoform X1 | 0.76 | 0.0003 | GO:0016020;GO:0005739 |
| TRINITY_DN396227_c3_g4_i1_m.1712258 | Diphosphomevalonate decarboxylase | 0.80 | 0.0004 | GO:0019287;GO:0005829;GO:0004163;GO:0005524 |
| TRINITY_DN385027_c2_g1_i17_m.2188570 | alpha galactosidase | 0.52 | 0.0097 | GO:0005975;GO:0009620;GO:0009911;GO:0009965;GO:0048046;GO:0009505;GO:0052692 |
| TRINITY_DN384380_c1_g2_i1_m.2376836 | ATP dependent Clp protease proteolytic subunit | 0.81 | 0.0182 | GO:0006508;GO:0005739;GO:0004252 |
| TRINITY_DN391523_c5_g1_i6_m.2833802 | Dihydroflavonol 4 reductase | 0.82 | 0.0256 | GO:0006694;GO:0055114;GO:0003854;GO:0050662 |
| TRINITY_DN398666_c2_g2_i2_m.2659535 | Replication factor C subunit 1 | 0.55 | 0.0216 | GO:0006468;GO:0031935;GO:0009737;GO:0006281;GO:0000712;GO:0006260;GO:0051570;GO:0005634;GO:0005663;GO:0003689;GO:0004672;GO:0005524 |
| TRINITY_DN396827_c0_g3_i1_m.2565619 | mevalonate kinase | 0.78 | 0.0034 | GO:0016310;GO:0008299;GO:0005829;GO:0004496;GO:0005524 |
| TRINITY_DN375108_c0_g1_i10_m.3264516 | mannose 1 phosphate guanyltransferase alpha | 0.74 | 0.0011 | GO:0009058;GO:0016779 |
| TRINITY_DN389938_c1_g4_i1_m.1920758 | arabinoxylan arabinofuranohydrolase | 0.75 | 0.0130 | GO:0006810;GO:0046373;GO:0016021;GO:0005215;GO:0046556 |
| TRINITY_DN391714_c3_g2_i1_m.2155887 | anthocyanidin 5,3 O glucosyltransferase | 0.78 | 0.0283 | GO:0009813;GO:0052696;GO:0009536;GO:0080043;GO:0080044 |
| TRINITY_DN391986_c1_g2_i1_m.1854740 | ankyrin repeat domain containing protein 2A like | 0.78 | 0.0426 | GO:0009536 |
| TRINITY_DN398166_c2_g1_i2_m.3071079 | Disease resistance protein RPM1 | 0.79 | 0.0108 | GO:0043531 |
| TRINITY_DN383275_c2_g1_i1_m.3049523 | 50S ribosomal protein L18 | 0.40 | 0.0232 | GO:0006412;GO:0005840;GO:0003735;GO:0019843 |
| TRINITY_DN395330_c1_g4_i1_m.2575409 | structural maintenance of chromosomes protein 1 | 0.81 | 0.0305 | GO:0051276;GO:0005634;GO:0005694;GO:0005524 |
| TRINITY_DN167110_c0_g1_i1_m.626077 | pre mRNA splicing factor RBM22 | 0.71 | 0.0327 | GO:0046872;GO:0003723;GO:0000166 |
| TRINITY_DN358323_c1_g1_i1_m.2722587 | mRNA decapping enzyme subunit 2 | 0.72 | 0.0268 | GO:0006468;GO:0000290;GO:0005739;GO:0030145;GO:0050072;GO:0003723;GO:0004672;GO:0005524 |
| TRINITY_DN386069_c0_g3_i6_m.2973127 | Epoxide hydrolase 2 | 0.78 | 0.0161 | GO:0016787 |
| TRINITY_DN382294_c1_g2_i5_m.1497186 | glyceraldehyde 3 phosphate dehydrogenase 2 | 0.70 | 0.0142 | GO:0006006;GO:0006096;GO:0055114;GO:0005737;GO:0004365;GO:0051287;GO:0050661 |
| TRINITY_DN381886_c0_g4_i1_m.1480960 | ubiquitin carboxyl terminal hydrolase isozyme L3 | 0.72 | 0.0191 | GO:0006511;GO:0016579;GO:0005737;GO:0004843 |
| TRINITY_DN381716_c1_g1_i5_m.1786564 | UDP glycosyltransferase 88A1 like | 0.76 | 0.0416 | GO:0009813;GO:0052696;GO:0043231;GO:0080043;GO:0080044 |
| TRINITY_DN399962_c10_g2_i1_m.1629047 | 50S ribosomal protein L3 | 0.36 | 0.0427 | GO:0006412;GO:0005840;GO:0003735;GO:0019843 |
| TRINITY_DN377554_c0_g1_i5_m.1935286 | Putative hydroquinone glucosyltransferase | 0.59 | 0.0047 | GO:0009813;GO:0052696;GO:0043231;GO:0080043;GO:0080044 |
| TRINITY_DN383957_c1_g1_i3_m.2787537 | BSD domain containing protein 1 | 0.69 | 0.0233 | GO:0009536 |
| TRINITY_DN165132_c0_g1_i1_m.407857 | splicing factor 3B subunit 1 | 0.67 | 0.0065 | GO:0000245;GO:0071013;GO:0071004;GO:0005689;GO:0005686;GO:0003729 |
| TRINITY_DN362495_c0_g2_i3_m.1862888 | Chaperonin CPN60 2 | 0.65 | 0.0438 | GO:0042026;GO:0009536;GO:0005739;GO:0005524 |
| TRINITY_DN291830_c0_g1_i2_m.3781587 | eukaryotic peptide chain release factor GTP binding subunit ERF3A like | 0.61 | 0.0342 | GO:0032790;GO:0002184;GO:0005829;GO:0018444;GO:0003924;GO:0003747;GO:0005525 |
| TRINITY_DN396565_c13_g1_i8_m.2308008 | cysteine endopeptidase EP gamma | 0.60 | 0.0052 | GO:0051603;GO:0006955;GO:0005764;GO:0005615;GO:0004197 |
| TRINITY_DN332903_c0_g1_i1_m.1735575 | probable proteasome inhibitor | 0.52 | 0.0044 | GO:0006511 |

| | | | | |
|---|---|---|---|---|
| TRINITY_DN360553_c2_g1_i8_m.1003782 | 26S protease regulatory subunit 6A like protein | 0.57 | 0.0161 | GO:0030433;GO:1901800;GO:0045899;GO:0031595;GO:0031597;GO:0008540;GO:0036402;GO:0017025;GO:0008233;GO:0005524 |
| TRINITY_DN398351_c7_g2_i1_m.2799133 | fructan beta | 0.58 | 0.0196 | GO:0005975;GO:0048046;GO:0005618;GO:0004575 |
| TRINITY_DN397445_c4_g2_i3_m.1053732 | cytosolic heat shock protein 90.1 | 0.44 | 0.0110 | GO:0006950;GO:0006457;GO:0005737;GO:0051082;GO:0005524 |
| TRINITY_DN374726_c1_g1_i3_m.3184904 | TSK1 protein | 0.80 | 0.0184 | GO:0016310;GO:0006511;GO:0016301 |
| TRINITY_DN397223_c1_g2_i4_m.1026247 | farnesyl pyrophosphate synthase B2 | 0.82 | 0.0179 | GO:0045337;GO:0006695;GO:0033384;GO:0005737;GO:0004161;GO:0004337;GO:0046872 |
| TRINITY_DN396817_c1_g1_i12_m.2568584 | Putative glycerophosphoryl diester phosphodiesterase 1 | 0.80 | 0.0309 | GO:0006629;GO:0016021;GO:0008889 |
| TRINITY_DN398786_c4_g1_i8_m.1999890 | staphylococcal nuclease domain containing protein 1 | 0.72 | 0.0091 | GO:0035194;GO:1903506;GO:0016442;GO:0003712 |
| TRINITY_DN365246_c0_g1_i1_m.2409209 | Spermidine synthase 1 | 0.71 | 0.0387 | GO:0008295;GO:0004766 |
| TRINITY_DN399252_c3_g1_i6_m.2319742 | lipoxygenase | 0.80 | 0.0092 | GO:0031408;GO:0055114;GO:0046872;GO:0016702 |
| TRINITY_DN395179_c0_g1_i2_m.2843068 | polyribonucleotide nucleotidyltransferase 2 | 0.77 | 0.0027 | GO:0090503;GO:0008033;GO:0006364;GO:0000963;GO:0000957;GO:0006402;GO:0006397;GO:0042991;GO:0005739;GO:0004654;GO:0000175;GO:0003723 |
| TRINITY_DN374140_c0_g1_i3_m.1698205 | Rab18l | 0.78 | 0.0003 | GO:0007264;GO:0005622;GO:0005886;GO:0005525 |
| TRINITY_DN395560_c0_g3_i2_m.3046384 | conserved oligomeric Golgi complex subunit 1 | 0.80 | 0.0001 | GO:0007030;GO:0000301;GO:0005829;GO:0017119 |
| TRINITY_DN362137_c0_g1_i4_m.2079138 | actin related protein 2/3 complex subunit 5A like | 0.83 | 0.0132 | GO:0034314;GO:0005885 |
| TRINITY_DN359313_c1_g1_i1_m.2479082 | Ubiquitin carboxyl terminal hydrolase 12 | 0.83 | 0.0026 | GO:0006511;GO:0016579;GO:0005739;GO:0036459 |
| TRINITY_DN351452_c2_g1_i7_m.2840834 | cytosolic glucose 6 phosphate dehydrogenase | 0.73 | 0.0000 | GO:0006098;GO:0006006;GO:0055114;GO:0016021;GO:0050661;GO:0004345 |
| TRINITY_DN397784_c10_g3_i1_m.2928080 | elongation factor 2 like | 0.82 | 0.0486 | GO:0006414;GO:0005622;GO:0003746;GO:0003924;GO:0005525 |
| TRINITY_DN396396_c1_g1_i17_m.2802165 | ATP dependent DNA helicase 2 subunit KU70 | 0.78 | 0.0092 | GO:0006310;GO:0006303;GO:0071480;GO:0071481;GO:0032508;GO:0000723;GO:0043564;GO:0000784;GO:0042162;GO:0005515;GO:0004003;GO:0003690;GO:0003684;GO:0005524 |
| TRINITY_DN368099_c5_g3_i1_m.3298410 | probable mediator of RNA polymerase II transcription subunit 37c | 0.49 | 0.0023 | GO:0005524 |
| TRINITY_DN399851_c5_g1_i1_m.2044808 | Sister chromatid cohesion protein PDS5 like protein B | 0.82 | 0.0129 | GO:0007067;GO:0005829;GO:0005634;GO:0005739;GO:0009507 |
| TRINITY_DN370792_c2_g2_i2_m.2476492 | DNA binding protein DDB_G0278111 | 0.79 | 0.0063 | GO:0005829;GO:0003677 |
| TRINITY_DN391511_c1_g1_i4_m.2836667 | oryzain alpha chain | 0.74 | 0.0382 | GO:0006508;GO:0008234 |
| TRINITY_DN391777_c0_g2_i2_m.2161793 | 4 hydroxy tetrahydrodipicolinate synthase 1 | 0.76 | 0.0175 | GO:0009089;GO:0019877;GO:0009507;GO:0008840 |
| TRINITY_DN396359_c5_g1_i25_m.2804840 | Cysteine rich receptor like protein kinase 41 | 0.74 | 0.0373 | GO:0016310;GO:0000166;GO:0004672 |
| TRINITY_DN390397_c1_g1_i2_m.2620022 | glyceraldehyde 3 phosphate dehydrogenase 1 | 0.70 | 0.0071 | GO:0006006;GO:0006096;GO:0055114;GO:0005737;GO:0004365;GO:0051287;GO:0050661 |
| TRINITY_DN351611_c0_g1_i1_m.926425 | SKP1 like protein 11 | 0.64 | 0.0042 | GO:0016310;GO:0006511;GO:0016301 |
| TRINITY_DN351057_c0_g1_i7_m.1703238 | signal recognition particle 9 kDa protein | 0.64 | 0.0064 | GO:0045900;GO:0006616;GO:0005786;GO:0005785;GO:0005047;GO:0008312 |
| TRINITY_DN396025_c1_g4_i2_m.1777594 | charged multivesicular body protein 5 | 0.60 | 0.0139 | GO:0007034;GO:0005622 |
| TRINITY_DN385905_c2_g1_i1_m.3097369 | translationally controlled tumor protein | 0.65 | 0.0352 | GO:0005737 |
| TRINITY_DN363743_c1_g1_i1_m.3274537 | 60S acidic ribosomal protein P0 | 0.65 | 0.0325 | GO:0042254;GO:0002181;GO:0006414;GO:0022625;GO:0030687;GO:0070180;GO:0003735 |
| TRINITY_DN352186_c0_g1_i2_m.1454729 | Putative chromatin remodeling complex ATPase chain | 0.70 | 0.0412 | GO:0043044;GO:0016589;GO:0031491;GO:0016887;GO:0003677;GO:0005524 |
| TRINITY_DN374683_c0_g1_i2_m.879690 | Ran binding protein 1 like protein c | 0.59 | 0.0289 | GO:0043547;GO:0006606;GO:0007051;GO:0046604;GO:0006511;GO:0006405;GO:0000082;GO:0005737;GO:0005634;GO:0005813;GO:0005096;GO:0008536 |
| TRINITY_DN389271_c0_g1_i6_m.3175056 | Importin subunit alpha 1a | 0.56 | 0.0302 | GO:0006607;GO:0016032;GO:0005643;GO:0005829;GO:0005654;GO:0048471;GO:0008565;GO:0008139 |
| TRINITY_DN385135_c2_g1_i11_m.1192889 | UDP glucose:sterol glucosyltransferase | 0.45 | 0.0008 | GO:0016125;GO:0048316;GO:0052696;GO:0009813;GO:0030259;GO:0043231;GO:0005886;GO:0051507 |
| TRINITY_DN941973_c0_g1_i1_m.370801 | phosphoglycerate mutase like protein 1 | 0.65 | 0.0432 | GO:0009507 |
| TRINITY_DN396136_c2_g1_i19_m.3081065 | peroxisomal acyl coenzyme A oxidase 1 like | 0.73 | 0.0447 | GO:0033539;GO:0055088;GO:0005777;GO:0050660;GO:0052890;GO:0000062;GO:0003995;GO:0003997;GO:0009055 |
| TRINITY_DN336951_c0_g2_i1_m.1927085 | histone H2B isoform 1b | 0.44 | 0.0348 | GO:0006334;GO:0000788;GO:0046982;GO:0003677 |
| TRINITY_DN371784_c3_g1_i3_m.2598020 | Peroxidase 52 | 0.62 | 0.0187 | GO:0098869;GO:0042744;GO:0006979;GO:0055114;GO:0005576;GO:0020037;GO:0004601;G |

| | | | | |
|---|---|---|---|---|
| | | | | O:0046872 |
| TRINITY_DN392709_c0_g2_i2_m.902077 | AF268595_1 molybdenum cofactor biosynthesis protein Cnx1 | 0.77 | 0.0220 | GO:0006777;GO:0009734;GO:0018315;GO:0032324;GO:0005829;GO:0061599;GO:0061598;GO:0030151 |
| TRINITY_DN155322_c0_g1_i1_m.447655 | Pyrophosphate fructose 6 phosphate 1 phosphotransferase subunit beta | 0.77 | 0.0100 | GO:0046835;GO:0006002;GO:0061615;GO:0009536;GO:0047334;GO:0003872;GO:0005524 |
| TRINITY_DN398780_c14_g3_i1_m.2000298 | respiratory burst oxidase homolog protein B | 0.69 | 0.0039 | GO:0098869;GO:0055114;GO:0016021;GO:0050664;GO:0005509;GO:0004601 |
| TRINITY_DN398213_c4_g1_i1_m.1381247 | Lysosomal beta glucosidase | 0.79 | 0.0048 | GO:0009251;GO:0004338 |
| TRINITY_DN398911_c3_g4_i1_m.1277838 | serine/threonine protein kinase BLUS1 like isoform X2 | 0.83 | 0.0075 | GO:0023014;GO:0005737;GO:0004713;GO:0005524;GO:0004702 |
| TRINITY_DN360329_c0_g1_i3_m.1833966 | Peroxidase 47 | 0.65 | 0.0192 | GO:0009664;GO:0006979;GO:0098869;GO:0042744;GO:0055114;GO:0005576;GO:0009505;GO:0020037;GO:0046872;GO:0004601 |
| TRINITY_DN399962_c13_g4_i2_m.1629169 | 50S ribosomal protein L30 | 0.83 | 0.0477 | GO:0006412;GO:0022625;GO:0003735 |
| TRINITY_DN393318_c1_g1_i1_m.2013335 | gamma glutamylcysteine synthetase | 0.81 | 0.0215 | GO:0006750;GO:0005739;GO:0009507;GO:0004357;GO:0005524 |
| TRINITY_DN326602_c1_g1_i3_m.983444 | ABC transporter E family member 2 | 0.76 | 0.0285 | GO:0005524;GO:0016887 |
| TRINITY_DN394237_c1_g1_i8_m.2225294 | exocyst complex component 5 | 0.77 | 0.0002 | GO:0006887;GO:0048278;GO:0015031;GO:0000145;GO:0005886;GO:0009506 |
| TRINITY_DN398909_c1_g1_i3_m.1281128 | superkiller viralicidic activity 2 like 2 | 0.82 | 0.0406 | GO:0006406;GO:0030422;GO:0051301;GO:0010212;GO:0048825;GO:0016571;GO:0006401;GO:0016579;GO:0009909;GO:0031125;GO:0043687;GO:0006606;GO:0045893;GO:0000741;GO:0010388;GO:0009640;GO:0010074;GO:0009560;GO:0016567;GO:0016021;GO:0005634;GO:0005524;GO:0003724;GO:0003723 |
| TRINITY_DN395813_c3_g1_i5_m.1523262 | serine carboxypeptidase 1 | 0.80 | 0.0135 | GO:0051603;GO:0019748;GO:0005773;GO:0005777;GO:0004185;GO:0016747 |
| TRINITY_DN379976_c0_g1_i5_m.2422746 | pyruvate kinase isozyme A | 0.75 | 0.0130 | GO:0006096;GO:0009536;GO:0016021;GO:0030955;GO:0000287;GO:0016301;GO:0004743 |
| TRINITY_DN397222_c0_g1_i3_m.1022680 | splicing factor 3A subunit 3 | 0.80 | 0.0284 | GO:0000398;GO:0005681;GO:0003723;GO:0008270 |
| TRINITY_DN392343_c1_g1_i5_m.2440572 | Medium chain fatty acid CoA ligase | 0.67 | 0.0166 | GO:0008152;GO:0016874 |
| TRINITY_DN389164_c0_g1_i10_m.1232364 | leucine carboxyl methyl transferase | 0.74 | 0.0058 | GO:0032259;GO:0005829;GO:0008757 |
| TRINITY_DN374318_c2_g1_i3_m.1765384 | cullin 1 like | 0.78 | 0.0237 | GO:0042787;GO:0031461;GO:0061630;GO:0031625 |
| TRINITY_DN382312_c0_g1_i2_m.2696589 | Brefeldin A inhibited guanine nucleotide exchange protein 2 | 0.70 | 0.0005 | GO:0016192;GO:0032012;GO:0043547;GO:0009536;GO:0005802;GO:0005086 |
| TRINITY_DN381087_c0_g1_i7_m.2074433 | flap endonuclease 1 A | 0.71 | 0.0149 | GO:0006284;GO:0090305;GO:0043137;GO:0005730;GO:0005739;GO:0005654;GO:0008409;GO:0017108;GO:0003677;GO:0000287 |
| TRINITY_DN255416_c0_g1_i2_m.4029218 | 40S ribosomal protein SA | 0.64 | 0.0157 | GO:0000028;GO:0000461;GO:0000447;GO:0006412;GO:0006407;GO:0022627;GO:0030686;GO:0003735 |
| TRINITY_DN387060_c2_g1_i4_m.1417745 | RNA binding protein FUS | 0.62 | 0.0482 | GO:0009813;GO:0052696;GO:0043231;GO:0080043;GO:0080044 |
| TRINITY_DN399572_c1_g1_i4_m.3000605 | SUMO activating enzyme subunit 2 | 0.68 | 0.0289 | GO:0009793;GO:0016925;GO:0005829;GO:0031510;GO:0009506;GO:0019948 |
| TRINITY_DN392068_c0_g1_i4_m.2009024 | Putative inactive purple acid phosphatase 1 | 0.58 | 0.0419 | GO:0016311;GO:0046872;GO:0003993 |
| TRINITY_DN397000_c2_g1_i4_m.2669980 | Heat shock 70 kDa protein 4L | 0.52 | 0.0145 | GO:0005524 |
| TRINITY_DN49751_c0_g1_i1_m.4527386 | 60S ribosomal protein L12 | 0.43 | 0.0260 | GO:0006412;GO:0000027;GO:0022625;GO:0003735;GO:0019843 |
| TRINITY_DN373443_c2_g1_i5_m.1235529 | cinnamyl alcohol dehydrogenase | 0.63 | 0.0213 | GO:0009809;GO:0055114;GO:0009536;GO:0052747;GO:0045551;GO:0008270 |
| TRINITY_DN397461_c0_g2_i4_m.1052183 | E3 ubiquitin protein ligase UPL4 | 0.49 | 0.0251 | GO:0042787;GO:0005737;GO:0016021;GO:0005634;GO:0004842;GO:0016874 |
| TRINITY_DN367632_c3_g1_i10_m.1318877 | zinc finger CCCH domain containing protein 11 | 0.40 | 0.0181 | GO:0005739;GO:0046872;GO:0003677 |
| TRINITY_DN706289_c0_g1_i1_m.4239305 | ubiquitin conjugating enzyme E2 17 kDa | 0.74 | 0.0160 | GO:0005524 |
| TRINITY_DN339158_c0_g1_i3_m.2961104 | Peroxidase 1 | 0.71 | 0.0025 | GO:0009664;GO:0006979;GO:0098869;GO:0042744;GO:0055114;GO:0016021;GO:0005576;GO:0009505;GO:0020037;GO:0046872;GO:0004601 |
| TRINITY_DN383275_c2_g1_i1_m.3049533 | 30S ribosomal protein S17 | 0.47 | 0.0485 | GO:0006412;GO:0005840;GO:0003735;GO:0019843 |
| TRINITY_DN384112_c0_g1_i7_m.2853778 | succinate dehydrogenase [ubiquinone] flavoprotein subunit | 0.82 | 0.0028 | GO:0006099;GO:0022900;GO:0005743;GO:0050660;GO:0008177 |
| TRINITY_DN387886_c1_g3_i2_m.1641350 | pre mRNA splicing factor syf2 like | 0.83 | 0.0112 | GO:0000398;GO:0009536;GO:0071010;GO:0071013;GO:0071012;GO:0071014;GO:0000974 |
| TRINITY_DN394508_c3_g4_i1_m.2967339 | replication factor C subunit 4 | 0.74 | 0.0307 | GO:0006261;GO:0005634;GO:0005663;GO:0003689;GO:0005524 |

| | | | | |
|---|---|---|---|---|
| TRINITY_DN390922_c0_g1_i15_m.2251367 | Seven transmembrane domain containing tyrosine protein kinase 1 | 0.75 | 0.0308 | GO:0006468;GO:0005739;GO:0016021;GO:0005524;GO:0004672 |
| TRINITY_DN370286_c0_g1_i8_m.2481295 | uncharacterized protein At2g39795 | 0.75 | 0.0220 | GO:0005759;GO:0009536 |
| TRINITY_DN386725_c2_g1_i1_m.2810910 | MAP KINASE | 0.59 | 0.0001 | GO:0000169;GO:0006952;GO:0009555;GO:0080136;GO:0010120;GO:0010183;GO:0010229;GO:0048481;GO:0010224;GO:0005622;GO:0004707;GO:0005524 |
| TRINITY_DN372442_c0_g1_i6_m.1592322 | Glutathione S transferase | 0.68 | 0.0000 | GO:0006749;GO:0005737;GO:0004364 |
| TRINITY_DN397580_c3_g2_i4_m.2293982 | Cullin associated NEDD8 dissociated protein 1 | 0.74 | 0.0182 | GO:0048449;GO:0009793;GO:0050826;GO:0009733;GO:0045010;GO:0071555;GO:0010182;GO:0009845;GO:0009909;GO:0000278;GO:0048765;GO:0010228;GO:0007155;GO:0009640;GO:0010051;GO:0010265;GO:0010090;GO:0016567;GO:0009933;GO:0010162;GO:0005618;GO:0016021;GO:0005886;GO:0005829;GO:0005634 |
| TRINITY_DN380550_c0_g1_i3_m.2741078 | SNW/SKI interacting protein isoform X1 | 0.79 | 0.0369 | GO:0006499;GO:0000398;GO:0042752;GO:0045893;GO:0010228;GO:0005730;GO:0005681 |
| TRINITY_DN391562_c2_g3_i3_m.2835610 | Aspartate aminotransferase A | 0.73 | 0.0035 | GO:0009793;GO:0009095;GO:0009570;GO:0033853;GO:0033854;GO:0004069;GO:0030170 |
| TRINITY_DN542301_c0_g1_i1_m.3625143 | T complex protein 1 subunit theta | 0.73 | 0.0143 | GO:0006457;GO:0051050;GO:0005829;GO:0016020;GO:0009506;GO:0044183;GO:0051082;GO:0005524 |
| TRINITY_DN387753_c0_g1_i1_m.3235166 | zinc finger protein ZPR1 like | 0.63 | 0.0001 | GO:0005737;GO:0005634;GO:0008270 |
| TRINITY_DN981214_c0_g1_i1_m.261264 | heat shock cognate protein 80 | 0.67 | 0.0184 | GO:0006950;GO:0006457;GO:0016787;GO:0051082;GO:0005524 |
| TRINITY_DN395861_c0_g1_i9_m.1519566 | phosphoribosylaminoimidazole carboxylase | 0.75 | 0.0318 | GO:0006189;GO:0046084;GO:0009536;GO:0004639;GO:0004638;GO:0046872;GO:0005524 |
| TRINITY_DN339877_c0_g1_i1_m.1557940 | S adenosylmethionine synthetase 2 | 0.70 | 0.0156 | GO:0006556;GO:0006730;GO:0005829;GO:0004478;GO:0046872;GO:0005524 |
| TRINITY_DN392520_c2_g2_i5_m.1491842 | ruvB like protein 1 | 0.64 | 0.0163 | GO:0032508;GO:2000072;GO:0048507;GO:0006355;GO:0005730;GO:0005829;GO:0009507;GO:0043141;GO:0005524 |
| TRINITY_DN370375_c2_g1_i3_m.2678177 | histone deacetylase HDAC2 | 0.43 | 0.0231 | GO:0046872 |
| TRINITY_DN396893_c2_g1_i8_m.2566893 | geranylgeranyl pyrophosphate synthase | 0.58 | 0.0048 | GO:0051501;GO:0008299;GO:0005739;GO:0004311 |
| TRINITY_DN379907_c0_g1_i10_m.2424353 | cytochrome b5 isoform E | 0.49 | 0.0493 | GO:0016021;GO:0046872;GO:0020037 |
| TRINITY_DN397008_c2_g2_i8_m.2504541 | G type lectin S receptor like serine/threonine protein kinase At2g19130 | 0.44 | 0.0268 | GO:0006468;GO:0048544;GO:0016021;GO:0005886;GO:0004674;GO:0005524 |
| TRINITY_DN395756_c1_g1_i7_m.1781977 | 26S proteasome non ATPase regulatory subunit 12 homolog A like | 0.82 | 0.0017 | GO:0043161;GO:0005737;GO:0031595;GO:0016021;GO:0008541 |
| TRINITY_DN372942_c0_g1_i10_m.1993049 | aldehyde dehydrogenase family 2 member C4 like | 0.82 | 0.0203 | GO:0055114;GO:0004029 |
| TRINITY_DN395532_c1_g1_i2_m.3042283 | putative 2 oxoglutarate/F | 0.72 | 0.0129 | GO:0055114;GO:0005506;GO:0051213 |
| TRINITY_DN372307_c1_g1_i1_m.2127630 | serine/arginine rich splicing factor SR34 isoform X2 | 0.69 | 0.0038 | GO:0005739;GO:0003676;GO:0000166 |
| TRINITY_DN391407_c0_g1_i4_m.3346960 | beta adaptin like protein A | 0.80 | 0.0008 | GO:0006886;GO:0016192;GO:0030131;GO:0008565 |
| TRINITY_DN388980_c0_g1_i2_m.1605996 | E3 ubiquitin protein ligase UPL3 | 0.77 | 0.0324 | GO:0042023;GO:0016567;GO:0010091;GO:0005886;GO:0004842;GO:0016874 |
| TRINITY_DN396009_c5_g1_i3_m.1773689 | AF123610_8 cytochrome P450 | 0.64 | 0.0084 | GO:0009808;GO:0044550;GO:0055114;GO:0016020;GO:0020037;GO:0016710;GO:0005506 |
| TRINITY_DN398843_c4_g4_i2_m.1564493 | benzyl alcohol O benzoyltransferase like | 0.82 | 0.0027 | GO:0016740 |
| TRINITY_DN397966_c2_g1_i2_m.2057111 | probable chromatin remodeling complex ATPase chain | 0.57 | 0.0047 | GO:0043044;GO:0016589;GO:0004386;GO:0031491;GO:0016887;GO:0003677;GO:0005524 |
| TRINITY_DN395742_c3_g1_i6_m.1780819 | T complex protein 1 subunit alpha like | 0.83 | 0.0383 | GO:0006457;GO:0005829;GO:0051082;GO:0005524 |
| TRINITY_DN359951_c5_g1_i1_m.2683331 | Peptidyl prolyl cis trans isomerase H | 0.77 | 0.0024 | GO:0000413;GO:0006457;GO:0005773;GO:0005829;GO:0005886;GO:0003755 |
| TRINITY_DN374018_c0_g1_i10_m.3035148 | hexokinase 5 | 0.80 | 0.0047 | GO:0009749;GO:0046835;GO:0051156;GO:0001678;GO:0006096;GO:0009707;GO:0016021;GO:0005829;GO:0005739;GO:0008865;GO:0004340;GO:0019158;GO:0005524;GO:0005536 |
| TRINITY_DN347063_c0_g1_i2_m.1739525 | Sulfotransferase 16 | 0.83 | 0.0381 | GO:0008146 |
| TRINITY_DN397946_c0_g2_i2_m.2061665 | guanine nucleotide exchange factor SPIKE 1 | 0.81 | 0.0051 | GO:0010928;GO:0016192;GO:0043547;GO:0009958;GO:0008064;GO:0008360;GO:0007264;GO:0019898;GO:0005829;GO:0005886;GO:0005634;GO:0070971;GO:0005089 |
| TRINITY_DN380935_c0_g2_i11_m.936991 | phytochrome associated serine/threonine protein phosphatase 1 | 0.77 | 0.0138 | GO:0006470;GO:0004721 |
| TRINITY_DN290021_c0_g1_i1_m.3896708 | beta glucosidase BoGH3B like | 0.61 | 0.0011 | GO:0009251;GO:0004338 |

| | | | | |
|---|---|---|---|---|
| TRINITY_DN398714_c3_g1_i1_m.1995748 | DEAD box ATP dependent RNA helicase 38 | 0.71 | 0.0000 | GO:0016973;GO:0009737;GO:0009409;GO:0009408;GO:0010501;GO:0006413;GO:0010468;GO:0005737;GO:0005635;GO:0005886;GO:0004004;GO:0003723;GO:0005524 |
| TRINITY_DN383773_c1_g1_i8_m.1705820 | PTI1 like tyrosine protein kinase At3g15890 | 0.80 | 0.0285 | GO:0006468;GO:0004674;GO:0005524 |
| TRINITY_DN393840_c2_g1_i11_m.3304885 | Pleiotropic drug resistance protein 4 | 0.82 | 0.0341 | GO:0055085;GO:0016021;GO:0005886;GO:0042626;GO:0005524 |
| TRINITY_DN383333_c1_g1_i10_m.980547 | DEAD box ATP dependent RNA helicase 5 | 0.53 | 0.0385 | GO:0001510;GO:0010501;GO:0006364;GO:0005730;GO:0005829;GO:0005739;GO:0004004;GO:0003723;GO:0005524 |
| TRINITY_DN385815_c3_g2_i6_m.3099303 | ferredoxin 3 | 0.32 | 0.0152 | GO:0022900;GO:0009507;GO:0046872;GO:0051537;GO:0009055 |
| TRINITY_DN370649_c1_g1_i8_m.2219720 | glutamine synthetase 1a | 0.53 | 0.0137 | GO:0006542;GO:0005737;GO:0004356;GO:0005524 |
| TRINITY_DN397220_c0_g2_i17_m.1026799 | Splicing factor 3B subunit 4 | 0.55 | 0.0343 | GO:0003676;GO:0000166 |
| TRINITY_DN389070_c0_g2_i2_m.2984398 | adenosine kinase 2 | 0.77 | 0.0153 | GO:0016310;GO:0006167;GO:0006166;GO:0004001 |
| TRINITY_DN395088_c4_g1_i1_m.2279116 | 50S ribosomal protein L2 | 0.52 | 0.0186 | GO:0006412;GO:0015934;GO:0003735;GO:0016740;GO:0019843 |
| TRINITY_DN317619_c0_g2_i1_m.2739164 | succinyl CoA ligase subunit alpha | 0.60 | 0.0013 | GO:0008152;GO:0016874;GO:0048037 |
| TRINITY_DN367778_c0_g1_i2_m.1694211 | H/ACA ribonucleoprotein complex subunit 3 like protein | 0.67 | 0.0234 | GO:0042254;GO:0010197;GO:0001522;GO:0009536;GO:0005730;GO:0030529;GO:0019013;GO:0015030;GO:0030515 |
| TRINITY_DN380526_c2_g1_i19_m.2743094 | 60S ribosomal protein L5 1 | 0.61 | 0.0429 | GO:0006412;GO:0000027;GO:0022625;GO:0003735;GO:0008097 |
| TRINITY_DN395617_c1_g1_i2_m.2557497 | auxilin related protein 2 like | 0.68 | 0.0341 | GO:0007076 |
| TRINITY_DN399834_c5_g1_i1_m.2042779 | transcription initiation factor IIF subunit alpha isoform X2 | 0.61 | 0.0348 | GO:0032968;GO:0006367;GO:0006413;GO:0015979;GO:0009522;GO:0016021;GO:0005634;GO:0003824;GO:0003677;GO:0003743 |
| TRINITY_DN394948_c0_g2_i2_m.2430045 | Apoptotic chromatin condensation inducer in the nucleus | 0.52 | 0.0033 | GO:0051276;GO:0033043;GO:0022402;GO:0006259;GO:0044444;GO:0043231 |
| TRINITY_DN380518_c3_g2_i4_m.2742043 | calcium dependent protein kinase | 0.40 | 0.0075 | GO:0018105;GO:0009738;GO:0035556;GO:0046777;GO:0005737;GO:0005634;GO:0005886;GO:0004683;GO:0009931;GO:0005509;GO:0005524;GO:0005516 |
| TRINITY_DN372839_c0_g1_i3_m.1408984 | probable 26S proteasome complex subunit sem1 1 | 0.46 | 0.0181 | GO:0000724;GO:0043248;GO:0006508;GO:0006406;GO:0005634;GO:0008541 |
| TRINITY_DN31340_c0_g1_i1_m.4445752 | superoxide dismutase | 0.38 | 0.0288 | GO:0019430;GO:0055114;GO:0046872;GO:0004784 |
| TRINITY_DN398911_c3_g2_i3_m.1277821 | serine/threonine protein kinase BLUS1 like isoform X2 | 0.77 | 0.0176 | GO:0023014;GO:0005737;GO:0005524;GO:0004702 |
| TRINITY_DN383275_c2_g1_i1_m.3049518 | 50S ribosomal protein L15 | 0.76 | 0.0219 | GO:0006412;GO:0015934;GO:0003735;GO:0019843 |
| TRINITY_DN394713_c5_g1_i1_m.2632767 | glutamate glyoxylate aminotransferase 2 isoform X1 | 0.75 | 0.0367 | GO:0009058;GO:0008483;GO:0030170 |
| TRINITY_DN399044_c5_g1_i1_m.2653256 | Glucan endo 1,3 beta glucosidase 13 | 0.68 | 0.0074 | GO:0005975;GO:0006177;GO:0046658;GO:0016462;GO:0003922;GO:0030247;GO:0004553;GO:0005524 |
| TRINITY_DN396120_c0_g2_i15_m.3080858 | Sorting nexin 1 | 0.82 | 0.0028 | GO:0051788;GO:0006897;GO:0048364;GO:0008333;GO:0016050;GO:0009958;GO:0010252;GO:0006511;GO:0006623;GO:0009853;GO:0006896;GO:0019898;GO:0043231;GO:0005829;GO:0030904;GO:0005771;GO:0035091 |
| TRINITY_DN373985_c0_g1_i1_m.2357695 | Carbamoyl phosphate synthase small chain | 0.82 | 0.0035 | GO:0044205;GO:0000050;GO:0006207;GO:0006541;GO:0006526;GO:0001510;GO:0009570;GO:0005951;GO:0004088;GO:0005524 |
| TRINITY_DN393674_c1_g1_i19_m.1035873 | calmodulin binding transcription activator 5 like | 0.79 | 0.0011 | GO:0045944;GO:0005634;GO:0043565;GO:0001077 |
| TRINITY_DN393069_c1_g1_i1_m.1151831 | cationic peroxidase SPC4 like | 0.44 | 0.0074 | GO:0009664;GO:0006979;GO:0098869;GO:0042744;GO:0055114;GO:0005576;GO:0009505;GO:0020037;GO:0046872;GO:0004601 |
| TRINITY_DN394046_c7_g1_i4_m.898799 | 40S ribosomal protein S18 | 0.67 | 0.0002 | GO:0042254;GO:0006412;GO:0015935;GO:0005829;GO:0003735;GO:0003723 |
| TRINITY_DN367472_c1_g1_i1_m.2615755 | U4/U6 small nuclear ribonucleoprotein PRP4 like protein | 0.82 | 0.0093 | GO:0000398;GO:0046540;GO:0019013;GO:0030621;GO:0017070 |
| TRINITY_DN396407_c1_g1_i2_m.2065592 | Putative receptor protein kinase ZmPK1 | 0.68 | 0.0033 | GO:0006468;GO:0048544;GO:0016021;GO:0005886;GO:0004674;GO:0005524 |
| TRINITY_DN395279_c1_g1_i17_m.1373644 | vacuolar protein sorting associated protein 18 homolog | 0.79 | 0.0077 | GO:0007032;GO:0006904;GO:0006886;GO:0035542;GO:0007040;GO:0030897;GO:0005768;GO:0030674;GO:0008270 |
| TRINITY_DN392217_c2_g1_i9_m.1911807 | eukaryotic translation initiation factor 3 subunit E | 0.81 | 0.0371 | GO:0001731;GO:0009908;GO:0006446;GO:0009640;GO:0005852;GO:0008180;GO:0016282;GO:0005829;GO:0005886;GO:0033290;GO:0003743 |
| TRINITY_DN390652_c1_g3_i1_m.1388512 | stromal 70 kDa heat shock related protein | 0.74 | 0.0105 | GO:0006457;GO:0009536;GO:0051082;GO:0005524 |

| | | | | |
|---|---|---|---|---|
| TRINITY_DN396445_c1_g1_i10_m.2063943 | alanyl tRNA synthetase, putative, expressed | 0.61 | 0.0028 | GO:0006450;GO:0006429;GO:0006419;GO:0004813;GO:0004823;GO:0000049;GO:0002196;GO:0005524 |
| TRINITY_DN362618_c0_g1_i3_m.1677850 | NAD dependent cytosolic glyceraldehyde 3 phosphate dehydrogenase | 0.58 | 0.0007 | GO:0006006;GO:0006096;GO:0055114;GO:0000015;GO:0004365;GO:0051287;GO:0000287;GO:0050661;GO:0004634 |
| TRINITY_DN375421_c0_g1_i2_m.1513082 | aspartate aminotransferase | 0.80 | 0.0240 | GO:0006520;GO:0009058;GO:0005739;GO:0080130;GO:0004069;GO:0030170;GO:0042802 |
| TRINITY_DN399036_c4_g1_i11_m.2648284 | inositol pentakisphosphate 2 kinase IPK1 | 0.64 | 0.0000 | GO:0016310;GO:0046872;GO:0005524;GO:0035299 |
| TRINITY_DN391777_c0_g1_i2_m.2161791 | Dihydrodipicolinate synthase 1 | 0.71 | 0.0033 | GO:0009089;GO:0019877;GO:0009507;GO:0008840 |
| TRINITY_DN376018_c0_g1_i12_m.3354382 | Splicing factor U2af large subunit A | 0.75 | 0.0247 | GO:0008380;GO:0006397;GO:0016607;GO:0089701;GO:0071004;GO:0000243;GO:0030628;GO:0000166;GO:0008187 |
| TRINITY_DN218686_c0_g1_i1_m.3804922 | Germin like protein 5 1 | 0.77 | 0.0353 | GO:0010497;GO:2000280;GO:0033609;GO:0005618;GO:0048046;GO:0009506;GO:0030145;GO:0045735;GO:0046564 |
| TRINITY_DN909827_c0_g1_i1_m.196894 | 30S ribosomal protein S21 | 0.25 | 0.0289 | GO:0006412;GO:0005840;GO:0003735 |
| TRINITY_DN386772_c7_g3_i4_m.2811090 | peptidyl prolyl cis trans isomerase CYP19 3 | 0.61 | 0.0078 | GO:0000413;GO:0006457;GO:0005794;GO:0005739;GO:0003755 |
| TRINITY_DN384891_c0_g1_i8_m.2590046 | UDP D glucuronate decarboxylase | 0.47 | 0.0097 | GO:0005829 |
| TRINITY_DN381352_c1_g1_i8_m.1334769 | 26S proteasome non ATPase regulatory subunit 4 homolog | 0.53 | 0.0218 | GO:0043161;GO:0043248;GO:0005634;GO:0005829;GO:0008540;GO:0031593 |
| TRINITY_DN317159_c0_g1_i2_m.1293783 | mavicyanin like | 0.65 | 0.0101 | GO:0046658;GO:0016021;GO:0009055 |
| TRINITY_DN855020_c0_g1_i1_m.3466374 | ATP synthase epsilon chain | 0.27 | 0.0281 | GO:0042777;GO:0045261;GO:0005886;GO:0046933;GO:0046961;GO:0005524 |
| TRINITY_DN390312_c3_g1_i5_m.2622112 | copper transport protein ATX1 | 0.50 | 0.0006 | GO:0006826;GO:0046916;GO:0005737;GO:0005507 |
| TRINITY_DN741371_c0_g1_i1_m.4247998 | LIM domain containing protein WLIM2b | 0.60 | 0.0211 | GO:0008270 |
| TRINITY_DN383734_c0_g1_i4_m.1705988 | putative DNA repair protein RAD23 | 0.48 | 0.0139 | GO:0043161;GO:0006289;GO:0005634;GO:0003684 |
| TRINITY_DN394974_c1_g1_i15_m.2428929 | protein REDUCED WALL ACETYLATION 4 like | 0.75 | 0.0204 | GO:0016021;GO:0016740 |
| TRINITY_DN388357_c0_g1_i2_m.1980016 | Serine/threonine protein phosphatase 2A 55 kDa regulatory subunit B alpha isoform | 0.79 | 0.0451 | GO:0034047;GO:0000159 |
| TRINITY_DN393272_c2_g1_i11_m.1874020 | Eukaryotic translation initiation factor 5 | 0.78 | 0.0094 | GO:0006413;GO:0003743;GO:0005525 |
| TRINITY_DN376161_c6_g1_i2_m.1046670 | ubiquitin thioesterase OTU1 | 0.79 | 0.0087 | GO:0005829 |
| TRINITY_DN391173_c0_g2_i1_m.2105985 | Protein PROLIFERA | 0.67 | 0.0009 | GO:0032508;GO:0006270;GO:0007049;GO:0005737;GO:0042555;GO:0000347;GO:0003677;GO:0003678;GO:0005524 |
| TRINITY_DN389331_c1_g1_i13_m.2194282 | Cytochrome b5 | 0.80 | 0.0182 | GO:0016021;GO:0046872;GO:0020037 |
| TRINITY_DN354805_c0_g1_i2_m.3195981 | Glycine rich RNA binding protein 2 | 0.78 | 0.0022 | GO:0009409;GO:0005737;GO:0008270;GO:0003676;GO:0000166 |
| TRINITY_DN295087_c0_g1_i1_m.3899549 | tubulin beta 2 chain | 0.77 | 0.0233 | GO:0007017;GO:0005794;GO:0016020;GO:0005874;GO:0003924;GO:0005200;GO:0005525 |
| TRINITY_DN399699_c7_g1_i5_m.3203168 | 12 oxophytodienoic acid reductase 2 | 0.71 | 0.0117 | GO:0031408;GO:0055114;GO:0005739;GO:0003959;GO:0010181 |
| TRINITY_DN376494_c2_g1_i1_m.865752 | serine tRNA ligase like | 0.76 | 0.0038 | GO:0006434;GO:0005737;GO:0016021;GO:0004828;GO:0005524 |
| TRINITY_DN399609_c4_g1_i3_m.3203915 | probable phosphoribosylformylglycinamidine synthase | 0.81 | 0.0332 | GO:0006541;GO:0006189;GO:0009536;GO:0004642;GO:0016740;GO:0005524 |
| TRINITY_DN389027_c0_g2_i5_m.2984722 | structural maintenance of chromosomes protein 3 | 0.80 | 0.0190 | GO:0006281;GO:0007064;GO:0005737;GO:0005819;GO:0000785;GO:0009506;GO:0016363;GO:0003682;GO:0005524 |
| TRINITY_DN384930_c1_g3_i6_m.973293 | hydroquinone glucosyltransferase, putative, expressed | 0.74 | 0.0098 | GO:0009813;GO:0052696;GO:0043231;GO:0080043;GO:0080044 |
| TRINITY_DN395097_c2_g2_i2_m.2278057 | monocopper oxidase like protein SKU5 | 0.63 | 0.0019 | GO:0055114;GO:0009505;GO:0009506;GO:0016722;GO:0005507 |
| TRINITY_DN381124_c0_g1_i2_m.2744650 | Ras related protein Rab7 | 0.77 | 0.0464 | GO:0007264;GO:0015031;GO:0005622;GO:0005886;GO:0005525 |
| TRINITY_DN396947_c2_g2_i2_m.2668452 | Valyl tRNA synthetase | 0.54 | 0.0002 | GO:0006450;GO:0006438;GO:0005829;GO:0016021;GO:0008270;GO:0002161;GO:0004832;GO:0005524 |
| TRINITY_DN387941_c2_g1_i11_m.1829767 | actin related protein 7 | 0.73 | 0.0189 | GO:0006325;GO:0009793;GO:0010227;GO:0009653;GO:0005737;GO:0005634 |
| TRINITY_DN359620_c5_g1_i2_m.3176306 | actin 3 | 0.68 | 0.0119 | GO:0005524 |
| TRINITY_DN362457_c3_g1_i8_m.1862073 | lavin containing monooxygenase 1 | 0.69 | 0.0449 | GO:0055114;GO:0050661;GO:0050660;GO:0004499 |
| TRINITY_DN785783_c0_g1_i1_m.4241934 | sugar ABC transporter substrate binding protein | 0.19 | 0.0421 | GO:0015774;GO:0005856;GO:0016020;GO:0015159 |

| | | | | |
|---|---|---|---|---|
| TRINITY_DN377195_c0_g1_i6_m.3242424 | probable ubiquitin receptor RAD23 isoform X2 | 0.61 | 0.0431 | GO:0043161;GO:0006289;GO:0005634;GO:0003684 |
| TRINITY_DN381171_c4_g2_i3_m.2744209 | Acyl CoA binding domain containing protein 2 | 0.48 | 0.0062 | GO:0016021;GO:0000062 |
| TRINITY_DN210631_c0_g2_i1_m.4127582 | LSU ribosomal protein L28p | 0.39 | 0.0008 | GO:0006412;GO:0005840;GO:0003735 |
| TRINITY_DN355749_c0_g1_i6_m.1741158 | ATP dependent zinc metalloprotease FTSH 8 | 0.73 | 0.0242 | GO:0045041;GO:0006461;GO:0008053;GO:0034982;GO:0042407;GO:0051301;GO:0009535;GO:0016021;GO:0005743;GO:0004176;GO:0004222;GO:0005524;GO:0008270 |
| TRINITY_DN389938_c1_g1_i2_m.1920740 | alpha L arabinofuranosidase 1 | 0.71 | 0.0186 | GO:0046373;GO:0016021;GO:0046556 |
| TRINITY_DN396132_c1_g3_i1_m.3078178 | phosphoglycerate mutase like protein 1 | 0.78 | 0.0052 | GO:0016311;GO:0005829;GO:0009536;GO:0050278 |
| TRINITY_DN342829_c2_g2_i4_m.2093562 | beta tubulin 6 | 0.79 | 0.0001 | GO:0007018;GO:0005737;GO:0005874;GO:0005200;GO:0003924;GO:0005525 |
| TRINITY_DN386163_c0_g2_i2_m.2908777 | 40S ribosomal protein SA | 0.68 | 0.0091 | GO:0000028;GO:0000461;GO:0000447;GO:0006412;GO:0006407;GO:0022627;GO:0030686;GO:0003735 |
| TRINITY_DN398897_c1_g2_i7_m.1565668 | U4/U6 small nuclear ribonucleoprotein Prp31 isoform X1 | 0.70 | 0.0283 | GO:0000244;GO:0046540;GO:0071011;GO:0005690;GO:0019013;GO:0005687 |
| TRINITY_DN381321_c1_g8_i1_m.1337970 | MFP1 attachment factor 1 like | 0.74 | 0.0047 | GO:0016020;GO:0009536 |
| TRINITY_DN985487_c0_g1_i1_m.346269 | presequence protease 2 | 0.46 | 0.0228 | GO:0016485;GO:0004222;GO:0008270 |
| TRINITY_DN365556_c1_g1_i2_m.1182462 | glycine cleavage system H protein 2 | 0.65 | 0.0121 | GO:0019464;GO:0005960;GO:0005739;GO:0005524 |
| TRINITY_DN395488_c3_g1_i5_m.2724713 | 60S ribosomal protein L23a | 0.61 | 0.0311 | GO:0006412;GO:0000027;GO:0009536;GO:0022625;GO:0000166;GO:0003735 |
| TRINITY_DN391424_c1_g1_i5_m.3345662 | Profilin | 0.62 | 0.0437 | GO:0042989;GO:0015629;GO:0005938;GO:0003785 |
| TRINITY_DN367224_c1_g2_i3_m.1107828 | Pleckstrin homology domain containing family A member 8 | 0.75 | 0.0104 | GO:0046836;GO:0009536;GO:0005829;GO:0005886;GO:0051861;GO:0017089 |
| TRINITY_DN360657_c0_g1_i1_m.1268519 | Aspartic proteinase nepenthesin 2 | 0.61 | 0.0168 | GO:0006508;GO:0030163;GO:0004190;GO:0003677 |
| TRINITY_DN371006_c0_g1_i13_m.2072487 | DEAD box ATP dependent RNA helicase 30 | 0.81 | 0.0054 | GO:0000184;GO:0010501;GO:0006364;GO:0005634;GO:0004004;GO:0003723;GO:0005524 |
| TRINITY_DN386748_c0_g1_i2_m.2814494 | Heat shock protein STI | 0.66 | 0.0235 | GO:0051131;GO:0005737;GO:0005634;GO:0070678;GO:0051879 |
| TRINITY_DN366084_c0_g1_i7_m.944944 | dynamin 2A like | 0.53 | 0.0156 | GO:0003924;GO:0005525 |
| TRINITY_DN381721_c1_g3_i3_m.1788890 | GTP binding protein YPTM2 | 0.47 | 0.0434 | GO:0007264;GO:0015031;GO:0005622;GO:0005886;GO:0005525 |
| TRINITY_DN736126_c0_g1_i1_m.4197991 | exocyst complex component SEC10 | 0.61 | 0.0110 | GO:0048278;GO:0006887;GO:0000145;GO:0009506;GO:0005886 |
| TRINITY_DN379994_c0_g1_i4_m.2420324 | syntaxin 121 like | 0.76 | 0.0070 | GO:0006906;GO:0006886;GO:0006887;GO:0048278;GO:0016021;GO:0012505;GO:0005886;GO:0031201;GO:0000149;GO:0005484 |
| TRINITY_DN376603_c1_g2_i20_m.3125966 | tropinone reductase homolog At5g06060 | 0.80 | 0.0121 | GO:0055114;GO:0016021;GO:0005829;GO:0016491 |
| TRINITY_DN496244_c0_g1_i1_m.813146 | ribosomal 40S subunit protein S8B | 0.83 | 0.0317 | GO:0000462;GO:0022627;GO:0003735 |
| TRINITY_DN391503_c1_g1_i9_m.3346224 | MICOS complex subunit mic60 | 0.70 | 0.0083 | GO:0006486;GO:0005743;GO:0016021 |
| TRINITY_DN388211_c2_g1_i10_m.1187648 | SH3 domain containing protein 3 | 0.73 | 0.0001 | GO:0006084;GO:0007020;GO:0016132;GO:0016126;GO:0000023;GO:0043085;GO:0019252 |
| TRINITY_DN243206_c0_g1_i1_m.3885500 | mitogen activated protein kinase kinase 5 | 0.82 | 0.0282 | GO:0023014;GO:0005737;GO:0005524;GO:0004702 |
| TRINITY_DN375923_c4_g1_i5_m.1103935 | vesicle associated protein 1 2 like | 0.67 | 0.0004 | GO:0016021;GO:0005789 |
| TRINITY_DN385982_c0_g2_i8_m.1073774 | 26S protease regulatory subunit 7 | 0.72 | 0.0454 | GO:0030433;GO:1901800;GO:0045899;GO:0005886;GO:0031595;GO:0031597;GO:0008540;GO:0036402;GO:0017025;GO:0008233;GO:0005524 |
| TRINITY_DN386312_c0_g1_i6_m.1131337 | alcohol dehydrogenase like 4 | 0.63 | 0.0050 | GO:0055114;GO:0008270;GO:0016491 |
| TRINITY_DN834291_c0_g1_i1_m.3441984 | Cytochrome P450 89A2 | 0.74 | 0.0370 | GO:0052696;GO:0044550;GO:0009813;GO:0055114;GO:0016021;GO:0043231;GO:0020037;GO:0016709;GO:0080043;GO:0080044;GO:0005506 |
| TRINITY_DN387355_c0_g1_i6_m.1541699 | 20 kDa chaperonin | 0.59 | 0.0014 | GO:0006986;GO:0051085;GO:1901671;GO:0005759;GO:0009507;GO:0046914;GO:0051087;GO:0051082;GO:0005524 |
| TRINITY_DN281449_c0_g2_i1_m.3956226 | cytochrome oxidase subunit 1 | 0.47 | 0.0168 | GO:0009060;GO:1902600;GO:0022900;GO:0006119;GO:0016021;GO:0005743;GO:0005886;GO:0070469;GO:0004129;GO:0020037;GO:0005507;GO:0005506 |
| TRINITY_DN398979_c4_g1_i7_m.1283243 | eukaryotic translation initiation factor 3 subunit A | 0.53 | 0.0122 | GO:0001731;GO:0006446;GO:0005852;GO:0016282;GO:0005829;GO:0005886;GO:0033290;GO:0003743 |
| TRINITY_DN372689_c0_g1_i9_m.2027489 | Endoglucanase 11 | 0.59 | 0.0497 | ------ |
| TRINITY_DN384828_c0_g1_i7_m.2594458 | WD repeat containing protein DWA2 like | 0.72 | 0.0005 | ------ |

| | | | | |
|---|---|---|---|---|
| TRINITY_DN384453_c0_g1_i27_m.1580531 | syntaxin 132 | 0.75 | 0.0154 | ------ |
| TRINITY_DN804239_c0_g1_i1_m.3510999 | PX domain containing protein | 0.78 | 0.0303 | ------ |
| TRINITY_DN398457_c5_g2_i2_m.2870135 | protein phosphatase 2A structural subunit | 0.72 | 0.0136 | ------ |
| TRINITY_DN397466_c2_g1_i10_m.1051725 | SEC13 related protein | 0.59 | 0.0481 | ------ |
| TRINITY_DN346356_c0_g1_i1_m.2947123 | protein EARLY RESPONSIVE TO DEHYDRATION 15 | 0.54 | 0.0476 | ------ |
| TRINITY_DN394454_c0_g2_i1_m.1162548 | translation machinery associated protein 7 like | 0.46 | 0.0151 | ------ |
| TRINITY_DN374529_c0_g1_i7_m.2413471 | dystrophia myotonica WD repeat containing protein like | 0.81 | 0.0034 | ------ |
| TRINITY_DN365125_c0_g1_i5_m.1645229 | Ankyrin repeat domain containing protein 2 | 0.52 | 0.0000 | ------ |
| TRINITY_DN361768_c0_g1_i5_m.2402328 | porin Gram negative type | 0.46 | 0.0091 | ------ |
| TRINITY_DN399240_c0_g1_i7_m.2318705 | titin isoform X1 | 0.30 | 0.0294 | ------ |
| TRINITY_DN377967_c0_g1_i9_m.2831369 | elicitor responsive gene 3 | 0.76 | 0.0168 | ------ |
| TRINITY_DN399008_c5_g1_i11_m.2648197 | F box protein At1g78280 | 0.79 | 0.0124 | ------ |
| TRINITY_DN385598_c2_g2_i9_m.2338168 | suppressor of the G2 | 0.52 | 0.0342 | ------ |
| TRINITY_DN385781_c0_g1_i1_m.1145203 | wheat cold induced 16 | 0.47 | 0.0420 | ------ |
| TRINITY_DN394536_c0_g1_i11_m.2966415 | WD repeat containing protein 75 | 0.55 | 0.0318 | ------ |
| TRINITY_DN359450_c0_g1_i6_m.1987539 | GRF1 interacting factor 2 | 0.56 | 0.0486 | ------ |
| TRINITY_DN376309_c1_g1_i9_m.3131343 | GLABRA2 expression modulator like | 0.81 | 0.0224 | ------ |
| TRINITY_DN396651_c2_g1_i1_m.2174977 | hsp70 nucleotide exchange factor fes1 like | 0.73 | 0.0012 | ------ |
| TRINITY_DN382391_c0_g3_i2_m.2699674 | protein SPIRAL1 like 1 | 0.64 | 0.0352 | ------ |
| TRINITY_DN229114_c1_g1_i1_m.4097918 | granule associated like protein | 0.32 | 0.0437 | ------ |
| TRINITY_DN385250_c0_g1_i12_m.2393884 | coiled coil domain containing protein 25 | 0.65 | 0.0016 | ------ |
| TRINITY_DN397466_c2_g1_i3_m.1051679 | Protein SEC13 like protein | 0.58 | 0.0348 | ------ |
| TRINITY_DN391257_c1_g1_i19_m.2777977 | plasminogen activator inhibitor 1 RNA binding protein like | 0.55 | 0.0483 | ------ |

DEPs: Differentially expression proteins, CK:control, AS:alkali stress(35mmol.$L^{-1}$ $NaCO_3$:$NaHCO_3$=1:1).
