# Supplementary material for: Proteomic Responses to Alkali Stress in Oats and the Alleviatory Effects of Exogenous Spermine Application
Source: Front Plant Sci. 2021 Apr 1;12:627129. doi: 10.3389/fpls.2021.627129 (PMC8049610; doi:10.3389/fpls.2021.627129)
Supplement: Supplementary file 13 [file Table_5.pdf]

SUPPLEMENTAL TABLE 5 The DEPs of leaves at AS+Spm vs AS

Protein ID	Description	Ratio	P-value	Go number
**Up-regulation**				
TRINITY_DN395470_c1_g1_i8_m.2730616	Arginine decarboxylase	1.45	0.0197	GO:0033388;GO:0006527;GO:0008295;GO:0008792
TRINITY_DN387562_c5_g1_i10_m.2262265	xylanase inhibitor protein 1-like	1.90	0.0154	GO:0006032;GO:0045493;GO:0005576;GO:0004568;GO:0008061
TRINITY_DN397833_c2_g1_i4_m.1946133	ATP-dependent 6-phosphofructokinase 6-like	1.21	0.0278	GO:0006002;GO:0006508;GO:0061615;GO:0005737;GO:0004222;GO:0003872;GO:0046872;GO:0005524
TRINITY_DN398938_c5_g4_i3_m.1279814	3'-N-debenzoyl-2'-deoxytaxol N-benzoyltransferase-like	1.27	0.0226	GO:0016747
TRINITY_DN387831_c0_g1_i9_m.1640573	Serine/threonine-protein kinase STN8	1.23	0.0024	GO:0006468;GO:0042549;GO:0004674;GO:0005524
TRINITY_DN386963_c0_g1_i1_m.2664437	Nuclear-interacting partner of ALK	1.27	0.0275	GO:0016021;GO:0005634;GO:0008270
TRINITY_DN399631_c1_g3_i1_m.3206417	light-induced protein 1-like	1.31	0.0107	GO:0005622
TRINITY_DN368820_c0_g1_i4_m.3056111	Putative invertase inhibitor	1.62	0.0003	GO:0043086, GO:0004857
TRINITY_DN771345_c0_g1_i1_m.4363218	Putative ornithine aminotransferase	1.38	0.0320	GO:0006593,GO:0005759,GO:0004587
TRINITY_DN393592_c1_g1_i4_m.2756609	glutamyl-tRNA reductase	1.43	0.0383	GO:0070681,GO:0005739,GO:0005524
TRINITY_DN371528_c1_g1_i12_m.1648156	glutathione S-transferase lambda1	1.22	0.0052	GO:0006749,GO:0005737,GO:0004364
TRINITY_DN355168_c0_g1_i1_m.2929380	Abscisic stress-ripening protein 2	1.27	0.0426	GO:0006950
TRINITY_DN367024_c0_g1_i6_m.1971459	Photosystem II 10 kDa polypeptide	1.63	0.0231	GO:0015979,GO:0009535
TRINITY_DN855066_c0_g1_i1_m.3466289	cold responsive protein	1.37	0.0248	------
**Down-regulation**				
TRINITY_DN391252_c3_g1_i21_m.2777254	endonuclease 2-like	0.67	0.0178	GO:0006308;GO:0090305;GO:0004519;GO:0003676
TRINITY_DN394962_c1_g1_i4_m.2427962	Lipoxygenase 2.3	0.75	0.0437	GO:0031408;GO:0055114;GO:0009507;GO:0046872;GO:0016165
TRINITY_DN318416_c0_g1_i3_m.916773	AAA-ATPase At3g50940-like	0.41	0.0185	GO:0005524
TRINITY_DN365517_c0_g1_i1_m.1183396	cell division AAA ATPase family protein	0.58	0.0287	GO:0051301;GO:0016021;GO:0005524
TRINITY_DN393650_c2_g1_i15_m.1033689	UDP-glucuronic acid decarboxylase 2-like	0.80	0.0140	GO:0016021
TRINITY_DN395289_c5_g1_i1_m.1374412	70 kDa peptidyl-prolyl isomerase-like	0.66	0.0459	GO:0061077;GO:0000413;GO:0005789;GO:0005528;GO:0003755
TRINITY_DN387588_c0_g1_i1_m.2260698	Putative gibberellin receptor GID1L3	0.54	0.0117	GO:0009423;GO:0033587;GO:0009073;GO:0009507;GO:0003849
TRINITY_DN377608_c2_g3_i1_m.3222128	tuliposide A-converting enzyme 2	0.70	0.0123	GO:0008152;GO:0016787
TRINITY_DN151429_c0_g1_i1_m.650610	B12D protein/NADH-ubiquinone reductase complex 1 MLRQ subunit	0.71	0.0133	GO:0016021
TRINITY_DN379801_c2_g1_i3_m.2459734	vacuolar-processing enzyme beta-isozyme 1-like	0.70	0.0393	GO:0006624;GO:0051603;GO:0005773;GO:0004197
TRINITY_DN389469_c2_g2_i6_m.1812626	Pathogenesis-related protein STH-21	0.49	0.0440	GO:0050896;GO:0050790;GO:0050794;GO:0044424
TRINITY_DN329781_c0_g1_i1_m.3164828	1-aminocyclopropane-1-carboxylate oxidase-1-like protein	0.82	0.0207	GO:0055114;GO:0005506;GO:0051213
TRINITY_DN362225_c0_g1_i1_m.2468766	phenylalanine ammonia-lyase	0.73	0.0056	GO:0010264;GO:0005739
TRINITY_DN389748_c1_g1_i1_m.997647	3-ketoacyl-CoA thiolase 2	0.73	0.0106	GO:0008152;GO:0003988
TRINITY_DN359715_c0_g1_i2_m.1514136	Eukaryotic translation initiation factor 5	0.78	0.0453	GO:0006413;GO:0003743;GO:0005525
TRINITY_DN384843_c0_g1_i4_m.2592006	thiamine thiazole synthase 2	0.79	0.0144	GO:0055114;GO:0010155;GO:0009228;GO:0042742;GO:0009637;GO:0046777;GO:0006974;GO:0052837;GO:0000023;GO:0043085;GO:0019252;GO:0009941;GO:0009570;GO:0010319;GO:0009579;GO:0005829;GO:0016491;GO:0008270
TRINITY_DN375173_c1_g1_i4_m.3262767	caffeic acid-O-methyltransferase	0.78	0.0030	GO:0009809;GO:0009813;GO:0032259;GO:0005829;GO:0016206;GO:0047763;GO:0046983
TRINITY_DN391911_c0_g1_i3_m.1856800	pyruvate dehydrogenase E1 component subunit	0.83	0.0023	GO:0006086;GO:0006096;GO:0055114;GO:0009941;GO:0009570;GO:0004739

	alpha-3			
TRINITY_DN393761_c0_g1_i10_m.3180472	Phosphate carrier protein	0.72	0.0434	GO:0006810;GO:0009723;GO:0009651;GO:0006412;GO:0016021;GO:0003735
TRINITY_DN398623_c1_g3_i1_m.2656500	5-methyltetrahydropteroyltriglutamate--homocysteine methyltransferase 1	0.80	0.0172	GO:0032259;GO:0050667;GO:0009086;GO:0005829;GO:0005576;GO:0008705;GO:0008270;GO:0003871
TRINITY_DN368573_c3_g2_i1_m.1507045	Thiazole biosynthetic enzyme 1-2	0.73	0.0058	GO:0055114;GO:0010155;GO:0009228;GO:0042742;GO:0009637;GO:0046777;GO:0006974;GO:0052837;GO:0000023;GO:0043085;GO:0019252;GO:0009941;GO:0009570;GO:0010319;GO:0009579;GO:0005829;GO:0016491;GO:0008270
TRINITY_DN385729_c1_g2_i8_m.1145747	phosphate transporter 6	0.82	0.0285	GO:0006817;GO:0055085;GO:0005887;GO:0022891;GO:0005315
TRINITY_DN360553_c2_g1_i8_m.1003782	26S protease regulatory subunit 6A-like protein	0.82	0.0407	GO:0030433;GO:1901800;GO:0045899;GO:0031595;GO:0031597;GO:0008540;GO:0036402;GO:0017025;GO:0008233;GO:0005524
TRINITY_DN376164_c0_g1_i3_m.1048354	Polyol transporter 5	0.70	0.0442	GO:0015992;GO:0046323;GO:1904659;GO:0005887;GO:0005355;GO:0005351
TRINITY_DN393046_c2_g1_i1_m.1154309	Peroxidase 12	0.82	0.0414	GO:0009664;GO:0006979;GO:0098869;GO:0042744;GO:0055114;GO:0005576;GO:0009505;GO:0020037;GO:0046872;GO:0004601
TRINITY_DN395714_c2_g2_i5_m.1783572	LRR receptor-like serine/threonine-protein kinase	0.65	0.0118	GO:0016310;GO:0016020;GO:0000166;GO:0004672

DEPs: Differentially expression proteins, AS:alkali stress(35mmol.L^{-1} $NaCO_3$:$NaHCO_3$=1:1), AS+Spm:35mmol.L^{-1} $NaCO_3$:$NaHCO_3$(1:1)+0.01mmol.L^{-1} spermine.
