# Supplementary material for: Proteomic Responses to Alkali Stress in Oats and the Alleviatory Effects of Exogenous Spermine Application
Source: Front Plant Sci. 2021 Apr 1;12:627129. doi: 10.3389/fpls.2021.627129 (PMC8049610; doi:10.3389/fpls.2021.627129)
Supplement: Supplementary file 14 [file Table_6.pdf]

SUPPLEMENTAL TABLE 6 The DEPs of roots at AS+Spm vs AS

Protein ID	Description	Ratio	P-value	Go number
**Up-regulation**				
TRINITY_DN388501_c0_g1_i3_m.2462822	sucrose synthase	2.60	0.0029	GO:0005985;GO:0016157
TRINITY_DN171701_c0_g1_i1_m.577450	vacuolar ATP synthase catalytic subunit a	1.82	0.0310	GO:0044267;GO:0015991;GO:0046034;GO:0007035;GO:0090463;GO:0090465;GO:0090464;GO:0055114;GO:0005829;GO:0000329;GO:0033180;GO:0050661;GO:0050660;GO:0004499;GO:0005524;GO:0046961
TRINITY_DN361148_c1_g1_i2_m.1753490	50S ribosomal protein L29	1.55	0.0468	GO:0006412;GO:0005840;GO:0003735
TRINITY_DN369975_c2_g2_i2_m.2128675	Reticuline oxidase-like protein	1.60	0.0287	GO:0055114;GO:0050660;GO:0016614
TRINITY_DN322423_c1_g2_i1_m.1661457	glycoside hydrolase family 7	2.72	0.0135	GO:0030245;GO:0005576;GO:0030248;GO:0004553
TRINITY_DN389810_c1_g1_i5_m.2490232	chitinase	1.74	0.0237	GO:0005975;GO:0006032;GO:0005576;GO:0004568;GO:0008061
TRINITY_DN370407_c0_g4_i4_m.3085052	polyribonucleotide nucleotidyltransferase	1.27	0.0183	GO:0090503;GO:0006402;GO:0006396;GO:0005737;GO:0004654;GO:0000287;GO:0000175;GO:0003723
TRINITY_DN950949_c0_g1_i1_m.296234	OmpA family protein	2.63	0.0121	GO:0016021;GO:0009279
TRINITY_DN358839_c0_g1_i16_m.1681041	elongation factor Tu	1.21	0.0079	GO:0006414;GO:0005737;GO:0003746;GO:0003924;GO:0005525
TRINITY_DN372334_c1_g1_i8_m.2125737	mavicyanin-like	1.77	0.0117	GO:0046658;GO:0009055
TRINITY_DN379389_c2_g2_i2_m.1906524	lipoxygenase	1.32	0.0258	GO:0031408;GO:0050832;GO:0002215;GO:0055114;GO:0016165;GO:0046872
TRINITY_DN73844_c0_g1_i1_m.4519729	30S ribosomal protein S1	1.23	0.0477	GO:0006412;GO:0005840;GO:0003735;GO:0003723
TRINITY_DN366217_c2_g2_i9_m.2500472	glutathione S-transferase GSTF1	1.34	0.0048	GO:0006749;GO:0005737;GO:0004364
TRINITY_DN309296_c0_g1_i1_m.2876981	50S ribosomal protein L22	1.22	0.0411	GO:0006412;GO:0022625;GO:0003735;GO:0019843
TRINITY_DN360746_c0_g1_i2_m.1664556	phosphate transporter	1.65	0.0226	GO:0006817;GO:0055085;GO:0016021;GO:0009536;GO:0005315
TRINITY_DN87505_c0_g1_i1_m.4533112	glyceraldehyde-3-phosphate dehydrogenase	1.37	0.0403	GO:0006006;GO:0006096;GO:0055114;GO:0005737;GO:0004365;GO:0051287;GO:0050661
TRINITY_DN391907_c0_g1_i3_m.1853034	LRR receptor-like protein kinase 2	1.93	0.0075	GO:0006468;GO:0016021;GO:0005739;GO:0005886;GO:0004674;GO:0005524
TRINITY_DN396451_c2_g2_i8_m.2063441	aleurone ribonuclease	1.75	0.0408	GO:0090502;GO:0033897;GO:0003723
TRINITY_DN193217_c0_g1_i1_m.531296	polyphenol oxidase	1.43	0.0412	GO:0055114;GO:0046148;GO:0004503;GO:0046872;GO:0004097
TRINITY_DN346344_c0_g2_i2_m.2947108	Nucleoside diphosphate kinase 1	1.80	0.0440	GO:0006228;GO:0006241;GO:0006183;GO:0006165;GO:0005622;GO:0004550;GO:0046872;GO:0005524
TRINITY_DN367766_c0_g1_i8_m.1693602	sucrose synthase type 3	1.58	0.0001	GO:0005985;GO:0016157
TRINITY_DN398030_c3_g1_i13_m.953156	Beta-glucosidase 6	1.76	0.0456	GO:1901657;GO:0030245;GO:0005829;GO:0102483;GO:0008422
TRINITY_DN355421_c3_g1_i3_m.2825170	glucan endo-1,3-beta-glucosidase, acidic isoform	1.31	0.0236	GO:0005975;GO:0046658;GO:0030247;GO:0004553
TRINITY_DN264745_c0_g1_i3_m.4139715	ABC transporter substrate-binding protein	1.40	0.0267	GO:0006810;GO:0005215
TRINITY_DN282840_c0_g1_i1_m.4051903	30S ribosomal protein S3	1.31	0.0103	GO:0006412;GO:0015935;GO:0003735;GO:0019843;GO:0003729
TRINITY_DN392171_c0_g1_i9_m.2203412	DNA topoisomerase 1	1.25	0.0411	GO:0006338;GO:0006265;GO:0006260;GO:0007059;GO:0005730;GO:0031298;GO:0003917;GO:0003677;GO:0003918
TRINITY_DN396088_c1_g1_i13_m.1772842	Patatin group A-3	1.39	0.0404	GO:0016042;GO:0006952;GO:0005737;GO:0016020;GO:0004620;GO:0047372
TRINITY_DN388838_c0_g1_i11_m.1730580	Membrane protein of ER body 2-like isoform X1	1.30	0.0049	GO:0000041;GO:0046916;GO:0098662;GO:0043231;GO:0016020;GO:0046915
TRINITY_DN368681_c1_g2_i3_m.1040132	GST	1.33	0.0022	GO:0006749;GO:0009407;GO:0005737;GO:0004364
TRINITY_DN399495_c11_g1_i5_m.1285756	isovaleryl-CoA dehydrogenase	1.81	0.0375	GO:0033539;GO:0006552;GO:0055088;GO:0005739;GO:0016021;GO:0050660;GO:0052890;GO:0000062;GO:0008470;GO:0009055
TRINITY_DN392237_c3_g1_i3_m.1907354	30S ribosomal protein S6	1.30	0.0266	GO:0006412;GO:0005840;GO:0003735;GO:0019843

TRINITY_DN387648_c1_g1_i11_m.3106792	cytochrome b561 and DOMON domain-containing protein At4g17280-like	1.22	0.0395	GO:0055114;GO:0016021
TRINITY_DN390847_c1_g1_i2_m.2287777	metal transporter Nramp2	1.23	0.0135	GO:0055072;GO:0006811;GO:0016021;GO:0005215
TRINITY_DN388359_c4_g4_i1_m.1978766	elongation factor G	1.52	0.0396	GO:0006414;GO:0005737;GO:0003746;GO:0003924;GO:0005525
TRINITY_DN339665_c0_g1_i2_m.2109730	2-hydroxyisoflavanone dehydratase	1.42	0.0028	GO:0008152;GO:0016787
TRINITY_DN387440_c0_g1_i5_m.2381371	probable serine protease EDA2	1.22	0.0319	GO:0006508;GO:0008239;GO:0004185
TRINITY_DN317132_c0_g1_i1_m.1293937	DNA-directed RNA polymerase subunit alpha	1.28	0.0037	GO:0006351;GO:0003899;GO:0046983;GO:0003677
TRINITY_DN377811_c0_g1_i13_m.1087158	Arginine/serine-rich-splicing factor RSP31	1.23	0.0138	GO:0003676;GO:0000166
TRINITY_DN361813_c0_g1_i2_m.1571332	Serine carboxypeptidase 2	1.32	0.0397	GO:0051603;GO:0005773;GO:0005576;GO:0005739;GO:0004185
TRINITY_DN371641_c2_g3_i2_m.2887268	NADH dehydrogenase subunit 6	1.32	0.0114	GO:0055114;GO:0016021;GO:0005747;GO:0008137
TRINITY_DN393761_c0_g1_i10_m.3180472	Phosphate carrier protein	1.40	0.0344	GO:0006810;GO:0009723;GO:0009651;GO:0006412;GO:0016021;GO:0003735
TRINITY_DN176725_c0_g2_i1_m.509562	phosphoenolpyruvate carboxykinase	1.31	0.0161	GO:0016310;GO:0006094;GO:0005737;GO:0016301;GO:0030145;GO:0004613;GO:0005525
TRINITY_DN383486_c0_g1_i3_m.2610452	phospho-2-dehydro-3-deoxyheptonate aldolase 2	1.52	0.0325	GO:0009073;GO:0009536;GO:0003849
TRINITY_DN395110_c0_g1_i8_m.2843907	cytochrome b561, DM13 and DOMON domain-containing protein At5g54830	1.46	0.0004	GO:0055114;GO:0016021
TRINITY_DN16407_c0_g1_i1_m.4415582	RNA polymerase sigma factor RpoD	1.25	0.0111	GO:0001123;GO:0006355;GO:0005737;GO:0003700;GO:0016987;GO:0003677
TRINITY_DN395217_c3_g3_i1_m.1370528	Germin-like protein 4-1	1.34	0.0253	GO:0033609;GO:0005618;GO:0048046;GO:0030145;GO:0045735;GO:0046564
TRINITY_DN392732_c1_g2_i4_m.904885	alternative oxidase	1.23	0.0180	GO:0055114;GO:0016021;GO:0005739;GO:0070469;GO:0009916;GO:0046872
TRINITY_DN398919_c2_g1_i8_m.1281963	Phosphoinositide phospholipase C 2	1.31	0.0019	GO:0035556;GO:0016042;GO:0005739;GO:0004435;GO:0004871
TRINITY_DN388147_c2_g1_i10_m.1808979	monodehydroascorbate reductase	1.30	0.0444	GO:0022900;GO:0045454;GO:0005623;GO:0050660;GO:0016656;GO:0015036
TRINITY_DN353247_c0_g1_i3_m.3365426	1-aminocyclopropane-1-carboxylate oxidase	1.48	0.0197	GO:0055114;GO:0009693;GO:0005506;GO:0009815;GO:0051213
TRINITY_DN374351_c5_g1_i7_m.1768499	1,3-beta glucanase	1.62	0.0362	GO:0006952;GO:0005975;GO:0042973
TRINITY_DN395713_c3_g2_i4_m.1786010	glycine-rich RNA-binding protein GRP2A	1.28	0.0328	GO:0045087;GO:0000380;GO:0009735;GO:0010119;GO:0006406;GO:0010228;GO:0006970;GO:0010501;GO:0032508;GO:0005829;GO:0005777;GO:0005634;GO:0048046;GO:0009507;GO:0009506;GO:0003697;GO:0000166;GO:0003690;GO:0003729
TRINITY_DN382045_c0_g1_i6_m.3191285	vesicle-associated membrane protein 721-like	1.27	0.0163	GO:0006887;GO:0006906;GO:0031201;GO:0016021;GO:0000149;GO:0005484
TRINITY_DN358296_c1_g1_i3_m.2976061	benzyl alcohol O-benzoyltransferase-like	1.23	0.0441	GO:0016747
TRINITY_DN399288_c2_g2_i1_m.2319935	Putative LRR receptor-like serine/threonine-protein kinase	1.61	0.0270	GO:0006468;GO:0016021;GO:0004674;GO:0005524
TRINITY_DN382438_c1_g1_i6_m.2032609	cellulose synthase-like protein E6	1.39	0.0373	GO:0071555;GO:0030244;GO:0016021;GO:0005739;GO:0000139;GO:0016760
TRINITY_DN389748_c1_g1_i1_m.997647	3-ketoacyl-CoA thiolase 2	1.25	0.0348	GO:0008152;GO:0003988
TRINITY_DN394295_c0_g2_i10_m.2224776	probable uridine nucleosidase 1	1.21	0.0204	GO:0006218;GO:0006152;GO:0005829;GO:0045437;GO:0047724;GO:0072585;GO:0047622
TRINITY_DN378149_c2_g2_i4_m.2684689	Translocation protein SEC62	1.26	0.0113	GO:0015031;GO:0016021;GO:0005886;GO:0008565
TRINITY_DN392732_c1_g7_i1_m.904859	ubiquinol oxidase 2	1.28	0.0482	GO:0055114;GO:0016021;GO:0005739;GO:0070469;GO:0009916;GO:0046872
TRINITY_DN378854_c1_g2_i1_m.3293814	peptidyl-prolyl cis-trans isomerase FKBP42	1.22	0.0124	GO:0000413;GO:0030244;GO:0048366;GO:0061077;GO:0016021;GO:0005886;GO:0005789;GO:0005528;GO:0005516;GO:0003755
TRINITY_DN395528_c0_g2_i7_m.3045280	Hsp90-like protein	1.59	0.0373	GO:0006457;GO:0010075;GO:0009306;GO:0009414;GO:0009651;GO:0009934;GO:0005788;GO:0005739;GO:0005774;GO:0048046;GO:0005886;GO:0009507;GO:0005634;GO:0009506;GO:0051082;GO:0005524
TRINITY_DN398262_c1_g3_i7_m.1377399	ABC transporter B family member 21-like	1.36	0.0369	GO:0055085;GO:0043190;GO:0042626;GO:0005524
TRINITY_DN315876_c0_g1_i1_m.3166231	Formamidase	1.22	0.0321	GO:0016811

TRINITY_DN392682_c1_g1_i12_m.2119493	calreticulin-3	1.23	0.0008	GO:0009697;GO:0010204;GO:0006995;GO:0006457;GO:0046283;GO:0055074;GO:0009627;GO:0009626;GO:0042742;GO:0034976;GO:0016021;GO:0005789;GO:0051082;GO:0005509
TRINITY_DN353140_c0_g1_i1_m.2737169	cold acclimation protein WCOR410c	1.25	0.0146	GO:0009737;GO:0009631;GO:0009414;GO:0005829;GO:0016020
TRINITY_DN391503_c1_g1_i9_m.3346224	MICOS complex subunit mic60	1.47	0.0068	GO:0006486;GO:0005743;GO:0016021
TRINITY_DN231000_c0_g1_i1_m.4087030	50S ribosomal protein L13	1.24	0.0192	GO:0006412;GO:0005840;GO:0003735
TRINITY_DN399628_c5_g1_i11_m.3204538	Nucleolar GTP-binding protein 1	1.23	0.0147	GO:0042254;GO:0005730;GO:0005525
TRINITY_DN395678_c6_g1_i2_m.2551249	alpha-tubulin	1.20	0.0391	GO:0007017;GO:0007010;GO:0010498;GO:0006094;GO:0005829;GO:0005886;GO:0005874;GO:0003924;GO:0005200;GO:0004553;GO:0005525
TRINITY_DN397008_c2_g2_i8_m.2504541	G-type lectin S-receptor-like serine/threonine-protein kinase At2g19130	1.25	0.0097	GO:0006468;GO:0048544;GO:0016021;GO:0005886;GO:0004674;GO:0005524
TRINITY_DN349697_c2_g1_i2_m.1401203	50S ribosomal protein L6	1.30	0.0286	GO:0006412;GO:0005840;GO:0003735;GO:0019843
TRINITY_DN392786_c1_g2_i1_m.907335	two pore potassium channel a	1.22	0.0178	GO:0007030;GO:0071805;GO:0051260;GO:0000271;GO:0006816;GO:0030007;GO:0010119;GO:0009651;GO:0010029;GO:0031004;GO:0009705;GO:0005509;GO:0005242
TRINITY_DN181509_c0_g1_i1_m.488631	C4-dicarboxylate ABC transporter substrate-binding protein	1.71	0.0132	------
TRINITY_DN605235_c0_g1_i1_m.106711	outer membrane adhesin-like protein	1.89	0.0308	------
TRINITY_DN144314_c0_g1_i1_m.401849	Fimbrial protein precursor	1.41	0.0065	------
TRINITY_DN14734_c0_g1_i1_m.4410580	Calreticulin	1.28	0.0438	------
TRINITY_DN396377_c0_g2_i1_m.2805415	Isoflavone reductase-like protein	1.33	0.0485	------
TRINITY_DN74804_c0_g1_i1_m.4483696	methylation site containing protein	1.24	0.0024	------
**Down-regulation**				
TRINITY_DN359075_c3_g1_i10_m.2564126	translation elongation factor aef-2	0.20	0.0112	GO:0006414;GO:0005622;GO:0003746;GO:0003924;GO:0005525
TRINITY_DN375838_c2_g1_i1_m.1093500	Serine/threonine-protein phosphatase	0.31	0.0325	GO:0015992;GO:0055085;GO:0016021;GO:0009678;GO:0004427
TRINITY_DN388968_c4_g1_i2_m.1606349	Chalcone synthase A	0.68	0.0300	GO:0009813;GO:0016747;GO:0042802
TRINITY_DN364974_c0_g2_i3_m.3336554	heme peroxidase	0.38	0.0352	GO:0006979;GO:0098869;GO:0055114;GO:0016021;GO:0020037;GO:0004601
TRINITY_DN376636_c0_g1_i8_m.1941000	translation elongation factor 1A-like	0.37	0.0447	GO:0006414;GO:0003746;GO:0003924;GO:0005525
TRINITY_DN178778_c0_g1_i2_m.529235	flagellar motor protein MotB	0.64	0.0089	GO:0016020
TRINITY_DN83834_c0_g1_i1_m.4548553	30S ribosomal protein S2	0.52	0.0004	GO:0006412;GO:0015935;GO:0003735
TRINITY_DN267350_c0_g1_i1_m.3830240	F0F1 ATP synthase subunit alpha	0.63	0.0082	GO:0015991;GO:0042777;GO:0045261;GO:0005886;GO:0046933;GO:0046961;GO:0005524
TRINITY_DN359424_c2_g4_i1_m.1988494	40S ribosomal protein S25	0.61	0.0064	GO:0005840
TRINITY_DN333361_c0_g1_i1_m.2601454	ATP synthase F0 subunit 1	0.55	0.0413	GO:0015986;GO:0015991;GO:0045261;GO:0005739;GO:0046933;GO:0046961;GO:0005524
TRINITY_DN587219_c0_g1_i1_m.3752731	translation elongation factor TU	0.66	0.0009	GO:0006414;GO:0005737;GO:0003746;GO:0003924;GO:0005525
TRINITY_DN374297_c0_g1_i9_m.2302768	Pyrophosphate-energized vacuolar membrane proton pump/Pyrophosphate-energized inorganic pyrophosphatase	0.64	0.0068	GO:0015992;GO:0055085;GO:0016021;GO:0005774;GO:0004427;GO:0009678;GO:0046872
TRINITY_DN382690_c2_g1_i2_m.919730	xylanase inhibitor protein 1-like	0.69	0.0480	GO:0006032;GO:0005975;GO:0005576;GO:0004568;GO:0008061
TRINITY_DN859512_c0_g1_i1_m.3426681	pilin	0.59	0.0382	GO:0007155;GO:0016021;GO:0009289
TRINITY_DN359316_c1_g1_i1_m.2479284	Propionyl-CoA carboxylase beta chain	0.65	0.0117	GO:0006633;GO:0009317;GO:0016740;GO:0003989
TRINITY_DN399461_c2_g1_i1_m.1289262	cysteine proteinase inhibitor	0.76	0.0466	GO:0010951;GO:0004869

TRINITY_DN380077_c0_g2_i5_m.1475262	dnaJ homolog subfamily B member 4-like	0.60	0.0242	GO:0006457;GO:0051082
TRINITY_DN350608_c0_g2_i1_m.1963834	60S ribosomal protein L17-2	0.62	0.0304	GO:0006412;GO:0015934;GO:0003735;GO:0003677
TRINITY_DN376991_c0_g5_i1_m.1059960	protein phosphatase 2C 45-like protein	0.45	0.0300	GO:0006470;GO:0016021;GO:0046872;GO:0004722
TRINITY_DN337729_c0_g2_i1_m.1456618	calmodulin	0.65	0.0029	GO:0005509
TRINITY_DN378851_c1_g1_i3_m.3294710	serine/threonine-protein kinase STY46	0.83	0.0228	GO:0006468;GO:0009658;GO:0005829;GO:0004712;GO:0016597;GO:0005524
TRINITY_DN631878_c0_g1_i1_m.27984	pyruvate decarboxylase 1	0.50	0.0439	GO:0000287;GO:0030976;GO:0004737
TRINITY_DN393034_c3_g1_i3_m.1155736	protein disulfide isomerase	0.81	0.0467	GO:0045454;GO:0034975;GO:0009960;GO:0005788;GO:0003756
TRINITY_DN373530_c1_g2_i6_m.1435364	signal recognition particle receptor subunit alpha	0.77	0.0118	GO:0006614;GO:0005785;GO:0005047;GO:0003924;GO:0005525
TRINITY_DN397056_c2_g2_i3_m.2503287	2,3-bisphosphoglycerate-independent phosphoglycerate mutase	0.80	0.0181	GO:0006007;GO:0006096;GO:0005737;GO:0004619;GO:0030145
TRINITY_DN394125_c2_g2_i1_m.1898221	22.3 kDa class VI heat shock protein	0.69	0.0302	GO:0009408;GO:0009644;GO:0042542;GO:0005739
TRINITY_DN379329_c0_g2_i4_m.1905873	aarF domain-containing protein kinase 4	0.76	0.0332	GO:0005739
TRINITY_DN352682_c0_g1_i3_m.1147563	vacuolar proton translocating atpase a	0.82	0.0407	GO:0070072;GO:0015986;GO:0007035;GO:0015991;GO:0016021;GO:0000220;GO:0051117;GO:0046961
TRINITY_DN350478_c1_g1_i4_m.2571987	60S ribosomal protein L23	0.68	0.0054	GO:0000184;GO:0006610;GO:0006614;GO:0019083;GO:0006364;GO:0006413;GO:0005730;GO:0070062;GO:00226 25;GO:0016020;GO:0005925;GO:0070180;GO:0044822;GO:0003735;GO:0005515
TRINITY_DN390545_c2_g1_i1_m.1820670	aldehyde dehydrogenase family 2 member C4	0.73	0.0388	GO:0055114;GO:0009699;GO:0004029;GO:0050269
TRINITY_DN368929_c2_g1_i2_m.3149760	xyloglucan endotransglycosylase	0.60	0.0410	GO:0042546;GO:0071555;GO:0010411;GO:0005618;GO:0048046;GO:0016762;GO:0004553
TRINITY_DN376972_c0_g2_i8_m.1061690	aci-reductone-dioxygenase-like protein	0.77	0.0042	GO:0019509;GO:0055114;GO:0005737;GO:0005634;GO:0010309;GO:0005506
TRINITY_DN390574_c0_g1_i2_m.1824163	hexokinase-7	0.82	0.0180	GO:0046835;GO:0051156;GO:0001678;GO:0006096;GO:0005829;GO:0008865;GO:0004340;GO:0019158;GO:00055 24;GO:0005536
TRINITY_DN387970_c0_g1_i8_m.1827634	3-oxoacyl-[acyl-carrier-protein] synthase I/Beta-ketoacyl-ACP synthase I	0.81	0.0247	GO:0006633;GO:0009507;GO:0004315
TRINITY_DN386500_c4_g2_i19_m.1442740	phosphoglycerate kinase	0.80	0.0342	GO:0006096;GO:0009570;GO:0005829;GO:0005634;GO:0005774;GO:0048046;GO:0009506;GO:0005886;GO:00046 18;GO:0005524
TRINITY_DN398753_c1_g1_i3_m.1998646	Serine/threonine-protein kinase CTR1	0.65	0.0307	GO:0006468;GO:0009536;GO:0004712;GO:0005524
TRINITY_DN399027_c5_g1_i1_m.2650400	putative nematode-resistance protein	0.82	0.0140	GO:0006952
TRINITY_DN200849_c0_g1_i1_m.3949917	succinyl-CoA ligase subunit alpha	0.78	0.0364	GO:0006105;GO:0006104;GO:0006099;GO:0009142;GO:0005829;GO:0005759;GO:0048037;GO:0004775;GO:00047 76
TRINITY_DN334125_c1_g1_i1_m.1216841	Haloacid dehalogenase-like hydrolase domain-containing protein 3	0.79	0.0342	GO:0009269
TRINITY_DN301992_c0_g1_i1_m.1403753	Cortical cell-delineating protein	0.81	0.0188	GO:0006869;GO:0008289
TRINITY_DN594738_c0_g1_i1_m.3663534	alpha-L-arabinofuranosidase	0.39	0.0032	GO:0045493;GO:0031176;GO:0030247
TRINITY_DN395696_c2_g1_i3_m.2553554	fructose-bisphosphate aldolase cytoplasmic isozyme	0.70	0.0168	GO:0006096;GO:0005737;GO:0004332
TRINITY_DN397541_c1_g1_i3_m.2299318	glucose-6-phosphate isomerase	0.82	0.0421	GO:0009744;GO:0009817;GO:0009813;GO:0006096;GO:0010224;GO:0006094;GO:0005829;GO:0004347
TRINITY_DN343551_c0_g1_i4_m.2713924	50S ribosomal protein L36e	0.58	0.0213	GO:0002181;GO:0022625;GO:0003735
TRINITY_DN386690_c0_g1_i1_m.988170	papain-like cysteine proteinase	0.75	0.0464	GO:0051603;GO:0005764;GO:0005615;GO:0004197
TRINITY_DN394931_c1_g1_i2_m.2425637	Alanine aminotransferase 2/Glutamate pyruvate transaminase 2/Glutamic--alanine transaminase 2	0.81	0.0091	GO:0009058;GO:0042853;GO:0030170;GO:0004021
TRINITY_DN362866_c0_g1_i1_m.1616217	26S proteasome non-ATPase regulatory subunit 5	0.78	0.0106	GO:0070682;GO:0008540
TRINITY_DN388845_c2_g4_i2_m.1727158	endotransglucosylase/hydrolase XTH5	0.72	0.0349	GO:0042546;GO:0071555;GO:0010411;GO:0005618;GO:0048046;GO:0016762;GO:0004553

TRINITY_DN378399_c1_g1_i2_m.862480	anthocyanidin 5,3-O-glucosyltransferase-like	0.80	0.0080	GO:0009813;GO:0052696;GO:0043231;GO:0080043;GO:0080044
TRINITY_DN371471_c0_g1_i3_m.1716633	glucose-6-phosphate 1-dehydrogenase 2	0.77	0.0232	GO:0006098;GO:0006006;GO:0055114;GO:0009536;GO:0005739;GO:0050661;GO:0004345
TRINITY_DN394931_c1_g1_i7_m.2425667	Glutamate pyruvate transaminase 2	0.78	0.0087	GO:0009058;GO:0042853;GO:0030170;GO:0004021
TRINITY_DN391233_c0_g1_i2_m.2778705	A Chain A, Crystal Structure Of Barley Thioredoxin H Isoform 1 Crystallized Using Ammonium Sulfate As Precipitant	0.79	0.0094	GO:0006662;GO:0006457;GO:0043086;GO:0010497;GO:0009409;GO:0045454;GO:0000103;GO:0034599;GO:0055114;GO:0005829;GO:0005886;GO:0015035;GO:0004857;GO:0016671
TRINITY_DN381158_c0_g1_i1_m.2747759	50S ribosomal protein L1	0.83	0.0169	GO:0006412;GO:0006354;GO:0000470;GO:0006508;GO:0022625;GO:0005739;GO:0003735;GO:0003723;GO:0008234
TRINITY_DN393067_c1_g1_i5_m.1152592	AAA-ATPase ASD	0.71	0.0440	GO:0051301;GO:0016021;GO:0005524
TRINITY_DN399937_c12_g1_i1_m.1630567	putative disease resistance RPP13-like protein 3	0.82	0.0326	GO:0000166
TRINITY_DN352112_c6_g2_i2_m.1454965	60S ribosomal protein L12	0.76	0.0419	GO:0006412;GO:0000027;GO:0022625;GO:0003735;GO:0019843
TRINITY_DN399048_c4_g1_i2_m.2648918	MAP3K epsilon protein kinase 1-like isoform X4	0.80	0.0207	GO:0023014;GO:0005737;GO:0005524;GO:0004702
TRINITY_DN378143_c0_g1_i2_m.2683410	SKP1-interacting partner 15	0.79	0.0243	GO:0016021
TRINITY_DN750625_c0_g1_i1_m.4221283	ubiquinol-cytochrome c reductase iron-sulfur subunit	0.80	0.0007	GO:1902600;GO:0055114;GO:0045275;GO:0051537;GO:0008121;GO:0046872;GO:0009055
TRINITY_DN377528_c0_g2_i4_m.1936772	triosephosphate isomerase, cytosolic	0.81	0.0248	GO:0019563;GO:0046166;GO:0006096;GO:0006094;GO:0005829;GO:0004807
TRINITY_DN367659_c0_g1_i4_m.1317034	universal stress protein 1561	0.77	0.0372	GO:0006950;GO:0002238;GO:0005773
TRINITY_DN371784_c2_g1_i1_m.2598011	Peroxidase 2	0.75	0.0247	GO:0098869;GO:0042744;GO:0006979;GO:0055114;GO:0005576;GO:0020037;GO:0004601;GO:0046872
TRINITY_DN398561_c3_g1_i7_m.1122061	probable E3 ubiquitin-protein ligase ARI8	0.77	0.0025	GO:0032436;GO:0042787;GO:0000209;GO:0000151;GO:0005739;GO:0031624;GO:0016874;GO:0008270;GO:0061630
TRINITY_DN376757_c3_g1_i4_m.2857885	50S ribosomal protein L9	0.78	0.0402	GO:0006412;GO:0005840;GO:0005739;GO:0003735
TRINITY_DN396498_c0_g1_i11_m.2067224	pyrophosphate--fructose 6-phosphate 1-phosphotransferase subunit alpha-like	0.78	0.0401	GO:0046835;GO:0006002;GO:0061615;GO:0009536;GO:0047334;GO:0003872;GO:0005524
TRINITY_DN386536_c0_g1_i11_m.2902657	Hydroxymethylglutaryl-CoA synthase	0.80	0.0407	GO:0009793;GO:0019287;GO:0005829;GO:0009506;GO:0004421
TRINITY_DN365340_c2_g1_i2_m.1112976	Cytochrome P450 71D7	0.68	0.0063	GO:0044550;GO:0055114;GO:0016021;GO:0020037;GO:0016709;GO:0005506
TRINITY_DN380354_c4_g1_i1_m.1210837	serine/threonine-protein phosphatase PP1	0.75	0.0328	GO:0006470;GO:0008287;GO:0046872;GO:0004722
TRINITY_DN386362_c1_g1_i1_m.1127904	guanine nucleotide-binding protein subunit beta-like protein A	0.81	0.0068	GO:0060267;GO:0050832;GO:0007165;GO:0005737;GO:0005886;GO:0004871;GO:0005515
TRINITY_DN399807_c0_g1_i1_m.2045126	Clathrin heavy chain 1	0.70	0.0196	GO:0006886;GO:0016192;GO:0030130;GO:0030132;GO:0005198
TRINITY_DN355991_c1_g1_i1_m.3350299	Pseudouridine-5'-monophosphatase		0.0183	GO:0008152;GO:0016787
TRINITY_DN265056_c0_g1_i1_m.3936482	Phosphoethanolamine N-methyltransferase	0.81	0.0221	GO:0032259;GO:0005737;GO:0008757
TRINITY_DN395594_c2_g3_i7_m.3044603	Cysteine-rich receptor-like protein kinase 10	0.82	0.0454	GO:0016310;GO:0000166;GO:0004672;GO:0043167
TRINITY_DN393136_c1_g1_i3_m.1552005	hypersensitive-induced response protein 1	0.72	0.0181	GO:0016020
TRINITY_DN353777_c1_g2_i1_m.2895556	Rab GDP dissociation inhibitor alpha	0.79	0.0227	GO:0050790;GO:0015031;GO:0007264;GO:0055114;GO:0005622;GO:0005093;GO:0016491
TRINITY_DN260710_c0_g1_i1_m.3839235	Peroxidase 72	0.53	0.0266	GO:0000287;GO:0030976;GO:0004737
TRINITY_DN359715_c0_g1_i4_m.1514143	Eukaryotic translation initiation factor 5	0.81	0.0190	GO:0006413;GO:0003743;GO:0005525
TRINITY_DN398213_c4_g1_i11_m.1381268	beta-glucosidase BoGH3B	0.78	0.0492	GO:0009251;GO:0004338
TRINITY_DN363721_c0_g1_i2_m.3274340	1,2-dihydroxy-3-keto-5-methylthiopentene dioxygenase 4	0.82	0.0203	GO:0019509;GO:0055114;GO:0005737;GO:0005634;GO:0010309;GO:0005506

TRINITY_DN405987_c0_g1_i1_m.674407	Glucokinase	0.15	0.0002	------
TRINITY_DN386362_c3_g2_i9_m.1127977	guanine nucleotide-binding protein subunit beta-like protein	0.32	0.0151	------
TRINITY_DN217568_c0_g1_i2_m.3969338	TonB-dependent receptor	0.48	0.0379	------
TRINITY_DN385076_c1_g2_i1_m.2193347	Universal stress protein A-like protein	0.70	0.0045	------
TRINITY_DN348454_c0_g1_i1_m.2983067	putative polygalacturonase inhibitor	0.80	0.0008	------

DEPs: Differentially expression proteins, AS:alkali stress(35mmol.L^{-1} $NaCO_3$:$NaHCO_3$=1:1), AS+Spm:35mmol.L^{-1} $NaCO_3$:$NaHCO_3$(1:1)+0.01mmol.L^{-1} spermine.
