# Supplementary material for: Proteomic Responses to Alkali Stress in Oats and the Alleviatory Effects of Exogenous Spermine Application
Source: Front Plant Sci. 2021 Apr 1;12:627129. doi: 10.3389/fpls.2021.627129 (PMC8049610; doi:10.3389/fpls.2021.627129)
Supplement: Supplementary file 15 [file Data_Sheet_1.PDF]

Supplemental Materials and Methods S1 The parameters related to the determination of IAA, ABA, JA, ACC

	Molecular Weight (Da)	Ion mode	Ionization model	Q1 (Da)	Q3 (Da)	Rt (min)
IAA	175.18	Positive	[M+H]+	176.1	130.1	4.79
ABA	264.32	Negative	[M-H]-	263.1	153.1	5.03
JA	210.27	Negative	[M-H]-	209.1	58.8	5.54
ACC	348.393	Positive	[M+H]+	102.2	56	0.78
