# Supplementary material for: Proteomic Responses to Alkali Stress in Oats and the Alleviatory Effects of Exogenous Spermine Application
Source: Front Plant Sci. 2021 Apr 1;12:627129. doi: 10.3389/fpls.2021.627129 (PMC8049610; doi:10.3389/fpls.2021.627129)
Supplement: Supplementary file 16 [file Data_Sheet_2.pdf]

Supplemental Materials and Methods S2 The primers used for Rt-qPCR

Gene name	Size of products		Sequence
Actin	155	F	GTAAGGGACATCAAGGAGAAGC
		R	ACCTCAGGGCAACGGAAC
Sucrose synthase type 3	147	F	AACCCTAAGCTAAGGGAGAACG
		R	CCAACGGAACTGCCCAAAC
aquaporin PIP1-1	186	F	GGATGAGCGGGAGGTAAGTT
		R	CGTGGTTGCGTAGAGCGT
Coproporphyrinogen III oxidase	135	F	TGAAGCATTTTCATTCTGTCCA
		R	ATATTCCGCCAAGCCCAC
ATP-dependent zinc metalloprotease FTSH 8	121	F	CTCTGCGGGAAGGATAGGA
		R	TGATTGGAAGAATCTCGACTGG
ruBisCO large subunit-binding protein subunit beta	145	F	GCCCCAGAAAATCAGTCCC
		R	ACCAAGAGTAACCCCAACCAG
protoporphyrinogen oxidase	173	F	CCCTGTATTTGTAGCACCCC
		R	TTAGAAAAGAATGCCTTGTTGG
cytochrome f	134	F	CTCCTCCCAGGCAAAATAAGA
		R	AAGCTGTGCTCCCCGATAC
Photosystem II 10 kDa polypeptide	166	F	TCCTTGAAATCCGCTCCTC
		R	TCATGGCTTCGTTGGCTC
polyphenol oxidase	118	F	TCTCGTACTTGTCCAGGTACTGC
		R	AACTGCTGCCCAGCGTTC
glucan endo-1,3-beta-glucosidase, acidic isoform	139	F	CGACTACGCCCTCTTCACC
		R	AACGATGCCGACGTTTCC
